# Immunogenicity and Safety of RSVPreF3 OA Coadministration Versus Sequential Administration in Adults: A Systematic Review and Meta-Analysis

**DOI:** 10.3390/vaccines14090826

**Published:** 2026-09-20

**Authors:** Shu Jiang, Yan Liu, Ran Cui

**Affiliations:** 1 Department of Health Services, School of Basic Medicine, Neijiang Health Vocational College, Neijiang 641000, China; 2 Department of Respiratory and Critical Care Medicine, First People’s Hospital of Neijiang, Neijiang 641000, China

**Keywords:** respiratory syncytial virus vaccine, coadministration, sequential administration, non-inferiority, adults aged 50 years or older, systematic review, meta-analysis, immunogenicity, safety

## Abstract

Background/Objectives: Adults eligible for respiratory syncytial virus (RSV) vaccination may also receive influenza, COVID-19, pneumococcal, or herpes zoster vaccines. We compared immunogenicity and safety between coadministration and sequential schedules. Methods: We searched PubMed, Embase, Web of Science, Scopus, and CENTRAL from inception to 3 August 2026 for randomized trials in adults aged 50 years or older. Eligible trials compared RSVPreF3 OA coadministration with sequential administration of the partner vaccine followed by RSVPreF3 OA. Random-effects models pooled neutralizing-antibody geometric mean ratios (GMRs; coadministration/sequential) separately for RSV-A and RSV-B and risk ratios for safety outcomes. Risk of bias and certainty were assessed using RoB 2 and GRADE, respectively. Results: Six trials randomized 5455 participants. Pooled RSV-A and RSV-B GMRs were 0.895 (95% CI 0.816–0.983) and 0.922 (95% CI 0.837–1.016), respectively. In trial-specific per-protocol analyses, non-inferiority criteria were met for recombinant zoster vaccine (RZV) and 20-valent pneumococcal conjugate vaccine (PCV20) antibody endpoints, but non-inferiority was not demonstrated for SARS-CoV-2 XBB.1.5 neutralizing antibodies (GMR 0.763, 95% CI 0.662–0.885) or the adjuvanted quadrivalent influenza vaccine A/H3N2 response. The pooled risk ratio for serious adverse events was 0.831 (95% CI 0.530–1.303), with limited precision. Certainty was low for RSV-A and very low for RSV-B, mainly reflecting risk of bias and inconsistency. Conclusions: In adults aged 50 years or older, same-day RSVPreF3 OA coadministration was associated with lower RSV-neutralizing antibody responses than sequential administration, while partner-vaccine immunogenicity varied by product and antigen. Non-inferiority was not demonstrated for the SARS-CoV-2 XBB.1.5 and adjuvanted-QIV A/H3N2 antibody endpoints. No clear safety difference between schedules was identified, and the clinical significance of the antibody differences remains uncertain. Registration: PROSPERO CRD420261470812.

## 1. Introduction

Respiratory syncytial virus (RSV) is a common respiratory pathogen that can cause severe lower respiratory tract illness in older adults, particularly those with underlying chronic conditions [1]. In high-income countries, RSV was estimated to cause approximately 470,000 hospitalizations and 33,000 in-hospital deaths among adults aged 60 years and older in 2019 [2]. A separate meta-analysis found higher RSV-associated hospital admission and in-hospital case-fatality rates in adults aged 65 years or older than in those aged 50–64 years [3].

RSV vaccines for older adults have been evaluated in phase 3 trials, and several products have been shown to reduce the risk of RSV-associated lower respiratory tract disease in this age group [4,5,6]. Current US guidance recommends a single dose of RSV vaccine for all adults aged 75 years or older and for adults aged 50–74 years who are at increased risk of severe RSV disease [7]. Adults eligible for RSV vaccination may also be eligible for influenza, COVID-19, pneumococcal, or herpes zoster vaccination. Coadministration can reduce the number of vaccination visits and may facilitate vaccine uptake [8,9,10]. Previous reviews have examined adult vaccine coadministration [11], but its effects on the immune response to each vaccine require evaluation for the specific vaccine combination.

Randomized non-inferiority trials have directly compared same-day coadministration with sequential administration, prompted by the concern that concurrent vaccination could modulate the immune response to either vaccine [12]. Comparable trials of other RSV vaccine platforms, including an RSVpreF vaccine coadministered with inactivated influenza vaccine and an mRNA-based RSV vaccine coadministered with influenza or COVID-19 vaccines, have reported non-inferiority for most primary immunogenicity endpoints according to the trials’ own criteria [13,14]. For RSVPreF3 OA, six randomized trials have each compared same-day coadministration with sequential administration for one of six partner vaccines (pneumococcal [15], COVID-19 [16], herpes zoster [17], or influenza [18,19,20]). Existing pooled analyses, by contrast, have either combined coadministration data across respiratory virus vaccines with different vaccines, outcomes, and non-inferiority definitions [21] or have synthesized RSV vaccine immunogenicity and safety without comparing coadministration with sequential administration [22,23]. These analyses do not provide a synthesis focused on RSVPreF3 OA coadministration across partner vaccines.

Accordingly, this systematic review and meta-analysis aims to compare same-day coadministration of RSVPreF3 OA with an indicated partner vaccine against sequential administration for both immunogenicity and safety, and to synthesize evidence across the evaluated partner vaccines. This review includes six randomized non-inferiority trials covering pneumococcal, COVID-19, herpes zoster, and influenza partner vaccines. RSV-neutralizing antibody responses, partner-vaccine immune responses, and safety outcomes are compared between the two administration strategies. By integrating evidence across the six partner vaccines, the findings are intended to inform scheduling decisions for adults aged 50 years or older receiving the evaluated vaccine combinations.

## 2. Materials and Methods

The review protocol was registered in PROSPERO (CRD420261470812) before the literature search was conducted. This review is reported in accordance with the PRISMA 2020 statement [24]; the search process follows the PRISMA-S extension [25], and the corresponding checklists are provided as Supplementary Files.

### 2.1. Search Strategy

We searched PubMed, Embase, the Cochrane Central Register of Controlled Trials, Web of Science, and Scopus from inception to 3 August 2026 using a combination of database-specific controlled vocabulary and free-text terms (full strategies in the Supplementary PRISMA-S appendix). Registry reports were used for protocol verification, trial characteristics, and study-completion data. Reference lists of retrieved records and relevant reviews were screened for additional studies. No language limits were applied in the searches.

### 2.2. Study Selection

Eligibility criteria followed the PICOS framework. Studies were eligible if they did the following: enrolled adults scheduled to receive an RSV vaccine and a specified partner vaccine, at an age threshold prespecified in each trial protocol (Population); evaluated RSVPreF3 OA (120 μg, intramuscular) coadministered with the partner vaccine on the same day (Intervention); compared coadministration with sequential administration, defined as the partner vaccine first and the RSV vaccine approximately 1 month later (Comparison); reported the outcomes defined in Section 2.3 (Outcomes); and were randomized controlled trials comparing coadministration with sequential administration (Study design). We excluded studies of other RSV vaccine platforms, non-randomized studies, and designs without a coadministration-versus-sequential comparison. Preprints were ineligible. Conference abstracts were linked as companion reports of included trials. Eligibility was not restricted by publication language.

Two reviewers independently screened titles and abstracts and assessed potentially eligible reports against the eligibility criteria; disagreements were resolved by discussion or by a third reviewer. Screening decisions and exclusion reasons were recorded and verified against the source reports. Reports were linked to the same trial using registration numbers and bibliographic information. Duplicate records of the same report were removed, and distinct reports of the same trial were grouped under a single study identifier.

### 2.3. Data Extraction and Outcome Measures

Data were extracted independently by two reviewers from the primary trial articles and their supplements, with registry participant-flow data for study-completion outcomes, using a piloted electronic form covering study characteristics, dosing schedules, outcome definitions, effect estimates and precision, and arm-level denominators; discrepancies were resolved by discussion or a third reviewer. Outcomes not reported in a trial were excluded from the corresponding analysis; no imputation was performed. Reported adjusted ratios were standardized to coadministration divided by sequential administration, and arm-level pre- and post-vaccination geometric mean titers or concentrations and geometric mean increases were extracted as reported. Log-GMR standard errors were derived from reported confidence intervals, and log risk-ratio variances were calculated from participant counts. Calculation details are provided in the Supplementary Methods. The pooled immunogenicity estimates used the trial-defined per-protocol sets; reported estimates from broader analysis populations were also extracted and compared with the per-protocol results.

The primary outcome was the ratio of geometric means (GMR) of RSV-neutralizing antibody titers (coadministration relative to sequential administration) at approximately one month after the RSV dose, as defined by each trial. Effect estimates were retained at the RSV-A and RSV-B subtype level. Only neutralizing antibody data reported on the ED60 assay scale were pooled. Secondary outcomes comprised partner-vaccine immunogenicity (influenza HI, PCV20 OPA, COVID-19 neutralizing antibody, and RZV anti-gE) and safety. Safety outcomes included serious adverse events and deaths through study end, grade 3 unsolicited adverse events, 4-day solicited local and systemic reactions, study non-completion for any reason and study completion, and 30-day unsolicited adverse events. Unsolicited adverse events, including grade 3 events, were assessed as the proportion of participants reporting at least one event across each schedule’s planned reporting windows. Denominators and schedule-specific reporting windows are defined in the Supplementary Methods.

For each trial, we extracted the prespecified non-inferiority margins, analysis populations, testing hierarchy, and reported conclusions. For RSV-neutralizing antibodies, the 1.5-fold margin corresponds to a boundary of 1/1.5 (approximately 0.667) for the coadministration-to-sequential GMR; the documented origins of trial-specific margins are summarized in the Supplementary Methods. Non-inferiority conclusions were retained within each trial’s original testing framework, and pooled GMRs summarized between-schedule differences [26,27].

### 2.4. Quality Assessment

Risk of bias and certainty of evidence were assessed independently by two reviewers using the revised Cochrane Risk-of-Bias tool for randomized trials (RoB 2) [28] and the GRADE framework [29], respectively; disagreements were resolved by consensus or a third reviewer. RoB 2 judgments addressed the effect of assignment to the vaccination schedule for each assessed result. Assessments were outcome-specific for RSV-A, RSV-B, partner-vaccine antibody responses, and the safety outcomes. Trial reports, protocols, and statistical analysis plans informed these assessments. Detailed assessment methods and judgments are provided in the Supplementary Materials and Supplementary Data and Code.

Certainty of evidence was rated across the five GRADE domains. Summary findings are presented in the main text, with detailed domain judgments in the Supplementary Materials. Product-specific partner-vaccine antibody endpoints were graded separately in the Supplementary Materials. Formal tests for funnel-plot asymmetry were omitted when fewer than ten studies contributed to a pooled analysis, and the publication-bias domain was rated qualitatively. Trial funding and sponsor roles were considered alongside registration and outcome reporting in this assessment.

### 2.5. Data Analysis

RSV-A and RSV-B neutralizing-antibody GMRs were pooled separately using random-effects models with restricted maximum likelihood estimation and Hartung–Knapp confidence intervals. Each trial contributed one estimate per subtype. Log-transformed GMRs were pooled and back-transformed to the ratio scale.

An exploratory three-level joint model combined the RSV-A and RSV-B estimates, with random effects for studies and endpoints within studies. The within-study sampling covariance was specified using a working correlation of ρ = 0.5. Confidence intervals for the joint estimate used study-clustered CR2 variance estimation and Satterthwaite degrees of freedom. Sensitivity analyses of the joint summary examined working correlations of ρ = 0 and 0.8, study-level composites pooled using the Paule–Mandel estimator with Hartung–Knapp adjustment, and fixed-effects generalized least squares. Further analyses omitted one trial at a time and excluded the seasonal-QIV RSV-B subset estimate and the adjuvanted-QIV RSV-B estimate that was no longer confirmatory after hierarchical testing stopped.

Subtype-specific heterogeneity was summarized using Q, the REML between-study variance, and conventional I^2^. For the joint model, the Q statistic was calculated using the specified sampling variance–covariance matrix. A descriptive I^2^ proxy was calculated as the sum of the study- and endpoint-level variance components divided by that sum plus the mean sampling variance. Full model specifications and prediction-interval calculations are provided in the Supplementary Methods.

Partner-vaccine immunogenicity was presented by product and antigen against each trial’s own non-inferiority margin. An exploratory cross-product A/H1N1 summary using a random-effects REML model with Hartung–Knapp adjustment is reported in Results Section 3.4 and the Supplementary Materials. Safety risk ratios were pooled using random-effects REML models with Hartung–Knapp confidence intervals. For studies with a zero cell, a fixed continuity correction of 0.5 was added to all four cells of the 2 × 2 table. Sensitivity analyses used a correction of 0.1 and a risk-difference model. Studies with zero events in both arms were retained, and continuity-corrected estimates were reported alongside crude event counts.

Pooled estimates were reported with two-sided 95% confidence intervals. A significance level of 0.05 was used for heterogeneity tests. Secondary and exploratory analyses were descriptive, with no adjustment for multiplicity. Analyses were performed in R (version 4.5.3) using the metafor package (version 4.8-0) and the clubSandwich package (version 0.7.0); Figures were generated using Python (version 3.12) and the R packages forestplot (version 3.2.0) and robvis (version 0.3.0.900). The analysis code is provided in Supplementary Data and Code.

## 3. Results

### 3.1. Search Results and Study Characteristics

**Figure 1:**
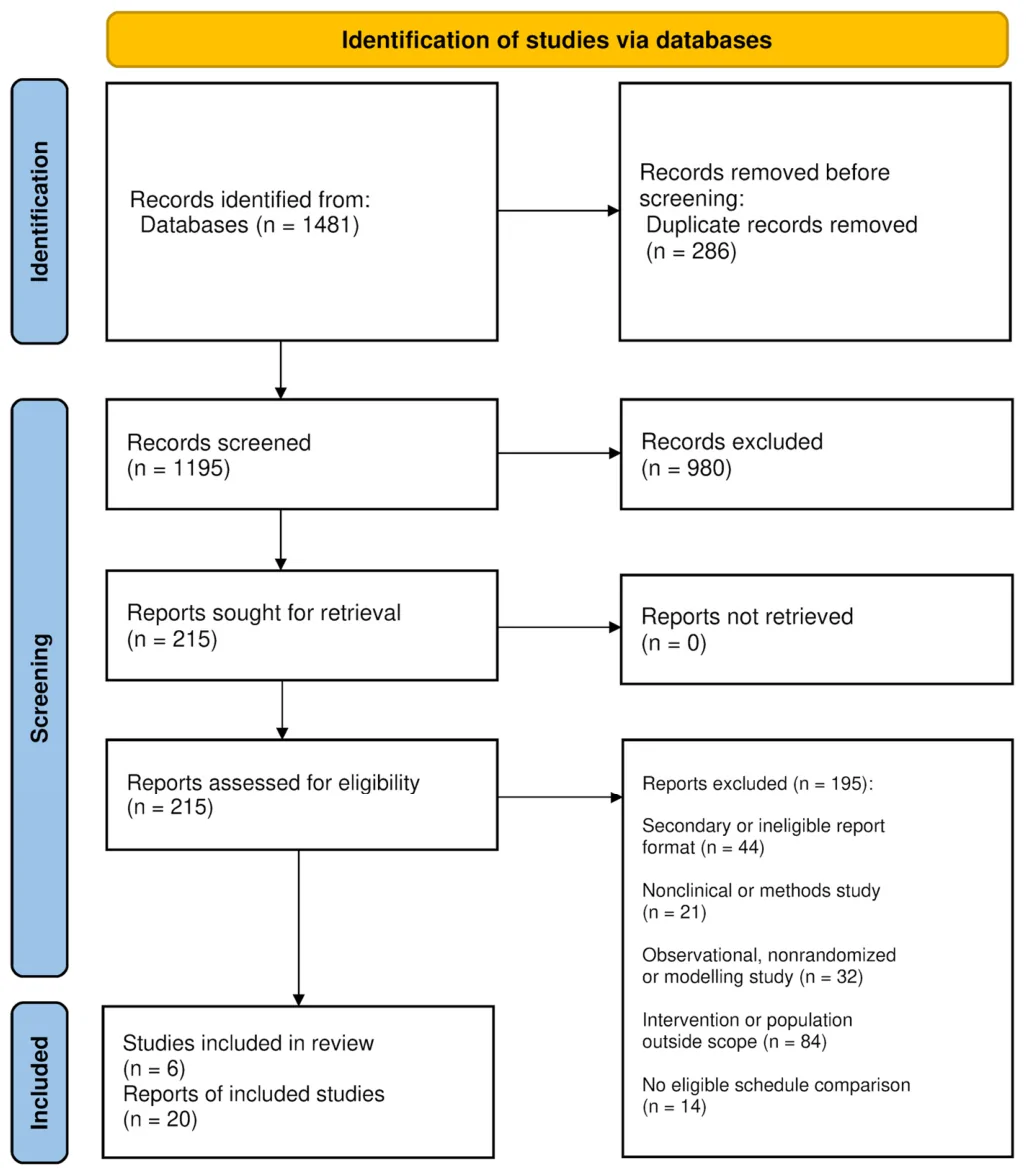
PRISMA 2020 flow diagram of study selection.

The database search identified 1481 records; after removal of 286 duplicates, 1195 records were screened, and 980 were excluded on title and abstract review. The remaining 215 reports were assessed for eligibility (Table S1), of which 195 were excluded for the reasons listed in Table S2. Six randomized trials were included (Figure 1), represented by 20 reports: six primary articles, nine registry reports, and five conference abstracts (Table S3). Each trial evaluated RSVPreF3 OA coadministered with one partner vaccine (PCV20, Comirnaty XBB.1.5, RZV, seasonal QIV, high-dose QIV, or adjuvanted QIV) (Table 1). Seventy-one records were reconciled to corrected report identities, source materials, and final dispositions; a total of 66 reports were excluded, and five were linked to the included trials (Table S4).

All six trials were randomized, open-label, phase 3, non-inferiority studies of AS01E-adjuvanted RSVPreF3 OA. Both vaccines were administered on Day 1 in the coadministration groups; the sequential groups received the partner vaccine on Day 1 and RSVPreF3 OA on Day 31. In the RZV trial, both groups received the second RZV dose on Day 61. Safety follow-up continued for 6 months after the last vaccination. Eligibility required age ≥50 years (two trials), ≥60 years (two trials), or ≥65 years (two trials). Sample sizes ranged from 530 to 1112 participants (Table 1). For the largest trial (PCV20), the publication reports 1112 randomized and 1110 exposed participants, whereas the trial-registry enrollment field lists 1113 (Table S5). All trials measured RSV-A and RSV-B neutralizing antibody responses approximately 1 month after RSV vaccination, partner-vaccine immunogenicity, and safety. Detailed trial characteristics are provided in Table S6.

### 3.2. RSV-A and RSV-B Subtype Analyses

**Figure 2:**
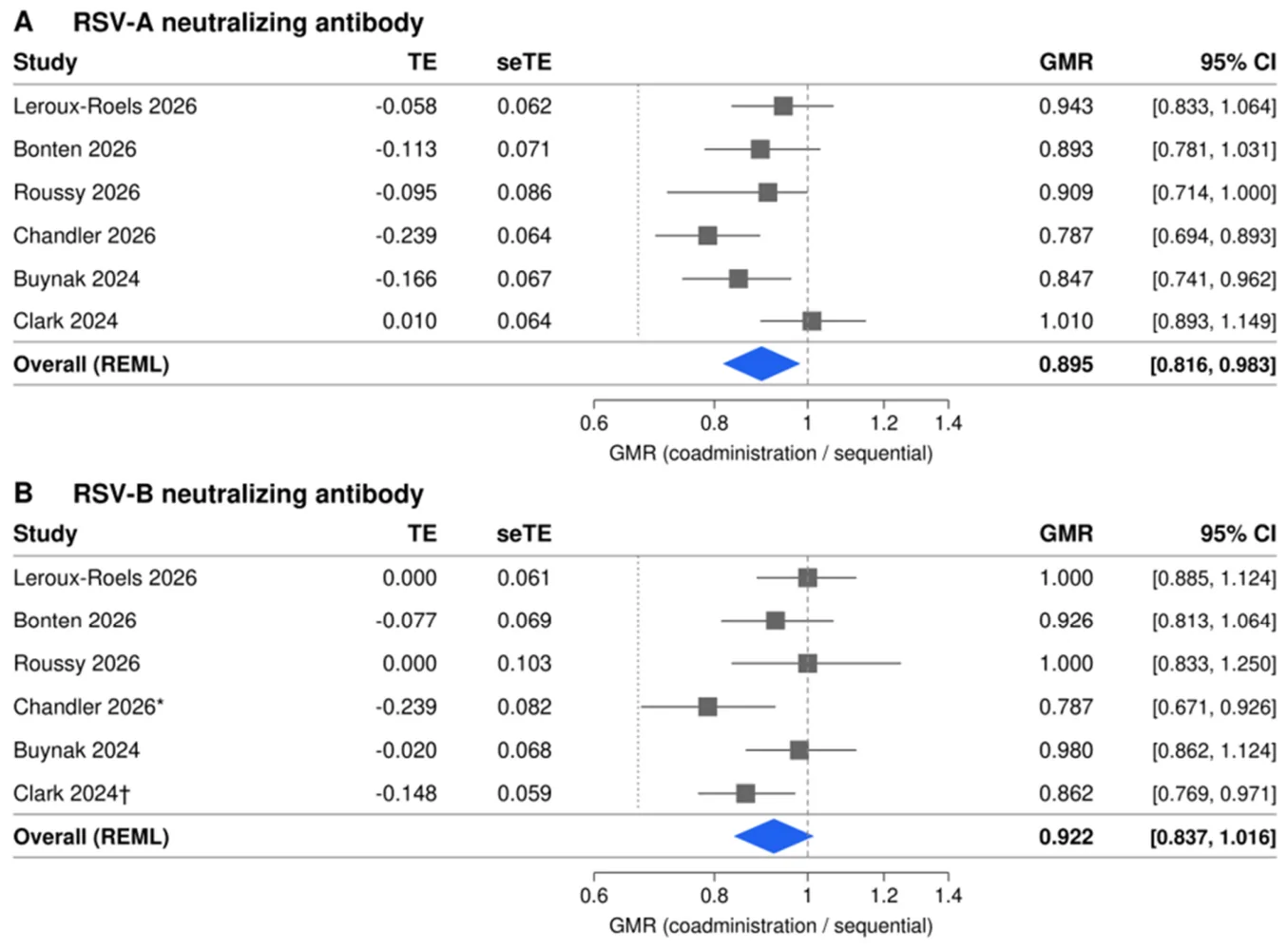
RSV-neutralizing antibody responses 1 month after vaccination: (**A**) RSV-A; (**B**) RSV-B. Diamonds indicate pooled GMRs with 95% CIs. Vertical lines indicate GMR = 1 (dashed) and the trial-level non-inferiority margin of 0.667 (dotted). * Secondary endpoint in a per-protocol subset (*n* = 425); † descriptive endpoint. TE, log(GMR); seTE, standard error of TE [15,16,17,18,19,20].

RSV-A and RSV-B subtypes were modelled separately as the principal pooled analyses (Figure 2A,B). The pooled RSV-A GMR was 0.895 (95% CI 0.816–0.983; I^2^ 45.0%) and the pooled RSV-B GMR was 0.922 (95% CI 0.837–1.016; I^2^ 38.1%). The RSV-A confidence interval was below 1, whereas the RSV-B interval included 1. Both approximate 95% prediction intervals crossed 1 (0.745–1.076 and 0.776–1.096, respectively).

The pooled estimates were based on the trial-defined per-protocol populations. In the seasonal-QIV trial, the RSV-B estimate was based on a per-protocol subset (*n* = 425) and was a secondary endpoint [18]. In the adjuvanted-QIV trial, RSV-B was evaluated descriptively after the preceding A/H3N2 test did not demonstrate non-inferiority [20]. Trial-level endpoint status and original conclusions are summarized in Table S7. Comparisons with reported exposed-set estimates are presented in Table S8, and arm-level antibody responses are reported in Tables S9 and S10.

### 3.3. Overall RSV Immunogenicity

**Figure 3:**
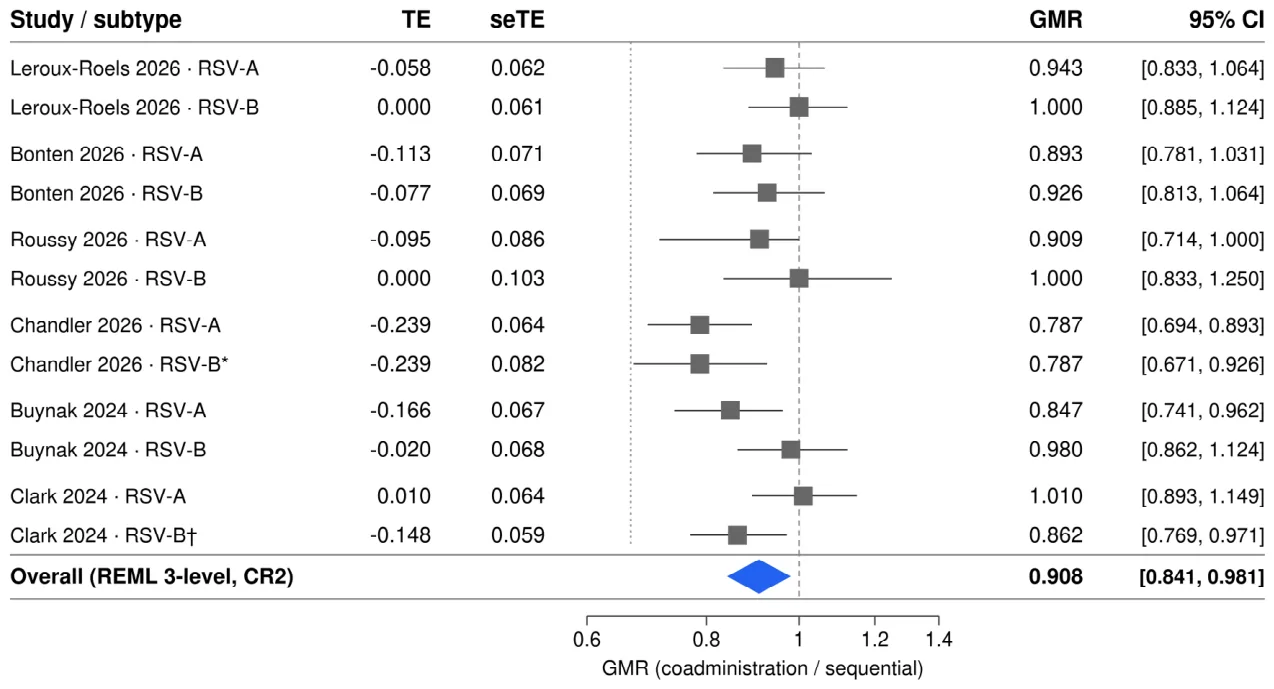
Exploratory joint analysis of RSV-neutralizing antibody responses 1 month after vaccination. Rows show 12 correlated RSV-A/RSV-B estimates from six trials; the diamond indicates the pooled GMR with its 95% CI. Vertical lines indicate GMR = 1 (dashed) and the trial-level non-inferiority margin of 0.667 (dotted). * Secondary endpoint in a per-protocol subset (*n* = 425); † descriptive endpoint. TE, log(GMR); seTE, standard error of TE [15,16,17,18,19,20].

RSV-neutralizing antibody responses approximately 1 month after vaccination were evaluated in an exploratory joint analysis of 12 subtype-specific estimates (RSV-A and RSV-B within each of the six trials), with the two subtypes treated as correlated endpoints nested within each study (Figure 3). The pooled RSV-neutralizing antibody GMR for coadministration versus sequential administration was 0.908 (95% CI 0.841–0.981; Satterthwaite df = 4.819). The point estimate corresponded to approximately 9% lower neutralizing-antibody titers with coadministration.

The joint model yielded Q(11) = 20.74 (*p* = 0.036), an I^2^ proxy of 37.2%, and a summed study- and endpoint-level variance of 0.0031. The approximate model-based 95% prediction interval was 0.802–1.027; this interval describes a subtype-specific true effect in a new study under the joint model and spans responses both below and above those with sequential administration. Variance components and prediction-interval calculations are provided in Table S11 and the Supplementary Methods.

### 3.4. Partner-Vaccine Immunogenicity

**Figure 4:**
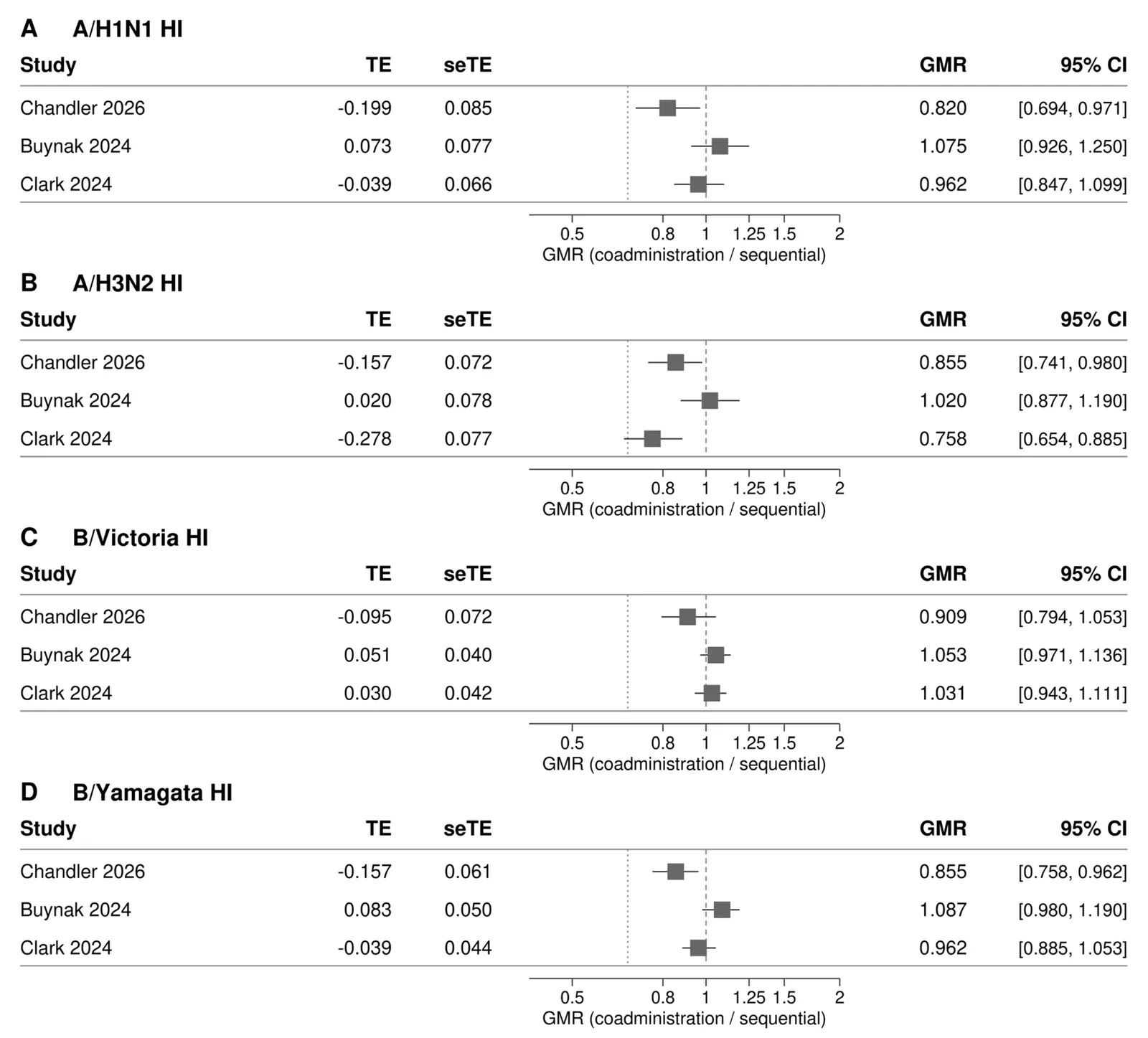
Influenza antibody responses 1 month after vaccination: (**A**) A/H1N1; (**B**) A/H3N2; (**C**) B/Victoria; (**D**) B/Yamagata. Vertical lines indicate GMR = 1 (dashed) and the trial-level non-inferiority margin of 0.667 (dotted). HI, hemagglutination inhibition; TE, log(GMR); seTE, standard error of TE [18,19,20].

A/H1N1 hemagglutination-inhibition (HI) antibody GMR was reported by the three influenza trials (Figure 4A). Each trial met its prespecified per-protocol A/H1N1 non-inferiority criterion (Table S12). The exploratory pooled A/H1N1 HI GMR was 0.950 (95% CI 0.683–1.322; I^2^ 65.9%; k = 3). This summary combined standard-dose, high-dose, and adjuvanted QIV products, and its confidence interval included 1 (Table S13).

A/H3N2, B/Victoria, and B/Yamagata HI results were presented per study without pooled estimates, because circulating strains and vaccine formulations differed across trials (Figure 4B–D; Table S12). Non-inferiority was demonstrated for these endpoints in the per-protocol analyses of the seasonal-QIV and high-dose-QIV trials. In the adjuvanted-QIV trial [20], the primary A/Darwin H3N2 HI GMR was 0.758 (95% CI 0.654–0.885), and non-inferiority was not demonstrated in the per-protocol analysis. The exposed-set H3N2 analysis also did not meet the numerical criterion (Table S8). Non-inferiority was demonstrated for the B/Victoria and B/Yamagata endpoints in the trial’s per-protocol analysis.

Non-influenza partner-vaccine immunogenicity was reported by single trials and was presented descriptively without pooling (Figures S1 and S2). For the COVID-19 mRNA (XBB.1.5) trial [16], the per-protocol GMR was 0.763 (95% CI 0.662–0.885), and non-inferiority was not demonstrated. The exposed-set sensitivity analysis met the numerical criterion (GMR 0.781, 95% CI 0.680–0.901; Table S8). For PCV20 [15], non-inferiority was demonstrated for all 20 serotypes in the per-protocol analysis (Tables S12 and S14). Serotype GMRs ranged from 0.676 to 0.847, and all lower 95% confidence limits exceeded the trial-specific boundary of 0.5, including serotype 1 (GMR 0.676, 95% CI 0.552–0.820). For RZV [17], the anti-gE GMR was 0.833 (95% CI 0.714–0.909), and non-inferiority was demonstrated in the HZ per-protocol population (Table S12). Arm-level partner-vaccine antibody responses are presented in Tables S10 and S15.

### 3.5. Safety

**Figure 5:**
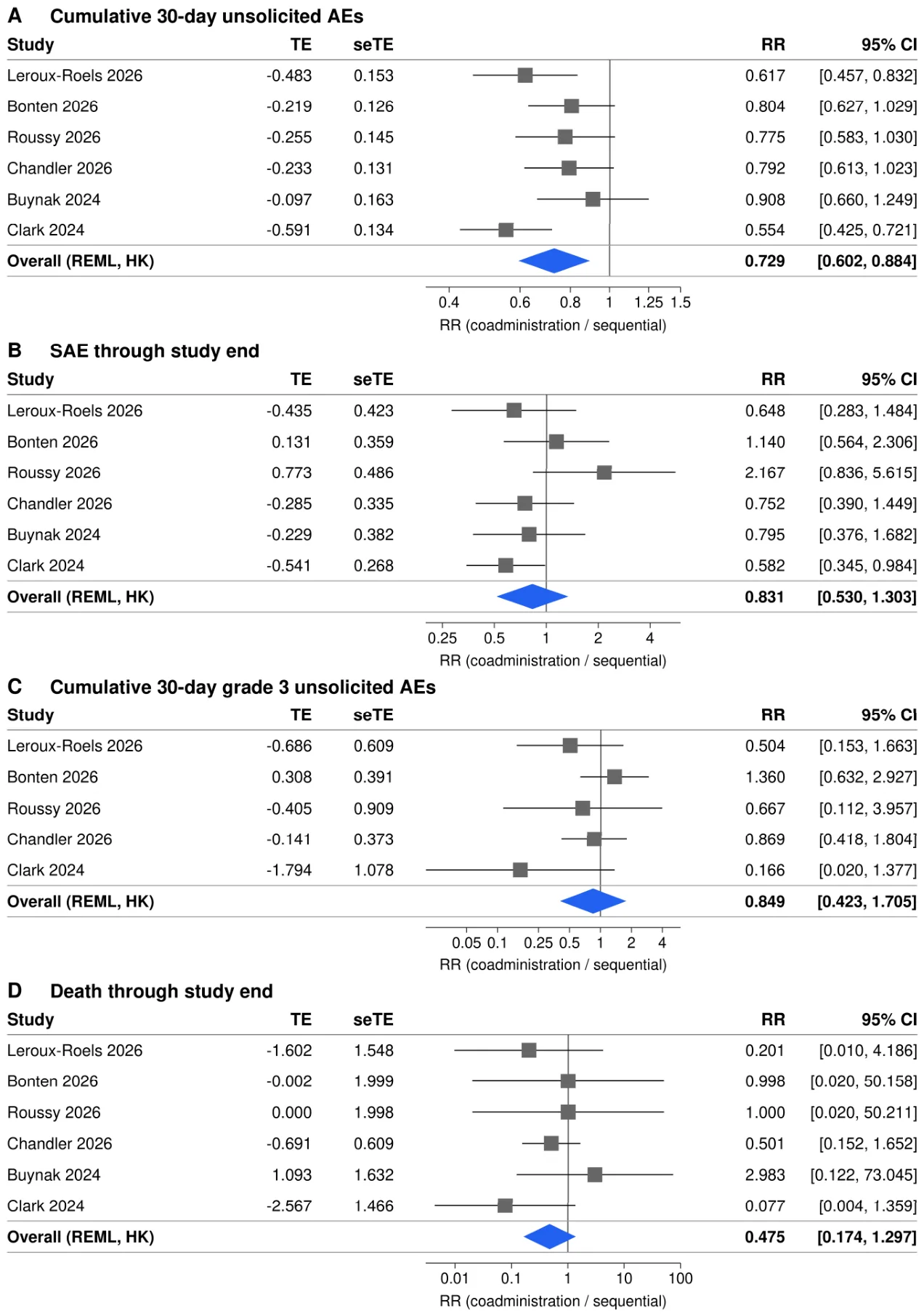
Safety outcomes: (**A**) cumulative 30-day unsolicited AEs; (**B**) SAEs through study end; (**C**) cumulative 30-day grade 3 unsolicited AEs; (**D**) deaths through study end. Diamonds indicate pooled RRs with 95% CIs. Reporting windows and continuity corrections are described in the Supplementary Methods. AE, adverse event; SAE, serious adverse event; TE, log(RR); seTE, standard error of TE [15,16,17,18,19,20].

The pooled RR for serious adverse events through study end was 0.831 (95% CI 0.530–1.303; I^2^ 20.2%; k = 6); the confidence interval included both lower and higher risk (Figure 5B). Deaths through study end were rare (5 versus 16 participants; RR 0.475, 95% CI 0.174–1.297) (Figure 5D).

The pooled RR for grade 3 unsolicited adverse events within 30 days was 0.849 (95% CI 0.423–1.705; k = 5; one trial [19] did not report this outcome) (Figure 5C). Solicited adverse events within 4 days were reported by two trials [18,19]. The pooled RRs were 1.109 (95% CI 0.440–2.795) for local reactions and 0.916 (95% CI 0.581–1.443) for systemic reactions (Figure S3; Table 2). Across six trials, the pooled RRs were 0.557 (95% CI 0.349–0.889) for study non-completion for any reason and 1.024 (95% CI 1.012–1.036) for study completion (Figure S4).

The pooled RR for any unsolicited adverse event within 30 days after any dose was 0.729 (95% CI 0.602–0.884; I^2^ 40.1%; k = 6), corresponding to 426 versus 588 participants with at least one event among 2716 exposed-set participants per strategy (Figure 5A; Table 2). Each participant was counted once across the assigned schedule. This was a cumulative comparison across one or two 30-day reporting windows for coadministration and two or three windows for sequential administration (Table S16).

### 3.6. Sensitivity Analyses

Sensitivity analyses of the exploratory joint RSV summary yielded GMRs of 0.907 (95% CI 0.840–0.980), 0.908 (95% CI 0.841–0.981), and 0.908 (95% CI 0.840–0.980) at working correlations of 0, 0.5, and 0.8, respectively (Figure S5). The study-level composite analysis yielded a GMR of 0.905 (95% CI 0.839–0.977; Figure S6).

Leave-one-study-out joint GMRs ranged from 0.893 to 0.933, with confidence intervals including one in three of the six analyses (Figure S7). Omitting the seasonal-QIV trial, which contributed the lowest subtype-specific GMRs, yielded a joint GMR of 0.933 (95% CI 0.897–0.970). Excluding the seasonal-QIV RSV-B subset estimate and the hierarchically non-confirmatory RSV-B estimate from the adjuvanted-QIV trial yielded a joint GMR of 0.922 (95% CI 0.853–0.998), based on 10 estimates from six trials. Full results across model specifications are presented in Tables S11 and S17. Death analyses were directionally consistent under alternative corrections (Figure S8; Table S18). Funnel plots and Egger’s tests were not used because only six or fewer studies contributed to each analysis.

### 3.7. Quality Assessment

Risk of bias was assessed with the Cochrane RoB 2 tool at the outcome level, separately for RSV-A, RSV-B, partner-vaccine immunogenicity, and safety outcomes. The randomization domain was rated low-risk in all six trials. For each RSV subtype, four trials were rated as having some concerns and two as high-risk because of per-protocol selection in the RZV trial and uncertainty about missing antibody outcomes in the seasonal-QIV trial (Figures S9 and S10). The cumulative unsolicited adverse-event, serious adverse-event, and death results were rated high-risk, principally because of incomplete follow-up. Solicited-reaction results had some concerns in the seasonal-QIV trial and were high-risk in the high-dose-QIV trial (Supplementary RoB 2).

GRADE certainty was low for RSV-A, the exploratory joint RSV summary, cumulative unsolicited adverse events, and serious adverse events, and very low for RSV-B and deaths (Table 3). Product-specific partner-vaccine antibody evidence ranged from moderate to low certainty (Table S19). Detailed judgments and downgrade reasons are reported in Tables S13 and S19 and the Supplementary RoB 2 assessments. All six trials were funded by GSK; sponsor roles are detailed in Table S6.

## 4. Discussion

This systematic review and meta-analysis evaluated the immunogenicity and safety of same-day coadministration of RSVPreF3 OA with indicated partner vaccines relative to sequential administration in adults aged 50 years or older. We found that the pooled RSV-A and RSV-B neutralizing-antibody point estimates were lower with coadministration and that partner-vaccine findings differed by product and antigen; serious adverse events and deaths were infrequent, with wide confidence intervals.

Individual trials have evaluated coadministration of other RSV vaccine platforms, including RSVpreF with influenza vaccine [13] and mRNA-1345 with influenza or COVID-19 vaccines [14]. A prior synthesis of respiratory-virus vaccine coadministration [21] pooled across vaccine platforms and serological endpoints. In the present synthesis, six trials evaluated the same RSV vaccine product and measured RSV-neutralizing antibody responses approximately 1 month after vaccination. The exploratory joint pooled estimate was about 9% lower with coadministration, with pooled point estimates below 1 for both RSV-A and RSV-B. These findings extend the evidence from individual vaccine pairs by describing the magnitude and variation in RSVPreF3 OA antibody responses across the evaluated partner vaccines.

The partner-vaccine findings differed by formulation and antigen. The PCV20 OPA and RZV anti-gE responses were lower with coadministration, although each trial met its prespecified per-protocol non-inferiority criteria [15,17]. For Comirnaty XBB.1.5, non-inferiority was not demonstrated for SARS-CoV-2 XBB.1.5 neutralizing antibodies in the primary per-protocol analysis [16]. The clinical implications of this reduced response to the evaluated formulation and variant remain uncertain. Influenza responses varied across formulations and individual strains, with non-inferiority not demonstrated for the A/H3N2 response in the adjuvanted-QIV trial [20]. Prior vaccination may also have influenced the influenza HI responses [12]. However, previous immunization was not unique to influenza: prior COVID-19 vaccination was an entry requirement in the Comirnaty trial [16].

Neutralizing antibodies is an important measure of the immune response to RSV vaccination, but no validated quantitative correlate of protection has been established for RSVPreF3 OA [30]. The responses after same-day coadministration were lower than those after sequential administration in the included trials, and the implications of this difference for protection against RSV disease remain uncertain. The interpretation of these antibody differences is of particular relevance to older adults, in whom vaccine responses can attenuate with advancing age [12]. Coadministration can spare a separate clinic visit for the RSV vaccine, a practical advantage for this age group. Adult immunization schedules have become increasingly crowded, and consolidating vaccines into a single appointment may help older adults complete the recommended vaccination plan [8,9]. For adults eligible for RSVPreF3 OA, coadministration with an evaluated partner vaccine may be considered when consistent with the approved product information and applicable immunization guidelines [10].

The safety assessments in this review examined serious adverse events, deaths, grade 3 unsolicited adverse events, and solicited local and systemic reactions. No clear difference between schedules emerged for these outcomes, although the estimates were imprecise. Unsolicited adverse events were reported less frequently in the coadministration arm than in the sequential arm across the full vaccination schedule. Sequential schedules included more post-vaccination reporting windows, providing more opportunities to record events. The lower cumulative reporting frequency should therefore be interpreted in relation to these differences in observation rather than as a reduction in adverse-event risk.

This review has several limitations. First, the number of included studies was small, heterogeneity was present, and publication bias could not be formally assessed. With only six trials, prediction intervals are sensitive to uncertainty in the estimated heterogeneity. All six trials were funded by GSK, with sponsor involvement in trial conduct, analysis, or reporting. Second, per-protocol selection and missing antibody measurements may have affected the pooled estimates, particularly in the RZV and seasonal-QIV trials. The certainty of evidence was low for RSV-A and very low for RSV-B. Open-label reporting, incomplete follow-up, and sparse events also limited the safety evidence. Third, partner-vaccine endpoints came from different formulations, strains, and single studies, limiting assessment of whether the effect of coadministration varied by partner vaccine. The sequential schedules consistently administered the partner vaccine first, approximately 1 month before RSVPreF3 OA. Fourth, baseline antibody measurements and eligibility criteria provided an incomplete account of participants’ prior vaccination and infection histories. The available aggregate data did not permit assessment of whether these histories or baseline immunity modified the antibody differences between coadministration and sequential administration, particularly for influenza HI responses. Future studies with standardized immunogenicity assays and adverse-event windows could refine the evidence base for scheduling. Real-world data on clinical RSV outcomes and vaccination completion could address questions that remain open for the populations represented in these trials.

## 5. Conclusions

This meta-analysis found that same-day RSVPreF3 OA coadministration was associated with lower RSV-neutralizing antibody responses, with partner-vaccine findings varying by product and antigen. Non-inferiority was not demonstrated for the SARS-CoV-2 XBB.1.5 and adjuvanted-QIV A/H3N2 antibody endpoints in the original per-protocol analyses. Coadministration may be considered for the evaluated vaccine combinations within approved product information and applicable immunization guidelines. Further evidence on clinical protection and safety would inform these scheduling decisions, given the low to very low certainty of the RSV antibody evidence.

## Figures and Tables

**Table 1:** Characteristics of the included studies.

Study; Registration Number	Countries	Age Eligibility, Years	Randomized Participants, *n*	Female, %	Partner Vaccine	Funding Source
Leroux-Roels, 2026 [15] NCT05879107	Belgium, Poland, Spain, USA	≥60	1112	56.8	PCV20	GSK
Bonten, 2026 [16] NCT06374394	USA, Belgium, Netherlands, Spain	≥50	841	52.7	COVID-19 mRNA, XBB.1.5	GSK
Roussy, 2026 [17] NCT05966090	Canada, USA	≥50	530	55.1	RZV	GSK
Chandler, 2026 [18] NCT04841577	New Zealand, Panama, South Africa	≥60	890	51.5	Seasonal QIV	GSK
Buynak, 2024 [19] NCT05559476	USA	≥65	1037	49.6	High-dose QIV	GSK
Clark, 2024 [20] NCT05568797	Belgium, Finland, France, Spain, UK	≥65	1045	49.3	Adjuvanted QIV	GSK

PCV20, 20-valent pneumococcal conjugate vaccine; QIV, quadrivalent influenza vaccine; RZV, recombinant zoster vaccine.

**Table 2:** Safety outcomes following coadministration versus sequential administration.

Outcome	No. of Studies	Coadministration, *n/N*	Sequential Administration, *n/N*	RR (95% CI)	I^2^ (%)	*p* for Heterogeneity
Unsolicited AEs within 30 days	6	426/2716	588/2716	0.729 (0.602–0.884)	40.1	0.136
SAEs through study end	6	86/2716	105/2716	0.831 (0.530–1.303)	20.2	0.231
Grade 3 unsolicited AEs within 30 days	5	35/2200	43/2203	0.849 (0.423–1.705)	3.2	0.337
Deaths through study end	6	5/2716	16/2716	0.475 (0.174–1.297)	0.0	0.638
Solicited local AEs within 4 days	2	498/861	446/855	1.109 (0.440–2.795)	63.3	0.099
Solicited systemic AEs within 4 days	2	405/861	436/855	0.916 (0.581–1.443)	0.0	0.457

*n*, participants with the outcome; *N*, participants analysed. Thirty-day outcomes reflect cumulative reporting across each vaccination schedule. AE, adverse event; SAE, serious adverse event; RR, risk ratio; CI, confidence interval.

**Table 3:** Summary of findings.

Outcome	No. of Studies	Participants, *n*	Effect Estimate (95% CI)	GRADE Certainty
RSV-A neutralizing antibodies	6	4586	GMR 0.895 (0.816–0.983)	Low ^a^
RSV-B neutralizing antibodies	6	4184	GMR 0.922 (0.837–1.016)	Very low ^b^
SAEs through study end	6	5432	RR 0.831 (0.530–1.303)	Low ^c^
Deaths through study end	6	5432	RR 0.475 (0.174–1.297)	Very low ^d^
Unsolicited AEs within 30 days	6	5432	RR 0.729 (0.602–0.884)	Low ^e^

The exploratory joint RSV analysis yielded a GMR of 0.908 (95% CI 0.841–0.981; low certainty ^a^), combining overlapping RSV-A (*n* = 4586) and RSV-B (*n* = 4184) populations from six trials. RSV participant counts follow the trial-reported analysis populations (Table S8). ^a^ Risk of bias and inconsistency. ^b^ Risk of bias, inconsistency and imprecision. ^c^ Risk of bias and imprecision. ^d^ Risk of bias and very serious imprecision. ^e^ Risk of bias and indirectness. Detailed assessments are provided in Table S13. AE, adverse event; SAE, serious adverse event; CI, confidence interval; GMR, geometric mean ratio; RR, risk ratio.

## Data Availability

The data analyzed in this study are derived from the six trial publications and their supplements, with study-completion counts from the corresponding trial registry records. The audited extraction datasets, search records, screening decisions, analysis code, and decision log are available at Figshare: https://doi.org/10.6084/m9.figshare.33441169 (accessed on 8 September 2026).

## Supplementary Materials

The following supporting information can be downloaded at: https://www.mdpi.com/article/10.3390/vaccines14090826/s1. Figure S1: Non-influenza partner-vaccine immunogenicity; Figure S2: PCV20 immunogenicity by serotype; Figure S3: Four-day solicited reactogenicity; Figure S4: Study non-completion and completion; Figure S5: Working-correlation sensitivity; Figure S6: Model-specification sensitivity; Figure S7: Leave-one-study-out sensitivity; Figure S8: Rare-event sensitivity for deaths; Figure S9: RoB 2 assessments for RSV-A and RSV-B; Figure S10: Distribution of RoB 2 judgments for RSV-A and RSV-B; Table S1: Reports assessed for eligibility (*n* = 215); Table S2: Excluded reports with specific eligibility reasons (*n* = 195); Table S3: Primary and companion reports of the six included randomized trials (20 reports); Table S4: Source materials and dispositions for 71 reconciled reports; Table S5: PCV20 participant flow and source definitions; Table S6: Detailed characteristics of the included trials; Table S7: Trial-level non-inferiority assessments of RSV-neutralizing antibody responses; Table S8: Immunogenicity estimates by analysis population; Table S9: RSV antibody geometric mean increases and between-schedule GMRs; Table S10: Reported absolute pre- and post-vaccination antibody titers and concentrations; Table S11: Heterogeneity and variance components across working correlations; Table S12: Non-inferiority assessments of partner-vaccine antibody responses; Table S13: GRADE Summary of Findings; Table S14: PCV20 serotype-specific OPA GMRs; Table S15: Partner-vaccine antibody geometric mean increases and between-schedule GMRs; Table S16: Trial-level construction of the 30-day unsolicited adverse-event outcome; Table S17: Joint model and sensitivity results; Table S18: Sensitivity analyses for deaths; Table S19: Product-specific partner-vaccine GRADE evidence profile; PRISMA 2020 checklist; PRISMA-S appendix; Risk-of-bias 2 (RoB 2) result-level assessments and full signalling-question responses. Supplementary Methods: Protocol and analysis amendments; effect-size orientation and standard-error reconstruction; Supplementary Data and Code: extraction datasets, search records, screening decisions, report-to-trial correspondence, and analysis scripts. References [15,16,17,18,19,20] are cited in the Supplementary Materials.

## Abbreviations

AE, adverse event; CI, confidence interval; CR2, bias-reduced cluster-robust variance estimator; GLS, generalized least squares; GMR, geometric mean ratio; GRADE, Grading of Recommendations, Assessment, Development and Evaluations; HI, hemagglutination inhibition; HK, Hartung–Knapp; I^2^, I-squared heterogeneity statistic; NI, non-inferiority; OPA, opsonophagocytic assay; PCV20, 20-valent pneumococcal conjugate vaccine; PPS, per-protocol set; PRISMA, Preferred Reporting Items for Systematic Reviews and Meta-Analyses; PROSPERO, International Prospective Register of Systematic Reviews; QIV, quadrivalent influenza vaccine; REML, restricted maximum likelihood; RoB 2, Cochrane Risk-of-Bias tool for randomized trials, version 2; RR, risk ratio; RSV, respiratory syncytial virus; RSVPreF3 OA, AS01E-adjuvanted respiratory syncytial virus prefusion F protein vaccine; RZV, recombinant zoster vaccine; SAE, serious adverse event.
