# Supplementary material for: Immunogenicity and Safety of RSVPreF3 OA Coadministration Versus Sequential Administration in Adults: A Systematic Review and Meta-Analysis
Source: Vaccines (Basel). 2026 Sep 20;14(9):826. doi: 10.3390/vaccines14090826 (PMC13611371; doi:10.3390/vaccines14090826)
Supplement: Supplementary file 1 [file vaccines-14-00826-s001.zip › PRISMA-S appendix.pdf]

PRISMA-S: PRISMA Search Reporting Extension - Appendix

Same-day coadministration of RSVPreF3 OA versus partner-first sequential administration in adults. Searches completed on 3 August 2026.

This appendix reports the search of the systematic review per the PRISMA-S (Search Reporting Extension) checklist.

Screening was performed by two reviewers independently, with disagreements resolved by discussion or a third reviewer. The source-to-final ledger and Table S4 distinguish report formats, bibliographic corrections and trial associations.

PRISMA-S item	Reporting focus	Review methods and location
1. Database name	Databases and platforms	PubMed (web and NCBI E-utilities); Embase (Elsevier Embase.com); CENTRAL (Cochrane Library); Web of Science Core Collection (Clarivate); Scopus (Elsevier). Full strategies follow below.
2. Multi-database searching	Simultaneous database searches	The five database searches were executed separately. Web of Science Core Collection was searched across its selected component indexes.
3. Study registries	Registry sources	ClinicalTrials.gov and European registry reports identified through database records were used for protocol verification and trial characteristics. Registry identifiers linked these reports to the included trials. ClinicalTrials.gov participant-flow results also supplied group-specific study-completion counts, cross-checked against the trial publications.
4. Online resources and browsing	Other browsed sources	Publisher pages, conference volumes and registry records were consulted to resolve report identity and obtain source material. Their URLs and report locators are retained in the report-eligibility ledger and Table S4.
5. Citation searching	Reference-list checking	Reference lists of included primary articles and relevant reviews were checked. The six primary articles are identified in Table S3.
6. Contacts	Author or expert contact	No additional study or unpublished numerical result was obtained through author, expert or manufacturer contact.
7. Other methods	Additional identification methods	No additional identification stream was added to the five-database record total. Companion reports were linked to studies already represented in those records.

PRISMA-S item	Reporting focus	Review methods and location
8. Full search strategies	Verbatim strategies	Full database-specific strategies appear below. Search records and execution logs are included in Supplementary Data and Code, 02_search_records.
9. Limits and restrictions	Applied search limits	No language, date, age or publication-status limit was applied to the primary searches. Eligibility was not restricted by publication language. Study-design and animal-only filters are described under item 10.
10. Search filters	Study-design filters	PubMed used an RCT filter and animal-only exclusion; Embase used RCT/human logic. CENTRAL used the Trials collection. Web of Science and Scopus used the terms shown in their full strategies.
11. Prior work	Strategy development	Strategies were developed for this review using controlled vocabulary and free-text RSV vaccination and coadministration concepts, with database-specific trial filters where applicable.
12. Updates	Search updates	The reported identification dataset comes from the final database runs on 2–3 August 2026. Earlier strategy-development runs were not added to these totals.
13. Dates of searches	Last database searches	PubMed and Embase: 2 August 2026; Embase export completed 3 August. CENTRAL, Web of Science and Scopus: 3 August 2026. Coverage was from database inception.
14. Peer review	Search-strategy checking	Search strategies were checked against PRESS and PRISMA-S criteria during development. The supporting tools are described in the Acknowledgments, and the search documentation accompanies this appendix.
15. Total records	Records before deduplication	PubMed 79; Embase 1,036; CENTRAL 66; Web of Science 141; Scopus 159. Total: 1,481 database records.
16. Deduplication	Record deduplication	DOI, title, authors, bibliographic details and registry identifiers were used to resolve duplicate records. Different abstracts in a shared-DOI conference volume were identified by their individual titles, abstract numbers and page locators and retained as separate reports. Removal of 286 duplicates left 1,195 records; the source-to-final mapping is in Supplementary Data and Code.

Search counts (Figure 1) - final

Step	n
Records identified from databases	1,481
Duplicate records removed	286
Records screened	1,195
Records excluded at title and abstract screening	980
Reports assessed for eligibility	215
Reports excluded	195
Reports of included studies	20
Independent randomized trials included	6

Report exclusion reasons: secondary report or report format not independently eligible, 44; nonclinical, laboratory or analytical methods study, 21; observational, nonrandomized or modelling study, 32; intervention or population outside scope, 84; no eligible coadministration-versus-sequential comparison, 14. Table S2 provides the specific reason for each report.

Full Search Strategies by Database

The verbatim strategies used for each database are provided below. Databases were searched from inception to 3 August 2026, with no date, language, age, or publication-status limits applied to the primary sets. Limits and filters are shown within each strategy where applicable. Eligibility was not restricted by publication language.

PubMed

```
#1
(
 "Respiratory Syncytial Virus Vaccines"[Mesh]
 OR
 (
  (
   "Respiratory Syncytial Virus, Human"[Mesh]
   OR "Respiratory Syncytial Viruses"[Mesh]
   OR RSV[tiab]
   OR HRSV[tiab]
   OR "respiratory syncytial virus"[tiab]
   OR "respiratory syncytial viral"[tiab]
   OR "respiratory syncytial"[tiab]
  )
  AND
  (
   vaccin*[tiab]
   OR immuniz*[tiab]
   OR immunis*[tiab]
  )
 )
 OR RSVPreF3[tiab]
 OR RSVpreF[tiab]
 OR "mRNA-1345"[tiab]
 OR "mRNA 1345"[tiab]
 OR Arexvy[tiab]
 OR Abrysvo[tiab]
 OR mResvia[tiab]
```

OR "Ad26.RSV.preF"[tiab]
OR "Ad26 RSV preF"[tiab]
OR MEDI7510[tiab]
)

#2
(
"Immunization Schedule"[Mesh]
OR "Drug Administration Schedule"[Mesh]
OR coadminist*[tiab]
OR co-administ*[tiab]
OR concomitant*[tiab]
OR concurrent*[tiab]
OR simultaneous*[tiab]
OR "same day"[tiab]
OR "same-day"[tiab]
OR "same visit"[tiab]
OR "same-visit"[tiab]
OR admix*[tiab]
OR co-inject*[tiab]
OR coinject*[tiab]
OR "given together"[tiab:~2]
OR "administered together"[tiab:~2]
)

#3
(
randomized controlled trial[pt]
OR controlled clinical trial[pt]
OR randomized[tiab]
OR placebo[tiab]
OR drug therapy[sh]
OR randomly[tiab]
OR trial[tiab]
OR groups[tiab]
)
NOT
(
animals[mh] NOT humans[mh]
)

#4
#1 AND #2 AND #3

Embase (Elsevier)

#1
'respiratory syncytial virus vaccine'/exp
OR
('respiratory syncytial virus'/exp AND 'vaccine'/exp)

#2
(
(rsv OR hrsv OR 'respiratory syncytial virus' OR 'respiratory syncytial viral')
NEAR/3
(vaccin* OR immuniz* OR immunis*)
):ti,ab,kw

#3
(

```
  rsvpref3
  OR rsvpref
  OR 'mrna-1345'
  OR 'mrna 1345'
  OR arexvy
  OR abrysvo
  OR mresvia
  OR 'ad26.rsv.pref'
  OR 'ad26 rsv pref'
  OR medi7510
):ti,ab,kw

#4
#1 OR #2 OR #3

#5
'immunization schedule'/exp
OR 'drug administration schedule'/exp

#6
(
  coadminist*
  OR (co NEXT/1 administ*)
  OR concomitant*
  OR concurrent*
  OR simultaneous*
  OR (same NEXT/1 day)
  OR (same NEXT/1 visit)
  OR admix*
  OR coinject*
  OR (co NEXT/1 inject*)
  OR (given NEAR/3 together)
  OR (administer* NEAR/3 together)
):ti,ab,kw

#7
#5 OR #6

#8
'randomized controlled trial'/exp

#9
'controlled clinical trial'/de

#10
random*:ti,ab,tt

#11
'randomization'/de

#12
'intermethod comparison'/de

#13
placebo:ti,ab,tt

#14
(compare:ti,tt OR compared:ti,tt OR comparison:ti,tt)
```

#15
(
  (evaluated:ab OR evaluate:ab OR evaluating:ab OR assessed:ab OR assess:ab)
  AND
  (compare:ab OR compared:ab OR comparing:ab OR comparison:ab)
)

#16
(open NEXT/1 label):ti,ab,tt

#17
((double OR single OR doubly OR singly) NEXT/1 (blind OR blinded OR blindly)):ti,ab,tt

#18
'double blind procedure'/de

#19
(parallel NEXT/1 group*):ti,ab,tt

#20
(crossover:ti,ab,tt OR 'cross over':ti,ab,tt)

#21
(
  (assign* OR match OR matched OR allocation)
  NEAR/6
  (alternate OR group OR groups OR intervention OR interventions OR patient OR patients OR subject OR subjects OR participant OR participants)
):ti,ab,tt

#22
(assigned:ti,ab,tt OR allocated:ti,ab,tt)

#23
(controlled NEAR/8 (study OR design OR trial)):ti,ab,tt

#24
(volunteer:ti,ab,tt OR volunteers:ti,ab,tt)

#25
'human experiment'/de

#26
trial:ti,tt

#27
#8 OR #9 OR #10 OR #11 OR #12 OR #13 OR #14 OR #15 OR #16 OR #17 OR #18 OR #19 OR #20 OR #21 OR #22 OR #23 OR #24 OR #25 OR #26

#28
(
  (
    (random* NEXT/1 sampl* NEAR/8 ('cross section*' OR questionnaire* OR survey OR surveys OR database OR databases)):ti,ab,tt
)
  NOT
  (

    'comparative study'/de
    OR 'controlled study'/de
    OR 'randomised controlled':ti,ab,tt
    OR 'randomized controlled':ti,ab,tt
    OR 'randomly assigned':ti,ab,tt
)
)

#29
(
  'cross-sectional study'/de
  NOT
  (
    'randomized controlled trial'/exp
    OR 'controlled clinical study'/de
    OR 'controlled study'/de
    OR 'randomised controlled':ti,ab,tt
    OR 'randomized controlled':ti,ab,tt
    OR 'control group':ti,ab,tt
    OR 'control groups':ti,ab,tt
)
)

#30
(
  'case control*':ti,ab,tt
  AND random*:ti,ab,tt
  NOT ('randomised controlled':ti,ab,tt OR 'randomized controlled':ti,ab,tt)
)

#31
('systematic review':ti,tt NOT (trial:ti,tt OR study:ti,tt))

#32
(nonrandom*:ti,ab,tt NOT random*:ti,ab,tt)

#33
'random field*':ti,ab,tt

#34
('random cluster' NEAR/4 sampl*):ti,ab,tt

#35
((review:ab AND review:it) NOT trial:ti,tt)

#36
('we searched':ab AND (review:ti,tt OR review:it))

#37
'update review':ab

#38
(databases NEAR/5 searched):ab

#39
(
  (
    rat:ti,tt OR rats:ti,tt OR mouse:ti,tt OR mice:ti,tt

OR swine:ti,tt OR porcine:ti,tt OR murine:ti,tt
OR sheep:ti,tt OR lambs:ti,tt OR pigs:ti,tt OR piglets:ti,tt
OR rabbit:ti,tt OR rabbits:ti,tt OR cat:ti,tt OR cats:ti,tt
OR dog:ti,tt OR dogs:ti,tt OR cattle:ti,tt OR bovine:ti,tt
OR monkey:ti,tt OR monkeys:ti,tt OR trout:ti,tt OR marmoset*:ti,tt
)
AND 'animal experiment'/de
)

#40
('animal experiment'/de NOT ('human experiment'/de OR 'human'/de))

#41
#28 OR #29 OR #30 OR #31 OR #32 OR #33 OR #34 OR #35 OR #36 OR #37 OR #38 OR #39 OR #40

#42
#27 NOT #41

#43
#4 AND #7 AND #42

Cochrane Central Register of Controlled Trials (CENTRAL)

#1
MeSH descriptor: [Respiratory Syncytial Virus Vaccines] explode all trees

#2
MeSH descriptor: [Respiratory Syncytial Virus, Human] explode all trees

#3
MeSH descriptor: [Vaccines] explode all trees

#4
#2 AND #3

#5
(
(RSV OR HRSV OR "respiratory syncytial virus" OR "respiratory syncytial viral")
NEAR/3
(vaccin* OR immuniz* OR immunis*)
):ti,ab,kw

#6
(
RSVPreF3 OR RSVpreF OR "mRNA-1345" OR "mRNA 1345"
OR Arexvy OR Abrysvo OR mResvia OR "Ad26.RSV.preF"
OR "Ad26 RSV preF" OR MEDI7510
):ti,ab,kw

#7
#1 OR #4 OR #5 OR #6

#8
MeSH descriptor: [Immunization Schedule] explode all trees

#9
MeSH descriptor: [Drug Administration Schedule] explode all trees

#10
(
  coadminist* OR co-administ* OR concomitant* OR concurrent* OR simultaneous*
  OR "same day" OR "same-day" OR "same visit" OR "same-visit"
  OR admix* OR co-inject* OR coinject*
  OR (given NEAR/2 together)
  OR (administer* NEAR/2 together)
):ti,ab,kw

#11
#8 OR #9 OR #10

#12
#7 AND #11

Web of Science Core Collection

#1
TS=(
  (
    (RSV OR HRSV OR "respiratory syncytial virus" OR "respiratory syncytial viral")
    NEAR/3
    (vaccin* OR immuniz* OR immunis*)
)
  OR RSVPreF3 OR RSVpreF OR "mRNA-1345" OR "mRNA 1345"
  OR Arexvy OR Abrysvo OR mResvia
  OR "Ad26.RSV.preF" OR "Ad26 RSV preF" OR MEDI7510
)

#2
TS=(
  coadminist* OR co-administ* OR concomitant* OR concurrent* OR simultaneous*
  OR "same day" OR "same-day" OR "same visit" OR "same-visit"
  OR admix* OR co-inject* OR coinject*
  OR (given NEAR/2 together)
  OR (administer* NEAR/2 together)
)

#3
TS=(
  random* OR placebo* OR trial*
  OR (controlled NEAR/3 trial*)
  OR (controlled NEAR/3 stud*)
  OR (parallel NEAR/1 group*)
  OR (single NEAR/1 blind*)
  OR (double NEAR/1 blind*)
  OR (observer NEAR/1 blind*)
  OR assign* OR allocat*
)

#4
#1 AND #2 AND #3

Scopus

TITLE-ABS-KEY(
  (
    (RSV OR HRSV OR "respiratory syncytial virus" OR "respiratory syncytial viral")
    W/3
    (vaccin* OR immuniz* OR immunis*)

)
OR RSVPreF3 OR RSVpreF OR "mRNA-1345" OR "mRNA 1345"
OR Arexvy OR Abrysvo OR mResvia
OR "Ad26.RSV.preF" OR "Ad26 RSV preF" OR MEDI7510
)
AND
TITLE-ABS-KEY(
coadminist* OR co-administ* OR concomitant* OR concurrent* OR simultaneous*
OR "same day" OR "same-day" OR "same visit" OR "same-visit"
OR admix* OR co-inject* OR coinject*
OR (given W/2 together)
OR (administer* W/2 together)
)
AND
TITLE-ABS-KEY(
random* OR placebo* OR trial*
OR (controlled W/3 trial*)
OR (controlled W/3 stud*)
OR (parallel W/1 group*)
OR (single W/1 blind*)
OR (double W/1 blind*)
OR (observer W/1 blind*)
OR assign* OR allocat*
)
