# Supplementary material for: Immunogenicity and Safety of RSVPreF3 OA Coadministration Versus Sequential Administration in Adults: A Systematic Review and Meta-Analysis
Source: Vaccines (Basel). 2026 Sep 20;14(9):826. doi: 10.3390/vaccines14090826 (PMC13611371; doi:10.3390/vaccines14090826)
Supplement: Supplementary file 1 [file vaccines-14-00826-s001.zip › Supplementary Materials.pdf]

Supplementary Materials

Immunogenicity and Safety of RSVPreF3 OA Coadministration versus Sequential Administration in Adults: A Systematic Review and Meta-Analysis

Supplementary Methods. Effect-size orientation and standard-error reconstruction

Selection of immunogenicity estimates. RSV synthesis used subtype-specific ED60 estimates at approximately one month after the RSV dose from the trial-defined per-protocol analyses. For the PCV20 trial, the pooled estimates were the adjusted per-protocol comparisons presented in the primary article's main figure; the additional per-protocol model and exposed-set estimates were retained separately in Table S8. Reported estimates from broader analysis populations were also retained in Table S8 for comparison. Adjusted between-schedule GMRs were extracted directly rather than calculated by dividing unadjusted arm-level geometric means. The arm-level geometric means and geometric mean increases are reported separately in Tables S17, S10 and S15.

Immunogenicity ratios. Adjusted ratios and their reported 95% confidence limits were extracted from the primary trial articles and their supplements. When a ratio was reported as sequential administration divided by coadministration, the point estimate was inverted and the confidence limits were exchanged and inverted: the new lower limit was the reciprocal of the original upper limit, and the new upper limit was the reciprocal of the original lower limit. Ratios already reported as coadministration divided by sequential administration retained their original direction.

Standard errors for log ratios. The natural logarithm of the oriented ratio was used as the effect estimate. Its standard error was calculated as the difference between the natural logarithms of the upper and lower 95% confidence limits, divided by twice the 97.5th percentile of the standard normal distribution (approximately 3.919928). The sampling variance was the square of this standard error. Reciprocal conversion leaves the log-scale standard error unchanged.

Worked example. For the RSV-A endpoint in the PCV20 trial (Leroux-Roels et al.; main-text reference [15], Figure 4), the reported control/coadministration ratio was 1.06 (95% CI 0.94–1.20). Conversion gave a coadministration/sequential ratio of 0.9434 (95% CI 0.8333–1.0638), a log ratio of −0.05827, a standard error of 0.06230 and a sampling variance of 0.00388083.

Binary outcomes. Each arm contributed the number of participants with the outcome and the number analyzed. For the high-dose-QIV 4-day solicited AE outcomes, diary-evaluable denominators were reconstructed from the published participant counts and percentages: 423 for coadministration and 417 for sequential administration [19]. These were the unique integer denominators consistent with the reported percentages rounded to one decimal place. The risk ratio was the coadministration-arm risk divided by the sequential-arm risk; its natural logarithm was the effect estimate. The sampling variance of the log risk ratio was calculated as the reciprocal of the coadministration event count minus the reciprocal of its arm total, plus the reciprocal of the sequential event count minus the reciprocal of its arm total. The standard error was the square root of this variance.

Zero-cell corrections. For the risk-ratio analyses, if any event or non-event cell in the two-by-two table was zero, 0.5 was added to all four cells before calculating the effect estimate and variance. Corrected arm totals were the sums of the corrected event and non-event cells. Otherwise, the original counts were used. The rare-event sensitivity analysis repeated the risk-ratio calculation using 0.1 in place of 0.5. Supplementary Data and Code contains the original counts, oriented estimates, reconstructed standard errors, and analysis scripts.

Supplementary Methods. Safety outcome construction and non-inferiority margins

Unsolicited adverse events. The 30-day unsolicited AE outcome used the trial-reported number of participants with at least one event across each schedule's planned reporting windows, with the exposed set as the denominator and each participant counted once within the assigned schedule. Coadministration contributed one window (Days 1–30) and sequential

administration two windows (Days 1–30 and 31–60) in the five two-vaccine trials; in the RZV trial, the windows were Days 1–30 and 61–90 for coadministration and Days 1–30, 31–60 and 61–90 for sequential administration. The windows were non-overlapping, but the cumulative observation period differed between schedules. Grade 3 unsolicited adverse events used the same schedule-specific windows. Table S16 lists the counts, denominators, planned windows and source locations by trial.

Study non-completion and completion. Group-specific counts through the end of each assigned study schedule were extracted from ClinicalTrials.gov participant-flow results and cross-checked against the trial publications. The exposed population was used as the denominator. Non-completion comprised all reported reasons for not completing the study, including loss to follow-up, withdrawal of consent, adverse events and death. Completed and non-completed counts summed to the exposed population in each arm. Both outcomes were pooled across six trials using random-effects REML models with Hartung–Knapp intervals. Source records, counts and analyses are provided in Supplementary Data and Code, folder 22_attrition_completion.

Non-inferiority margins. The 1.5-fold RSV margin corresponds to a boundary of 1/1.5 (approximately 0.667) for the coadministration-to-sequential GMR. The PCV20 trial attributed its RSV margin to feedback from the US Food and Drug Administration's Center for Biologics Evaluation and Research [15]; the seasonal-QIV protocol amendment of 24 November 2020 changed its co-primary margins from 2.0 to 1.5 following a CBER recommendation [18] (NCT04841577 protocol, Amendment 1, summary of changes). An explicit rationale for the 1.5-fold margin was not identified in the other four protocols. Trial-specific margins, analysis populations, testing hierarchies and reported conclusions are listed in Table S7.

Supplementary Methods. Risk of bias and certainty of evidence

Risk of bias. RoB 2 judgments addressed the effect of assignment to the vaccination schedule for each assessed result, distinguishing protocol-based exclusions from unavailable outcome measurements. Assessments were outcome-specific for RSV-A, RSV-B, partner-vaccine antibody responses, unsolicited adverse events, grade 3 unsolicited adverse events, serious adverse events, deaths and diary reactogenicity. Each result was compared against the original trial report, analysis population, protocol and statistical analysis plan, including the timing of amendments relative to access to unblinded data. Detailed result-level judgments, signalling-question responses and source-based rationales are provided in Supplementary Data and Code.

Certainty of evidence. Evidence was rated across the five GRADE domains. Summary findings are presented in Table 3, with detailed domain judgments in Table S13. Product-specific partner-vaccine antibody endpoints were graded separately (Table S19). Formal tests for funnel-plot asymmetry were omitted when fewer than ten studies contributed to a pooled analysis, and the publication-bias domain was rated qualitatively. Funding and sponsor roles were extracted from the trial reports and considered alongside registration and outcome reporting in the qualitative publication-bias assessment. Certainty ratings refer to the measured outcomes in the evaluated populations and schedules. For antibody outcomes, imprecision was assessed for the direction of the laboratory response relative to GMR = 1, considering the confidence interval and endpoint-specific information.

Supplementary Methods. RSV models and sensitivity analyses

Each subtype was fitted separately using rma.uni with method = REML and test = knha, with one estimate per study and 5 residual degrees of freedom. These subtype models use a single between-study variance component. The joint model uses rma.mv with study- and endpoint-level random intercepts. Its sampling covariance has diagonal entries v_i and within-study off-diagonal entries $\rho \times \mathrm{sqrt}(v_i \times v_j)$; between-study sampling covariances are zero. CR2 variance estimation clusters on study, with Satterthwaite inference for the pooled log GMR.

For the joint model, $Q = (y - Xb_0)^T V^{-1}(y - Xb_0)$, where b_0 is the generalized least-squares intercept calculated from the sampling covariance V. The reference degrees of freedom are $12 - 1 = 11$. This sampling-covariance calculation differs from fitting the REML random effects. The descriptive I^2 proxy is $100 \times (\sigma^2\_study + \sigma^2\_endpoint)/(\sigma^2\_study + \sigma^2\_endpoint + mean(v\_i))$. Subtype I^2 uses the conventional metafor calculation with the typical sampling variance.

Approximate joint prediction limits were calculated on the log scale as b ± 1.96 × sqrt(SE_model² + σ²_study + σ²_endpoint), then exponentiated. They describe a subtype-specific true effect in a new study under the joint model, with both random components contributing. These are model-based limits, separate from the CR2 confidence interval for the mean. Approximate subtype prediction limits used b ± t_0.975,5 × sqrt(SE_HK² + τ²). With six studies, these plug-in intervals do not capture the full uncertainty in estimated heterogeneity.

Sensitivity analyses of the joint summary examined working correlations of ρ = 0 and 0.8 and fixed-effects generalized least squares, in addition to study-level composites. Study-level composites combine the two log GMRs using generalized least-squares weights and the same assumed sampling covariance, yielding one estimate and variance per trial. The six composites were pooled using the Paule–Mandel estimator with Hartung–Knapp intervals. Leave-one-study-out analyses refitted the joint model with CR2 inference after each omission. The exclusion analysis removed only the seasonal-QIV RSV-B subset and adjuvanted-QIV hierarchically non-confirmatory RSV-B rows, retaining six trials and 10 estimates. Tables S11 and S17 report the complete results.

Protocol and analysis amendments

During peer review, eligibility was broadened to remove the English-language restriction and address potential language bias. Non-English records were assessed against the clinical eligibility criteria. Report identity and trial linkage are documented in Tables S1, S2, S4 and S15.

Separate RSV-A and RSV-B REML analyses with Hartung–Knapp intervals were adopted during peer review to present subtype-specific responses and distinguish endpoints with different confirmatory status; the correlated joint model with CR2/Satterthwaite inference is exploratory. The interpretation of unsolicited adverse events was clarified as cumulative participant-level reporting across the assigned schedules' reporting windows. The continuity-correction label was corrected to describe the implemented addition of 0.5 to all four cells of studies with a zero cell, without changing the estimates. Antibody certainty assessments address the direction of the laboratory response relative to GMR = 1, using confidence intervals and endpoint-specific information. Supporting assessment and analysis records are provided in Supplementary Data and Code. Study-completion data were supplemented with registry participant-flow results during revision, extending both study non-completion and completion analyses to all six trials.

Supplementary Results. Figure and table notes

Table S6. Dates retain the period type reported in the source (study conduct or enrollment); study-conduct periods include follow-up, and day numbers follow the original trial schedules. Seasonal-QIV post-RSV PPS counts refer to Day 31 in Co-Ad and Day 61 in Seq; the Day-31 visit-2 cohort of 427/410 is used for influenza. Laboratory blinding in the seasonal- and high-dose-QIV trials is specified in protocol Section 6.3.5; clinical safety assessments used participant reports and investigator review. PCV20 population counts and source comparisons are provided in Table S5.

Figure 5 and Table 2. Coadministration arms contributed one 30-day reporting window in five trials and two windows in the RZV trial, whereas sequential arms contributed two or three windows; the cumulative 30-day unsolicited AE and grade 3 unsolicited AE comparisons therefore describe cumulative reporting across the assigned schedules. Serious adverse events were assessed through schedule-specific study end (approximately 6 versus 7 months from first vaccination in five trials and approximately 8 months in both arms in the RZV trial). Deaths were rare: five of six trials contained zero-event cells and were analysed with a fixed continuity correction of 0.5 added to all four cells; all six trials entered the death model, including two double-zero trials. The 4-day solicited AE analyses used participants with diary data in the seasonal-QIV trial and diary-evaluable participants in the modified safety set of the high-dose-QIV trial. Approximate prediction intervals for the pooled unsolicited AE, SAE and death outcomes are reported in Table S13; heterogeneity statistics are in Table 2. In Figure 5, TE denotes the log risk ratio and seTE its standard error.

Risk-of-bias 2 (RoB 2) domain-level judgments.

Study	Outcome / set	D1	D2	D3	D4	D5	Overall	Judgment basis	Evidence locators

Study	Outcome / set	D1	D2	D3	D4	D5	Overall	Judgment basis	Evidence locators
PCV20 NCT05879107	EC001 RSV-A neutralizing-antibody GMR Trial-defined RSV PPS; PPS 513/383	Low	Some concerns	Some concerns	Low	Low	Some concerns	D2: PPS 513/383; Figure 3: 16/108 visit-schedule exclusions, 10/24 laboratory-assessment exclusions and 8/9 withdrawals. D3: The 535/511 RSV-A and 534/512 RSV-B exposed-set analyses distinguish measured results from PPS eligibility. The smaller laboratory/withdrawal losses and documented sample-handling causes make response-dependent assay loss unlikely, although possible. D5: The pre-enrollment SAP and documented amendment history support the planned endpoint and analysis.	ciag250 primary report Methods/Statistical analyses, participant flow (Figure 3), antibody figures and safety results; original supplementary methods/population analyses; detailed questions: EC001; folder 21, EC001
PCV20 NCT05879107	EC002 RSV-B neutralizing-antibody GMR Trial-defined RSV PPS; PPS 513/383	Low	Some concerns	Some concerns	Low	Low	Some concerns	D2: PPS 513/383; Figure 3: 16/108 visit-schedule exclusions, 10/24 laboratory-assessment exclusions and 8/9 withdrawals. D3: The 535/511 RSV-A and 534/512 RSV-B exposed-set analyses distinguish measured results from PPS eligibility. The smaller laboratory/withdrawal losses and documented sample-handling causes make response-dependent assay loss unlikely, although possible. D5: The pre-enrollment SAP and documented amendment history support the planned endpoint and analysis.	ciag250 primary report Methods/Statistical analyses, participant flow (Figure 3), antibody figures and safety results; original supplementary methods/population analyses; detailed questions: EC002; folder 21, EC002
COVID-19 XBB.1.5 NCT06374394	EC003 RSV-A neutralizing-antibody GMR Trial-defined RSV PPS; RSV PPS 382/364	Low	Some concerns	Low	Low	Some concerns	Some concerns	D2: RSV PPS 382/364; Figure 3: 9/29 out-of-window assessments, 7/1 missing predose results, 2/2 missed immunogenicity assessments, 4/13 withdrawals; reasons overlap. D3: The exposed-set RSV model includes 404/400 participants, 804/841 randomized (95.6%) and 804/833 exposed (96.5%). For this continuous outcome, coverage is judged nearly complete; the 4.1% difference between PPS and broader RSV-A GMRs is considered in D2. D5: Timing of the relevant analysis amendment relative to unblinded outcome access is uncertain.	ciag344 primary report Methods/Statistical analyses, participant flow (Figure 3), antibody figures and safety results; original supplementary methods/population analyses; detailed questions: EC003; folder 21, EC003
COVID-19 XBB.1.5 NCT06374394	EC004 RSV-B neutralizing-antibody GMR Trial-defined RSV PPS; RSV PPS 382/364	Low	Some concerns	Low	Low	Some concerns	Some concerns	D2: RSV PPS 382/364; Figure 3: 9/29 out-of-window assessments, 7/1 missing predose results, 2/2 missed immunogenicity assessments, 4/13 withdrawals; reasons overlap. D3: The exposed-set RSV model includes 404/400 participants, 804/841 randomized (95.6%) and 804/833 exposed (96.5%). For this continuous outcome, coverage is judged nearly complete; the 4.1% difference between PPS and broader RSV-A GMRs is considered in D2. D5: Timing of the relevant analysis amendment relative to unblinded outcome access is uncertain.	ciag344 primary report Methods/Statistical analyses, participant flow (Figure 3), antibody figures and safety results; original supplementary methods/population analyses; detailed questions: EC004; folder 21, EC004
RZV NCT05966090	EC005 RSV-A neutralizing-antibody GMR Trial-defined RSV PPS; RSV PPS 240/219	Low	High	Some concerns	Low	Low	High	D2: RSV PPS 240/219; Figure 3: 12/36 visit-schedule exclusions, 2/1 laboratory exclusions and 2/3 withdrawals; 3/1 discontinued vaccine. D3: The reported RSV GMT sample counts are 247/219 after vaccination, compared with 261/249 before vaccination; these are available samples, not adjusted-model denominators. Laboratory-exclusion counts, visit-specific sample counts and PPS counts describe different quantities. D5: The pre-enrollment SAP and documented amendment history support the planned endpoint and analysis.	ciag208 primary report Methods/Statistical analyses, participant flow (Figure 3), antibody figures and safety results; original supplementary methods/population analyses; detailed questions: EC005; folder 21, EC005
RZV NCT05966090	EC006 RSV-B neutralizing-antibody GMR Trial-defined RSV PPS; RSV PPS 240/219	Low	High	Some concerns	Low	Low	High	D2: RSV PPS 240/219; Figure 3: 12/36 visit-schedule exclusions, 2/1 laboratory exclusions and 2/3 withdrawals; 3/1 discontinued vaccine. D3: The reported RSV GMT sample counts are 247/219 after vaccination, compared with 261/249 before vaccination; these are available samples, not adjusted-model denominators. Laboratory-exclusion counts, visit-specific sample counts and PPS counts describe different quantities. D5: The pre-enrollment SAP and documented amendment history support the planned endpoint and analysis.	ciag208 primary report Methods/Statistical analyses, participant flow (Figure 3), antibody figures and safety results; original supplementary methods/population analyses; detailed questions: EC006; folder 21, EC006

Study	Outcome / set	D1	D2	D3	D4	D5	Overall	Judgment basis	Evidence locators
Seasonal QIV NCT04841577	EC007 RSV-A neutralizing-antibody GMR Trial-defined RSV PPS; RSV PPS 427 at Day 31 versus 397 at Day 61. Figure 2 lists 3 versus 19 withdrawals at the relevant RSV visits, including 2 versus 7 AEs requiring expedited reporting. RSV-B was a planned subset (425 PPS participants).	Low	Some concerns	High	Low	Low	High	D2: RSV PPS 427 at Day 31 versus 397 at Day 61. D3: The post-RSV visit-specific losses include health-related withdrawals and differ across arms. RSV-B was measured in a planned subset. D5: The pre-enrollment SAP and documented amendment history support the planned endpoint and analysis.	ciad786 primary report Methods/Statistical analyses, participant flow (Figure 2), antibody figures and safety results; original supplementary methods/population analyses; detailed questions: EC007; folder 21, EC007
Seasonal QIV NCT04841577	EC008 RSV-B neutralizing-antibody GMR Trial-defined RSV PPS; RSV PPS 427 at Day 31 versus 397 at Day 61. Figure 2 lists 3 versus 19 withdrawals at the relevant RSV visits, including 2 versus 7 AEs requiring expedited reporting. RSV-B was a planned subset (425 PPS participants).	Low	Some concerns	High	Low	Low	High	D2: RSV PPS 427 at Day 31 versus 397 at Day 61. D3: The post-RSV visit-specific losses include health-related withdrawals and differ across arms. RSV-B was measured in a planned subset. D5: The pre-enrollment SAP and documented amendment history support the planned endpoint and analysis.	ciad786 primary report Methods/Statistical analyses, participant flow (Figure 2), antibody figures and safety results; original supplementary methods/population analyses; detailed questions: EC008; folder 21, EC008
High-dose QIV NCT05559476	EC009 RSV-A neutralizing-antibody GMR Trial-defined RSV PPS; RSV PPS flow 458/356	Low	Some concerns	Some concerns	Low	Low	Some concerns	D2: RSV PPS flow 458/356; model n 459/358 for RSV-A and 459/357 for RSV-B. D3: Broader RSV models include 493/480 (A) and 493/479 (B), well above sequential PPS retention. The remaining losses are compatible with logistical loss rather than likely response-driven absence, supporting PN for 3.4 while retaining uncertainty. D5: The pre-enrollment SAP and documented amendment history support the planned endpoint and analysis.	s40121-024-00985-4 primary report Methods/Statistical analyses, participant flow (Figure 2), antibody figures and safety results; original supplementary methods/population analyses; detailed questions: EC009; folder 21, EC009
High-dose QIV NCT05559476	EC010 RSV-B neutralizing-antibody GMR Trial-defined RSV PPS; RSV PPS flow 458/356	Low	Some concerns	Some concerns	Low	Some concerns	Some concerns	D2: RSV PPS flow 458/356; model n 459/358 for RSV-A and 459/357 for RSV-B. D3: Broader RSV models include 493/480 (A) and 493/479 (B), well above sequential PPS retention. The remaining losses are compatible with logistical loss rather than likely response-driven absence, supporting PN for 3.4 while retaining uncertainty. D5: Timing of the relevant analysis amendment relative to unblinded outcome access is uncertain.	s40121-024-00985-4 primary report Methods/Statistical analyses, participant flow (Figure 2), antibody figures and safety results; original supplementary methods/population analyses; detailed questions: EC010; folder 21, EC010
Adjuvanted QIV NCT05568797	EC011 RSV-A neutralizing-antibody GMR Trial-defined RSV PPS; RSV PPS 471/374	Low	Some concerns	Low	Low	Low	Some concerns	D2: RSV PPS 471/374; Figure 2 lists 30/91 out-of-window and 2/7 missed assessments. D3: Broader models include 1018/1045 (97.4%) for RSV-A and 1016/1045 (97.2%) for RSV-B, judged nearly complete for these continuous outcomes. The difference from the PPS primarily concerns timing/protocol eligibility, assessed in D2. D5: The pre-enrollment SAP and documented amendment history support the planned endpoint and analysis.	ciae365 primary report Methods/Statistical analyses, participant flow (Figure 2), antibody figures and safety results; original supplementary methods/population analyses; detailed questions: EC011; folder 21, EC011

Study	Outcome / set	D1	D2	D3	D4	D5	Overall	Judgment basis	Evidence locators
Adjuvanted QIV NCT05568797	EC012 RSV-B neutralizing-antibody GMR Trial-defined RSV PPS; RSV PPS 471/374	Low	Some concerns	Low	Low	Some concerns	Some concerns	D2: RSV PPS 471/374; Figure 2 lists 30/91 out-of-window and 2/7 missed assessments. D3: Broader models include 1018/1045 (97.4%) for RSV-A and 1016/1045 (97.2%) for RSV-B, judged nearly complete for these continuous outcomes. The difference from the PPS primarily concerns timing/protocol eligibility, assessed in D2. D5: Timing of the relevant analysis amendment relative to unblinded outcome access is uncertain.	ciae365 primary report Methods/Statistical analyses, participant flow (Figure 2), antibody figures and safety results; original supplementary methods/population analyses; detailed questions: EC012; folder 21, EC012
COVID-19 XBB.1.5 NCT06374394	EC013 COVID-19 mRNA, SARS-CoV-2 XBB.1.5 (NT50) Partner-specific PPS 380/381	Low	Some concerns	High	Low	Some concerns	High	D2: COVID PPS 380/381; broader antibody analysis 402/396. D3: The broader COVID model includes 798/841 randomized participants (94.9%). PPS versus exposed-set agreement does not test plausible outcome-dependent missingness, and its likely relation to the unobserved antibody response remains unresolved. D5: Timing of the relevant analysis amendment relative to unblinded outcome access is uncertain.	ciag344 primary report Methods/Statistical analyses, participant flow (Figure 3), antibody figures and safety results; original supplementary methods/population analyses; detailed questions: EC013; folder 21, EC013
RZV NCT05966090	EC014 RZV, VZV anti-gE (ELISA) Partner-specific PPS 198/170 (PPS cohort)	Low	High	High	Low	Low	High	D2: HZ PPS 198/170 (368/530; 69.4%); Figure 3: 27/60 visit-schedule exclusions, 27/30 laboratory exclusions, 5/3 withdrawals and 3/2 discontinued vaccine; reasons overlap. D3: The HZ assessment has 27/30 laboratory exclusions, beyond 5/3 withdrawals, at Day 91. No broader anti-gE analysis or missingness sensitivity analysis addresses its mechanism. D5: The pre-enrollment SAP and documented amendment history support the planned endpoint and analysis.	ciag208 primary report Methods/Statistical analyses, participant flow (Figure 3), antibody figures and safety results; original supplementary methods/population analyses; detailed questions: EC014; folder 21, EC014
Seasonal QIV NCT04841577	EC015 Seasonal QIV, A/H1N1 HI Partner-specific PPS 427/410 (post-dose PPS)	Low	Some concerns	Some concerns	Low	Low	Some concerns	D2: Influenza PPS 427/410, both at Day 31. D3: Both influenza assessments occur at Day 31. This supports probably no for a likely relation to unobserved HI response, while retaining possible missingness-related bias. D5: The pre-enrollment SAP and documented amendment history support the planned endpoint and analysis.	ciad786 primary report Methods/Statistical analyses, participant flow (Figure 2), antibody figures and safety results; original supplementary methods/population analyses; detailed questions: EC015; folder 21, EC015
Seasonal QIV NCT04841577	EC016 Seasonal QIV, A/H3N2 HI Partner-specific PPS 427/410 (post-dose PPS)	Low	Some concerns	Some concerns	Low	Low	Some concerns	D2: Influenza PPS 427/410, both at Day 31. D3: Both influenza assessments occur at Day 31. This supports probably no for a likely relation to unobserved HI response, while retaining possible missingness-related bias. D5: The pre-enrollment SAP and documented amendment history support the planned endpoint and analysis.	ciad786 primary report Methods/Statistical analyses, participant flow (Figure 2), antibody figures and safety results; original supplementary methods/population analyses; detailed questions: EC016; folder 21, EC016
Seasonal QIV NCT04841577	EC017 Seasonal QIV, B/Victoria HI Partner-specific PPS 427/410 (post-dose PPS)	Low	Some concerns	Some concerns	Low	Low	Some concerns	D2: Influenza PPS 427/410, both at Day 31. D3: Both influenza assessments occur at Day 31. This supports probably no for a likely relation to unobserved HI response, while retaining possible missingness-related bias. D5: The pre-enrollment SAP and documented amendment history support the planned endpoint and analysis.	ciad786 primary report Methods/Statistical analyses, participant flow (Figure 2), antibody figures and safety results; original supplementary methods/population analyses; detailed questions: EC017; folder 21, EC017
Seasonal QIV NCT04841577	EC018 Seasonal QIV, B/Yamagata HI Partner-specific PPS 427/410 (post-dose PPS)	Low	Some concerns	Some concerns	Low	Low	Some concerns	D2: Influenza PPS 427/410, both at Day 31. D3: Both influenza assessments occur at Day 31. This supports probably no for a likely relation to unobserved HI response, while retaining possible missingness-related bias. D5: The pre-enrollment SAP and documented amendment history support the planned endpoint and analysis.	ciad786 primary report Methods/Statistical analyses, participant flow (Figure 2), antibody figures and safety results; original supplementary methods/population analyses; detailed questions: EC018; folder 21, EC018
High-dose QIV NCT05559476	EC019 High-dose QIV, A/H1N1 HI Partner-specific PPS 452/435	Low	Some concerns	Some concerns	Low	Low	Some concerns	D2: Influenza PPS flow 458/440 at Day 31; endpoint-specific model counts and broader ES counts are reported in Table S8 and original Supplementary Table S2. D3: Both influenza assessments are at Day 31; the original Supplementary Table S2 includes 484-491/482-490 in broader models. The near-balanced available model counts and close PPS/ES HI ratios support PN for likely response-dependent missingness. D5: The pre-enrollment SAP and documented amendment history support the planned endpoint and analysis.	s40121-024-00985-4 primary report Methods/Statistical analyses, participant flow (Figure 2), antibody figures and safety results; original supplementary methods/population analyses; detailed questions: EC019; folder 21, EC019

Study	Outcome / set	D1	D2	D3	D4	D5	Overall	Judgment basis	Evidence locators
High-dose QIV NCT05559476	EC020 High-dose QIV, A/H3N2 HI Partner-specific PPS 458/441	Low	Some concerns	Some concerns	Low	Low	Some concerns	D2: Influenza PPS flow 458/440 at Day 31; endpoint-specific model counts and broader ES counts are reported in Table S8 and original Supplementary Table S2. D3: Both influenza assessments are at Day 31; the original Supplementary Table S2 includes 484-491/482-490 in broader models. The near-balanced available model counts and close PPS/ES HI ratios support PN for likely response-dependent missingness. D5: The pre-enrollment SAP and documented amendment history support the planned endpoint and analysis.	s40121-024-00985-4 primary report Methods/Statistical analyses, participant flow (Figure 2), antibody figures and safety results; original supplementary methods/population analyses; detailed questions: EC020; folder 21, EC020
High-dose QIV NCT05559476	EC021 High-dose QIV, B/Victoria HI Partner-specific PPS 458/441	Low	Some concerns	Some concerns	Low	Low	Some concerns	D2: Influenza PPS flow 458/440 at Day 31; endpoint-specific model counts and broader ES counts are reported in Table S8 and original Supplementary Table S2. D3: Both influenza assessments are at Day 31; the original Supplementary Table S2 includes 484-491/482-490 in broader models. The near-balanced available model counts and close PPS/ES HI ratios support PN for likely response-dependent missingness. D5: The pre-enrollment SAP and documented amendment history support the planned endpoint and analysis.	s40121-024-00985-4 primary report Methods/Statistical analyses, participant flow (Figure 2), antibody figures and safety results; original supplementary methods/population analyses; detailed questions: EC021; folder 21, EC021
High-dose QIV NCT05559476	EC022 High-dose QIV, B/Yamagata HI Partner-specific PPS 456/441	Low	Some concerns	Some concerns	Low	Low	Some concerns	D2: Influenza PPS flow 458/440 at Day 31; endpoint-specific model counts and broader ES counts are reported in Table S8 and original Supplementary Table S2. D3: Both influenza assessments are at Day 31; the original Supplementary Table S2 includes 484-491/482-490 in broader models. The near-balanced available model counts and close PPS/ES HI ratios support PN for likely response-dependent missingness. D5: The pre-enrollment SAP and documented amendment history support the planned endpoint and analysis.	s40121-024-00985-4 primary report Methods/Statistical analyses, participant flow (Figure 2), antibody figures and safety results; original supplementary methods/population analyses; detailed questions: EC022; folder 21, EC022
Adjuvanted QIV NCT05568797	EC023 Adjuvanted QIV, A/H1N1 HI Partner-specific PPS 440/399 (post-dose PPS)	Low	Some concerns	High	Low	Low	High	D2: Influenza PPS cohort 471/400; ES model n range 465-474/448-453 across HI antigens. D3: Broader HI models retain approximately 87-89% of randomized participants, depending on antigen, unlike the more complete RSV models. The likely relation of missingness to true HI response is unresolved. D5: The pre-enrollment SAP and documented amendment history support the planned endpoint and analysis.	ciae365 primary report Methods/Statistical analyses, participant flow (Figure 2), antibody figures and safety results; original supplementary methods/population analyses; detailed questions: EC023; folder 21, EC023
Adjuvanted QIV NCT05568797	EC024 Adjuvanted QIV, A/Darwin H3N2 HI Partner-specific PPS 442/400 (post-dose PPS)	Low	Some concerns	High	Low	Low	High	D2: Influenza PPS cohort 471/400; ES model n range 465-474/448-453 across HI antigens. D3: Broader HI models retain approximately 87-89% of randomized participants, depending on antigen, unlike the more complete RSV models. The likely relation of missingness to true HI response is unresolved. D5: The pre-enrollment SAP and documented amendment history support the planned endpoint and analysis.	ciae365 primary report Methods/Statistical analyses, participant flow (Figure 2), antibody figures and safety results; original supplementary methods/population analyses; detailed questions: EC024; folder 21, EC024
Adjuvanted QIV NCT05568797	EC025 Adjuvanted QIV, B/Victoria HI Partner-specific PPS 442/400 (post-dose PPS)	Low	Some concerns	High	Low	Low	High	D2: Influenza PPS cohort 471/400; ES model n range 465-474/448-453 across HI antigens. D3: Broader HI models retain approximately 87-89% of randomized participants, depending on antigen, unlike the more complete RSV models. The likely relation of missingness to true HI response is unresolved. D5: The pre-enrollment SAP and documented amendment history support the planned endpoint and analysis.	ciae365 primary report Methods/Statistical analyses, participant flow (Figure 2), antibody figures and safety results; original supplementary methods/population analyses; detailed questions: EC025; folder 21, EC025
Adjuvanted QIV NCT05568797	EC026 Adjuvanted QIV, B/Yamagata HI Partner-specific PPS 442/400 (post-dose PPS)	Low	Some concerns	High	Low	Low	High	D2: Influenza PPS cohort 471/400; ES model n range 465-474/448-453 across HI antigens. D3: Broader HI models retain approximately 87-89% of randomized participants, depending on antigen, unlike the more complete RSV models. The likely relation of missingness to true HI response is unresolved. D5: The pre-enrollment SAP and documented amendment history support the planned endpoint and analysis.	ciae365 primary report Methods/Statistical analyses, participant flow (Figure 2), antibody figures and safety results; original supplementary methods/population analyses; detailed questions: EC026; folder 21, EC026

Study	Outcome / set	D1	D2	D3	D4	D5	Overall	Judgment basis	Evidence locators
PCV20 NCT05879107	EC027 PCV20 OPA serotype 1 Partner-specific PPS 514/497 (cohort)	Low	High	Some concerns	Low	Low	High	D2: PCV20 PPS 514/497; 19/24 visit-schedule exclusions, 10/14 laboratory exclusions and 8/7 withdrawals. D3: Figure 3 separates the 10/14 laboratory exclusions and 8/7 withdrawals from visit and compliance exclusions. These relatively balanced losses and sample-handling causes support probably no for likely response-dependent missingness; the impact of PPS selection is judged separately in D2. D5: The pre-enrollment SAP and documented amendment history support the planned endpoint and analysis.	ciag250 primary report Methods/Statistical analyses, participant flow (Figure 3), antibody figures and safety results; original supplementary methods/population analyses; detailed questions: EC027; folder 21, EC027
PCV20 NCT05879107	EC028 PCV20 OPA serotype 3 Partner-specific PPS 514/497 (cohort)	Low	High	Some concerns	Low	Low	High	D2: PCV20 PPS 514/497; 19/24 visit-schedule exclusions, 10/14 laboratory exclusions and 8/7 withdrawals. D3: Figure 3 separates the 10/14 laboratory exclusions and 8/7 withdrawals from visit and compliance exclusions. These relatively balanced losses and sample-handling causes support probably no for likely response-dependent missingness; the impact of PPS selection is judged separately in D2. D5: The pre-enrollment SAP and documented amendment history support the planned endpoint and analysis.	ciag250 primary report Methods/Statistical analyses, participant flow (Figure 3), antibody figures and safety results; original supplementary methods/population analyses; detailed questions: EC028; folder 21, EC028
PCV20 NCT05879107	EC029 PCV20 OPA serotype 4 Partner-specific PPS 514/497 (cohort)	Low	High	Some concerns	Low	Low	High	D2: PCV20 PPS 514/497; 19/24 visit-schedule exclusions, 10/14 laboratory exclusions and 8/7 withdrawals. D3: Figure 3 separates the 10/14 laboratory exclusions and 8/7 withdrawals from visit and compliance exclusions. These relatively balanced losses and sample-handling causes support probably no for likely response-dependent missingness; the impact of PPS selection is judged separately in D2. D5: The pre-enrollment SAP and documented amendment history support the planned endpoint and analysis.	ciag250 primary report Methods/Statistical analyses, participant flow (Figure 3), antibody figures and safety results; original supplementary methods/population analyses; detailed questions: EC029; folder 21, EC029
PCV20 NCT05879107	EC030 PCV20 OPA serotype 5 Partner-specific PPS 514/497 (cohort)	Low	High	Some concerns	Low	Low	High	D2: PCV20 PPS 514/497; 19/24 visit-schedule exclusions, 10/14 laboratory exclusions and 8/7 withdrawals. D3: Figure 3 separates the 10/14 laboratory exclusions and 8/7 withdrawals from visit and compliance exclusions. These relatively balanced losses and sample-handling causes support probably no for likely response-dependent missingness; the impact of PPS selection is judged separately in D2. D5: The pre-enrollment SAP and documented amendment history support the planned endpoint and analysis.	ciag250 primary report Methods/Statistical analyses, participant flow (Figure 3), antibody figures and safety results; original supplementary methods/population analyses; detailed questions: EC030; folder 21, EC030
PCV20 NCT05879107	EC031 PCV20 OPA serotype 6A Partner-specific PPS 514/497 (cohort)	Low	High	Some concerns	Low	Low	High	D2: PCV20 PPS 514/497; 19/24 visit-schedule exclusions, 10/14 laboratory exclusions and 8/7 withdrawals. D3: Figure 3 separates the 10/14 laboratory exclusions and 8/7 withdrawals from visit and compliance exclusions. These relatively balanced losses and sample-handling causes support probably no for likely response-dependent missingness; the impact of PPS selection is judged separately in D2. D5: The pre-enrollment SAP and documented amendment history support the planned endpoint and analysis.	ciag250 primary report Methods/Statistical analyses, participant flow (Figure 3), antibody figures and safety results; original supplementary methods/population analyses; detailed questions: EC031; folder 21, EC031
PCV20 NCT05879107	EC032 PCV20 OPA serotype 6B Partner-specific PPS 514/497 (cohort)	Low	High	Some concerns	Low	Low	High	D2: PCV20 PPS 514/497; 19/24 visit-schedule exclusions, 10/14 laboratory exclusions and 8/7 withdrawals. D3: Figure 3 separates the 10/14 laboratory exclusions and 8/7 withdrawals from visit and compliance exclusions. These relatively balanced losses and sample-handling causes support probably no for likely response-dependent missingness; the impact of PPS selection is judged separately in D2. D5: The pre-enrollment SAP and documented amendment history support the planned endpoint and analysis.	ciag250 primary report Methods/Statistical analyses, participant flow (Figure 3), antibody figures and safety results; original supplementary methods/population analyses; detailed questions: EC032; folder 21, EC032
PCV20 NCT05879107	EC033 PCV20 OPA serotype 7F Partner-specific PPS 514/497 (cohort)	Low	High	Some concerns	Low	Low	High	D2: PCV20 PPS 514/497; 19/24 visit-schedule exclusions, 10/14 laboratory exclusions and 8/7 withdrawals. D3: Figure 3 separates the 10/14 laboratory exclusions and 8/7 withdrawals from visit and compliance exclusions. These relatively balanced losses and sample-handling causes support probably no for likely response-dependent missingness; the impact of PPS selection is judged separately in D2. D5: The pre-enrollment SAP and documented amendment history support the planned endpoint and analysis.	ciag250 primary report Methods/Statistical analyses, participant flow (Figure 3), antibody figures and safety results; original supplementary methods/population analyses; detailed questions: EC033; folder 21, EC033

Study	Outcome / set	D1	D2	D3	D4	D5	Overall	Judgment basis	Evidence locators
PCV20 NCT05879107	EC034 PCV20 OPA serotype 8 Partner-specific PPS 514/497 (cohort)	Low	High	Some concerns	Low	Low	High	D2: PCV20 PPS 514/497; 19/24 visit-schedule exclusions, 10/14 laboratory exclusions and 8/7 withdrawals. D3: Figure 3 separates the 10/14 laboratory exclusions and 8/7 withdrawals from visit and compliance exclusions. These relatively balanced losses and sample-handling causes support probably no for likely response-dependent missingness; the impact of PPS selection is judged separately in D2. D5: The pre-enrollment SAP and documented amendment history support the planned endpoint and analysis.	ciag250 primary report Methods/Statistical analyses, participant flow (Figure 3), antibody figures and safety results; original supplementary methods/population analyses; detailed questions: EC034; folder 21, EC034
PCV20 NCT05879107	EC035 PCV20 OPA serotype 9V Partner-specific PPS 514/497 (cohort)	Low	High	Some concerns	Low	Low	High	D2: PCV20 PPS 514/497; 19/24 visit-schedule exclusions, 10/14 laboratory exclusions and 8/7 withdrawals. D3: Figure 3 separates the 10/14 laboratory exclusions and 8/7 withdrawals from visit and compliance exclusions. These relatively balanced losses and sample-handling causes support probably no for likely response-dependent missingness; the impact of PPS selection is judged separately in D2. D5: The pre-enrollment SAP and documented amendment history support the planned endpoint and analysis.	ciag250 primary report Methods/Statistical analyses, participant flow (Figure 3), antibody figures and safety results; original supplementary methods/population analyses; detailed questions: EC035; folder 21, EC035
PCV20 NCT05879107	EC036 PCV20 OPA serotype 10A Partner-specific PPS 514/497 (cohort)	Low	High	Some concerns	Low	Low	High	D2: PCV20 PPS 514/497; 19/24 visit-schedule exclusions, 10/14 laboratory exclusions and 8/7 withdrawals. D3: Figure 3 separates the 10/14 laboratory exclusions and 8/7 withdrawals from visit and compliance exclusions. These relatively balanced losses and sample-handling causes support probably no for likely response-dependent missingness; the impact of PPS selection is judged separately in D2. D5: The pre-enrollment SAP and documented amendment history support the planned endpoint and analysis.	ciag250 primary report Methods/Statistical analyses, participant flow (Figure 3), antibody figures and safety results; original supplementary methods/population analyses; detailed questions: EC036; folder 21, EC036
PCV20 NCT05879107	EC037 PCV20 OPA serotype 11A Partner-specific PPS 514/497 (cohort)	Low	High	Some concerns	Low	Low	High	D2: PCV20 PPS 514/497; 19/24 visit-schedule exclusions, 10/14 laboratory exclusions and 8/7 withdrawals. D3: Figure 3 separates the 10/14 laboratory exclusions and 8/7 withdrawals from visit and compliance exclusions. These relatively balanced losses and sample-handling causes support probably no for likely response-dependent missingness; the impact of PPS selection is judged separately in D2. D5: The pre-enrollment SAP and documented amendment history support the planned endpoint and analysis.	ciag250 primary report Methods/Statistical analyses, participant flow (Figure 3), antibody figures and safety results; original supplementary methods/population analyses; detailed questions: EC037; folder 21, EC037
PCV20 NCT05879107	EC038 PCV20 OPA serotype 12F Partner-specific PPS 514/497 (cohort)	Low	High	Some concerns	Low	Low	High	D2: PCV20 PPS 514/497; 19/24 visit-schedule exclusions, 10/14 laboratory exclusions and 8/7 withdrawals. D3: Figure 3 separates the 10/14 laboratory exclusions and 8/7 withdrawals from visit and compliance exclusions. These relatively balanced losses and sample-handling causes support probably no for likely response-dependent missingness; the impact of PPS selection is judged separately in D2. D5: The pre-enrollment SAP and documented amendment history support the planned endpoint and analysis.	ciag250 primary report Methods/Statistical analyses, participant flow (Figure 3), antibody figures and safety results; original supplementary methods/population analyses; detailed questions: EC038; folder 21, EC038
PCV20 NCT05879107	EC039 PCV20 OPA serotype 14 Partner-specific PPS 514/497 (cohort)	Low	High	Some concerns	Low	Low	High	D2: PCV20 PPS 514/497; 19/24 visit-schedule exclusions, 10/14 laboratory exclusions and 8/7 withdrawals. D3: Figure 3 separates the 10/14 laboratory exclusions and 8/7 withdrawals from visit and compliance exclusions. These relatively balanced losses and sample-handling causes support probably no for likely response-dependent missingness; the impact of PPS selection is judged separately in D2. D5: The pre-enrollment SAP and documented amendment history support the planned endpoint and analysis.	ciag250 primary report Methods/Statistical analyses, participant flow (Figure 3), antibody figures and safety results; original supplementary methods/population analyses; detailed questions: EC039; folder 21, EC039
PCV20 NCT05879107	EC040 PCV20 OPA serotype 15B Partner-specific PPS 514/497 (cohort)	Low	High	Some concerns	Low	Low	High	D2: PCV20 PPS 514/497; 19/24 visit-schedule exclusions, 10/14 laboratory exclusions and 8/7 withdrawals. D3: Figure 3 separates the 10/14 laboratory exclusions and 8/7 withdrawals from visit and compliance exclusions. These relatively balanced losses and sample-handling causes support probably no for likely response-dependent missingness; the impact of PPS selection is judged separately in D2. D5: The pre-enrollment SAP and documented amendment history support the planned endpoint and analysis.	ciag250 primary report Methods/Statistical analyses, participant flow (Figure 3), antibody figures and safety results; original supplementary methods/population analyses; detailed questions: EC040; folder 21, EC040

Study	Outcome / set	D1	D2	D3	D4	D5	Overall	Judgment basis	Evidence locators
PCV20 NCT05879107	EC041 PCV20 OPA serotype 18C Partner-specific PPS 514/497 (cohort)	Low	High	Some concerns	Low	Low	High	D2: PCV20 PPS 514/497; 19/24 visit-schedule exclusions, 10/14 laboratory exclusions and 8/7 withdrawals. D3: Figure 3 separates the 10/14 laboratory exclusions and 8/7 withdrawals from visit and compliance exclusions. These relatively balanced losses and sample-handling causes support probably no for likely response-dependent missingness; the impact of PPS selection is judged separately in D2. D5: The pre-enrollment SAP and documented amendment history support the planned endpoint and analysis.	ciag250 primary report Methods/Statistical analyses, participant flow (Figure 3), antibody figures and safety results; original supplementary methods/population analyses; detailed questions: EC041; folder 21, EC041
PCV20 NCT05879107	EC042 PCV20 OPA serotype 19A Partner-specific PPS 514/497 (cohort)	Low	High	Some concerns	Low	Low	High	D2: PCV20 PPS 514/497; 19/24 visit-schedule exclusions, 10/14 laboratory exclusions and 8/7 withdrawals. D3: Figure 3 separates the 10/14 laboratory exclusions and 8/7 withdrawals from visit and compliance exclusions. These relatively balanced losses and sample-handling causes support probably no for likely response-dependent missingness; the impact of PPS selection is judged separately in D2. D5: The pre-enrollment SAP and documented amendment history support the planned endpoint and analysis.	ciag250 primary report Methods/Statistical analyses, participant flow (Figure 3), antibody figures and safety results; original supplementary methods/population analyses; detailed questions: EC042; folder 21, EC042
PCV20 NCT05879107	EC043 PCV20 OPA serotype 19F Partner-specific PPS 514/497 (cohort)	Low	High	Some concerns	Low	Low	High	D2: PCV20 PPS 514/497; 19/24 visit-schedule exclusions, 10/14 laboratory exclusions and 8/7 withdrawals. D3: Figure 3 separates the 10/14 laboratory exclusions and 8/7 withdrawals from visit and compliance exclusions. These relatively balanced losses and sample-handling causes support probably no for likely response-dependent missingness; the impact of PPS selection is judged separately in D2. D5: The pre-enrollment SAP and documented amendment history support the planned endpoint and analysis.	ciag250 primary report Methods/Statistical analyses, participant flow (Figure 3), antibody figures and safety results; original supplementary methods/population analyses; detailed questions: EC043; folder 21, EC043
PCV20 NCT05879107	EC044 PCV20 OPA serotype 22F Partner-specific PPS 514/497 (cohort)	Low	High	Some concerns	Low	Low	High	D2: PCV20 PPS 514/497; 19/24 visit-schedule exclusions, 10/14 laboratory exclusions and 8/7 withdrawals. D3: Figure 3 separates the 10/14 laboratory exclusions and 8/7 withdrawals from visit and compliance exclusions. These relatively balanced losses and sample-handling causes support probably no for likely response-dependent missingness; the impact of PPS selection is judged separately in D2. D5: The pre-enrollment SAP and documented amendment history support the planned endpoint and analysis.	ciag250 primary report Methods/Statistical analyses, participant flow (Figure 3), antibody figures and safety results; original supplementary methods/population analyses; detailed questions: EC044; folder 21, EC044
PCV20 NCT05879107	EC045 PCV20 OPA serotype 23F Partner-specific PPS 514/497 (cohort)	Low	High	Some concerns	Low	Low	High	D2: PCV20 PPS 514/497; 19/24 visit-schedule exclusions, 10/14 laboratory exclusions and 8/7 withdrawals. D3: Figure 3 separates the 10/14 laboratory exclusions and 8/7 withdrawals from visit and compliance exclusions. These relatively balanced losses and sample-handling causes support probably no for likely response-dependent missingness; the impact of PPS selection is judged separately in D2. D5: The pre-enrollment SAP and documented amendment history support the planned endpoint and analysis.	ciag250 primary report Methods/Statistical analyses, participant flow (Figure 3), antibody figures and safety results; original supplementary methods/population analyses; detailed questions: EC045; folder 21, EC045
PCV20 NCT05879107	EC046 PCV20 OPA serotype 33F Partner-specific PPS 514/497 (cohort)	Low	High	Some concerns	Low	Low	High	D2: PCV20 PPS 514/497; 19/24 visit-schedule exclusions, 10/14 laboratory exclusions and 8/7 withdrawals. D3: Figure 3 separates the 10/14 laboratory exclusions and 8/7 withdrawals from visit and compliance exclusions. These relatively balanced losses and sample-handling causes support probably no for likely response-dependent missingness; the impact of PPS selection is judged separately in D2. D5: The pre-enrollment SAP and documented amendment history support the planned endpoint and analysis.	ciag250 primary report Methods/Statistical analyses, participant flow (Figure 3), antibody figures and safety results; original supplementary methods/population analyses; detailed questions: EC046; folder 21, EC046
PCV20 NCT05879107	EC047 30-day any unsolicited AE exposed set; 553/557	Low	Low	High	Some concerns	Low	High	D2: Vaccinated participants remain in their assigned schedule for safety analysis; the reports show no substantive crossover. D3: Withdrawal information at the relevant post-vaccination visits does not establish complete AE status through every collection window; the relation between incomplete reporting and the outcome is uncertain. D4: Open-label reporting may influence event assessment; both groups receive the active vaccines under the same definitions and grading procedures. D5: The pre-enrollment SAP and documented amendment history support the planned endpoint and analysis.	ciag250 primary report Methods/Statistical analyses, participant flow (Figure 3), antibody figures and safety results; original supplementary methods/population analyses; detailed questions: EC047; folder 21, EC047

Study	Outcome / set	D1	D2	D3	D4	D5	Overall	Judgment basis	Evidence locators
COVID-19 XBB.1.5 NCT06374394	EC048 30-day any unsolicited AE exposed population; 417/416	Low	Low	High	Some concerns	Some concerns	High	D2: Vaccinated participants remain in their assigned schedule for safety analysis; the reports show no substantive crossover. D3: Withdrawal information at the relevant post-vaccination visits does not establish complete AE status through every collection window; the relation between incomplete reporting and the outcome is uncertain. D4: Open-label reporting may influence event assessment; both groups receive the active vaccines under the same definitions and grading procedures. D5: Timing of the relevant analysis amendment relative to unblinded outcome access is uncertain.	ciag344 primary report Methods/Statistical analyses, participant flow (Figure 3), antibody figures and safety results; original supplementary methods/population analyses; detailed questions: EC048; folder 21, EC048
RZV NCT05966090	EC049 30-day any unsolicited AE exposed set; 265/265	Low	Low	High	Some concerns	Low	High	D2: Vaccinated participants remain in their assigned schedule for safety analysis; the reports show no substantive crossover. D3: Withdrawal information at the relevant post-vaccination visits does not establish complete AE status through every collection window; the relation between incomplete reporting and the outcome is uncertain. D4: Open-label reporting may influence event assessment; both groups receive the active vaccines under the same definitions and grading procedures. D5: The pre-enrollment SAP and documented amendment history support the planned endpoint and analysis.	ciag208 primary report Methods/Statistical analyses, participant flow (Figure 3), antibody figures and safety results; original supplementary methods/population analyses; detailed questions: EC049; folder 21, EC049
Seasonal QIV NCT04841577	EC050 30-day any unsolicited AE exposed set; 442/443	Low	Low	High	Some concerns	Low	High	D2: Vaccinated participants remain in their assigned schedule for safety analysis; the reports show no substantive crossover. D3: Withdrawal information at the relevant post-vaccination visits does not establish complete AE status through every collection window; the relation between incomplete reporting and the outcome is uncertain. D4: Open-label reporting may influence event assessment; both groups receive the active vaccines under the same definitions and grading procedures. D5: The pre-enrollment SAP and documented amendment history support the planned endpoint and analysis.	ciad786 primary report Methods/Statistical analyses, participant flow (Figure 2), antibody figures and safety results; original supplementary methods/population analyses; detailed questions: EC050; folder 21, EC050
High-dose QIV NCT05559476	EC051 30-day any unsolicited AE exposed set; 516/513	Low	Low	High	Some concerns	Low	High	D2: Vaccinated participants remain in their assigned schedule for safety analysis; the reports show no substantive crossover. D3: Withdrawal information at the relevant post-vaccination visits does not establish complete AE status through every collection window; the relation between incomplete reporting and the outcome is uncertain. D4: Open-label reporting may influence event assessment; both groups receive the active vaccines under the same definitions and grading procedures. D5: The pre-enrollment SAP and documented amendment history support the planned endpoint and analysis.	s40121-024-00985-4 primary report Methods/Statistical analyses, participant flow (Figure 2), antibody figures and safety results; original supplementary methods/population analyses; detailed questions: EC051; folder 21, EC051
Adjuvanted QIV NCT05568797	EC052 30-day any unsolicited AE exposed set; 523/522	Low	Low	High	Some concerns	Low	High	D2: Vaccinated participants remain in their assigned schedule for safety analysis; the reports show no substantive crossover. D3: Withdrawal information at the relevant post-vaccination visits does not establish complete AE status through every collection window; the relation between incomplete reporting and the outcome is uncertain. D4: Open-label reporting may influence event assessment; both groups receive the active vaccines under the same definitions and grading procedures. D5: The pre-enrollment SAP and documented amendment history support the planned endpoint and analysis.	ciae365 primary report Methods/Statistical analyses, participant flow (Figure 2), antibody figures and safety results; original supplementary methods/population analyses; detailed questions: EC052; folder 21, EC052
PCV20 NCT05879107	EC053 SAE through study end exposed set; 553/557	Low	Low	High	Some concerns	Low	High	D2: Vaccinated participants remain in their assigned schedule for safety analysis; the reports show no substantive crossover. D3: For this uncommon outcome, the documented losses cannot be assumed immaterial from the overall retention percentage. No missing-outcome sensitivity analysis resolves the mechanism; 3.4 remains NI. D4: Open-label reporting may influence event assessment; both groups receive the active vaccines under the same definitions and grading procedures. D5: The pre-enrollment SAP and documented amendment history support the planned endpoint and analysis.	ciag250 primary report Methods/Statistical analyses, participant flow (Figure 3), antibody figures and safety results; original supplementary methods/population analyses; detailed questions: EC053; folder 21, EC053

Study	Outcome / set	D1	D2	D3	D4	D5	Overall	Judgment basis	Evidence locators
COVID-19 XBB.1.5 NCT06374394	EC054 SAE through study end exposed population; 417/416	Low	Low	High	Some concerns	Some concerns	High	D2: Vaccinated participants remain in their assigned schedule for safety analysis; the reports show no substantive crossover. D3: For this uncommon outcome, the documented losses cannot be assumed immaterial from the overall retention percentage. No missing-outcome sensitivity analysis resolves the mechanism; 3.4 remains NI. D4: Open-label reporting may influence event assessment; both groups receive the active vaccines under the same definitions and grading procedures. D5: Timing of the relevant analysis amendment relative to unblinded outcome access is uncertain.	ciag344 primary report Methods/Statistical analyses, participant flow (Figure 3), antibody figures and safety results; original supplementary methods/population analyses; detailed questions: EC054; folder 21, EC054
RZV NCT05966090	EC055 SAE through study end exposed set; 265/265	Low	Low	High	Some concerns	Low	High	D2: Vaccinated participants remain in their assigned schedule for safety analysis; the reports show no substantive crossover. D3: For this uncommon outcome, the documented losses cannot be assumed immaterial from the overall retention percentage. No missing-outcome sensitivity analysis resolves the mechanism; 3.4 remains NI. D4: Open-label reporting may influence event assessment; both groups receive the active vaccines under the same definitions and grading procedures. D5: The pre-enrollment SAP and documented amendment history support the planned endpoint and analysis.	ciag208 primary report Methods/Statistical analyses, participant flow (Figure 3), antibody figures and safety results; original supplementary methods/population analyses; detailed questions: EC055; folder 21, EC055
Seasonal QIV NCT04841577	EC056 SAE through study end exposed set; 442/443	Low	Low	High	Some concerns	Low	High	D2: Vaccinated participants remain in their assigned schedule for safety analysis; the reports show no substantive crossover. D3: For this uncommon outcome, the documented losses cannot be assumed immaterial from the overall retention percentage. No missing-outcome sensitivity analysis resolves the mechanism; 3.4 remains NI. D4: Open-label reporting may influence event assessment; both groups receive the active vaccines under the same definitions and grading procedures. D5: The pre-enrollment SAP and documented amendment history support the planned endpoint and analysis.	ciad786 primary report Methods/Statistical analyses, participant flow (Figure 2), antibody figures and safety results; original supplementary methods/population analyses; detailed questions: EC056; folder 21, EC056
High-dose QIV NCT05559476	EC057 SAE through study end exposed set; 516/513	Low	Low	High	Some concerns	Low	High	D2: Vaccinated participants remain in their assigned schedule for safety analysis; the reports show no substantive crossover. D3: For this uncommon outcome, the documented losses cannot be assumed immaterial from the overall retention percentage. No missing-outcome sensitivity analysis resolves the mechanism; 3.4 remains NI. D4: Open-label reporting may influence event assessment; both groups receive the active vaccines under the same definitions and grading procedures. D5: The pre-enrollment SAP and documented amendment history support the planned endpoint and analysis.	s40121-024-00985-4 primary report Methods/Statistical analyses, participant flow (Figure 2), antibody figures and safety results; original supplementary methods/population analyses; detailed questions: EC057; folder 21, EC057
Adjuvanted QIV NCT05568797	EC058 SAE through study end exposed set; 523/522	Low	Low	High	Some concerns	Low	High	D2: Vaccinated participants remain in their assigned schedule for safety analysis; the reports show no substantive crossover. D3: For this uncommon outcome, the documented losses cannot be assumed immaterial from the overall retention percentage. No missing-outcome sensitivity analysis resolves the mechanism; 3.4 remains NI. D4: Open-label reporting may influence event assessment; both groups receive the active vaccines under the same definitions and grading procedures. D5: The pre-enrollment SAP and documented amendment history support the planned endpoint and analysis.	ciae365 primary report Methods/Statistical analyses, participant flow (Figure 2), antibody figures and safety results; original supplementary methods/population analyses; detailed questions: EC058; folder 21, EC058
PCV20 NCT05879107	EC059 30-day grade 3 unsolicited AE exposed set; 553/557	Low	Low	High	Some concerns	Low	High	D2: Vaccinated participants remain in their assigned schedule for safety analysis; the reports show no substantive crossover. D3: Withdrawal information at the relevant post-vaccination visits does not establish complete AE status through every collection window; the relation between incomplete reporting and the outcome is uncertain. D4: Open-label reporting may influence event assessment; both groups receive the active vaccines under the same definitions and grading procedures. D5: The pre-enrollment SAP and documented amendment history support the planned endpoint and analysis.	ciag250 primary report Methods/Statistical analyses, participant flow (Figure 3), antibody figures and safety results; original supplementary methods/population analyses; detailed questions: EC059; folder 21, EC059

Study	Outcome / set	D1	D2	D3	D4	D5	Overall	Judgment basis	Evidence locators
COVID-19 XBB.1.5 NCT06374394	EC060 30-day grade 3 unsolicited AE exposed population; 417/416	Low	Low	High	Some concerns	Some concerns	High	D2: Vaccinated participants remain in their assigned schedule for safety analysis; the reports show no substantive crossover. D3: Withdrawal information at the relevant post-vaccination visits does not establish complete AE status through every collection window; the relation between incomplete reporting and the outcome is uncertain. D4: Open-label reporting may influence event assessment; both groups receive the active vaccines under the same definitions and grading procedures. D5: Timing of the relevant analysis amendment relative to unblinded outcome access is uncertain.	ciag344 primary report Methods/Statistical analyses, participant flow (Figure 3), antibody figures and safety results; original supplementary methods/population analyses; detailed questions: EC060; folder 21, EC060
RZV NCT05966090	EC061 30-day grade 3 unsolicited AE exposed set; 265/265	Low	Low	High	Some concerns	Low	High	D2: Vaccinated participants remain in their assigned schedule for safety analysis; the reports show no substantive crossover. D3: Withdrawal information at the relevant post-vaccination visits does not establish complete AE status through every collection window; the relation between incomplete reporting and the outcome is uncertain. D4: Open-label reporting may influence event assessment; both groups receive the active vaccines under the same definitions and grading procedures. D5: The pre-enrollment SAP and documented amendment history support the planned endpoint and analysis.	ciag208 primary report Methods/Statistical analyses, participant flow (Figure 3), antibody figures and safety results; original supplementary methods/population analyses; detailed questions: EC061; folder 21, EC061
Seasonal QIV NCT04841577	EC062 30-day grade 3 unsolicited AE exposed set; 442/443	Low	Low	High	Some concerns	Low	High	D2: Vaccinated participants remain in their assigned schedule for safety analysis; the reports show no substantive crossover. D3: Withdrawal information at the relevant post-vaccination visits does not establish complete AE status through every collection window; the relation between incomplete reporting and the outcome is uncertain. D4: Open-label reporting may influence event assessment; both groups receive the active vaccines under the same definitions and grading procedures. D5: The pre-enrollment SAP and documented amendment history support the planned endpoint and analysis.	ciad786 primary report Methods/Statistical analyses, participant flow (Figure 2), antibody figures and safety results; original supplementary methods/population analyses; detailed questions: EC062; folder 21, EC062
Adjuvanted QIV NCT05568797	EC063 30-day grade 3 unsolicited AE exposed set; 523/522	Low	Low	High	Some concerns	Low	High	D2: Vaccinated participants remain in their assigned schedule for safety analysis; the reports show no substantive crossover. D3: Withdrawal information at the relevant post-vaccination visits does not establish complete AE status through every collection window; the relation between incomplete reporting and the outcome is uncertain. D4: Open-label reporting may influence event assessment; both groups receive the active vaccines under the same definitions and grading procedures. D5: The pre-enrollment SAP and documented amendment history support the planned endpoint and analysis.	ciae365 primary report Methods/Statistical analyses, participant flow (Figure 2), antibody figures and safety results; original supplementary methods/population analyses; detailed questions: EC063; folder 21, EC063
Seasonal QIV NCT04841577	EC064 Death through study end exposed set; 442/443	Low	Low	High	Low	Low	High	D2: Vaccinated participants remain in their assigned schedule for safety analysis; the reports show no substantive crossover. D3: For this uncommon outcome, the documented losses cannot be assumed immaterial from the overall retention percentage. No missing-outcome sensitivity analysis resolves the mechanism; 3.4 remains NI. D5: The pre-enrollment SAP and documented amendment history support the planned endpoint and analysis.	ciad786 primary report Methods/Statistical analyses, participant flow (Figure 2), antibody figures and safety results; original supplementary methods/population analyses; detailed questions: EC064; folder 21, EC064
High-dose QIV NCT05559476	EC065 Death through study end exposed set; 516/513	Low	Low	High	Low	Low	High	D2: Vaccinated participants remain in their assigned schedule for safety analysis; the reports show no substantive crossover. D3: For this uncommon outcome, the documented losses cannot be assumed immaterial from the overall retention percentage. No missing-outcome sensitivity analysis resolves the mechanism; 3.4 remains NI. D5: The pre-enrollment SAP and documented amendment history support the planned endpoint and analysis.	s40121-024-00985-4 primary report Methods/Statistical analyses, participant flow (Figure 2), antibody figures and safety results; original supplementary methods/population analyses; detailed questions: EC065; folder 21, EC065

Study	Outcome / set	D1	D2	D3	D4	D5	Overall	Judgment basis	Evidence locators
Adjuvanted QIV NCT05568797	EC066 Death through study end exposed set; 523/522	Low	Low	High	Low	Low	High	D2: Vaccinated participants remain in their assigned schedule for safety analysis; the reports show no substantive crossover. D3: For this uncommon outcome, the documented losses cannot be assumed immaterial from the overall retention percentage. No missing-outcome sensitivity analysis resolves the mechanism; 3.4 remains NI. D5: The pre-enrollment SAP and documented amendment history support the planned endpoint and analysis.	ciae365 primary report Methods/Statistical analyses, participant flow (Figure 2), antibody figures and safety results; original supplementary methods/population analyses; detailed questions: EC066; folder 21, EC066
PCV20 NCT05879107	EC067 Death through study end exposed set; 553/557	Low	Low	High	Low	Low	High	D2: Vaccinated participants remain in their assigned schedule for safety analysis; the reports show no substantive crossover. D3: For this uncommon outcome, the documented losses cannot be assumed immaterial from the overall retention percentage. No missing-outcome sensitivity analysis resolves the mechanism; 3.4 remains NI. D5: The pre-enrollment SAP and documented amendment history support the planned endpoint and analysis.	ciag250 primary report Methods/Statistical analyses, participant flow (Figure 3), antibody figures and safety results; original supplementary methods/population analyses; detailed questions: EC067; folder 21, EC067
RZV NCT05966090	EC068 Death through study end exposed set; 265/265	Low	Low	High	Low	Low	High	D2: Vaccinated participants remain in their assigned schedule for safety analysis; the reports show no substantive crossover. D3: For this uncommon outcome, the documented losses cannot be assumed immaterial from the overall retention percentage. No missing-outcome sensitivity analysis resolves the mechanism; 3.4 remains NI. D5: The pre-enrollment SAP and documented amendment history support the planned endpoint and analysis.	ciag208 primary report Methods/Statistical analyses, participant flow (Figure 3), antibody figures and safety results; original supplementary methods/population analyses; detailed questions: EC068; folder 21, EC068
COVID-19 XBB.1.5 NCT06374394	EC069 Death through study end exposed population; 417/416	Low	Low	High	Low	Some concerns	High	D2: Vaccinated participants remain in their assigned schedule for safety analysis; the reports show no substantive crossover. D3: For this uncommon outcome, the documented losses cannot be assumed immaterial from the overall retention percentage. No missing-outcome sensitivity analysis resolves the mechanism; 3.4 remains NI. D5: Timing of the relevant analysis amendment relative to unblinded outcome access is uncertain.	ciag344 primary report Methods/Statistical analyses, participant flow (Figure 3), antibody figures and safety results; original supplementary methods/population analyses; detailed questions: EC069; folder 21, EC069
Seasonal QIV NCT04841577	EC070 4-day any solicited local AE ES with diary data; 438/438	Low	Low	Low	Some concerns	Low	Some concerns	D2: Vaccinated participants remain in their assigned schedule for safety analysis; the reports show no substantive crossover. D3: Outcome data cover 876/890 randomized participants (98.4%), and 876/885 exposed participants (99.0%). For these frequent local/systemic events, this is judged nearly complete; subsequent D3 questions are NA. D4: Open-label reporting may influence event assessment; both groups receive the active vaccines under the same definitions and grading procedures. D5: The pre-enrollment SAP and documented amendment history support the planned endpoint and analysis.	ciad786 primary report Methods/Statistical analyses, participant flow (Figure 2), antibody figures and safety results; original supplementary methods/population analyses; detailed questions: EC070; folder 21, EC070
High-dose QIV NCT05559476	EC071 4-day any solicited local AE modified safety set; 423/417	Low	High	High	Some concerns	Some concerns	High	D2: Exposed participants 516/513; 51 withdrew (23/28); 25 lost to follow-up and 22 discontinued for reasons unrelated to AEs/SAEs. D3: Diary validity exclusions plus additional unavailable endpoint data remove approximately 18-19% of the exposed groups from these results. No adequate missing-diary sensitivity analysis is supplied; 3.4 remains NI. D4: Open-label reporting may influence event assessment; both groups receive the active vaccines under the same definitions and grading procedures. D5: Timing of the relevant analysis amendment relative to unblinded outcome access is uncertain.	s40121-024-00985-4 primary report Methods/Statistical analyses, participant flow (Figure 2), antibody figures and safety results; original supplementary methods/population analyses; detailed questions: EC071; folder 21, EC071
Seasonal QIV NCT04841577	EC072 4-day any solicited systemic AE ES with diary data; 438/438	Low	Low	Low	Some concerns	Low	Some concerns	D2: Vaccinated participants remain in their assigned schedule for safety analysis; the reports show no substantive crossover. D3: Outcome data cover 876/890 randomized participants (98.4%), and 876/885 exposed participants (99.0%). For these frequent local/systemic events, this is judged nearly complete; subsequent D3 questions are NA. D4: Open-label reporting may influence event assessment; both groups receive the active vaccines under the same definitions and grading procedures. D5: The pre-enrollment SAP and documented amendment history support the planned endpoint and analysis.	ciad786 primary report Methods/Statistical analyses, participant flow (Figure 2), antibody figures and safety results; original supplementary methods/population analyses; detailed questions: EC072; folder 21, EC072

Study	Outcome / set	D1	D2	D3	D4	D5	Overall	Judgment basis	Evidence locators
High-dose QIV NCT05559476	EC073 4-day any solicited systemic AE modified safety set; 423/417	Low	High	High	Some concerns	Some concerns	High	D2: Exposed participants 516/513; 51 withdrew (23/28); 25 lost to follow-up and 22 discontinued for reasons unrelated to AEs/SAEs. D3: Diary validity exclusions plus additional unavailable endpoint data remove approximately 18-19% of the exposed groups from these results. No adequate missing-diary sensitivity analysis is supplied; 3.4 remains NI. D4: Open-label reporting may influence event assessment; both groups receive the active vaccines under the same definitions and grading procedures. D5: Timing of the relevant analysis amendment relative to unblinded outcome access is uncertain.	s40121-024-00985-4 primary report Methods/Statistical analyses, participant flow (Figure 2), antibody figures and safety results; original supplementary methods/population analyses; detailed questions: EC073; folder 21, EC073

RoB 2 signalling questions and response records

RoB 2 question	Question focus	Detailed responses
1.1	Random allocation sequence	20_RoB2_verification/RoB2_signalling_responses.csv; assessment IDs EC001–EC073
1.2	Concealed allocation	20_RoB2_verification/RoB2_signalling_responses.csv; assessment IDs EC001–EC073
1.3	Baseline imbalance suggesting randomization problems	20_RoB2_verification/RoB2_signalling_responses.csv; assessment IDs EC001–EC073
2.1	Participant awareness	20_RoB2_verification/RoB2_signalling_responses.csv; assessment IDs EC001–EC073
2.2	Care-provider awareness	20_RoB2_verification/RoB2_signalling_responses.csv; assessment IDs EC001–EC073
2.3	Trial-context deviations	20_RoB2_verification/RoB2_signalling_responses.csv; assessment IDs EC001–EC073
2.4	Effect of deviations on outcome	20_RoB2_verification/RoB2_signalling_responses.csv; assessment IDs EC001–EC073
2.5	Balance of deviations	20_RoB2_verification/RoB2_signalling_responses.csv; assessment IDs EC001–EC073
2.6	Analysis appropriate for assignment effect	20_RoB2_verification/RoB2_signalling_responses.csv; assessment IDs EC001–EC073
2.7	Potential substantial impact of analysis exclusions	20_RoB2_verification/RoB2_signalling_responses.csv; assessment IDs EC001–EC073
3.1	Outcome data available for all or nearly all participants	20_RoB2_verification/RoB2_signalling_responses.csv; assessment IDs EC001–EC073
3.2	Evidence that missing data did not bias the result	20_RoB2_verification/RoB2_signalling_responses.csv; assessment IDs EC001–EC073
3.3	Possible dependence of missingness on true outcome	20_RoB2_verification/RoB2_signalling_responses.csv; assessment IDs EC001–EC073
3.4	Likely dependence of missingness on true outcome	20_RoB2_verification/RoB2_signalling_responses.csv; assessment IDs EC001–EC073

RoB 2 question	Question focus	Detailed responses
4.1	Inappropriate measurement method	20_RoB2_verification/RoB2_signalling_responses.csv; assessment IDs EC001–EC073
4.2	Measurement differs between arms	20_RoB2_verification/RoB2_signalling_responses.csv; assessment IDs EC001–EC073
4.3	Assessor awareness	20_RoB2_verification/RoB2_signalling_responses.csv; assessment IDs EC001–EC073
4.4	Possible influence of awareness	20_RoB2_verification/RoB2_signalling_responses.csv; assessment IDs EC001–EC073
4.5	Likely influence of awareness	20_RoB2_verification/RoB2_signalling_responses.csv; assessment IDs EC001–EC073
5.1	Analysis plan established before unblinded results	20_RoB2_verification/RoB2_signalling_responses.csv; assessment IDs EC001–EC073
5.2	Selection among eligible outcome measurements	20_RoB2_verification/RoB2_signalling_responses.csv; assessment IDs EC001–EC073
5.3	Selection among eligible analyses	20_RoB2_verification/RoB2_signalling_responses.csv; assessment IDs EC001–EC073

The full RoB 2 question identifiers are mapped below. Supplementary Data and Code, folder 20_RoB2_verification, provides the result-level assessments and signalling responses with source-based rationales. Folder 21_trial_plan_and_followup provides the comparisons of trial plans with published results and the follow-up evidence for the adverse-event collection windows. Y, yes; PY, probably yes; PN, probably no; N, no; NI, no information; NA, conditional question not applicable. The accompanying RoB 2 guidance provides the official wording and decision tables.

Table S1. Reports assessed for eligibility (n = 215).

Record	Report and bibliographic identifier	Report type	Eligibility disposition
000001	Duckett, M. et al.<br>1076 Community Pharmacy Outreach Program to Promote RSV Vaccination in Eligible Older Adults and Improve Vaccination Rates<br>Journal of the American Pharmacists Association (2025)<br>DOI: 10.1016/j.japh.2025.102563	Conference abstract	Excluded<br>Community pharmacy outreach evaluates vaccination uptake, without randomized assignment to coadministration or sequential administration.
000005	Weiser, S. et al.<br>1100 Preparation Time and Usability Assessment of Commercially Available Freeze-Dried Respiratory Syncytial Virus Vaccine Presentations<br>Journal of the American Pharmacists Association (2025)<br>DOI: 10.1016/j.japh.2025.102587	Conference abstract	Excluded<br>Time-and-motion and usability assessment of vaccine preparation by healthcare providers, rather than a clinical vaccination-schedule trial.

Record	Report and bibliographic identifier	Report type	Eligibility disposition
000007	2019 IDSOG Annual Meeting<br>American Journal of Obstetrics and Gynecology (2019); pp. A1–A18<br>DOI: 10.1016/s0002-9378(19)31324-9	Meeting collection	Excluded<br>Meeting collection indexed as a conference review; its DOI identifies a table of contents, not an individual eligible trial report.
000028	A Partially Randomized, Partly Placebo Controlled Phase I Trial to Evaluate the Safety, Tolerability and Immunogenicity of the Recombinant MVA-BN® RSV Vaccine After Intranasal and Intramuscular Administration<br>ClinicalTrials.gov (2016)<br>NCT02864628	ClinicalTrials.gov registry report	Excluded<br>MVA-BN-RSV is a viral-vector vaccine, outside the RSVPreF3 OA product scope.
000029	A PHASE 1/2, PLACEBO-CONTROLLED, RANDOMIZED, OBSERVER-BLIND, DOSE-FINDING, FIRST-IN-HUMAN STUDY TO DESCRIBE THE SAFETY, TOLERABILITY, AND IMMUNOGENICITY OF A RESPIRATORY SYNCYTIAL VIRUS (RSV) VACCINE IN HEALTHY ADULTS<br>ClinicalTrials.gov (2018)<br>NCT03529773	ClinicalTrials.gov registry report	Excluded<br>Pfizer RSVpreF is a different RSV vaccine product from adjuvanted RSVPreF3 OA.
000030	A PHASE 1/2, PLACEBO-CONTROLLED, RANDOMIZED, OBSERVER-BLIND,DOSE-FINDING, FIRST-IN-HUMAN STUDY TO DESCRIBE THE SAFETY, TOLERABILITY, AND IMMUNOGENICITY OF AN ADJUVANTED RESPIRATORY SYNCYTIAL VIRUS (RSV) VACCINE IN HEALTHY OLDER ADULTS<br>ClinicalTrials.gov (2018)<br>NCT03572062	ClinicalTrials.gov registry report	Excluded<br>Pfizer RSVpreF is a different RSV vaccine product from adjuvanted RSVPreF3 OA.
000034	Baber, J et al.<br>A Phase 1/2 Study of a Respiratory Syncytial Virus Prefusion F Vaccine With and Without Adjuvant in Healthy Older Adults<br>Journal Of Infectious Diseases (2022); pp. 2054–2063<br>DOI: 10.1093/infdis/jiac189<br>NCT03572062	Journal article	Excluded<br>Pfizer RSVpreF is a different RSV vaccine product from adjuvanted RSVPreF3 OA.
000039	A Phase 1, Randomized, Double-Blind, Placebo-Controlled, Dose-Ranging Trial to Determine the Safety and Immunogenicity of an Adenoviral-Vector Based Respiratory Syncytial Virus (RSV) F Protein Vaccine (VXA-RSV-f) Expressing Protein F and dsRNA Adjuvant Administered Orally to Healthy Volunteers<br>ClinicalTrials.gov (2016)<br>NCT02830932	ClinicalTrials.gov registry report	Excluded<br>Oral adenoviral-vector vaccine VXA-RSV-f, rather than RSVPreF3 OA.

Record	Report and bibliographic identifier	Report type	Eligibility disposition
000041	A Phase 1 Randomized, Observer Blind, Placebo Controlled, Dosage-Escalation Single Center Study to Evaluate the Safety and Immunogenicity of an RSV Fusion Glycoprotein (F) Subunit Vaccine in Healthy Adults<br>ClinicalTrials.gov (2014)<br>NCT02298179	ClinicalTrials.gov registry report	Excluded<br>Dose-escalation study of two RSV F subunit doses versus placebo; no randomized partner-vaccine coadministration versus sequential comparison.
000042	A Phase 1 Randomized, Observer-Blinded,Placebo-Controlled Trial to Evaluate the Safety and Immunogenicity of a Recombinant Respiratory Syncytial Virus F Protein Particle Vaccine in Healthy Adults<br>ClinicalTrials.gov (2011)<br>NCT01290419	ClinicalTrials.gov registry report	Excluded<br>RSV F protein nanoparticle vaccine, rather than adjuvanted RSVPreF3 OA.
000048	A Phase 1b Study to Evaluate the Safety and Immunogenicity of MEDI7510 in Older Adults<br>ClinicalTrials.gov (2014)<br>NCT02289820	ClinicalTrials.gov registry report	Excluded<br>MEDI7510 is a different RSV vaccine product from RSVPreF3 OA.
000056	A Phase 2a, Randomised, Double-Blinded, Placebo-Controlled Study to Assess the Safety, Immunogenicity and Efficacy of the Recombinant MVA-BN®-RSV Vaccine Against Respiratory Syncytial Virus Infection in the Virus Challenge Model in Healthy Adult Participants<br>ClinicalTrials.gov (2021)<br>NCT04752644	ClinicalTrials.gov registry report	Excluded<br>MVA-BN-RSV is a viral-vector vaccine, outside the RSVPreF3 OA product scope.
000057	A Phase 2b, Non-randomized, Controlled, Open-label, Extension Study to Evaluate the Persistence of Immune Response of the Adjuvanted RSVPreF3 Vaccine and the Safety and Immunogenicity Following Revaccination in Lung and Kidney Transplant Recipients (>=18 Years of Age)<br>ClinicalTrials.gov (2025)<br>NCT07092865	ClinicalTrials.gov registry report	Excluded<br>Nonrandomized extension of an RSV vaccination study in transplant recipients; evaluates persistence and revaccination rather than assigned partner-vaccine schedules.
000059	A PHASE 2b, PLACEBO-CONTROLLED, RANDOMIZED, OBSERVER-BLIND STUDY TO EVALUATE THE SAFETY, TOLERABILITY, AND IMMUNOGENICITY OF A RESPIRATORY SYNCYTIAL VIRUS (RSV) VACCINE WHEN ADMINISTERED CONCOMITANTLY WITH TETANUS, DIPHTHERIA, AND ACELLULAR PERTUSSIS VACCINE (TDAP) IN HEALTHY NONPREGNANT WOMEN 18 THROUGH 49 YEARS OF AGE<br>ClinicalTrials.gov (2019)<br>NCT04071158	ClinicalTrials.gov registry report	Excluded<br>Pfizer RSVpreF is a different RSV vaccine product from adjuvanted RSVPreF3 OA.

Record	Report and bibliographic identifier	Report type	Eligibility disposition
000061	A PHASE 3, MULTICENTER, PARALLEL-GROUP, OPEN-LABEL STUDY TO EVALUATE THE SAFETY, TOLERABILITY, AND IMMUNOGENICITY OF RESPIRATORY SYNCYTIAL VIRUS PREFUSION F SUBUNIT VACCINE WHEN COADMINISTERED WITH HERPES ZOSTER SUBUNIT VACCINE IN ADULTS ≥50 YEARS OF AGE<br>ClinicalTrials.gov (2025)<br>NCT06890416	ClinicalTrials.gov registry report	Excluded<br>Pfizer RSVpreF is a different RSV vaccine product from adjuvanted RSVPreF3 OA.
000062	A PHASE 3, MULTICENTER, SINGLE-ARM, OPEN-LABEL STUDY TO EVALUATE THE SAFETY, TOLERABILITY, AND IMMUNOGENICITY OF RESPIRATORY SYNCYTIAL VIRUS (RSV) PREFUSION F SUBUNIT VACCINE IN ADULTS CONSIDERED AT HIGH RISK OF SEVERE RSV DISEASE IN JAPAN<br>ClinicalTrials.gov (2026)<br>NCT07543380	ClinicalTrials.gov registry report	Excluded<br>Pfizer RSVpreF is a different RSV vaccine product from adjuvanted RSVPreF3 OA.
000063	A Phase 3, Open-label, Randomized, Controlled, Multi-country Study to Evaluate the Immune Response, Safety and Reactogenicity of RSVPreF3 OA Investigational Vaccine When Co-administered With FLU-QIV Vaccine in Adults Aged 60 Years and Above<br>ClinicalTrials.gov (2021)<br>NCT04841577	ClinicalTrials.gov registry report	Associated report<br>Associated registry record of NCT04841577.
000064	A Phase 3, Open-label, Randomized, Controlled, Multicountry Study to Evaluate the Immune Response, Safety and Reactogenicity of RSVPreF3 OA Investigational Vaccine When Co-administered With FLU HD Vaccine in Adults Aged 65 Years and Above<br>ClinicalTrials.gov (2022)<br>NCT05559476	ClinicalTrials.gov registry report	Associated report<br>Associated registry record of NCT05559476.
000065	A Phase 3, Open-label, Randomized, Controlled Study to Evaluate the Immune Response, Safety and Reactogenicity of RSVPreF3 OA Investigational Vaccine When Co-Administered With a COVID-19 mRNA Vaccine (Omicron XBB.1.5) in Adults Aged 50 Years and Above<br>ClinicalTrials.gov (2024)<br>NCT06374394	ClinicalTrials.gov registry report	Associated report<br>Associated registry record of NCT06374394.
000068	A PHASE 3, RANDOMIZED, DOUBLE-BLIND, PLACEBO-CONTROLLED STUDY TO EVALUATE THE SAFETY, TOLERABILITY, AND IMMUNOGENICITY OF RESPIRATORY SYNCYTIAL VIRUS PREFUSION F SUBUNIT VACCINE WHEN COADMINISTERED WITH SEASONAL INACTIVATED INFLUENZA VACCINE IN ADULTS ≥65 YEARS OF AGE<br>ClinicalTrials.gov (2022)<br>NCT05301322	ClinicalTrials.gov registry report	Excluded<br>Pfizer RSVpreF is a different RSV vaccine product from adjuvanted RSVPreF3 OA.

Record	Report and bibliographic identifier	Report type	Eligibility disposition
000071	A Phase 3 Randomized, Observer-Blind, Study to Evaluate Safety, Tolerability, and Immunogenicity of mRNA-1345, an mRNA Vaccine Targeting Respiratory Syncytial Virus (RSV), When Given Alone or Coadministered With a Seasonal Influenza Vaccine or SARS-CoV-2 Vaccine and When Given as an Open-label Boost at 1 Year Following a Primary Dose in Adults ≥ 50 Years of Age<br>ClinicalTrials.gov (2022)<br>NCT05330975	ClinicalTrials.gov registry report	Excluded<br>mRNA-1345 RSV vaccine, rather than RSVPreF3 OA; coadministration results are outside the current product scope.
000072	A Phase 3, Randomized, Observer-Blind Study to Evaluate Safety, Tolerability, and Immunogenicity of mRNA-1345, an mRNA Vaccine Targeting Respiratory Syncytial Virus, When Coadministered With a High-Dose, Quadrivalent Seasonal Influenza Vaccine in Adults ≥65 Years of Age<br>ClinicalTrials.gov (2023)<br>NCT06060457	ClinicalTrials.gov registry report	Excluded<br>mRNA-1345 RSV vaccine, rather than RSVPreF3 OA; coadministration results are outside the current product scope.
000081	A Phase I Double-Blind Placebo Controlled Trial to Evaluate the Safety and Immunogenicity of Intranasal Sendai Virus Vectored Respiratory Syncytial Virus (SeVRSV) Vaccine in Healthy Adults<br>ClinicalTrials.gov (2018)<br>NCT03473002	ClinicalTrials.gov registry report	Excluded<br>Intranasal Sendai-virus-vectored RSV vaccine, rather than RSVPreF3 OA.
000089	A Phase I Randomized, Observer-Blinded, Dose-Ranging Study to Evaluate the Immunogenicity and Safety of a Respiratory Syncytial Virus (RSV) Recombinant Fusion (F) Nanoparticle Vaccine, With or Without Aluminum Adjuvant, in Healthy Subjects 24 to <72 Months of Age<br>ClinicalTrials.gov (2014)<br>NCT02296463	ClinicalTrials.gov registry report	Excluded<br>RSV F protein nanoparticle vaccine, rather than adjuvanted RSVPreF3 OA.
000090	A Phase I Randomized, Observer-Blinded, Dose-Ranging Study to Evaluate the Immunogenicity and Safety of an RSV-F Protein Nanoparticle Vaccine, With or Without Aluminum Adjuvant, and Co-administered With a Licensed Inactivated Influenza Vaccine, in Healthy Subjects ≥ 60 Years of Age.<br>ClinicalTrials.gov (2012)<br>NCT01709019	ClinicalTrials.gov registry report	Excluded<br>RSV F protein nanoparticle vaccine, rather than adjuvanted RSVPreF3 OA.
000105	A Phase III, Open-label, Randomized, Controlled, Multi-country Study to Evaluate the Immune Response, Safety and Reactogenicity of an RSVPreF3 OA Investigational Vaccine When Co-administered With FLU aQIV (Inactivated Influenza Vaccine - Adjuvanted) in Adults Aged 65 Years and Above<br>ClinicalTrials.gov (2022)<br>NCT05568797	ClinicalTrials.gov registry report	Associated report<br>Associated registry record of NCT05568797.

Record	Report and bibliographic identifier	Report type	Eligibility disposition
000106	A Phase III, open-label, randomized, controlled, multi-country study to evaluate the immune response, safety, and reactogenicity of respiratory syncytial virus (RSV) prefusion protein 3 older adult (RSVPreF3 OA) investigational vaccine when co-administered with 20-Valent Pneumococcal Conjugate Vaccine (PCV20) in adults aged 60 Years and older<br>European trial registry (2023); 2022-501988-40-00<br>NCT05879107	European registry report	Associated report<br>Associated registry record of NCT05879107.
000107	A Phase III, Open-label, Randomized, Controlled, Multi-country Study to Evaluate the Immune Response, Safety and Reactogenicity of RSVPreF3 OA Investigational Vaccine When Co-administered With Herpes Zoster Recombinant Subunit (HZ/su) Vaccine in Adults Aged 50 Years and Older<br>ClinicalTrials.gov (2023)<br>NCT05966090	ClinicalTrials.gov registry report	Associated report<br>Associated registry record of NCT05966090.
000108	A Phase III, Open-label, Randomized, Controlled, Multi-Country Study to Evaluate the Immune Response, Safety and Reactogenicity of RSVPreF3 OA Investigational Vaccine When Co Administered With PCV20 in Adults Aged 60 Years and Older<br>ClinicalTrials.gov (2023)<br>NCT05879107	ClinicalTrials.gov registry report	Associated report<br>Associated registry record of NCT05879107.
000122	A Pragmatic Randomized Trial to Evaluate the Vaccine Effectiveness of Abrysvo® for Preventing RSV Hospitalizations in Adults Aged 18 Years or Above<br>ClinicalTrials.gov (2024)<br>NCT06684743	ClinicalTrials.gov registry report	Excluded<br>Abrysvo effectiveness trial comparing RSV vaccination with no RSV vaccination, outside the RSVPreF3 OA product scope.
000130	Langley, J.M. et al.<br>A Randomized, Controlled, Observer-Blinded Phase 1 Study of the Safety and Immunogenicity of a Respiratory Syncytial Virus Vaccine with or without Alum Adjuvant<br>Journal of Infectious Diseases (2017); pp. 24–33<br>DOI: 10.1093/infdis/jiw453<br>NCT01905215	Journal article	Excluded<br>First-generation RSV-PreF antigen at 10, 30 or 60 micrograms, with or without alum, in men aged 18–44 years; not RSVPreF3 OA.
000133	A Randomized, Double-blind, Placebo-controlled Phase 1/2 Clinical Trial to Evaluate the Safety, Tolerability, and Immunogenicity of Recombinant Respiratory Syncytial Virus Vaccine (SCTV02) in Healthy Adults ≥18 Years of Age<br>ClinicalTrials.gov (2024)<br>NCT06754605	ClinicalTrials.gov registry report	Excluded<br>Recombinant RSV vaccine SCTV02, rather than RSVPreF3 OA.

Record	Report and bibliographic identifier	Report type	Eligibility disposition
000137	A Randomized, Double-blind, Placebo-controlled Phase 2a Study to Evaluate the Safety and Immunogenicity of Seasonal Influenza Vaccine and Ad26.RSV.preF, With and Without Co-administration, in Adults Aged 60 Years and Older in Stable Health<br>ClinicalTrials.gov (2017)<br>NCT03339713	ClinicalTrials.gov registry report	Excluded<br>Ad26.RSV.preF, alone or combined with RSV preF protein, is outside the RSVPreF3 OA product scope.
000138	A Randomized, Double-blind, Placebo-controlled Phase 3 Study to Evaluate the Immunogenicity and Safety of Ad26.RSV.preF-based Vaccine and High-dose Seasonal Influenza Vaccine, With and Without Coadministration, in Adults Aged 65 Years and Older<br>ClinicalTrials.gov (2021)<br>NCT05071313	ClinicalTrials.gov registry report	Excluded<br>Ad26.RSV.preF, alone or combined with RSV preF protein, is outside the RSVPreF3 OA product scope.
000142	A Randomized, Double-Blind, Placebo Controlled Trial of North American Ginseng Extract (CVT-E002; COLD-fX®) to Prevent Respiratory Infection and Reduce Antibiotic Use in Patients With Chronic Lymphocytic Leukemia<br>ClinicalTrials.gov (2008)<br>NCT00752895	ClinicalTrials.gov registry report	Excluded<br>American ginseng extract versus placebo for prevention of respiratory illness; not an RSV vaccine intervention.
000146	A Randomized, Single-blind, Placebo Controlled, Dose-ranging Phase II Trial in ≥ 55 Year Old Adults to Evaluate the Safety and Immunogenicity of the Recombinant MVA-BN-RSV Vaccine<br>ClinicalTrials.gov (2016)<br>NCT02873286	ClinicalTrials.gov registry report	Excluded<br>MVA-BN-RSV is a viral-vector vaccine, outside the RSVPreF3 OA product scope.
000147	A Randomized, Single-blind, Placebo-controlled Phase I Trial to Evaluate the Safety, Tolerability and Immunogenicity of the Recombinant MVA BN® RSV Vaccine in Healthy Adult Subjects<br>ClinicalTrials.gov (2015)<br>NCT02419391	ClinicalTrials.gov registry report	Excluded<br>MVA-BN-RSV is a viral-vector vaccine, outside the RSVPreF3 OA product scope.
000153	Henry, C. et al.<br>A Seasonal Influenza Hemagglutinin mRNA-Based Vaccine Induces Humoral and Cellular Immunity Comparable to an Adjuvanted Inactivated Influenza Virus Vaccine<br>Open Forum Infectious Diseases (2026); pp. S925<br>DOI: 10.1093/ofid/ofaf695.1638<br>NCT05397223	Conference abstract	Excluded<br>mRNA-1010 versus FLUAD influenza vaccination; no RSVPreF3 OA intervention.
000163	A study on the immune response and safety of a vaccine against respiratory syncytial virus (RSV) when given alone and together with a vaccine against SARS-CoV-2 in adults aged 50 years and above<br>European trial registry (2024); 2023-510196-59-00<br>NCT06374394	European registry report	Associated report<br>Associated registry record of NCT06374394.

Record	Report and bibliographic identifier	Report type	Eligibility disposition
000164	A study on the immune response and safety of a vaccine against respiratory syncytial virus (RSV) when given alone or co-administered with an adjuvanted vaccine against influenza in adults aged 65 years and above<br>European trial registry (2022); 2022-000623-21-BE<br>NCT05568797	European registry report	Associated report<br>Associated registry record of NCT05568797.
000173	A STUDY TO EVALUATE THE SAFETY, TOLERABILITY, AND IMMUNOGENICITY OF COMBINED VACCINE CANDIDATE(S) AGAINST INFECTIOUS RESPIRATORY ILLNESSES, INCLUDING COVID-19 AND RSV, IN HEALTHY INDIVIDUALS<br>ClinicalTrials.gov (2023)<br>NCT05886777	ClinicalTrials.gov registry report	Excluded<br>Pfizer bivalent RSVpreF and BNT162b2 formulations, rather than RSVPreF3 OA.
000174	Maltezou, H.C. et al.<br>Absenteeism due to COVID-19, influenza and RSV among hospital-based healthcare personnel in Greece during the 2024–2025 season: A cohort study<br>Infection, Disease and Health (2026)<br>DOI: 10.1016/j.idh.2026.100448	Journal article	Excluded<br>Healthcare-personnel cohort measuring respiratory-virus-associated absenteeism; not a randomized vaccination-schedule comparison.
000180	Broad, Jonathan et al.<br>Adapting COVID-19 research infrastructure to capture influenza and respiratory syncytial virus alongside SARS-CoV-2 in UK healthcare workers winter 2022/23 and beyond: protocol for a pragmatic sub-study.<br>NIHR open research (2024)<br>DOI: 10.3310/nihropenres.13517.3	Journal article	Excluded<br>Protocol for an observational healthcare-worker surveillance cohort, rather than an RSVPreF3 OA coadministration trial.
000184	Najdowski, M. et al.<br>Adult Respiratory Syncytial Virus Vaccination Receipt among Adults Testing Negative for RSV - VISION Network, October 1, 2024-March 31, 2025<br>Open Forum Infectious Diseases (2026); pp. S958–S959<br>DOI: 10.1093/ofid/ofaf695.1701	Conference abstract	Excluded<br>VISION Network observational analysis of vaccination receipt among adults testing negative for RSV; no randomized schedule comparison.
000188	Kwetkat, A. et al.<br>After the COVID-19 pandemic—Which new vaccinations for adults are available or coming soon?<br>Innere Medizin (Germany) (2024); pp. 79–85<br>DOI: 10.1007/s00108-023-01640-9	Review	Excluded<br>Overview of adult vaccines and recommendations; not an original randomized trial report.

Record	Report and bibliographic identifier	Report type	Eligibility disposition
000191	Keus, J.A.R.<br>An accidental human trial of recombinant vaccinia virus: A step towards acceptance of live recombinant vaccines?<br>Trends in Biotechnology (1986); pp. 105–106<br>DOI: 10.1016/0167-7799(86)90133-2	Journal report	Excluded<br>Report concerns recombinant vaccinia virus, rather than the RSVPreF3 OA vaccination-schedule intervention.
000194	An Exploratory Pilot Study to Evaluate the Clinical Safety and Virologic Profile of an Experimental RSV Infection in Adults 60 to 75 Years of Age<br>ClinicalTrials.gov (2019)<br>NCT03919591	ClinicalTrials.gov registry report	Excluded<br>Experimental RSV infection challenge study; no partner-vaccine coadministration versus sequential comparison.
000195	An Observer-blind Safety and Reactogenicity Study to Assess GlaxoSmithKline (GSK) Biologicals' Investigational Respiratory Syncytial Virus (RSV) Vaccine (GSK3003891A) in Healthy Women<br>ClinicalTrials.gov (2016)<br>NCT02753413	ClinicalTrials.gov registry report	Excluded<br>GSK3003891A first-generation RSV vaccine formulation studies, rather than RSVPreF3 OA coadministration.
000196	An Observer-blind Study to Assess the Safety, Reactogenicity and Immunogenicity of Different Formulations of GSK Biologicals' Investigational RSV Vaccine (GSK3003891A), in Healthy Women<br>ClinicalTrials.gov (2015)<br>NCT02360475	ClinicalTrials.gov registry report	Excluded<br>GSK3003891A first-generation RSV vaccine formulation studies, rather than RSVPreF3 OA coadministration.
000198	An Observer-blind Study to Evaluate the Safety, Reactogenicity and Immunogenicity of GSK Biologicals' Respiratory Syncytial Virus (RSV) Investigational Vaccine (GSK3003891A) in Healthy Men<br>ClinicalTrials.gov (2013)<br>NCT01905215	ClinicalTrials.gov registry report	Excluded<br>GSK3003891A first-generation RSV vaccine formulation studies, rather than RSVPreF3 OA coadministration.
000208	Karaba, A.H. et al.<br>Antibody Response to Respiratory Syncytial Virus Vaccination in Immunocompromised Persons<br>Jama (2025); pp. 429–432<br>DOI: 10.1001/jama.2024.25395	Research letter reporting a cohort	Excluded<br>Prospective observational cohort of immunocompromised vaccine recipients with paired antibody samples; vaccination schedules were not randomized.
000210a	Aung, G. et al.<br>AS01E-ADJUVANTED RSV PREFUSION F PROTEIN VACCINE REDUCES RSV ACUTE RESPIRATORY ILLNESS-RELATED COMPLICATIONS AND MEDICATION USE IN PARTICIPANTS WITH COPD/ASTHMA<br>Internal Medicine Journal (2026); pp. 178<br>DOI: 10.1111/imj.70364<br>NCT04886596	Conference abstract	Excluded<br>Post-hoc RSVPreF3 efficacy analysis in COPD or asthma; vaccine versus placebo, without a partner-vaccine schedule comparison.

Record	Report and bibliographic identifier	Report type	Eligibility disposition
000210b	Aung, G. et al.<br>IMMUNOGENICITY AND SAFETY OF AS01E-ADJUVANTED RSVPREF3 VACCINE IN ADULTS 18–49 AT RISK AND ≥60 YOA THROUGH 6 MONTHS<br>Internal Medicine Journal (2026); pp. 183<br>DOI: 10.1111/imj.70364<br>NCT06389487	Conference abstract	Excluded<br>Age-based immunobridging after one RSVPreF3 dose; no randomized partner-vaccine schedule comparison.
000211	McDonald, J.U. et al.<br>Assessing commutability of the first WHO International Standard for antiserum to respiratory syncytial virus<br>Vaccine (2025)<br>DOI: 10.1016/j.vaccine.2025.127430	Journal article	Excluded<br>Commutability study of a WHO antiserum standard for RSV assays; not a clinical vaccination-schedule comparison.
000217	Simmons, A.E. et al.<br>Association Between Seasonal Respiratory Virus Activity and Invasive Pneumococcal Disease in Central Ontario, Canada<br>medRxiv (2024)<br>DOI: 10.1101/2024.09.03.24312990	Preprint	Excluded<br>Observational analysis of respiratory-virus activity and invasive pneumococcal disease in Ontario; not a vaccine trial.
000218	Simmons, A.E. et al.<br>Association between seasonal respiratory virus activity and invasive pneumococcal disease in central Ontario, Canada<br>BMC Infectious Diseases (2026)<br>DOI: 10.1186/s12879-025-12419-8	Journal article	Excluded<br>Observational analysis of respiratory-virus activity and invasive pneumococcal disease in Ontario; not a vaccine trial.
000221	Karaba, A.H. et al.<br>Attenuated PreFusion F Antibody Response to RSV Vaccines in Solid Organ Transplant Recipients<br>Open Forum Infectious Diseases (2025); pp. S49–S50<br>DOI: 10.1093/ofid/ofae631.076	Conference abstract	Excluded<br>Prospective antibody-response cohort of solid organ transplant recipients; no randomized coadministration versus sequential comparison.
000223	Golmakani, E. et al.<br>Bacterial and Viral Respiratory Co-infections With SARS-CoV-2 in Deceased Patients of North Khorasan, Iran, During 2020-2023<br>Avicenna Journal of Clinical Microbiology and Infection (2026); pp. 66–71<br>DOI: 10.34172/ajcmi.3719	Journal article	Excluded<br>Observational comparison of bacterial and viral coinfections in SARS-CoV-2-positive patients; not a vaccination-schedule trial.

Record	Report and bibliographic identifier	Report type	Eligibility disposition
000224	Lingblom, C.M.D. et al.<br>Baseline immune profile by CyTOF can predict response to an investigational adjuvanted vaccine in elderly adults<br>Journal of Translational Medicine (2018); pp. 153<br>DOI: 10.1186/s12967-018-1528-1<br>NCT02115815	Journal article	Excluded<br>CyTOF analysis of vaccine responders and nonresponders from a phase 1a RSV study; no partner-vaccine schedule comparison.
000233	Glanternik, J. et al.<br>Bivalent RSVpreF Subunit Vaccine Safety and Immunogenicity in Seropositive 2–<18 Year Olds<br>Vaccines (2026)<br>DOI: 10.3390/vaccines14020128<br>NCT05900154	Journal article	Excluded<br>Bivalent RSVpreF study in children aged 2 to under 18 years; outside the product and population scope.
000247	Pershagen, G.<br>Can immunization affect the development of allergy?<br>Pediatric Allergy and Immunology, Supplement (2000); pp. 26–28<br>DOI: 10.1034/j.1399-3038.2000.00507.x	Review	Excluded<br>Review of immunization and allergic disease; not an original eligible randomized comparison.
000263	Pacheco, G.A. et al.<br>Characterization of the humoral and cellular immunity induced by a recombinant BCG vaccine for the respiratory syncytial virus in healthy adults<br>Frontiers in Immunology (2023)<br>DOI: 10.3389/fimmu.2023.1215893<br>NCT03213405	Journal article	Excluded<br>Recombinant BCG RSV vaccine, rather than RSVPreF3 OA.
000274	Venkatesan, P<br>Co-administered RSV and pneumococcal disease vaccines in older adults<br>Lancet Infectious Diseases (2026); pp. e216–e216<br>DOI: 10.1016/s1473-3099(26)00248-3	Research news	Excluded<br>Research news item. Author, journal, volume, issue and page identify the parent Research in brief report; it is not an original randomized trial report.
000275	Mangan, N et al.<br>CO-ADMINISTRATION OF ADJUVANTED RESPIRATORY SYNCYTIAL VIRUS PREFUSION F PROTEIN VACCINE WITH ADJUVANTED RECOMBINANT ZOSTER VACCINE IN ADULTS AGED ≥50 YEARS<br>Internal medicine journal (2025); TO148, pp. 67–68<br>DOI: 10.1111/imj.70003<br>NCT05966090	Conference abstract	Associated report<br>Associated conference report of NCT05966090.

Record	Report and bibliographic identifier	Report type	Eligibility disposition
000276	Dennis, P et al.<br>Co-administration of the adjuvanted respiratory syncytial virus (RSV) prefusion F protein vaccine (RSVPreF3 OA) with the adjuvanted recombinant zoster vaccine (RZV) in adults 50 years of age<br>European geriatric medicine (2024); p. S410<br>DOI: 10.1007/s41999-024-01090-y<br>NCT05966090	Conference abstract	Associated report<br>Associated conference report of NCT05966090.
000277	RSV-020 study grp<br>Co-administration of the adjuvanted respiratory syncytial virus (RSV) prefusion F protein vaccine (RSVPreF3 OA) with the adjuvanted recombinant zoster vaccine (RZV) in adults ≥50years of age<br>Australasian Journal On Ageing (2025); 44(S1):e70037; subsection beginning Co-administration of the adjuvanted respiratory syncytial virus<br>DOI: 10.1111/ajag.70037<br>NCT05966090	Encore conference abstract	Associated report<br>Associated conference report of NCT05966090.
000283	Baber, JA et al.<br>Coadministration of Bivalent Respiratory Syncytial Virus (RSVpreF) Vaccine With Influenza Vaccine in Older Adults<br>Open forum infectious diseases (2023); pp. S58–S59<br>DOI: 10.1093/ofid/ofad500.095<br>NCT05301322	Conference abstract	Excluded<br>Pfizer bivalent RSVpreF vaccine, rather than adjuvanted RSVPreF3 OA.
000284	Comstock, E. et al.<br>Coadministration of mRNA-1345 RSV vaccine with high-dose quadrivalent influenza vaccine in adults aged 65 and older: An observer-blinded, placebo-controlled, randomized, phase 3 trial<br>Human Vaccines and Immunotherapeutics (2026); pp. 2649335<br>DOI: 10.1080/21645515.2026.2649335<br>NCT06060457	Journal article	Excluded<br>mRNA-1345 RSV vaccine, rather than RSVPreF3 OA; coadministration results are outside the current product scope.
000285	Dacso, C.C.<br>Coadministration of RSV + influenza or COVID-19 vaccines was noninferior to separate administration for immune responses in adults aged ≥50 y<br>Annals of Internal Medicine (2025); pp. JC41<br>DOI: 10.7326/annals-25-00904-jc	ACP Journal Club summary	Excluded<br>ACP Journal Club evidence summary of a published coadministration trial; no independent trial dataset.

Record	Report and bibliographic identifier	Report type	Eligibility disposition
000296	Ackerson, B.K. et al.<br>Comparison of Hospitalization Costs Associated With Human Metapneumovirus and Respiratory Syncytial Virus Infection in US Adults<br>Open Forum Infectious Diseases (2026)<br>DOI: 10.1093/ofid/ofag202	Journal article	Excluded<br>Observational comparison of hospitalization costs for RSV and human metapneumovirus infections; not a vaccination-schedule trial.
000301	Kachikis, A. et al.<br>Comparison of Respiratory Syncytial Virus (RSV)-Specific Antibody Durability in Pregnant/Postpartum Individuals and Older Adults After RSV Vaccination<br>The Journal of infectious diseases (2026)<br>DOI: 10.1093/infdis/jiag111	Journal article	Excluded<br>Prospective comparison of antibody durability in pregnant or postpartum individuals and older adults; no randomized schedule comparison.
000302	Kachikis, A.B. et al.<br>Comparison of Respiratory Syncytial Virus-Specific Antibody Durability in Pregnant/Postpartum Individuals and Older Adults After RSV Vaccination<br>Open Forum Infectious Diseases (2026); pp. S903–S904<br>DOI: 10.1093/ofid/ofaf695.1606	Conference abstract	Excluded<br>Prospective comparison of antibody durability in pregnant or postpartum individuals and older adults; no randomized schedule comparison.
000308	Falsey, A.R. et al.<br>Comparison of the safety and immunogenicity of 2 Respiratory Syncytial Virus (RSV) vaccines - Nonadjuvanted vaccine or vaccine adjuvanted with alum - Given concomitantly with influenza vaccine to high-risk elderly individuals<br>Journal of Infectious Diseases (2008); pp. 1317–1326<br>DOI: 10.1086/592168	Journal article	Excluded<br>Older nonadjuvanted or alum-adjuvanted RSV formulations with trivalent influenza vaccine, rather than RSVPreF3 OA; controls received influenza vaccine with placebo.
000310	Kautz, M. et al.<br>Compliance and Performance Characteristics of Subject Collected Versus Health-care Worker Collected Nasal Swabs for Respiratory Viral Surveillance<br>Open Forum Infectious Diseases (2021); pp. S440–S441<br>DOI: 10.1093/ofid/ofab466.874	Conference abstract	Excluded<br>Evaluation of participant-collected versus healthcare-worker-collected nasal swabs, rather than vaccination schedules.
000315	Widagdo, W et al.<br>Concomitant Administration of Ad26.RSV.preF/RSV preF Protein Vaccine and High-Dose Influenza Vaccine in Adults 65 Years and Older: A Noninferiority Trial<br>Journal Of Infectious Diseases (2024); pp. e374–e383<br>DOI: 10.1093/infdis/jiad594<br>NCT05071313	Journal article	Excluded<br>Ad26.RSV.preF, alone or combined with RSV preF protein, is outside the RSVPreF3 OA product scope.

Record	Report and bibliographic identifier	Report type	Eligibility disposition
000319	Dayananda, P. et al.<br>Controlled Human Infection Challenge Studies with RSV<br>Current Topics in Microbiology and Immunology (2024); pp. 41–68<br>DOI: 10.1007/82_2022_257	Book chapter	Excluded<br>Book chapter reviewing controlled human RSV infection studies; not an individual eligible coadministration trial report.
000357	CSP #2038 - COVID-19 Pharmacotherapy Effectiveness in the VA Healthcare System (COPE-VA)<br>ClinicalTrials.gov (2023)<br>NCT06160128	ClinicalTrials.gov registry report	Excluded<br>Observational evaluation of COVID-19 pharmacotherapy in the VA healthcare system; not an RSV vaccine-schedule trial.
000358	Neuzil, K.M.<br>Current and Emerging Approaches to Evaluating Influenza Vaccine Performance<br>New England Journal of Medicine (2025); pp. 2361–2362<br>DOI: 10.1056/nejme2513191	Editorial	Excluded<br>Editorial on evaluation of influenza vaccine performance; not an original RSV coadministration trial report.
000363	Nyiro, J.U. et al.<br>Defining the vaccination window for Respiratory syncytial virus (RSV) using ageseroprevalence data for children in Kilifi, Kenya<br>PLoS ONE (2017)<br>DOI: 10.1371/journal.pone.0177803	Journal article	Excluded<br>Age-seroprevalence study in children, rather than a randomized vaccination-schedule study.
000367	Watt, P.J. et al.<br>Determinants of susceptibility to challenge and the antibody response of adult volunteers given experimental respiratory syncytial virus vaccines<br>Vaccine (1990); pp. 231–236<br>DOI: 10.1016/0264-410x(90)90051-m	Journal article	Excluded<br>Experimental temperature-sensitive live RSV vaccines and challenge, rather than RSVPreF3 OA.
000369	Itell, H.L. et al.<br>Development and application of a multiplex assay for the simultaneous measurement of antibody responses elicited by common childhood vaccines<br>Vaccine (2018); pp. 5600–5608<br>DOI: 10.1016/j.vaccine.2018.07.048	Journal article	Excluded<br>Multiplex assay development for antibody responses to childhood vaccines; not a clinical RSV coadministration trial.
000370	Anderson, L.J. et al.<br>Development and comparison of immunologic assays to detect primary RSV infections in infants<br>Frontiers in Immunology (2023)<br>DOI: 10.3389/fimmu.2023.1332772	Journal article	Excluded<br>Development and comparison of assays for primary RSV infection in infants; not a vaccination-schedule trial.

Record	Report and bibliographic identifier	Report type	Eligibility disposition
000372	Schepp, RM et al.<br>Development and Standardization of a High-Throughput Multiplex Immunoassay for the Simultaneous Quantification of Specific Antibodies to Five Respiratory Syncytial Virus Proteins<br>Msphere (2019)<br>DOI: 10.1128/msphere.00236-19	Journal article	Excluded<br>Development and standardization of a respiratory-antibody multiplex immunoassay; not an eligible clinical trial.
000376	Kumari, S. et al.<br>Development of a luciferase immunoprecipitation system assay to detect IgG antibodies against human respiratory syncytial virus nucleoprotein<br>Clinical and Vaccine Immunology (2014); pp. 383–390<br>DOI: 10.1128/cvi.00594-13	Journal article	Excluded<br>Development of an RSV nucleoprotein antibody assay; not an eligible clinical vaccination-schedule comparison.
000417	Effect of Montelukast on Experimentally-Induced RV16 Infection in Volunteers With Mild Asthma<br>ClinicalTrials.gov (2006)<br>NCT00359073	ClinicalTrials.gov registry report	Excluded<br>Montelukast in experimentally induced rhinovirus infection; not an RSV vaccine intervention.
000419	Skaarup, K.G. et al.<br>Effect of RSV Vaccine on Heart Failure Hospitalizations: A Prespecified Analysis of the DAN-RSV Trial<br>Journal of the American College of Cardiology (2025); pp. 2062–2067<br>DOI: 10.1016/j.jacc.2025.08.023	Journal article	Excluded<br>DAN-RSV subgroup report evaluates bivalent RSVpreF versus no vaccination, outside the RSVPreF3 OA product scope.
000449	Efficacy of bivalent prefusion F vaccine during pregnancy in preventing respiratory syncytial virus-related diseases in infants: a phase III, double-blind, randomized controlled trial<br>Chinese Journal of Perinatal Medicine (2023); pp. 495<br>DOI: 10.3760/cma.j.issn.1007-9408.2023.06.104	Secondary research summary	Excluded<br>Indexed as a note summarizing a maternal bivalent RSV vaccine efficacy trial with infant outcomes; not an original RSVPreF3 OA schedule trial report.
000459	Phung, E. et al.<br>Elicitation of pneumovirus-specific B cell responses by a prefusion-stabilized respiratory syncytial virus F subunit vaccine<br>Science Translational Medicine (2022)<br>DOI: 10.1126/scitranslmed.abo5032<br>NCT03049488	Journal article	Excluded<br>Prefusion-stabilized DS-Cav1 vaccine immunology, rather than the RSVPreF3 OA product.

Record	Report and bibliographic identifier	Report type	Eligibility disposition
000495	Wright, P.F. et al.<br>Evaluation of a live, cold-passaged, temperature-sensitive, respiratory syncytial virus vaccine candidate in infancy<br>Journal of Infectious Diseases (2000); pp. 1331–1342<br>DOI: 10.1086/315859	Journal article	Excluded<br>Live-attenuated RSV vaccination in infants or children; outside the product and population scope.
000501	Phelps, K. et al.<br>Evaluation of immunological parameters for correlates of protection against live siv challenge in recombinant simian varicella virus-simian immunodeficiency virus (RSVV-SIV) vaccinated nonhuman primates<br>Journal of Medical Primatology (2011); pp. 268–269<br>DOI: 10.1111/j.1600-0684.2011.00491.x	Conference abstract	Excluded<br>Recombinant simian varicella/SIV vaccination and SIV challenge in nonhuman primates.
000503	Gonzalez, IM et al.<br>Evaluation of the live attenuated cpts 248/404 RSV vaccine in combination with a subunit RSV vaccine (PFP-2) in healthy young and older adults<br>Vaccine (2000); pp. 1763–1772<br>DOI: 10.1016/s0264-410x(99)00527-7	Journal article	Excluded<br>Combination of live cpts248/404 RSV vaccine and PFP-2 subunit RSV vaccine; not RSVPreF3 OA with a partner vaccine.
000513	Richard, S.A.<br>Exploring the Pivotal Immunomodulatory and Anti-Inflammatory Potentials of Glycyrrhizic and Glycyrrhetinic Acids<br>Mediators of Inflammation (2021)<br>DOI: 10.1155/2021/6699560	Review	Excluded<br>Review of glycyrrhizic and glycyrrhetinic acids; not an original eligible RSV vaccine trial.
000514	Huynh, K et al.<br>Exploring the "Two-Hit" Phenomenon of Guillain-Barré Syndrome Following Respiratory Syncytial Virus and Influenza Vaccinations<br>Cureus Journal Of Medical Science (2024)<br>DOI: 10.7759/cureus.68899	Journal article	Excluded<br>Individual Guillain–Barré syndrome case report after RSV and influenza vaccination; no randomized comparison.
000525	Van Der Plas, J. et al.<br>First-in-human, randomized study to assess the safety, tolerability, immunogenicity, and shedding of a liveattenuated respiratory syncytial virus vaccine<br>British Journal of Clinical Pharmacology (2020); pp. 1189–1190<br>DOI: 10.1111/bcp.14266	Conference abstract	Excluded<br>Live-attenuated RSV vaccine study, rather than RSVPreF3 OA.

Record	Report and bibliographic identifier	Report type	Eligibility disposition
000538	Hause, A.M. et al.<br>Health events reported by US adults aged ≥60 years who received a respiratory syncytial virus vaccine alone or with other vaccines<br>Ssrn (2026)<br>DOI: 10.2139/ssrn.6556803	Preprint	Excluded<br>Voluntary v-safe survey of self-reported health events; observational exposure groups rather than randomized vaccine schedules.
000544	Vera-Punzano, N. et al.<br>Hospitalisation due to respiratory syncytial virus in a population-based cohort of older adults in Spain, 2016/17 to 2019/20<br>Eurosurveillance (2025)<br>DOI: 10.2807/1560-7917.es.2025.30.10.2400364	Journal article	Excluded<br>Population-based cohort of RSV hospitalization in Spain; not a randomized vaccination-schedule trial.
000552	Burton, D.R. et al.<br>Human antibodies from combinatorial libraries<br>Advances in Immunology (1994); pp. 191–280<br>DOI: 10.1016/s0065-2776(08)60674-4	Review	Excluded<br>Review concerning combinatorial antibody libraries; not an original vaccine-schedule trial.
000554	Ulloa-Aguilar, J.M. et al.<br>Human respiratory syncytial virus regulates the expression of interferon-stimulated genes through modulation of fibrillarin<br>Frontiers in Cellular and Infection Microbiology (2026)<br>DOI: 10.3389/fcimb.2026.1706028	Journal article	Excluded<br>Laboratory investigation of fibrillarin and interferon-stimulated genes during RSV infection.
000555	Jozwik, A. et al.<br>Human T-cell responses to RSV infection in the lower airway<br>Immunology (2014); pp. 76<br>DOI: 10.1111/imm.12406	Conference abstract	Excluded<br>Experimental RSV infection and lower-airway T-cell responses; no partner-vaccine schedule comparison.
000569	Ferguson, M. et al.<br>Immune persistence and safety of the AS01E-adjuvanted respiratory syncytial virus prefusion F protein-based vaccine in adults 50–59 years of age, including at-increased-risk adults: A randomized controlled trial<br>Human Vaccines and Immunotherapeutics (2025)<br>DOI: 10.1080/21645515.2025.2579335<br>NCT05590403	Journal article	Excluded<br>RSVPreF3 OA immune persistence and age-based immunobridging study; no partner-vaccine coadministration versus sequential comparison.
000571	Murphy, B.R. et al.<br>Immunization of cotton rats with the fusion (F) and large (G) glycoproteins of respiratory syncytial virus (RSV) protects against RSV challenge without potentiating RSV disease<br>Vaccine (1989); pp. 533–540<br>DOI: 10.1016/0264-410x(89)90278-8	Journal article	Excluded<br>Cotton-rat immunization with RSV F and G glycoproteins and viral challenge.

Record	Report and bibliographic identifier	Report type	Eligibility disposition
000575	Davis, M et al.<br>Immunobridging Demonstrating Effectiveness of the Bivalent Respiratory Syncytial Virus (RSV) Prefusion F Subunit Vaccine in Adults 18-59 Years of Age at High Risk of Severe RSV Disease in a Phase 3 Trial: the C3671023 MONeT Study Results<br>Open forum infectious diseases (2025); pp. S462–S463<br>DOI: 10.1093/ofid/ofae631.794<br>NCT05842967	Conference abstract	Excluded<br>Pfizer bivalent RSVpreF immunobridging in adults aged 18–59 years; outside the RSVPreF3 OA product scope.
000577	Pringle, C.R. et al.<br>Immunogenciity and pathogenicity of a triple temperature-sensitive modified respiratory syncytial virus in adult volunteers<br>Vaccine (1993); pp. 473–478<br>DOI: 10.1016/0264-410x(93)90290-e	Journal article	Excluded<br>Experimental temperature-sensitive live RSV vaccines and challenge, rather than RSVPreF3 OA.
000579	McKay, E. et al.<br>Immunogenicity and pathogenicity of temperature-sensitive modified respiratory syncytial virus in adult volunteers<br>Journal of Medical Virology (1988); pp. 411–421<br>DOI: 10.1002/jmv.1890250405	Journal article	Excluded<br>Experimental temperature-sensitive live RSV vaccines and challenge, rather than RSVPreF3 OA.
000580	Hannaford, A. et al.<br>Immunogenicity and Reactogenicity of Adjuvanted (Arexvy) and Non-Adjuvanted (Abrysvo) RSV Vaccines in Immunocompromised Individuals with Hematological Malignancies<br>Open Forum Infectious Diseases (2026); pp. S905–S906<br>DOI: 10.1093/ofid/ofaf695.1609	Conference abstract	Excluded<br>Immunocompromised-patient and healthy-control vaccine-response cohorts; no randomized coadministration versus sequential comparison.
000583	Fries, L. et al.<br>Immunogenicity and safety of a respiratory syncytial virus fusion protein (RSV F) nanoparticle vaccine in older adults<br>Immunity and Ageing (2017)<br>DOI: 10.1186/s12979-017-0090-7<br>NCT01709019	Journal article	Excluded<br>RSV F protein nanoparticle vaccine, rather than adjuvanted RSVPreF3 OA.
000584	Hosman, T. et al.<br>Immunogenicity and safety of Ad26.RSV.preF/RSV preF protein vaccine at predicted intermediate- and end-of-shelf-life as an evaluation of potency throughout shelf life<br>Human Vaccines and Immunotherapeutics (2024)<br>DOI: 10.1080/21645515.2024.2344970<br>NCT05101486	Journal article	Excluded<br>Ad26.RSV.preF, alone or combined with RSV preF protein, is outside the RSVPreF3 OA product scope.

Record	Report and bibliographic identifier	Report type	Eligibility disposition
000586	Leroux-Roels, I. et al.<br>Immunogenicity and Safety of Co-administration of Adjuvanted Respiratory Syncytial Virus Prefusion F Protein Vaccine With 20-Valent Pneumococcal Conjugate Vaccine in Adults Aged ≥60 Years: A Randomized, Non-inferiority Trial<br>Clinical Infectious Diseases (2026)<br>DOI: 10.1093/cid/ciag250<br>NCT05879107	Journal article	Primary trial article<br>Eligible RSVPreF3 OA randomized coadministration versus sequential trial; primary article for outcome extraction.
000587	Bonten, M et al.<br>Immunogenicity and Safety of Coadministration of AS01E-Adjuvanted Respiratory Syncytial Virus Prefusion F Protein Vaccine and a Coronavirus Disease 2019 mRNA Vaccine in Adults Aged ≥50 Years: A Phase 3, Randomized, Noninferiority Trial<br>Clinical Infectious Diseases (2026)<br>DOI: 10.1093/cid/ciag344<br>NCT06374394	Journal article	Primary trial article<br>Eligible RSVPreF3 OA randomized coadministration versus sequential trial; primary article for outcome extraction.
000589	Immunogenicity and Safety of Multiple-Dose Adjuvanted RSVPreF3 (Arexvy®) Vaccination Among Immunocompromised Persons<br>ClinicalTrials.gov (2025)<br>NCT07050732	ClinicalTrials.gov registry report	Excluded<br>One-dose versus two-dose Arexvy vaccination and boosting in immunocompromised persons; no partner-vaccine schedule comparison.
000591	Roussy, J.-F. et al.<br>Immunogenicity and Safety of the Adjuvanted Respiratory Syncytial Virus Prefusion F Protein-Based Vaccine When Co-administered With the Adjuvanted Recombinant Herpes Zoster Subunit Vaccine in Adults ≥ 50 Years of Age<br>Clinical Infectious Diseases (2026)<br>DOI: 10.1093/cid/ciag208<br>NCT05966090	Journal article	Primary trial article<br>Eligible RSVPreF3 OA randomized coadministration versus sequential trial; primary article for outcome extraction.
000597	Chandler, R et al.<br>Immunogenicity, reactogenicity and safety of a Respiratory Syncytial Virus prefusion F (RSVPreF3) candidate vaccine co-administered with the seasonal quadrivalent influenza vaccine in older adults<br>Open forum infectious diseases (2022); pp. S864–S865<br>DOI: 10.1093/ofid/ofac492.1759<br>NCT04841577	Conference abstract	Associated report<br>Associated conference report of NCT04841577.

Record	Report and bibliographic identifier	Report type	Eligibility disposition
000598	Chandler, R. et al.<br>Immunogenicity, Reactogenicity, and Safety of AS01E-adjuvanted Respiratory Syncytial Virus (RSV) Prefusion F Protein-based Candidate Vaccine (RSVPreF3 OA) When Co-administered With a Seasonal Quadrivalent Influenza Vaccine in Older Adults: Results of a Phase 3, Open-Label, Randomized Controlled Trial<br>Clinical Infectious Diseases (2026); pp. e62–e70<br>DOI: 10.1093/cid/ciad786<br>NCT04841577	Journal article	Primary trial article<br>Eligible RSVPreF3 OA randomized coadministration versus sequential trial; primary article for outcome extraction.
000629	Suresh, G. et al.<br>IMpact-RSV study group report [1] (multiple letters)<br>Pediatrics (1999); pp. 993–995<br>DOI: 10.1542/peds.104.4.993	Letters and comments	Excluded<br>Letters commenting on the IMpact-RSV palivizumab study; not an original RSVPreF3 OA trial report.
000630	Awar, M. et al.<br>In older adults, an AS01E-adjuvanted RSVPreF3 OA vaccine reduced RSV-related lower respiratory tract disease<br>Annals of Internal Medicine (2023); pp. JC62–JC63<br>DOI: 10.7326/j23-0038	ACP Journal Club summary	Excluded<br>ACP Journal Club evidence summary of RSV vaccine effectiveness; not an original vaccination-schedule trial report.
000631	Sacks, H.S.<br>In older adults, RSV prefusion F vaccine reduced hospitalization for RSV-related respiratory tract disease vs. no vaccine<br>Annals of Internal Medicine (2026); pp. JC4<br>DOI: 10.7326/annals-25-05113-jc	ACP Journal Club summary	Excluded<br>ACP Journal Club evidence summary of RSV vaccine effectiveness; not an original vaccination-schedule trial report.
000645	Gallwitz, B. et al.<br>Indication-based vaccinations for people with diabetes mellitus<br>Diabetologie (2025); pp. 758–767<br>DOI: 10.1007/s11428-025-01379-x	Recommendations	Excluded<br>Vaccination recommendations for people with diabetes; not an original randomized trial report.
000646	Covián, C. et al.<br>Induction of Trained Immunity by Recombinant Vaccines<br>Frontiers in Immunology (2021)<br>DOI: 10.3389/fimmu.2020.611946	Review	Excluded<br>Review of recombinant vaccines and trained immunity; not an original eligible schedule trial.
000658	Woźniak-Kosek, A. et al.<br>[Influenza in Poland in 2010].<br>Przegląd epidemiologiczny (2012); pp. 599–604	Journal article	Excluded<br>National influenza surveillance report for Poland; not an RSV vaccination-schedule trial.

Record	Report and bibliographic identifier	Report type	Eligibility disposition
000663	Mihălțan, F.-D. et al.<br>Innovative approaches in RSV prevention: The expanding role of monoclonal antibodies in protection for all infants<br>Germs (2025); pp. 279–282<br>DOI: 10.18683/germs.2025.1475	Review	Excluded<br>Overview of monoclonal-antibody prevention in infants; not an original RSVPreF3 OA trial report.
000680	Cunningham, C.K. et al.<br>Live-attenuated respiratory syncytial virus vaccine with deletion of RNA synthesis regulatory protein M2-2 and cold passage mutations is overattenuated<br>Open Forum Infectious Diseases (2019)<br>DOI: 10.1093/ofid/ofz212<br>NCT02890381; NCT02948127	Journal article	Excluded<br>Live-attenuated RSV vaccination in infants or children; outside the product and population scope.
000684	Ascough, S. et al.<br>Local and Systemic Immunity against Respiratory Syncytial Virus Induced by a Novel Intranasal Vaccine A Randomized, Double-Blind, Placebo-controlled Clinical Trial<br>American Journal of Respiratory and Critical Care Medicine (2019); pp. 481–492<br>DOI: 10.1164/rccm.201810-1921oc<br>NCT02958540	Journal article	Excluded<br>Intranasal SynGEM vaccine, rather than RSVPreF3 OA.
000691	Omelyanovskiy, V.V. et al.<br>Main changes in the model of diagnosis-related groups in 2022<br>Medical Technologies. Assessment and Choice (2022); pp. 8–21<br>DOI: 10.17116/medtech2022440218	Policy and reimbursement discussion	Excluded<br>Discussion of changes to diagnosis-related group reimbursement models; not an original vaccine trial.
000693	Mills, J.<br>Management of respiratory syncytial virus infections<br>Advances in Experimental Medicine and Biology (1996); pp. 163–174<br>DOI: 10.1007/978-1-4757-9209-6_17	Review and conference paper	Excluded<br>Review of RSV infection management, indexed as a conference paper; not an original eligible trial report.
000713	Gagneux-Brunon, A. et al.<br>Midwives' attitudes toward participation of pregnant individuals in a preventive vaccine hypothetical clinical trial<br>Vaccine (2022); pp. 5459–5463<br>DOI: 10.1016/j.vaccine.2022.07.049	Journal article	Excluded<br>Survey of midwives about a hypothetical maternal vaccine trial; no trial participants randomized to vaccination schedules.

Record	Report and bibliographic identifier	Report type	Eligibility disposition
000722	Sun, Y. et al.<br>mRNA-Based Respiratory Syncytial Virus Vaccine mRNA-1345 Induces Robust, Polyfunctional, and Durable CD4+ and CD8+ T Cell Responses with Similar Features in Adults Across Age Groups<br>Open Forum Infectious Diseases (2026); pp. S926<br>DOI: 10.1093/ofid/ofaf695.1640<br>NCT05397223	Conference abstract	Excluded<br>mRNA-1345 RSV vaccine study, rather than RSVPreF3 OA.
000747	Eyles, J.E. et al.<br>Nonreplicating vaccines can protect african green monkeys from the memphis 37 strain of respiratory syncytial virus<br>Journal of Infectious Diseases (2013); pp. 319–329<br>DOI: 10.1093/infdis/jit169	Journal article	Excluded<br>RSV vaccination and challenge in African green monkeys.
000748	Green, Christopher A et al.<br>Novel genetically-modified chimpanzee adenovirus and MVA-vectored respiratory syncytial virus vaccine safely boosts humoral and cellular immunity in healthy older adults.<br>The Journal of infection (2019); pp. 382–392<br>DOI: 10.1016/j.jinf.2019.02.003	Journal article	Excluded<br>PanAd3-RSV and MVA-RSV vectored vaccination, rather than RSVPreF3 OA.
000750	Covert, K. et al.<br>Novel Maintenance Therapies for Chronic Obstructive Pulmonary Disorder<br>Annals of Pharmacotherapy (2025)<br>DOI: 10.1177/10600280251382202	Review	Excluded<br>Review of COPD maintenance therapies and recommendations; not an original coadministration trial.
000753	Open-label, Multicenter Trial of RSV Vaccination to Reduce Moderate-to-severe Exacerbations in COPD Frequent Exacerbators: Clinical Effectiveness and RSV-specific Immune Responses<br>ClinicalTrials.gov (2026)<br>NCT07508644	ClinicalTrials.gov registry report	Excluded<br>RSV vaccination plus standard care versus standard care in COPD; no assigned same-pair sequential vaccine comparator.
000754	Open Label, Single Arm Phase 1 Study to Evaluate the Shedding, Biodistribution, Safety and Immunogenicity of Ad26.RSV.preF Vaccine in Adults<br>ClinicalTrials.gov (2019)<br>NCT03795441	ClinicalTrials.gov registry report	Excluded<br>Ad26.RSV.preF, alone or combined with RSV preF protein, is outside the RSVPreF3 OA product scope.

Record	Report and bibliographic identifier	Report type	Eligibility disposition
000782	Schmoele-Thoma, B et al.<br>Phase 1/2, first-in-human study of the safety, tolerability, and immunogenicity of an RSV prefusion F-based subunit vaccine candidate<br>Open forum infectious diseases (2019); pp. S970<br>DOI: 10.1093/ofid/ofz360.2432<br>NCT03529773	Conference abstract	Excluded<br>Pfizer bivalent RSVpreF vaccine, rather than adjuvanted RSVPreF3 OA.
000783	Falsey, A.R. et al.<br>Phase 1/2 Randomized Study of the Immunogenicity, Safety, and Tolerability of a Respiratory Syncytial Virus Prefusion F Vaccine in Adults with Concomitant Inactivated Influenza Vaccine<br>Journal of Infectious Diseases (2022); pp. 2056–2066<br>DOI: 10.1093/infdis/jiab611<br>NCT03529773	Journal article	Excluded<br>Pfizer bivalent RSVpreF vaccine, rather than adjuvanted RSVPreF3 OA.
000784	Phase 1, Open-Label, Dose-Escalation Study to Evaluate Tolerability, Safety, and Immunogenicity of an Intranasal Live Attenuated Respiratory Syncytial Virus Vaccine Expressing Spike Protein of SARS-CoV-2 in Healthy Adults Ages 18 - 69 Years<br>ClinicalTrials.gov (2021)<br>NCT04798001	ClinicalTrials.gov registry report	Excluded<br>Intranasal live-attenuated RSV vector expressing SARS-CoV-2 spike, rather than RSVPreF3 OA.
000789	Phase 3 clinical trial results for nirsevimab: Is it time to consider population-level RSV immunoprophylaxis?<br>Journal of Paediatrics and Child Health (2022); pp. 1117–1118<br>DOI: 10.1111/jpc.15999	Commentary	Excluded<br>Comment on a nirsevimab trial and infant immunoprophylaxis; not an original eligible RSV vaccine trial report.
000790	Jaidhauser, I et al.<br>Phase 3, open-label, randomized, controlled study to evaluate immune response, safety and reactogenicity of the RSVPreF3 OA vaccine when coadministered with FLU-QIV-HD vaccine in adults aged ≥65 years<br>Innere Medizin (2024); P17-03, pp. S115–S116<br>DOI: 10.1007/s00108-024-01701-7<br>NCT05559476	Conference abstract	Associated report<br>Associated conference report of NCT05559476.
000791	Chime, N. et al.<br>Phase 3 Study Assessing Lot-to-Lot Consistency of Respiratory Syncytial Virus Prefusion Protein F3 Vaccine and Its Immune Response, Safety, and Reactogenicity When Co-administered With Quadrivalent Influenza Vaccine<br>Journal of Infectious Diseases (2025); pp. e144–e153<br>DOI: 10.1093/infdis/jiae342<br>NCT05045144	Journal article	Excluded<br>Unadjuvanted RSVPreF3 maternal vaccine in nonpregnant women aged 18–49 years; not the adjuvanted RSVPreF3 OA product.

Record	Report and bibliographic identifier	Report type	Eligibility disposition
000804	Muder, R.R.<br>Pneumonia in residents of long-term care facilities: Epidemiology, etiology, management, and prevention<br>American Journal of Medicine (1998); pp. 319–330<br>DOI: 10.1016/s0002-9343(98)00262-9	Review	Excluded<br>Review of pneumonia epidemiology, management and prevention in long-term care; not an original eligible trial.
000810	Fust, K. et al.<br>Potential Clinical Impact of Respiratory Syncytial Virus (RSV) Vaccination in Older Adults in the United States<br>Open Forum Infectious Diseases (2022); pp. S107<br>DOI: 10.1093/ofid/ofac492.194	Conference abstract	Excluded<br>Decision-analytic model of hypothetical RSV vaccination impact; not a randomized trial.
000814	Laurichesse, H. et al.<br>Pre- and in-hospital management of community-acquired pneumonia in Southern France, 1998-99<br>European Journal of Clinical Microbiology and Infectious Diseases (2001); pp. 770–778<br>DOI: 10.1007/s100960100613	Journal article	Excluded<br>Prospective observational study of community-acquired pneumonia management in southern France; not a vaccine trial.
000815	Fuentes, S. et al.<br>Preclinical evaluation of bacterially produced RSV-G protein vaccine: Strong protection against RSV challenge in cotton rat model<br>Scientific reports (2017); pp. 42428<br>DOI: 10.1038/srep42428	Journal article	Excluded<br>Bacterially produced RSV G-protein vaccination and challenge in cotton rats.
000828	Babawale, P.I. et al.<br>Preventing RSV Infection in Children: Current Passive Immunizations and Vaccine Development<br>Pathogens (2025)<br>DOI: 10.3390/pathogens14020104	Review	Excluded<br>Review of pediatric passive immunization and RSV vaccine development; not an original eligible trial.
000842	Barrella Harboe, Z. et al.<br>Prioritising at-risk adults in vaccine coadministration trials<br>The Lancet Infectious Diseases (2025); pp. 362–363<br>DOI: 10.1016/s1473-3099(24)00674-1	Commentary	Excluded<br>Comment on the Goswami mRNA-1345 coadministration trial; no independent trial dataset.
000846	Abbasi, A. et al.<br>Propagation Properties of a New Human Diploid Cell Line, RAZI-HDC, and Its Suitability as a Candidate Cell Substrate for Respiratory Syncytial Virus Vaccine Production in Comparison to MRC-5<br>Archives of Razi Institute (2025); pp. 51–59<br>DOI: 10.32592/ari.2025.80.1.51	Journal article	Excluded<br>Development of a human diploid cell substrate for vaccine production; not a clinical vaccination-schedule trial.

Record	Report and bibliographic identifier	Report type	Eligibility disposition
000854	Riaz, R. et al.<br>Protecting our future: call for respiratory syncytial virus vaccine trial in infants<br>Annals of Medicine and Surgery (2024); pp. 4960–4962<br>DOI: 10.1097/ms9.0000000000002385	Editorial	Excluded<br>Editorial calling for infant RSV vaccine trials; not a report of an original eligible trial.
000877	Buynak, R et al.<br>Randomized, Open-Label Phase 3 Study Evaluating Immunogenicity, Safety, and Reactogenicity of RSVPreF3 OA Coadministered with FLU-QIV-HD in Adults Aged ≥ 65<br>Infectious Diseases And Therapy (2024); pp. 1789–1805<br>DOI: 10.1007/s40121-024-00985-4<br>NCT05559476	Journal article	Primary trial article<br>Eligible RSVPreF3 OA randomized coadministration versus sequential trial; primary article for outcome extraction.
000882	Kuitunen, I. et al.<br>Rapid review showed that real-world results on how nirsevimab prevented respiratory syncytial virus hospitalisations were similar to randomised trials<br>Acta Paediatrica, International Journal of Paediatrics (2024); pp. 1940–1941<br>DOI: 10.1111/apa.17301	Rapid review	Excluded<br>Rapid review of nirsevimab effectiveness; not an original RSVPreF3 OA trial report.
000908	Walsh, E.E. et al.<br>RENOIR Trial — RSVpreF Vaccine Efficacy over Two Seasons: To the Editor<br>New England Journal of Medicine (2024); pp. 1459–1460<br>DOI: 10.1056/nejmc2311560	Letter	Excluded<br>Letter concerning RENOIR vaccine efficacy; not an original eligible coadministration trial report.
000909	Stokes, A.H. et al.<br>Repeated Dose Toxicity Study and Developmental and Reproductive Toxicology Studies of a Respiratory Syncytial Virus Candidate Vaccine in Rabbits and Rats<br>International Journal of Toxicology (2021); pp. 125–142<br>DOI: 10.1177/1091581820985782	Journal article	Excluded<br>Repeated-dose and reproductive toxicology studies in rabbits and rats.
000914	Lokteff, M. et al.<br>Residual DNA quantification in clinical batches of BBG2Na, a recombinant subunit vaccine against human respiratory syncytial virus<br>Biologicals (2001); pp. 123–132<br>DOI: 10.1006/biol.2001.0283	Journal article	Excluded<br>Residual-DNA quantification in BBG2Na vaccine manufacturing batches; not a clinical schedule comparison.
000918	Chávez-Bueno, S. et al.<br>Respiratory syncytial virus bronchiolitis: Current and future strategies for treatment and prophylaxis<br>Treatments in Respiratory Medicine (2006); pp. 483–494<br>DOI: 10.2165/00151829-200605060-00011	Review	Excluded<br>Review of RSV bronchiolitis treatment and prophylaxis; not an original eligible trial report.

Record	Report and bibliographic identifier	Report type	Eligibility disposition
000924	Berni Canani, M. et al.<br>Respiratory syncytial virus infection<br>Rivista Italiana di Pediatria (1998); pp. 617–621	Conference paper	Excluded<br>Italian conference paper on RSV infection in a pediatric journal; not independently eligible under the current report-format criteria.
000932	Bigoni, T. et al.<br>Respiratory Syncytial Virus Prevention in the Adult Population: State of the Art<br>Seminars in Respiratory and Critical Care Medicine (2025); pp. 41–52<br>DOI: 10.1055/a-2586-3974	Review	Excluded<br>Review of RSV prevention strategies; not an original randomized coadministration trial report.
000933	Greenough, A. et al.<br>Respiratory syncytial virus prevention: past and present strategies.<br>Expert opinion on pharmacotherapy (2000); pp. 1195–1201	Review	Excluded<br>Review of RSV prevention strategies; not an original randomized coadministration trial report.
000936	Branche, A.R. et al.<br>Respiratory Syncytial Virus Risk Assessment in Adults: Implications for Vaccine Recommendations Beyond Age<br>Open Forum Infectious Diseases (2025)<br>DOI: 10.1093/ofid/ofaf394	Journal article	Excluded<br>Observational assessment of RSV hospitalization risk factors; not a randomized vaccination-schedule trial.
000943	Niu, X. et al.<br>Respiratory Syncytial Virus (RSV) Prophylaxis: Clesrovimab and Beyond<br>Journal of Medicinal Chemistry (2026); pp. 4–9<br>DOI: 10.1021/acs.jmedchem.5c03667	Editorial	Excluded<br>Editorial on clesrovimab and RSV prophylaxis; not an original eligible vaccine trial report.
000951	Welliver, R.C. et al.<br>Respiratory syncytial virus-specific cell-mediated immune responses after vaccination with a purified fusion protein subunit vaccine<br>Journal of Infectious Diseases (1994); pp. 425–428	Journal article	Excluded<br>PFP-2 subunit RSV vaccine in children aged 12–18 months; outside the product and population scope.
000952	Zeme, M. et al.<br>Respiratory Syncytial Virus Vaccination Acceptance: The Importance of Asking “Why?”<br>Journal of Pediatrics: Clinical Practice (2025)<br>DOI: 10.1016/j.jpedcp.2025.200163	Commentary	Excluded<br>Commentary on reasons for vaccine acceptance; not an original randomized schedule trial report.

Record	Report and bibliographic identifier	Report type	Eligibility disposition
000955	Alfaro, T et al.<br>Respiratory syncytial virus vaccination in older adults and patients with chronic disorders: A position paper from the Portuguese Society of Pulmonology, the Portuguese Association of General and Family Medicine, the Portuguese Society of Cardiology, the Portuguese Society of Infectious Diseases and Clinical Microbiology, the Portuguese Society of Endocrinology, Diabetes and Metabolism, and the Portuguese Society of Internal Medicine<br>Pulmonology (2025); pp. 2451456<br>DOI: 10.1080/25310429.2025.2451456	Position paper	Excluded<br>Professional-society position paper on RSV vaccination; not an original randomized trial report.
000958	Mayer, E.F. et al.<br>Respiratory Syncytial Virus Vaccination in Solid Organ Transplant Recipients: Interim Findings From a Phase 3 Trial of mRNA-1345<br>Clinical Infectious Diseases (2026); pp. e1112–e1121<br>DOI: 10.1093/cid/ciag108<br>NCT06067230	Journal article	Excluded<br>mRNA-1345 RSV vaccine study, rather than RSVPreF3 OA.
000964	Meinrenken, S.<br>Respiratory syncytial virus vaccine is successful in phase 2a trial<br>Pneumologie (2022); pp. 849–850<br>DOI: 10.1055/a-1947-3783	Research news	Excluded<br>Research news note summarizing a phase 2a RSV vaccine study; not an original trial report.
000969	Fulginiti, V.A. et al.<br>Respiratory virus immunization. I. A field trial of two inactivated respiratory virus vaccines; an aqueous trivalent parainfluenza virus vaccine and an alum-precipitated respiratory syncytial virus vaccine.<br>American journal of epidemiology (1969); pp. 435–448	Journal article	Excluded<br>Historical inactivated parainfluenza and alum-precipitated RSV vaccines, rather than RSVPreF3 OA.
000971	Douglas Jr., R.G.<br>Respiratory virus vaccines: recent advances<br>Amer.Soc.Microbiol. (1975); pp. 409–415	Journal article	Excluded<br>Review of respiratory-virus vaccine development; not an original eligible trial report.
000988	Lenney, W.<br>RSV disease-Is there a role for prevention?<br>Respiratory Medicine (2001); pp. 170–172<br>DOI: 10.1053/rmed.2000.1018	Editorial	Excluded<br>Editorial on RSV disease prevention; not an original eligible randomized trial report.
000993	Terstappen, J. et al.<br>RSV neutralizing antibodies in dried blood<br>medRxiv (2023)<br>DOI: 10.1101/2023.08.10.23293875	Preprint	Excluded<br>Development and evaluation of an RSV neutralizing-antibody assay using dried blood, rather than a randomized vaccine-schedule comparison.

Record	Report and bibliographic identifier	Report type	Eligibility disposition
000994	Terstappen, J. et al.<br>RSV Neutralizing Antibodies in Dried Blood<br>Journal of Infectious Diseases (2024); pp. e93–e101<br>DOI: 10.1093/infdis/jiad543	Journal article	Excluded<br>Development and evaluation of an RSV neutralizing-antibody assay using dried blood, rather than a randomized vaccine-schedule comparison.
001000	Jairam, N. et al.<br>RSV Vaccination Increases CD4 T-Cell Response, but Not CD8, in Solid Organ Transplant Recipients<br>American Journal of Transplantation (2025); pp. S807–S808<br>DOI: 10.1016/j.ajt.2025.07.1909	Conference abstract	Excluded<br>Prospective T-cell-response study in solid organ transplant vaccine recipients; no randomized coadministration versus sequential comparison.
001002	[Anonymous]<br>RSV vaccine candidate immunogenic in older people<br>Human Vaccines & Immunotherapeutics (2017); pp. 1958–1958<br>DOI: 10.1080/21645515.2017.1374796	Research news	Excluded<br>RSV vaccine news subsection in Human Vaccines & Immunotherapeutics News; imported DOI and abstract were mismatched and are corrected in this ledger.
001019	Power, U.F. et al.<br>Safety and immunogenicity of a novel recombinant subunit respiratory syncytial virus vaccine (BBG2Na) in healthy young adults<br>Journal of Infectious Diseases (2001); pp. 1456–1460<br>DOI: 10.1086/324426	Journal article	Excluded<br>Recombinant BBG2Na RSV vaccine, rather than RSVPreF3 OA.
001028	Comeaux, C et al.<br>Safety and immunogenicity of a seasonal influenza vaccine and AD26.RSV.pref vaccine with and without co-administration: a randomized, double-blind, placebo-controlled phase 2A study in adults aged ≥ 60 years<br>Open forum infectious diseases (2019); pp. S979<br>DOI: 10.1093/ofid/ofz360.2452<br>NCT03339713	Conference abstract	Excluded<br>Ad26.RSV.preF, alone or combined with RSV preF protein, is outside the RSVPreF3 OA product scope.
001029	Glenn, G.M. et al.<br>Safety and immunogenicity of a Sf9 insect cell-derived respiratory syncytial virus fusion protein nanoparticle vaccine<br>Vaccine (2013); pp. 524–532<br>DOI: 10.1016/j.vaccine.2012.11.009<br>NCT01290419	Journal article	Excluded<br>RSV F protein nanoparticle vaccine, rather than adjuvanted RSVPreF3 OA.

Record	Report and bibliographic identifier	Report type	Eligibility disposition
001030	Hall, V.G. et al.<br>Safety and immunogenicity of adjuvanted respiratory syncytial virus vaccine in high-risk transplant recipients: an interventional cohort study<br>Clinical Microbiology and Infection (2026); pp. 161–168<br>DOI: 10.1016/j.cmi.2025.09.013<br>NCT06593210	Journal article	Excluded<br>Interventional transplant-recipient cohort; no randomized coadministration versus sequential comparison.
001034	Athan, Eugene et al.<br>Safety and Immunogenicity of Bivalent RSVpreF Vaccine Coadministered With Seasonal Inactivated Influenza Vaccine in Older Adults.<br>Clinical infectious diseases : an official publication of the Infectious Diseases Society of America (2024); pp. 1360–1368<br>DOI: 10.1093/cid/ciad707<br>NCT05301322	Journal article	Excluded<br>Pfizer bivalent RSVpreF vaccine, rather than adjuvanted RSVPreF3 OA.
001035	Sarwar, U.N. et al.<br>Safety and immunogenicity of bivalent RSVpreF vaccine formulated in a multidose vial in healthy female participants in the USA: a multicentre, randomised, open-label, noninferiority phase 3 study<br>eClinicalMedicine (2026)<br>DOI: 10.1016/j.eclinm.2026.103767<br>NCT06473519	Journal article	Excluded<br>Bivalent RSVpreF multidose-vial formulation study, rather than RSVPreF3 OA.
001036	Neutel, J et al.<br>Safety and Immunogenicity of Coadministered Bivalent BNT162b2 COVID-19 Vaccine and Bivalent RSVpreF Respiratory Syncytial Virus Vaccine With and Without Quadrivalent Influenza Vaccine in Adults ≥ 65 Years of Age<br>Open forum infectious diseases (2025); pp. S111–S112<br>DOI: 10.1093/ofid/ofae631.172<br>NCT05886777	Conference abstract	Excluded<br>Pfizer bivalent RSVpreF and BNT162b2 formulations, rather than RSVPreF3 OA.
001037	Neutel, J.M. et al.<br>Safety and Immunogenicity of Concomitant Administration and Combined Administration of Bivalent BNT162b2 COVID-19 Vaccine and Bivalent RSVpreF Respiratory Syncytial Virus Vaccine with or Without Quadrivalent Influenza Vaccine in Adults ≥ 65 Years of Age<br>Vaccines (2025)<br>DOI: 10.3390/vaccines13020158<br>NCT05886777	Journal article	Excluded<br>Pfizer bivalent RSVpreF and BNT162b2 formulations, rather than RSVPreF3 OA.

Record	Report and bibliographic identifier	Report type	Eligibility disposition
001038	Study Investigators<br>Safety and immunogenicity of mRNA-1345 RSV vaccine coadministered with an influenza or COVID-19 vaccine in adults aged 50 years or older: an observer-blinded, placebo controlled, randomised, phase 3 trial<br>Lancet Infectious Diseases (2025); pp. 411–423<br>DOI: 10.1016/s1473-3099(24)00589-9<br>NCT05330975	Journal article	Excluded<br>mRNA-1345 RSV vaccine, rather than RSVPreF3 OA; coadministration results are outside the current product scope.
001043	Clark, R et al.<br>Safety and Immunogenicity of Respiratory Syncytial Virus Prefusion F Protein Vaccine when Co-administered with Adjuvanted Seasonal Quadrivalent Influenza Vaccine in Older Adults: A Phase 3 Randomized Trial<br>Clinical Infectious Diseases (2024); pp. 1088–1098<br>DOI: 10.1093/cid/ciae365<br>NCT05568797	Journal article	Primary trial article<br>Eligible RSVPreF3 OA randomized coadministration versus sequential trial; primary article for outcome extraction.
001048	Sadoff, J. et al.<br>Safety and Immunogenicity of the Ad26.RSV.preF Investigational Vaccine Coadministered with an Influenza Vaccine in Older Adults<br>Journal of Infectious Diseases (2021); pp. 699–708<br>DOI: 10.1093/infdis/jiaa409<br>NCT03339713	Journal article	Excluded<br>Ad26.RSV.preF, alone or combined with RSV preF protein, is outside the RSVPreF3 OA product scope.
001049	Piedra, P.A. et al.<br>Safety and immunogenicity of the PFP vaccine against respiratory syncytial virus (RSV): The Western blot assay aids in distinguishing immune responses of the PFP vaccine from RSV infection<br>Vaccine (1995); pp. 1095–1101<br>DOI: 10.1016/0264-410x(95)00034-x	Journal article	Excluded<br>PFP-1 RSV vaccine in children with influenza vaccine as the comparator; outside the product, population and schedule scope.
001062	Shaw, C.A. et al.<br>Safety, Tolerability, and Immunogenicity of an mRNA-Based Respiratory Syncytial Virus Vaccine in Healthy Young Adults in a Phase 1 Clinical Trial<br>Journal of Infectious Diseases (2024); pp. e637–e646<br>DOI: 10.1093/infdis/jiae035<br>NCT04528719	Journal article	Excluded<br>mRNA-1345 RSV vaccine study, rather than RSVPreF3 OA.

Record	Report and bibliographic identifier	Report type	Eligibility disposition
001067	Mayer, EF et al.<br>Safety, Tolerability, and Immunogenicity of the mRNA-1345 RSV Vaccine in Solid Organ Transplant Recipients Aged ≥18 Years<br>Open forum infectious diseases (2026); pp. S55<br>DOI: 10.1093/ofid/ofaf695.081<br>NCT06067230	Conference abstract	Excluded<br>mRNA-1345 RSV vaccine study, rather than RSVPreF3 OA.
001076	Seroconversion Following RSV Vaccination in Bone Marrow Transplant and CAR-T Patients<br>ClinicalTrials.gov (2025)<br>NCT07296120	ClinicalTrials.gov registry report	Excluded<br>Abrysvo vaccination in hematopoietic transplant or CAR-T recipients; outside the RSVPreF3 OA product scope.
001077	Arankalle, Vidya A et al.<br>Seroepidemiology of respiratory syncytial virus in western India with special reference to appropriate age for infant vaccination.<br>Journal of medical virology (2019)<br>DOI: 10.1002/jmv.25489	Journal article	Excluded<br>RSV seroepidemiology in children in western India; not a vaccination-schedule trial.
001087	Fierro, C. et al.<br>Shared Clinical and Immunologic Features of mRNA Vaccines: Preliminary Results from a Comparative Clinical Study<br>medRxiv (2024)<br>DOI: 10.1101/2024.11.26.24318005<br>NCT05397223	Preprint	Excluded<br>mRNA-1345 RSV vaccine study, rather than RSVPreF3 OA.
001088	Fierro, C. et al.<br>Shared clinical and immunologic features of mRNA vaccines: preliminary results from a comparative clinical study<br>Frontiers in Immunology (2025)<br>DOI: 10.3389/fimmu.2025.1501275<br>NCT05397223	Journal article	Excluded<br>mRNA-1345 RSV vaccine study, rather than RSVPreF3 OA.
001089	Nguyen, T. et al.<br>Short term safety profile of respiratory syncytial virus vaccine in adults aged ≥ 60 years in Australia<br>The Lancet Regional Health - Western Pacific (2025)<br>DOI: 10.1016/j.lanwpc.2025.101506	Comment reporting surveillance data	Excluded<br>AusVaxSafety active postmarketing surveillance of reported adverse events; no randomized vaccination-schedule comparison.

Record	Report and bibliographic identifier	Report type	Eligibility disposition
001090	Yamaji, Y. et al.<br>Simultaneous administration of recombinant measles viruses expressing respiratory syncytial virus fusion (F) and nucleo (N) proteins induced humoral and cellular immune responses in cotton rats<br>Vaccines (2019)<br>DOI: 10.3390/vaccines7010027	Journal article	Excluded<br>Recombinant measles vectors expressing RSV proteins evaluated in a cotton-rat experiment.
001092	Biegus, J. et al.<br>Simultaneous vaccination against influenza and respiratory syncytial virus in high-risk heart failure patients<br>ESC Heart Failure (2025); pp. 3869–3877<br>DOI: 10.1002/ehf2.15432<br>NCT06259487	Journal article	Excluded<br>Coordinated RSV and influenza vaccination versus standard-care recommendations in heart failure; the comparator does not assign the same vaccine pair sequentially.
001097	Flynn, J.A. et al.<br>Stability characterization of a vaccine antigen based on the respiratory syncytial virus fusion glycoprotein<br>PLoS ONE (2016)<br>DOI: 10.1371/journal.pone.0164789	Journal article	Excluded<br>Biophysical stability characterization of an RSV antigen; not a clinical vaccination-schedule trial.
001100	Study on the Impact of Coordinated Immunizations Against RSV and Influenza on the Prognosis in Patients With Chronic Heart Failure.<br>ClinicalTrials.gov (2024)<br>NCT06259487	ClinicalTrials.gov registry report	Excluded<br>Coordinated RSV and influenza vaccination versus standard-care recommendations in heart failure; the comparator does not assign the same vaccine pair sequentially.
001104	Ellison III, R.T.<br>Surgical masks or N95 respirators for the flu?<br>Medicine Today (2009); pp. 69–70	Research commentary	Excluded<br>Surgical masks versus N95 respirators, rather than an RSV vaccination intervention.
001112	Biegus, J. et al.<br>The early safety profile of simultaneous vaccination against influenza and Respiratory Syncytial Virus (RSV) in patients with high-risk heart failure<br>Vaccine (2024); pp. 2937–2940<br>DOI: 10.1016/j.vaccine.2024.03.060<br>NCT06259487	Journal article	Excluded<br>Early single-arm safety report of same-day RSV and influenza vaccination in heart failure; no sequential comparator.
001120	Zhang, Lei et al.<br>The global trends and clinical progress in influenza co-infection: a visualization and bibliometric analysis (2005-2025).<br>Frontiers in microbiology (2025)<br>DOI: 10.3389/fmicb.2025.1658752	Bibliometric analysis	Excluded<br>Bibliometric analysis of influenza coinfection research; not an individual eligible randomized trial.

Record	Report and bibliographic identifier	Report type	Eligibility disposition
001133	Wright, P.F. et al.<br>The interferon antagonist NS2 protein of respiratory syncytial virus is an important virulence determinant for humans<br>Journal of Infectious Diseases (2006); pp. 573–581<br>DOI: 10.1086/499600	Journal article	Excluded<br>NS2-deleted live RSV vaccine candidates, rather than RSVPreF3 OA.
001137	Zhu, T. et al.<br>The preventive effect of vaccine prophylaxis on severe respiratory syncytial virus infection: A meta-analysis<br>Virologica Sinica (2015); pp. 371–378<br>DOI: 10.1007/s12250-015-3630-3	Meta-analysis	Excluded<br>Meta-analysis of RSV vaccine prophylaxis; not an original randomized trial report.
001143	Sacconnay, L. et al.<br>The RSVPreF3-AS01 vaccine elicits broad neutralization of contemporary and antigenically distant respiratory syncytial virus strains<br>Science Translational Medicine (2023)<br>DOI: 10.1126/scitranslmed.adg6050<br>NCT03814590	Journal article	Excluded<br>RSVPreF3-AS01 neutralization-breadth analysis across RSV strains; no partner-vaccine schedule comparison.
001147	The WHO Vaccine Trial Registry<br>European trial registry (2001); pp. 31–41<br>DOI: 10.1016/s0264-410x(01)00261-4	Registry methods review	Excluded<br>Descriptive report on the WHO Vaccine Trial Registry; not an individual eligible trial report.
001148	Francica, J.R. et al.<br>Thermoresponsive Polymer Nanoparticles Co-deliver RSV F Trimers with a TLR-7/8 Adjuvant<br>Bioconjugate Chemistry (2016); pp. 2372–2385<br>DOI: 10.1021/acs.bioconjchem.6b00370	Journal article	Excluded<br>Preclinical RSV F-trimer and TLR-7/8-adjuvant nanoparticle study; not clinical coadministration of two vaccines.
001159	Williams, G.P. et al.<br>Unaltered T cell responses to common antigens in individuals with Parkinson's disease<br>Journal of the Neurological Sciences (2023)<br>DOI: 10.1016/j.jns.2022.120510	Journal article	Excluded<br>Case-control comparison of antigen-specific T-cell responses in Parkinson disease; not a vaccination-schedule trial.
001172	Lequien, P.<br>Vaccination and extreme prematurity<br>Archives de Pediatrie (2002); pp. 431s–434s	Journal article	Excluded<br>Vaccination in extremely premature infants, outside the adult RSVPreF3 OA population.

Record	Report and bibliographic identifier	Report type	Eligibility disposition
001174	Luijckx, A.M.C. et al.<br>Vaccination of hospital staff against hepatitis B; the intramuscular method vs combined intradermal/intramuscular method<br>Nederlands Tijdschrift voor Geneeskunde (1985); pp. 2163–2166	Journal article	Excluded<br>Hepatitis B vaccination routes in hospital staff, rather than RSVPreF3 OA coadministration.
001176	Vaccination Response in Individual Monozygotic Twins<br>ClinicalTrials.gov (2014)<br>NCT02296359	ClinicalTrials.gov registry report	Excluded<br>Influenza-vaccine responses in monozygotic twins, rather than an RSVPreF3 OA vaccination-schedule comparison.
001179	McCool, RS et al.<br>Vaccination with prefusion-stabilized respiratory syncytial virus fusion protein elicits antibodies targeting a membrane-proximal epitope<br>Journal Of Virology (2023)<br>DOI: 10.1128/jvi.00929-23	Journal article	Excluded<br>Prefusion-stabilized DS-Cav1 vaccine immunology, rather than the RSVPreF3 OA product.
001180	Mukhamedova, M. et al.<br>Vaccination with prefusion-stabilized respiratory syncytial virus fusion protein induces genetically and antigenically diverse antibody responses<br>Immunity (2021); pp. 769–780.e6<br>DOI: 10.1016/j.immuni.2021.03.004	Journal article	Excluded<br>Prefusion-stabilized DS-Cav1 vaccine immunology, rather than the RSVPreF3 OA product.
001182	Seufert, J. et al.<br>Vaccinations in Patients with Diabetes: Current Recommendations<br>Diabetologie und Stoffwechsel (2025); pp. 347–359<br>DOI: 10.1055/a-2603-9028	Recommendations	Excluded<br>Vaccination recommendations for people with diabetes; not an original randomized trial report.
001185	Malarski, M. et al.<br>Vaccine impact on antimicrobial resistance to inform Gavi, the vaccine alliance’s 2018 vaccine investment strategy: Report from an expert survey.<br>F1000Research (2019)<br>DOI: 10.12688/f1000research.20100.1	Journal article	Excluded<br>Expert survey on vaccine impact on antimicrobial resistance, rather than an original randomized clinical comparison.
001186	Bartsch, Y.C. et al.<br>Vaccine-induced antibody Fc-effector functions in humans immunized with a combination Ad26.RSV.preF/RSV preF protein vaccine<br>Journal of Virology (2023)<br>DOI: 10.1128/jvi.00771-23<br>NCT03502707	Journal article	Excluded<br>Ad26.RSV.preF, alone or combined with RSV preF protein, is outside the RSVPreF3 OA product scope.

Record	Report and bibliographic identifier	Report type	Eligibility disposition
001189	Vaccine Responses in Patients With B Cell Malignancies<br>ClinicalTrials.gov (2021)<br>NCT05170399	ClinicalTrials.gov registry report	Excluded<br>Vaccine responses and possible BTK-inhibitor treatment interruption in B-cell malignancy; no assigned coadministration versus sequential vaccination schedules.
001190	Kricorian, C. et al.<br>Vaccine Status of Older Adults and Trust in Federal Institutions<br>Journal of the American Geriatrics Society (2026); pp. S244–S245<br>DOI: 10.1111/jgs.70425	Conference abstract	Excluded<br>Survey of vaccination status and trust in institutions, rather than a randomized vaccine-schedule comparison.
001192	Black, S. et al.<br>Vaccines and otitis media<br>Pediatric Annals (2000); pp. 648–651<br>DOI: 10.3928/0090-4481-20001001-10	Review	Excluded<br>Review of vaccines and otitis media; not an original eligible randomized trial report.
001197	Bermingham, A. et al.<br>VII international symposium on respiratory viral infections<br>Antiviral Therapy (2007); pp. 671–693	Symposium report	Excluded<br>Multi-topic symposium report on respiratory viral infections; not an individual eligible trial report.
001198	Rashid, H. et al.<br>Viral respiratory infections at the Hajj: Comparison between UK and Saudi pilgrims<br>Clinical Microbiology and Infection (2008); pp. 569–574<br>DOI: 10.1111/j.1469-0691.2008.01987.x	Journal article	Excluded<br>Comparison of respiratory-virus infections in UK and Saudi Hajj pilgrims; not a randomized vaccination-schedule trial.
001205	Rebuffo-Scheer, C. et al.<br>Whole genome sequencing and evolutionary analysis of human respiratory syncytial virus A and B from Milwaukee, WI 1998-2010<br>PLoS ONE (2011)<br>DOI: 10.1371/journal.pone.0025468	Journal article	Excluded<br>RSV genome sequencing and evolutionary analysis, rather than a clinical vaccine-schedule comparison.
001206	Cilloniz, C.<br>Why RSV Infection Matter in Older Adults? The Urgent Need for Vaccination<br>Archivos de Bronconeumologia (2025); pp. 654–656<br>DOI: 10.1016/j.arbres.2025.05.010	Editorial	Excluded<br>Editorial on RSV vaccination in older adults; not an original randomized trial report.

Table S2. Excluded reports with specific eligibility reasons (n = 195).

Record	Report and bibliographic identifier	Specific exclusion reason

Record	Report and bibliographic identifier	Specific exclusion reason
000001	Duckett, M. et al.<br>1076 Community Pharmacy Outreach Program to Promote RSV Vaccination in Eligible Older Adults and Improve Vaccination Rates<br>Journal of the American Pharmacists Association (2025)<br>DOI: 10.1016/j.japh.2025.102563<br>Conference abstract	E3: Community pharmacy outreach evaluates vaccination uptake, without randomized assignment to coadministration or sequential administration.
000005	Weiser, S. et al.<br>1100 Preparation Time and Usability Assessment of Commercially Available Freeze-Dried Respiratory Syncytial Virus Vaccine Presentations<br>Journal of the American Pharmacists Association (2025)<br>DOI: 10.1016/j.japh.2025.102587<br>Conference abstract	E2: Time-and-motion and usability assessment of vaccine preparation by healthcare providers, rather than a clinical vaccination-schedule trial.
000007	2019 IDSOG Annual Meeting<br>American Journal of Obstetrics and Gynecology (2019); pp. A1–A18<br>DOI: 10.1016/s0002-9378(19)31324-9<br>Meeting collection	E1: Meeting collection indexed as a conference review; its DOI identifies a table of contents, not an individual eligible trial report.
000028	A Partially Randomized, Partly Placebo Controlled Phase I Trial to Evaluate the Safety, Tolerability and Immunogenicity of the Recombinant MVA-BN® RSV Vaccine After Intranasal and Intramuscular Administration<br>ClinicalTrials.gov (2016)<br>NCT02864628<br>ClinicalTrials.gov registry report	E4: MVA-BN-RSV is a viral-vector vaccine, outside the RSVPreF3 OA product scope.
000029	A PHASE 1/2, PLACEBO-CONTROLLED, RANDOMIZED, OBSERVER-BLIND, DOSE-FINDING, FIRST-IN-HUMAN STUDY TO DESCRIBE THE SAFETY, TOLERABILITY, AND IMMUNOGENICITY OF A RESPIRATORY SYNCYTIAL VIRUS (RSV) VACCINE IN HEALTHY ADULTS<br>ClinicalTrials.gov (2018)<br>NCT03529773<br>ClinicalTrials.gov registry report	E4: Pfizer RSVpreF is a different RSV vaccine product from adjuvanted RSVPreF3 OA.
000030	A PHASE 1/2, PLACEBO-CONTROLLED, RANDOMIZED, OBSERVER-BLIND,DOSE-FINDING, FIRST-IN-HUMAN STUDY TO DESCRIBE THE SAFETY, TOLERABILITY, AND IMMUNOGENICITY OF AN ADJUVANTED RESPIRATORY SYNCYTIAL VIRUS (RSV) VACCINE IN HEALTHY OLDER ADULTS<br>ClinicalTrials.gov (2018)<br>NCT03572062<br>ClinicalTrials.gov registry report	E4: Pfizer RSVpreF is a different RSV vaccine product from adjuvanted RSVPreF3 OA.

Record	Report and bibliographic identifier	Specific exclusion reason
000034	Baber, J et al.<br>A Phase 1/2 Study of a Respiratory Syncytial Virus Prefusion F Vaccine With and Without Adjuvant in Healthy Older Adults<br>Journal Of Infectious Diseases (2022); pp. 2054–2063<br>DOI: 10.1093/infdis/jiac189<br>NCT03572062<br>Journal article	E4: Pfizer RSVpreF is a different RSV vaccine product from adjuvanted RSVPreF3 OA.
000039	A Phase 1, Randomized, Double-Blind, Placebo-Controlled, Dose-Ranging Trial to Determine the Safety and Immunogenicity of an Adenoviral-Vector Based Respiratory Syncytial Virus (RSV) F Protein Vaccine (VXA-RSV-f) Expressing Protein F and dsRNA Adjuvant Administered Orally to Healthy Volunteers<br>ClinicalTrials.gov (2016)<br>NCT02830932<br>ClinicalTrials.gov registry report	E4: Oral adenoviral-vector vaccine VXA-RSV-f, rather than RSVPreF3 OA.
000041	A Phase 1 Randomized, Observer Blind, Placebo Controlled, Dosage-Escalation Single Center Study to Evaluate the Safety and Immunogenicity of an RSV Fusion Glycoprotein (F) Subunit Vaccine in Healthy Adults<br>ClinicalTrials.gov (2014)<br>NCT02298179<br>ClinicalTrials.gov registry report	E5: Dose-escalation study of two RSV F subunit doses versus placebo; no randomized partner-vaccine coadministration versus sequential comparison.
000042	A Phase 1 Randomized, Observer-Blinded,Placebo-Controlled Trial to Evaluate the Safety and Immunogenicity of a Recombinant Respiratory Syncytial Virus F Protein Particle Vaccine in Healthy Adults<br>ClinicalTrials.gov (2011)<br>NCT01290419<br>ClinicalTrials.gov registry report	E4: RSV F protein nanoparticle vaccine, rather than adjuvanted RSVPreF3 OA.
000048	A Phase 1b Study to Evaluate the Safety and Immunogenicity of MEDI7510 in Older Adults<br>ClinicalTrials.gov (2014)<br>NCT02289820<br>ClinicalTrials.gov registry report	E4: MEDI7510 is a different RSV vaccine product from RSVPreF3 OA.

Record	Report and bibliographic identifier	Specific exclusion reason
000056	A Phase 2a, Randomised, Double-Blinded, Placebo-Controlled Study to Assess the Safety, Immunogenicity and Efficacy of the Recombinant MVA-BN®-RSV Vaccine Against Respiratory Syncytial Virus Infection in the Virus Challenge Model in Healthy Adult Participants<br>ClinicalTrials.gov (2021)<br>NCT04752644<br>ClinicalTrials.gov registry report	E4: MVA-BN-RSV is a viral-vector vaccine, outside the RSVPreF3 OA product scope.
000057	A Phase 2b, Non-randomized, Controlled, Open-label, Extension Study to Evaluate the Persistence of Immune Response of the Adjuvanted RSVPreF3 Vaccine and the Safety and Immunogenicity Following Revaccination in Lung and Kidney Transplant Recipients (>=18 Years of Age)<br>ClinicalTrials.gov (2025)<br>NCT07092865<br>ClinicalTrials.gov registry report	E3: Nonrandomized extension of an RSV vaccination study in transplant recipients; evaluates persistence and revaccination rather than assigned partner-vaccine schedules.
000059	A PHASE 2b, PLACEBO-CONTROLLED, RANDOMIZED, OBSERVER-BLIND STUDY TO EVALUATE THE SAFETY, TOLERABILITY, AND IMMUNOGENICITY OF A RESPIRATORY SYNCYTIAL VIRUS (RSV) VACCINE WHEN ADMINISTERED CONCOMITANTLY WITH TETANUS, DIPHTHERIA, AND ACELLULAR PERTUSSIS VACCINE (TDAP) IN HEALTHY NONPREGNANT WOMEN 18 THROUGH 49 YEARS OF AGE<br>ClinicalTrials.gov (2019)<br>NCT04071158<br>ClinicalTrials.gov registry report	E4: Pfizer RSVpreF is a different RSV vaccine product from adjuvanted RSVPreF3 OA.
000061	A PHASE 3, MULTICENTER, PARALLEL-GROUP, OPEN-LABEL STUDY TO EVALUATE THE SAFETY, TOLERABILITY, AND IMMUNOGENICITY OF RESPIRATORY SYNCYTIAL VIRUS PREFUSION F SUBUNIT VACCINE WHEN COADMINISTERED WITH HERPES ZOSTER SUBUNIT VACCINE IN ADULTS ≥50 YEARS OF AGE<br>ClinicalTrials.gov (2025)<br>NCT06890416<br>ClinicalTrials.gov registry report	E4: Pfizer RSVpreF is a different RSV vaccine product from adjuvanted RSVPreF3 OA.
000062	A PHASE 3, MULTICENTER, SINGLE-ARM, OPEN-LABEL STUDY TO EVALUATE THE SAFETY, TOLERABILITY, AND IMMUNOGENICITY OF RESPIRATORY SYNCYTIAL VIRUS (RSV) PREFUSION F SUBUNIT VACCINE IN ADULTS CONSIDERED AT HIGH RISK OF SEVERE RSV DISEASE IN JAPAN<br>ClinicalTrials.gov (2026)<br>NCT07543380<br>ClinicalTrials.gov registry report	E4: Pfizer RSVpreF is a different RSV vaccine product from adjuvanted RSVPreF3 OA.

Record	Report and bibliographic identifier	Specific exclusion reason
000068	A PHASE 3, RANDOMIZED, DOUBLE-BLIND, PLACEBO-CONTROLLED STUDY TO EVALUATE THE SAFETY, TOLERABILITY, AND IMMUNOGENICITY OF RESPIRATORY SYNCYTIAL VIRUS PREFUSION F SUBUNIT VACCINE WHEN COADMINISTERED WITH SEASONAL INACTIVATED INFLUENZA VACCINE IN ADULTS ≥65 YEARS OF AGE<br>ClinicalTrials.gov (2022)<br>NCT05301322<br>ClinicalTrials.gov registry report	E4: Pfizer RSVpreF is a different RSV vaccine product from adjuvanted RSVPreF3 OA.
000071	A Phase 3 Randomized, Observer-Blind, Study to Evaluate Safety, Tolerability, and Immunogenicity of mRNA-1345, an mRNA Vaccine Targeting Respiratory Syncytial Virus (RSV), When Given Alone or Coadministered With a Seasonal Influenza Vaccine or SARS-CoV-2 Vaccine and When Given as an Open-label Boost at 1 Year Following a Primary Dose in Adults ≥ 50 Years of Age<br>ClinicalTrials.gov (2022)<br>NCT05330975<br>ClinicalTrials.gov registry report	E4: mRNA-1345 RSV vaccine, rather than RSVPreF3 OA; coadministration results are outside the current product scope.
000072	A Phase 3, Randomized, Observer-Blind Study to Evaluate Safety, Tolerability, and Immunogenicity of mRNA-1345, an mRNA Vaccine Targeting Respiratory Syncytial Virus, When Coadministered With a High-Dose, Quadrivalent Seasonal Influenza Vaccine in Adults ≥65 Years of Age<br>ClinicalTrials.gov (2023)<br>NCT06060457<br>ClinicalTrials.gov registry report	E4: mRNA-1345 RSV vaccine, rather than RSVPreF3 OA; coadministration results are outside the current product scope.
000081	A Phase I Double-Blind Placebo Controlled Trial to Evaluate the Safety and Immunogenicity of Intranasal Sendai Virus Vectored Respiratory Syncytial Virus (SeVRSV) Vaccine in Healthy Adults<br>ClinicalTrials.gov (2018)<br>NCT03473002<br>ClinicalTrials.gov registry report	E4: Intranasal Sendai-virus-vectored RSV vaccine, rather than RSVPreF3 OA.
000089	A Phase I Randomized, Observer-Blinded, Dose-Ranging Study to Evaluate the Immunogenicity and Safety of a Respiratory Syncytial Virus (RSV) Recombinant Fusion (F) Nanoparticle Vaccine, With or Without Aluminum Adjuvant, in Healthy Subjects 24 to <72 Months of Age<br>ClinicalTrials.gov (2014)<br>NCT02296463<br>ClinicalTrials.gov registry report	E4: RSV F protein nanoparticle vaccine, rather than adjuvanted RSVPreF3 OA.

Record	Report and bibliographic identifier	Specific exclusion reason
000090	A Phase I Randomized, Observer-Blinded, Dose-Ranging Study to Evaluate the Immunogenicity and Safety of an RSV-F Protein Nanoparticle Vaccine, With or Without Aluminum Adjuvant, and Co-administered With a Licensed Inactivated Influenza Vaccine, in Healthy Subjects ≥ 60 Years of Age.<br>ClinicalTrials.gov (2012)<br>NCT01709019<br>ClinicalTrials.gov registry report	E4: RSV F protein nanoparticle vaccine, rather than adjuvanted RSVPreF3 OA.
000122	A Pragmatic Randomized Trial to Evaluate the Vaccine Effectiveness of Abrysvo® for Preventing RSV Hospitalizations in Adults Aged 18 Years or Above<br>ClinicalTrials.gov (2024)<br>NCT06684743<br>ClinicalTrials.gov registry report	E4: Abrysvo effectiveness trial comparing RSV vaccination with no RSV vaccination, outside the RSVPreF3 OA product scope.
000130	Langley, J.M. et al.<br>A Randomized, Controlled, Observer-Blinded Phase 1 Study of the Safety and Immunogenicity of a Respiratory Syncytial Virus Vaccine with or without Alum Adjuvant<br>Journal of Infectious Diseases (2017); pp. 24–33<br>DOI: 10.1093/infdis/jiw453<br>NCT01905215<br>Journal article	E4: First-generation RSV-PreF antigen at 10, 30 or 60 micrograms, with or without alum, in men aged 18–44 years; not RSVPreF3 OA.
000133	A Randomized, Double-blind, Placebo-controlled Phase 1/2 Clinical Trial to Evaluate the Safety, Tolerability, and Immunogenicity of Recombinant Respiratory Syncytial Virus Vaccine (SCTV02) in Healthy Adults ≥18 Years of Age<br>ClinicalTrials.gov (2024)<br>NCT06754605<br>ClinicalTrials.gov registry report	E4: Recombinant RSV vaccine SCTV02, rather than RSVPreF3 OA.
000137	A Randomized, Double-blind, Placebo-controlled Phase 2a Study to Evaluate the Safety and Immunogenicity of Seasonal Influenza Vaccine and Ad26.RSV.preF, With and Without Co-administration, in Adults Aged 60 Years and Older in Stable Health<br>ClinicalTrials.gov (2017)<br>NCT03339713<br>ClinicalTrials.gov registry report	E4: Ad26.RSV.preF, alone or combined with RSV preF protein, is outside the RSVPreF3 OA product scope.

Record	Report and bibliographic identifier	Specific exclusion reason
000138	A Randomized, Double-blind, Placebo-controlled Phase 3 Study to Evaluate the Immunogenicity and Safety of Ad26.RSV.preF-based Vaccine and High-dose Seasonal Influenza Vaccine, With and Without Coadministration, in Adults Aged 65 Years and Older<br>ClinicalTrials.gov (2021)<br>NCT05071313<br>ClinicalTrials.gov registry report	E4: Ad26.RSV.preF, alone or combined with RSV preF protein, is outside the RSVPreF3 OA product scope.
000142	A Randomized, Double-Blind, Placebo Controlled Trial of North American Ginseng Extract (CVT-E002; COLD-fX®) to Prevent Respiratory Infection and Reduce Antibiotic Use in Patients With Chronic Lymphocytic Leukemia<br>ClinicalTrials.gov (2008)<br>NCT00752895<br>ClinicalTrials.gov registry report	E4: American ginseng extract versus placebo for prevention of respiratory illness; not an RSV vaccine intervention.
000146	A Randomized, Single-blind, Placebo Controlled, Dose-ranging Phase II Trial in ≥ 55 Year Old Adults to Evaluate the Safety and Immunogenicity of the Recombinant MVA-BN-RSV Vaccine<br>ClinicalTrials.gov (2016)<br>NCT02873286<br>ClinicalTrials.gov registry report	E4: MVA-BN-RSV is a viral-vector vaccine, outside the RSVPreF3 OA product scope.
000147	A Randomized, Single-blind, Placebo-controlled Phase I Trial to Evaluate the Safety, Tolerability and Immunogenicity of the Recombinant MVA BN® RSV Vaccine in Healthy Adult Subjects<br>ClinicalTrials.gov (2015)<br>NCT02419391<br>ClinicalTrials.gov registry report	E4: MVA-BN-RSV is a viral-vector vaccine, outside the RSVPreF3 OA product scope.
000153	Henry, C. et al.<br>A Seasonal Influenza Hemagglutinin mRNA-Based Vaccine Induces Humoral and Cellular Immunity Comparable to an Adjuvanted Inactivated Influenza Virus Vaccine<br>Open Forum Infectious Diseases (2026); pp. S925<br>DOI: 10.1093/ofid/ofaf695.1638<br>NCT05397223<br>Conference abstract	E4: mRNA-1010 versus FLUAD influenza vaccination; no RSVPreF3 OA intervention.

Record	Report and bibliographic identifier	Specific exclusion reason
000173	A STUDY TO EVALUATE THE SAFETY, TOLERABILITY, AND IMMUNOGENICITY OF COMBINED VACCINE CANDIDATE(S) AGAINST INFECTIOUS RESPIRATORY ILLNESSES, INCLUDING COVID-19 AND RSV, IN HEALTHY INDIVIDUALS<br>ClinicalTrials.gov (2023)<br>NCT05886777<br>ClinicalTrials.gov registry report	E4: Pfizer bivalent RSVpreF and BNT162b2 formulations, rather than RSVPreF3 OA.
000174	Maltezou, H.C. et al.<br>Absenteeism due to COVID-19, influenza and RSV among hospital-based healthcare personnel in Greece during the 2024–2025 season: A cohort study<br>Infection, Disease and Health (2026)<br>DOI: 10.1016/j.idh.2026.100448<br>Journal article	E3: Healthcare-personnel cohort measuring respiratory-virus-associated absenteeism; not a randomized vaccination-schedule comparison.
000180	Broad, Jonathan et al.<br>Adapting COVID-19 research infrastructure to capture influenza and respiratory syncytial virus alongside SARS-CoV-2 in UK healthcare workers winter 2022/23 and beyond: protocol for a pragmatic sub-study.<br>NIHR open research (2024)<br>DOI: 10.3310/nihropenres.13517.3<br>Journal article	E3: Protocol for an observational healthcare-worker surveillance cohort, rather than an RSVPreF3 OA coadministration trial.
000184	Najdowski, M. et al.<br>Adult Respiratory Syncytial Virus Vaccination Receipt among Adults Testing Negative for RSV - VISION Network, October 1, 2024-March 31, 2025<br>Open Forum Infectious Diseases (2026); pp. S958–S959<br>DOI: 10.1093/ofid/ofaf695.1701<br>Conference abstract	E3: VISION Network observational analysis of vaccination receipt among adults testing negative for RSV; no randomized schedule comparison.
000188	Kwetkat, A. et al.<br>After the COVID-19 pandemic—Which new vaccinations for adults are available or coming soon?<br>Innere Medizin (Germany) (2024); pp. 79–85<br>DOI: 10.1007/s00108-023-01640-9<br>Review	E1: Overview of adult vaccines and recommendations; not an original randomized trial report.

Record	Report and bibliographic identifier	Specific exclusion reason
000191	Keus, J.A.R.<br>An accidental human trial of recombinant vaccinia virus: A step towards acceptance of live recombinant vaccines?<br>Trends in Biotechnology (1986); pp. 105–106<br>DOI: 10.1016/0167-7799(86)90133-2<br>Journal report	E4: Report concerns recombinant vaccinia virus, rather than the RSVPreF3 OA vaccination-schedule intervention.
000194	An Exploratory Pilot Study to Evaluate the Clinical Safety and Virologic Profile of an Experimental RSV Infection in Adults 60 to 75 Years of Age<br>ClinicalTrials.gov (2019)<br>NCT03919591<br>ClinicalTrials.gov registry report	E5: Experimental RSV infection challenge study; no partner-vaccine coadministration versus sequential comparison.
000195	An Observer-blind Safety and Reactogenicity Study to Assess GlaxoSmithKline (GSK) Biologicals' Investigational Respiratory Syncytial Virus (RSV) Vaccine (GSK3003891A) in Healthy Women<br>ClinicalTrials.gov (2016)<br>NCT02753413<br>ClinicalTrials.gov registry report	E4: GSK3003891A first-generation RSV vaccine formulation studies, rather than RSVPreF3 OA coadministration.
000196	An Observer-blind Study to Assess the Safety, Reactogenicity and Immunogenicity of Different Formulations of GSK Biologicals' Investigational RSV Vaccine (GSK3003891A), in Healthy Women<br>ClinicalTrials.gov (2015)<br>NCT02360475<br>ClinicalTrials.gov registry report	E4: GSK3003891A first-generation RSV vaccine formulation studies, rather than RSVPreF3 OA coadministration.
000198	An Observer-blind Study to Evaluate the Safety, Reactogenicity and Immunogenicity of GSK Biologicals' Respiratory Syncytial Virus (RSV) Investigational Vaccine (GSK3003891A) in Healthy Men<br>ClinicalTrials.gov (2013)<br>NCT01905215<br>ClinicalTrials.gov registry report	E4: GSK3003891A first-generation RSV vaccine formulation studies, rather than RSVPreF3 OA coadministration.
000208	Karaba, A.H. et al.<br>Antibody Response to Respiratory Syncytial Virus Vaccination in Immunocompromised Persons<br>Jama (2025); pp. 429–432<br>DOI: 10.1001/jama.2024.25395<br>Research letter reporting a cohort	E3: Prospective observational cohort of immunocompromised vaccine recipients with paired antibody samples; vaccination schedules were not randomized.

Record	Report and bibliographic identifier	Specific exclusion reason
000210a	Aung, G. et al.<br>AS01E-ADJUVANTED RSV PREFUSION F PROTEIN VACCINE REDUCES RSV ACUTE RESPIRATORY ILLNESS-RELATED COMPLICATIONS AND MEDICATION USE IN PARTICIPANTS WITH COPD/ASTHMA<br>Internal Medicine Journal (2026); pp. 178<br>DOI: 10.1111/imj.70364<br>NCT04886596<br>Conference abstract	E5: Post-hoc RSVPreF3 efficacy analysis in COPD or asthma; vaccine versus placebo, without a partner-vaccine schedule comparison.
000210b	Aung, G. et al.<br>IMMUNOGENICITY AND SAFETY OF AS01E-ADJUVANTED RSVPREF3 VACCINE IN ADULTS 18–49 AT RISK AND ≥60 YOA THROUGH 6 MONTHS<br>Internal Medicine Journal (2026); pp. 183<br>DOI: 10.1111/imj.70364<br>NCT06389487<br>Conference abstract	E5: Age-based immunobridging after one RSVPreF3 dose; no randomized partner-vaccine schedule comparison.
000211	McDonald, J.U. et al.<br>Assessing commutability of the first WHO International Standard for antiserum to respiratory syncytial virus<br>Vaccine (2025)<br>DOI: 10.1016/j.vaccine.2025.127430<br>Journal article	E2: Commutability study of a WHO antiserum standard for RSV assays; not a clinical vaccination-schedule comparison.
000217	Simmons, A.E. et al.<br>Association Between Seasonal Respiratory Virus Activity and Invasive Pneumococcal Disease in Central Ontario, Canada<br>medRxiv (2024)<br>DOI: 10.1101/2024.09.03.24312990<br>Preprint	E3: Observational analysis of respiratory-virus activity and invasive pneumococcal disease in Ontario; not a vaccine trial.
000218	Simmons, A.E. et al.<br>Association between seasonal respiratory virus activity and invasive pneumococcal disease in central Ontario, Canada<br>BMC Infectious Diseases (2026)<br>DOI: 10.1186/s12879-025-12419-8<br>Journal article	E3: Observational analysis of respiratory-virus activity and invasive pneumococcal disease in Ontario; not a vaccine trial.

Record	Report and bibliographic identifier	Specific exclusion reason
000221	Karaba, A.H. et al.<br>Attenuated PreFusion F Antibody Response to RSV Vaccines in Solid Organ Transplant Recipients<br>Open Forum Infectious Diseases (2025); pp. S49–S50<br>DOI: 10.1093/ofid/ofae631.076<br>Conference abstract	E3: Prospective antibody-response cohort of solid organ transplant recipients; no randomized coadministration versus sequential comparison.
000223	Golmakani, E. et al.<br>Bacterial and Viral Respiratory Co-infections With SARS-CoV-2 in Deceased Patients of North Khorasan, Iran, During 2020-2023<br>Avicenna Journal of Clinical Microbiology and Infection (2026); pp. 66–71<br>DOI: 10.34172/ajcmi.3719<br>Journal article	E3: Observational comparison of bacterial and viral coinfections in SARS-CoV-2-positive patients; not a vaccination-schedule trial.
000224	Lingblom, C.M.D. et al.<br>Baseline immune profile by CyTOF can predict response to an investigational adjuvanted vaccine in elderly adults<br>Journal of Translational Medicine (2018); pp. 153<br>DOI: 10.1186/s12967-018-1528-1<br>NCT02115815<br>Journal article	E5: CyTOF analysis of vaccine responders and nonresponders from a phase 1a RSV study; no partner-vaccine schedule comparison.
000233	Glanternik, J. et al.<br>Bivalent RSVpreF Subunit Vaccine Safety and Immunogenicity in Seropositive 2–<18 Year Olds<br>Vaccines (2026)<br>DOI: 10.3390/vaccines14020128<br>NCT05900154<br>Journal article	E4: Bivalent RSVpreF study in children aged 2 to under 18 years; outside the product and population scope.
000247	Pershagen, G.<br>Can immunization affect the development of allergy?<br>Pediatric Allergy and Immunology, Supplement (2000); pp. 26–28<br>DOI: 10.1034/j.1399-3038.2000.00507.x<br>Review	E1: Review of immunization and allergic disease; not an original eligible randomized comparison.

Record	Report and bibliographic identifier	Specific exclusion reason
000263	Pacheco, G.A. et al.<br>Characterization of the humoral and cellular immunity induced by a recombinant BCG vaccine for the respiratory syncytial virus in healthy adults<br>Frontiers in Immunology (2023)<br>DOI: 10.3389/fimmu.2023.1215893<br>NCT03213405<br>Journal article	E4: Recombinant BCG RSV vaccine, rather than RSVPreF3 OA.
000274	Venkatesan, P<br>Co-administered RSV and pneumococcal disease vaccines in older adults<br>Lancet Infectious Diseases (2026); pp. e216–e216<br>DOI: 10.1016/s1473-3099(26)00248-3<br>Research news	E1: Research news item. Author, journal, volume, issue and page identify the parent Research in brief report; it is not an original randomized trial report.
000283	Baber, JA et al.<br>Coadministration of Bivalent Respiratory Syncytial Virus (RSVpreF) Vaccine With Influenza Vaccine in Older Adults<br>Open forum infectious diseases (2023); pp. S58–S59<br>DOI: 10.1093/ofid/ofad500.095<br>NCT05301322<br>Conference abstract	E4: Pfizer bivalent RSVpreF vaccine, rather than adjuvanted RSVPreF3 OA.
000284	Comstock, E. et al.<br>Coadministration of mRNA-1345 RSV vaccine with high-dose quadrivalent influenza vaccine in adults aged 65 and older: An observer-blinded, placebo-controlled, randomized, phase 3 trial<br>Human Vaccines and Immunotherapeutics (2026); pp. 2649335<br>DOI: 10.1080/21645515.2026.2649335<br>NCT06060457<br>Journal article	E4: mRNA-1345 RSV vaccine, rather than RSVPreF3 OA; coadministration results are outside the current product scope.
000285	Dacso, C.C.<br>Coadministration of RSV + influenza or COVID-19 vaccines was noninferior to separate administration for immune responses in adults aged ≥50 y<br>Annals of Internal Medicine (2025); pp. JC41<br>DOI: 10.7326/annals-25-00904-jc<br>ACP Journal Club summary	E1: ACP Journal Club evidence summary of a published coadministration trial; no independent trial dataset.

Record	Report and bibliographic identifier	Specific exclusion reason
000296	Ackerson, B.K. et al.<br>Comparison of Hospitalization Costs Associated With Human Metapneumovirus and Respiratory Syncytial Virus Infection in US Adults<br>Open Forum Infectious Diseases (2026)<br>DOI: 10.1093/ofid/ofag202<br>Journal article	E3: Observational comparison of hospitalization costs for RSV and human metapneumovirus infections; not a vaccination-schedule trial.
000301	Kachikis, A. et al.<br>Comparison of Respiratory Syncytial Virus (RSV)-Specific Antibody Durability in Pregnant/Postpartum Individuals and Older Adults After RSV Vaccination<br>The Journal of infectious diseases (2026)<br>DOI: 10.1093/infdis/jiag111<br>Journal article	E3: Prospective comparison of antibody durability in pregnant or postpartum individuals and older adults; no randomized schedule comparison.
000302	Kachikis, A.B. et al.<br>Comparison of Respiratory Syncytial Virus-Specific Antibody Durability in Pregnant/Postpartum Individuals and Older Adults After RSV Vaccination<br>Open Forum Infectious Diseases (2026); pp. S903–S904<br>DOI: 10.1093/ofid/ofaf695.1606<br>Conference abstract	E3: Prospective comparison of antibody durability in pregnant or postpartum individuals and older adults; no randomized schedule comparison.
000308	Falsey, A.R. et al.<br>Comparison of the safety and immunogenicity of 2 Respiratory Syncytial Virus (RSV) vaccines - Nonadjuvanted vaccine or vaccine adjuvanted with alum - Given concomitantly with influenza vaccine to high-risk elderly individuals<br>Journal of Infectious Diseases (2008); pp. 1317–1326<br>DOI: 10.1086/592168<br>Journal article	E4: Older nonadjuvanted or alum-adjuvanted RSV formulations with trivalent influenza vaccine, rather than RSVPreF3 OA; controls received influenza vaccine with placebo.
000310	Kautz, M. et al.<br>Compliance and Performance Characteristics of Subject Collected Versus Health-care Worker Collected Nasal Swabs for Respiratory Viral Surveillance<br>Open Forum Infectious Diseases (2021); pp. S440–S441<br>DOI: 10.1093/ofid/ofab466.874<br>Conference abstract	E2: Evaluation of participant-collected versus healthcare-worker-collected nasal swabs, rather than vaccination schedules.

Record	Report and bibliographic identifier	Specific exclusion reason
000315	Widagdo, W et al.<br>Concomitant Administration of Ad26.RSV.preF/RSV preF Protein Vaccine and High-Dose Influenza Vaccine in Adults 65 Years and Older: A Noninferiority Trial<br>Journal Of Infectious Diseases (2024); pp. e374–e383<br>DOI: 10.1093/infdis/jiad594<br>NCT05071313<br>Journal article	E4: Ad26.RSV.preF, alone or combined with RSV preF protein, is outside the RSVPreF3 OA product scope.
000319	Dayananda, P. et al.<br>Controlled Human Infection Challenge Studies with RSV<br>Current Topics in Microbiology and Immunology (2024); pp. 41–68<br>DOI: 10.1007/82_2022_257<br>Book chapter	E1: Book chapter reviewing controlled human RSV infection studies; not an individual eligible coadministration trial report.
000357	CSP #2038 - COVID-19 Pharmacotherapy Effectiveness in the VA Healthcare System (COPE-VA)<br>ClinicalTrials.gov (2023)<br>NCT06160128<br>ClinicalTrials.gov registry report	E3: Observational evaluation of COVID-19 pharmacotherapy in the VA healthcare system; not an RSV vaccine-schedule trial.
000358	Neuzil, K.M.<br>Current and Emerging Approaches to Evaluating Influenza Vaccine Performance<br>New England Journal of Medicine (2025); pp. 2361–2362<br>DOI: 10.1056/nejme2513191<br>Editorial	E1: Editorial on evaluation of influenza vaccine performance; not an original RSV coadministration trial report.
000363	Nyiro, J.U. et al.<br>Defining the vaccination window for Respiratory syncytial virus (RSV) using ageseroprevalence data for children in Kilifi, Kenya<br>PLoS ONE (2017)<br>DOI: 10.1371/journal.pone.0177803<br>Journal article	E3: Age-seroprevalence study in children, rather than a randomized vaccination-schedule study.
000367	Watt, P.J. et al.<br>Determinants of susceptibility to challenge and the antibody response of adult volunteers given experimental respiratory syncytial virus vaccines<br>Vaccine (1990); pp. 231–236<br>DOI: 10.1016/0264-410x(90)90051-m<br>Journal article	E4: Experimental temperature-sensitive live RSV vaccines and challenge, rather than RSVPreF3 OA.

Record	Report and bibliographic identifier	Specific exclusion reason
000369	Itell, H.L. et al.<br>Development and application of a multiplex assay for the simultaneous measurement of antibody responses elicited by common childhood vaccines<br>Vaccine (2018); pp. 5600–5608<br>DOI: 10.1016/j.vaccine.2018.07.048<br>Journal article	E2: Multiplex assay development for antibody responses to childhood vaccines; not a clinical RSV coadministration trial.
000370	Anderson, L.J. et al.<br>Development and comparison of immunologic assays to detect primary RSV infections in infants<br>Frontiers in Immunology (2023)<br>DOI: 10.3389/fimmu.2023.1332772<br>Journal article	E2: Development and comparison of assays for primary RSV infection in infants; not a vaccination-schedule trial.
000372	Schepp, RM et al.<br>Development and Standardization of a High-Throughput Multiplex Immunoassay for the Simultaneous Quantification of Specific Antibodies to Five Respiratory Syncytial Virus Proteins<br>Msphere (2019)<br>DOI: 10.1128/msphere.00236-19<br>Journal article	E2: Development and standardization of a respiratory-antibody multiplex immunoassay; not an eligible clinical trial.
000376	Kumari, S. et al.<br>Development of a luciferase immunoprecipitation system assay to detect IgG antibodies against human respiratory syncytial virus nucleoprotein<br>Clinical and Vaccine Immunology (2014); pp. 383–390<br>DOI: 10.1128/cvi.00594-13<br>Journal article	E2: Development of an RSV nucleoprotein antibody assay; not an eligible clinical vaccination-schedule comparison.
000417	Effect of Montelukast on Experimentally-Induced RV16 Infection in Volunteers With Mild Asthma<br>ClinicalTrials.gov (2006)<br>NCT00359073<br>ClinicalTrials.gov registry report	E4: Montelukast in experimentally induced rhinovirus infection; not an RSV vaccine intervention.

Record	Report and bibliographic identifier	Specific exclusion reason
000419	Skaarup, K.G. et al.<br>Effect of RSV Vaccine on Heart Failure Hospitalizations: A Prespecified Analysis of the DAN-RSV Trial<br>Journal of the American College of Cardiology (2025); pp. 2062–2067<br>DOI: 10.1016/j.jacc.2025.08.023<br>Journal article	E4: DAN-RSV subgroup report evaluates bivalent RSVpreF versus no vaccination, outside the RSVPreF3 OA product scope.
000449	Efficacy of bivalent prefusion F vaccine during pregnancy in preventing respiratory syncytial virus-related diseases in infants: a phase III, double-blind, randomized controlled trial<br>Chinese Journal of Perinatal Medicine (2023); pp. 495<br>DOI: 10.3760/cma.j.issn.1007-9408.2023.06.104<br>Secondary research summary	E1: Indexed as a note summarizing a maternal bivalent RSV vaccine efficacy trial with infant outcomes; not an original RSVPreF3 OA schedule trial report.
000459	Phung, E. et al.<br>Elicitation of pneumovirus-specific B cell responses by a prefusion-stabilized respiratory syncytial virus F subunit vaccine<br>Science Translational Medicine (2022)<br>DOI: 10.1126/scitranslmed.abo5032<br>NCT03049488<br>Journal article	E4: Prefusion-stabilized DS-Cav1 vaccine immunology, rather than the RSVPreF3 OA product.
000495	Wright, P.F. et al.<br>Evaluation of a live, cold-passaged, temperature-sensitive, respiratory syncytial virus vaccine candidate in infancy<br>Journal of Infectious Diseases (2000); pp. 1331–1342<br>DOI: 10.1086/315859<br>Journal article	E4: Live-attenuated RSV vaccination in infants or children; outside the product and population scope.
000501	Phelps, K. et al.<br>Evaluation of immunological parameters for correlates of protection against live siv challenge in recombinant simian varicella virus-simian immunodeficiency virus (RSVV-SIV) vaccinated nonhuman primates<br>Journal of Medical Primatology (2011); pp. 268–269<br>DOI: 10.1111/j.1600-0684.2011.00491.x<br>Conference abstract	E2: Recombinant simian varicella/SIV vaccination and SIV challenge in nonhuman primates.

Record	Report and bibliographic identifier	Specific exclusion reason
000503	Gonzalez, IM et al.<br>Evaluation of the live attenuated cpts 248/404 RSV vaccine in combination with a subunit RSV vaccine (PFP-2) in healthy young and older adults<br>Vaccine (2000); pp. 1763–1772<br>DOI: 10.1016/s0264-410x(99)00527-7<br>Journal article	E4: Combination of live cpts248/404 RSV vaccine and PFP-2 subunit RSV vaccine; not RSVPreF3 OA with a partner vaccine.
000513	Richard, S.A.<br>Exploring the Pivotal Immunomodulatory and Anti-Inflammatory Potentials of Glycyrrhizic and Glycyrrhetinic Acids<br>Mediators of Inflammation (2021)<br>DOI: 10.1155/2021/6699560<br>Review	E1: Review of glycyrrhizic and glycyrrhetinic acids; not an original eligible RSV vaccine trial.
000514	Huynh, K et al.<br>Exploring the "Two-Hit" Phenomenon of Guillain-Barré Syndrome Following Respiratory Syncytial Virus and Influenza Vaccinations<br>Cureus Journal Of Medical Science (2024)<br>DOI: 10.7759/cureus.68899<br>Journal article	E3: Individual Guillain–Barré syndrome case report after RSV and influenza vaccination; no randomized comparison.
000525	Van Der Plas, J. et al.<br>First-in-human, randomized study to assess the safety, tolerability, immunogenicity, and shedding of a liveattenuated respiratory syncytial virus vaccine<br>British Journal of Clinical Pharmacology (2020); pp. 1189–1190<br>DOI: 10.1111/bcp.14266<br>Conference abstract	E4: Live-attenuated RSV vaccine study, rather than RSVPreF3 OA.
000538	Hause, A.M. et al.<br>Health events reported by US adults aged ≥60 years who received a respiratory syncytial virus vaccine alone or with other vaccines<br>Ssrn (2026)<br>DOI: 10.2139/ssrn.6556803<br>Preprint	E3: Voluntary v-safe survey of self-reported health events; observational exposure groups rather than randomized vaccine schedules.

Record	Report and bibliographic identifier	Specific exclusion reason
000544	Vera-Punzano, N. et al.<br>Hospitalisation due to respiratory syncytial virus in a population-based cohort of older adults in Spain, 2016/17 to 2019/20<br>Eurosurveillance (2025)<br>DOI: 10.2807/1560-7917.es.2025.30.10.2400364<br>Journal article	E3: Population-based cohort of RSV hospitalization in Spain; not a randomized vaccination-schedule trial.
000552	Burton, D.R. et al.<br>Human antibodies from combinatorial libraries<br>Advances in Immunology (1994); pp. 191–280<br>DOI: 10.1016/s0065-2776(08)60674-4<br>Review	E1: Review concerning combinatorial antibody libraries; not an original vaccine-schedule trial.
000554	Ulloa-Aguilar, J.M. et al.<br>Human respiratory syncytial virus regulates the expression of interferon-stimulated genes through modulation of fibrillarin<br>Frontiers in Cellular and Infection Microbiology (2026)<br>DOI: 10.3389/fcimb.2026.1706028<br>Journal article	E2: Laboratory investigation of fibrillarin and interferon-stimulated genes during RSV infection.
000555	Jozwik, A. et al.<br>Human T-cell responses to RSV infection in the lower airway<br>Immunology (2014); pp. 76<br>DOI: 10.1111/imm.12406<br>Conference abstract	E5: Experimental RSV infection and lower-airway T-cell responses; no partner-vaccine schedule comparison.
000569	Ferguson, M. et al.<br>Immune persistence and safety of the AS01E-adjuvanted respiratory syncytial virus prefusion F protein-based vaccine in adults 50–59 years of age, including at-increased-risk adults: A randomized controlled trial<br>Human Vaccines and Immunotherapeutics (2025)<br>DOI: 10.1080/21645515.2025.2579335<br>NCT05590403<br>Journal article	E5: RSVPreF3 OA immune persistence and age-based immunobridging study; no partner-vaccine coadministration versus sequential comparison.

Record	Report and bibliographic identifier	Specific exclusion reason
000571	Murphy, B.R. et al.<br>Immunization of cotton rats with the fusion (F) and large (G) glycoproteins of respiratory syncytial virus (RSV) protects against RSV challenge without potentiating RSV disease<br>Vaccine (1989); pp. 533–540<br>DOI: 10.1016/0264-410x(89)90278-8<br>Journal article	E2: Cotton-rat immunization with RSV F and G glycoproteins and viral challenge.
000575	Davis, M et al.<br>Immunobridging Demonstrating Effectiveness of the Bivalent Respiratory Syncytial Virus (RSV) Prefusion F Subunit Vaccine in Adults 18-59 Years of Age at High Risk of Severe RSV Disease in a Phase 3 Trial: the C3671023 MONeT Study Results<br>Open forum infectious diseases (2025); pp. S462–S463<br>DOI: 10.1093/ofid/ofae631.794<br>NCT05842967<br>Conference abstract	E4: Pfizer bivalent RSVpreF immunobridging in adults aged 18–59 years; outside the RSVPreF3 OA product scope.
000577	Pringle, C.R. et al.<br>Immunogenciity and pathogenicity of a triple temperature-sensitive modified respiratory syncytial virus in adult volunteers<br>Vaccine (1993); pp. 473–478<br>DOI: 10.1016/0264-410x(93)90290-e<br>Journal article	E4: Experimental temperature-sensitive live RSV vaccines and challenge, rather than RSVPreF3 OA.
000579	McKay, E. et al.<br>Immunogenicity and pathogenicity of temperature-sensitive modified respiratory syncytial virus in adult volunteers<br>Journal of Medical Virology (1988); pp. 411–421<br>DOI: 10.1002/jmv.1890250405<br>Journal article	E4: Experimental temperature-sensitive live RSV vaccines and challenge, rather than RSVPreF3 OA.
000580	Hannaford, A. et al.<br>Immunogenicity and Reactogenicity of Adjuvanted (Arexvy) and Non-Adjuvanted (Abrysvo) RSV Vaccines in Immunocompromised Individuals with Hematological Malignancies<br>Open Forum Infectious Diseases (2026); pp. S905–S906<br>DOI: 10.1093/ofid/ofaf695.1609<br>Conference abstract	E3: Immunocompromised-patient and healthy-control vaccine-response cohorts; no randomized coadministration versus sequential comparison.

Record	Report and bibliographic identifier	Specific exclusion reason
000583	Fries, L. et al.<br>Immunogenicity and safety of a respiratory syncytial virus fusion protein (RSV F) nanoparticle vaccine in older adults<br>Immunity and Ageing (2017)<br>DOI: 10.1186/s12979-017-0090-7<br>NCT01709019<br>Journal article	E4: RSV F protein nanoparticle vaccine, rather than adjuvanted RSVPreF3 OA.
000584	Hosman, T. et al.<br>Immunogenicity and safety of Ad26.RSV.preF/RSV preF protein vaccine at predicted intermediate- and end-of-shelf-life as an evaluation of potency throughout shelf life<br>Human Vaccines and Immunotherapeutics (2024)<br>DOI: 10.1080/21645515.2024.2344970<br>NCT05101486<br>Journal article	E4: Ad26.RSV.preF, alone or combined with RSV preF protein, is outside the RSVPreF3 OA product scope.
000589	Immunogenicity and Safety of Multiple-Dose Adjuvanted RSVPreF3 (Arexvy®) Vaccination Among Immunocompromised Persons<br>ClinicalTrials.gov (2025)<br>NCT07050732<br>ClinicalTrials.gov registry report	E5: One-dose versus two-dose Arexvy vaccination and boosting in immunocompromised persons; no partner-vaccine schedule comparison.
000629	Suresh, G. et al.<br>IMpact-RSV study group report [1] (multiple letters)<br>Pediatrics (1999); pp. 993–995<br>DOI: 10.1542/peds.104.4.993<br>Letters and comments	E1: Letters commenting on the IMpact-RSV palivizumab study; not an original RSVPreF3 OA trial report.
000630	Awar, M. et al.<br>In older adults, an AS01E-adjuvanted RSVPreF3 OA vaccine reduced RSV-related lower respiratory tract disease<br>Annals of Internal Medicine (2023); pp. JC62–JC63<br>DOI: 10.7326/j23-0038<br>ACP Journal Club summary	E1: ACP Journal Club evidence summary of RSV vaccine effectiveness; not an original vaccination-schedule trial report.

Record	Report and bibliographic identifier	Specific exclusion reason
000631	Sacks, H.S.<br>In older adults, RSV prefusion F vaccine reduced hospitalization for RSV-related respiratory tract disease vs. no vaccine<br>Annals of Internal Medicine (2026); pp. JC4<br>DOI: 10.7326/annals-25-05113-jc<br>ACP Journal Club summary	E1: ACP Journal Club evidence summary of RSV vaccine effectiveness; not an original vaccination-schedule trial report.
000645	Gallwitz, B. et al.<br>Indication-based vaccinations for people with diabetes mellitus<br>Diabetologie (2025); pp. 758–767<br>DOI: 10.1007/s11428-025-01379-x<br>Recommendations	E1: Vaccination recommendations for people with diabetes; not an original randomized trial report.
000646	Covián, C. et al.<br>Induction of Trained Immunity by Recombinant Vaccines<br>Frontiers in Immunology (2021)<br>DOI: 10.3389/fimmu.2020.611946<br>Review	E1: Review of recombinant vaccines and trained immunity; not an original eligible schedule trial.
000658	Woźniak-Kosek, A. et al.<br>[Influenza in Poland in 2010].<br>Przegląd epidemiologiczny (2012); pp. 599–604<br>Journal article	E3: National influenza surveillance report for Poland; not an RSV vaccination-schedule trial.
000663	Mihălțan, F.-D. et al.<br>Innovative approaches in RSV prevention: The expanding role of monoclonal antibodies in protection for all infants<br>Germs (2025); pp. 279–282<br>DOI: 10.18683/germs.2025.1475<br>Review	E1: Overview of monoclonal-antibody prevention in infants; not an original RSVPreF3 OA trial report.
000680	Cunningham, C.K. et al.<br>Live-attenuated respiratory syncytial virus vaccine with deletion of RNA synthesis regulatory protein M2-2 and cold passage mutations is overattenuated<br>Open Forum Infectious Diseases (2019)<br>DOI: 10.1093/ofid/ofz212<br>NCT02890381; NCT02948127<br>Journal article	E4: Live-attenuated RSV vaccination in infants or children; outside the product and population scope.

Record	Report and bibliographic identifier	Specific exclusion reason
000684	Ascough, S. et al.<br>Local and Systemic Immunity against Respiratory Syncytial Virus Induced by a Novel Intranasal Vaccine A Randomized, Double-Blind, Placebo-controlled Clinical Trial<br>American Journal of Respiratory and Critical Care Medicine (2019); pp. 481–492<br>DOI: 10.1164/rccm.201810-1921oc<br>NCT02958540<br>Journal article	E4: Intranasal SynGEM vaccine, rather than RSVPreF3 OA.
000691	Omelyanovskiy, V.V. et al.<br>Main changes in the model of diagnosis-related groups in 2022<br>Medical Technologies. Assessment and Choice (2022); pp. 8–21<br>DOI: 10.17116/medtech2022440218<br>Policy and reimbursement discussion	E1: Discussion of changes to diagnosis-related group reimbursement models; not an original vaccine trial.
000693	Mills, J.<br>Management of respiratory syncytial virus infections<br>Advances in Experimental Medicine and Biology (1996); pp. 163–174<br>DOI: 10.1007/978-1-4757-9209-6_17<br>Review and conference paper	E1: Review of RSV infection management, indexed as a conference paper; not an original eligible trial report.
000713	Gagneux-Brunon, A. et al.<br>Midwives' attitudes toward participation of pregnant individuals in a preventive vaccine hypothetical clinical trial<br>Vaccine (2022); pp. 5459–5463<br>DOI: 10.1016/j.vaccine.2022.07.049<br>Journal article	E3: Survey of midwives about a hypothetical maternal vaccine trial; no trial participants randomized to vaccination schedules.
000722	Sun, Y. et al.<br>mRNA-Based Respiratory Syncytial Virus Vaccine mRNA-1345 Induces Robust, Polyfunctional, and Durable CD4+ and CD8+ T Cell Responses with Similar Features in Adults Across Age Groups<br>Open Forum Infectious Diseases (2026); pp. S926<br>DOI: 10.1093/ofid/ofaf695.1640<br>NCT05397223<br>Conference abstract	E4: mRNA-1345 RSV vaccine study, rather than RSVPreF3 OA.

Record	Report and bibliographic identifier	Specific exclusion reason
000747	Eyles, J.E. et al.<br>Nonreplicating vaccines can protect african green monkeys from the memphis 37 strain of respiratory syncytial virus<br>Journal of Infectious Diseases (2013); pp. 319–329<br>DOI: 10.1093/infdis/jit169<br>Journal article	E2: RSV vaccination and challenge in African green monkeys.
000748	Green, Christopher A et al.<br>Novel genetically-modified chimpanzee adenovirus and MVA-vectored respiratory syncytial virus vaccine safely boosts humoral and cellular immunity in healthy older adults.<br>The Journal of infection (2019); pp. 382–392<br>DOI: 10.1016/j.jinf.2019.02.003<br>Journal article	E4: PanAd3-RSV and MVA-RSV vectored vaccination, rather than RSVPreF3 OA.
000750	Covert, K. et al.<br>Novel Maintenance Therapies for Chronic Obstructive Pulmonary Disorder<br>Annals of Pharmacotherapy (2025)<br>DOI: 10.1177/10600280251382202<br>Review	E1: Review of COPD maintenance therapies and recommendations; not an original coadministration trial.
000753	Open-label, Multicenter Trial of RSV Vaccination to Reduce Moderate-to-severe Exacerbations in COPD Frequent Exacerbators: Clinical Effectiveness and RSV-specific Immune Responses<br>ClinicalTrials.gov (2026)<br>NCT07508644<br>ClinicalTrials.gov registry report	E5: RSV vaccination plus standard care versus standard care in COPD; no assigned same-pair sequential vaccine comparator.
000754	Open Label, Single Arm Phase 1 Study to Evaluate the Shedding, Biodistribution, Safety and Immunogenicity of Ad26.RSV.preF Vaccine in Adults<br>ClinicalTrials.gov (2019)<br>NCT03795441<br>ClinicalTrials.gov registry report	E4: Ad26.RSV.preF, alone or combined with RSV preF protein, is outside the RSVPreF3 OA product scope.

Record	Report and bibliographic identifier	Specific exclusion reason
000782	Schmoele-Thoma, B et al.<br>Phase 1/2, first-in-human study of the safety, tolerability, and immunogenicity of an RSV prefusion F-based subunit vaccine candidate<br>Open forum infectious diseases (2019); pp. S970<br>DOI: 10.1093/ofid/ofz360.2432<br>NCT03529773<br>Conference abstract	E4: Pfizer bivalent RSVpreF vaccine, rather than adjuvanted RSVPreF3 OA.
000783	Falsey, A.R. et al.<br>Phase 1/2 Randomized Study of the Immunogenicity, Safety, and Tolerability of a Respiratory Syncytial Virus Prefusion F Vaccine in Adults with Concomitant Inactivated Influenza Vaccine<br>Journal of Infectious Diseases (2022); pp. 2056–2066<br>DOI: 10.1093/infdis/jiab611<br>NCT03529773<br>Journal article	E4: Pfizer bivalent RSVpreF vaccine, rather than adjuvanted RSVPreF3 OA.
000784	Phase 1, Open-Label, Dose-Escalation Study to Evaluate Tolerability, Safety, and Immunogenicity of an Intranasal Live Attenuated Respiratory Syncytial Virus Vaccine Expressing Spike Protein of SARS-CoV-2 in Healthy Adults Ages 18 - 69 Years<br>ClinicalTrials.gov (2021)<br>NCT04798001<br>ClinicalTrials.gov registry report	E4: Intranasal live-attenuated RSV vector expressing SARS-CoV-2 spike, rather than RSVPreF3 OA.
000789	Phase 3 clinical trial results for nirsevimab: Is it time to consider population-level RSV immunoprophylaxis?<br>Journal of Paediatrics and Child Health (2022); pp. 1117–1118<br>DOI: 10.1111/jpc.15999<br>Commentary	E1: Comment on a nirsevimab trial and infant immunoprophylaxis; not an original eligible RSV vaccine trial report.
000791	Chime, N. et al.<br>Phase 3 Study Assessing Lot-to-Lot Consistency of Respiratory Syncytial Virus Prefusion Protein F3 Vaccine and Its Immune Response, Safety, and Reactogenicity When Co-administered With Quadrivalent Influenza Vaccine<br>Journal of Infectious Diseases (2025); pp. e144–e153<br>DOI: 10.1093/infdis/jiae342<br>NCT05045144<br>Journal article	E4: Unadjuvanted RSVPreF3 maternal vaccine in nonpregnant women aged 18–49 years; not the adjuvanted RSVPreF3 OA product.

Record	Report and bibliographic identifier	Specific exclusion reason
000804	Muder, R.R.<br>Pneumonia in residents of long-term care facilities: Epidemiology, etiology, management, and prevention<br>American Journal of Medicine (1998); pp. 319–330<br>DOI: 10.1016/s0002-9343(98)00262-9<br>Review	E1: Review of pneumonia epidemiology, management and prevention in long-term care; not an original eligible trial.
000810	Fust, K. et al.<br>Potential Clinical Impact of Respiratory Syncytial Virus (RSV) Vaccination in Older Adults in the United States<br>Open Forum Infectious Diseases (2022); pp. S107<br>DOI: 10.1093/ofid/ofac492.194<br>Conference abstract	E3: Decision-analytic model of hypothetical RSV vaccination impact; not a randomized trial.
000814	Laurichesse, H. et al.<br>Pre- and in-hospital management of community-acquired pneumonia in Southern France, 1998-99<br>European Journal of Clinical Microbiology and Infectious Diseases (2001); pp. 770–778<br>DOI: 10.1007/s100960100613<br>Journal article	E3: Prospective observational study of community-acquired pneumonia management in southern France; not a vaccine trial.
000815	Fuentes, S. et al.<br>Preclinical evaluation of bacterially produced RSV-G protein vaccine: Strong protection against RSV challenge in cotton rat model<br>Scientific reports (2017); pp. 42428<br>DOI: 10.1038/srep42428<br>Journal article	E2: Bacterially produced RSV G-protein vaccination and challenge in cotton rats.
000828	Babawale, P.I. et al.<br>Preventing RSV Infection in Children: Current Passive Immunizations and Vaccine Development<br>Pathogens (2025)<br>DOI: 10.3390/pathogens14020104<br>Review	E1: Review of pediatric passive immunization and RSV vaccine development; not an original eligible trial.
000842	Barrella Harboe, Z. et al.<br>Prioritising at-risk adults in vaccine coadministration trials<br>The Lancet Infectious Diseases (2025); pp. 362–363<br>DOI: 10.1016/s1473-3099(24)00674-1<br>Commentary	E1: Comment on the Goswami mRNA-1345 coadministration trial; no independent trial dataset.

Record	Report and bibliographic identifier	Specific exclusion reason
000846	Abbasi, A. et al.<br>Propagation Properties of a New Human Diploid Cell Line, RAZI-HDC, and Its Suitability as a Candidate Cell Substrate for Respiratory Syncytial Virus Vaccine Production in Comparison to MRC-5<br>Archives of Razi Institute (2025); pp. 51–59<br>DOI: 10.32592/ari.2025.80.1.51<br>Journal article	E2: Development of a human diploid cell substrate for vaccine production; not a clinical vaccination-schedule trial.
000854	Riaz, R. et al.<br>Protecting our future: call for respiratory syncytial virus vaccine trial in infants<br>Annals of Medicine and Surgery (2024); pp. 4960–4962<br>DOI: 10.1097/ms9.0000000000002385<br>Editorial	E1: Editorial calling for infant RSV vaccine trials; not a report of an original eligible trial.
000882	Kuitunen, I. et al.<br>Rapid review showed that real-world results on how nirsevimab prevented respiratory syncytial virus hospitalisations were similar to randomised trials<br>Acta Paediatrica, International Journal of Paediatrics (2024); pp. 1940–1941<br>DOI: 10.1111/apa.17301<br>Rapid review	E1: Rapid review of nirsevimab effectiveness; not an original RSVPreF3 OA trial report.
000908	Walsh, E.E. et al.<br>RENOIR Trial — RSVpreF Vaccine Efficacy over Two Seasons: To the Editor<br>New England Journal of Medicine (2024); pp. 1459–1460<br>DOI: 10.1056/nejmc2311560<br>Letter	E1: Letter concerning RENOIR vaccine efficacy; not an original eligible coadministration trial report.
000909	Stokes, A.H. et al.<br>Repeated Dose Toxicity Study and Developmental and Reproductive Toxicology Studies of a Respiratory Syncytial Virus Candidate Vaccine in Rabbits and Rats<br>International Journal of Toxicology (2021); pp. 125–142<br>DOI: 10.1177/1091581820985782<br>Journal article	E2: Repeated-dose and reproductive toxicology studies in rabbits and rats.
000914	Lokteff, M. et al.<br>Residual DNA quantification in clinical batches of BBG2Na, a recombinant subunit vaccine against human respiratory syncytial virus<br>Biologicals (2001); pp. 123–132<br>DOI: 10.1006/biol.2001.0283<br>Journal article	E2: Residual-DNA quantification in BBG2Na vaccine manufacturing batches; not a clinical schedule comparison.

Record	Report and bibliographic identifier	Specific exclusion reason
000918	Chávez-Bueno, S. et al.<br>Respiratory syncytial virus bronchiolitis: Current and future strategies for treatment and prophylaxis<br>Treatments in Respiratory Medicine (2006); pp. 483–494<br>DOI: 10.2165/00151829-200605060-00011<br>Review	E1: Review of RSV bronchiolitis treatment and prophylaxis; not an original eligible trial report.
000924	Berni Canani, M. et al.<br>Respiratory syncytial virus infection<br>Rivista Italiana di Pediatria (1998); pp. 617–621<br>Conference paper	E1: Italian conference paper on RSV infection in a pediatric journal; not independently eligible under the current report-format criteria.
000932	Bigoni, T. et al.<br>Respiratory Syncytial Virus Prevention in the Adult Population: State of the Art<br>Seminars in Respiratory and Critical Care Medicine (2025); pp. 41–52<br>DOI: 10.1055/a-2586-3974<br>Review	E1: Review of RSV prevention strategies; not an original randomized coadministration trial report.
000933	Greenough, A. et al.<br>Respiratory syncytial virus prevention: past and present strategies.<br>Expert opinion on pharmacotherapy (2000); pp. 1195–1201<br>Review	E1: Review of RSV prevention strategies; not an original randomized coadministration trial report.
000936	Branche, A.R. et al.<br>Respiratory Syncytial Virus Risk Assessment in Adults: Implications for Vaccine Recommendations Beyond Age<br>Open Forum Infectious Diseases (2025)<br>DOI: 10.1093/ofid/ofaf394<br>Journal article	E3: Observational assessment of RSV hospitalization risk factors; not a randomized vaccination-schedule trial.
000943	Niu, X. et al.<br>Respiratory Syncytial Virus (RSV) Prophylaxis: Clesrovimab and Beyond<br>Journal of Medicinal Chemistry (2026); pp. 4–9<br>DOI: 10.1021/acs.jmedchem.5c03667<br>Editorial	E1: Editorial on clesrovimab and RSV prophylaxis; not an original eligible vaccine trial report.

Record	Report and bibliographic identifier	Specific exclusion reason
000951	Welliver, R.C. et al.<br>Respiratory syncytial virus-specific cell-mediated immune responses after vaccination with a purified fusion protein subunit vaccine<br>Journal of Infectious Diseases (1994); pp. 425–428<br>Journal article	E4: PFP-2 subunit RSV vaccine in children aged 12–18 months; outside the product and population scope.
000952	Zeme, M. et al.<br>Respiratory Syncytial Virus Vaccination Acceptance: The Importance of Asking "Why?"<br>Journal of Pediatrics: Clinical Practice (2025)<br>DOI: 10.1016/j.jpedcp.2025.200163<br>Commentary	E1: Commentary on reasons for vaccine acceptance; not an original randomized schedule trial report.
000955	Alfaro, T et al.<br>Respiratory syncytial virus vaccination in older adults and patients with chronic disorders: A position paper from the Portuguese Society of Pulmonology, the Portuguese Association of General and Family Medicine, the Portuguese Society of Cardiology, the Portuguese Society of Infectious Diseases and Clinical Microbiology, the Portuguese Society of Endocrinology, Diabetes and Metabolism, and the Portuguese Society of Internal Medicine<br>Pulmonology (2025); pp. 2451456<br>DOI: 10.1080/25310429.2025.2451456<br>Position paper	E1: Professional-society position paper on RSV vaccination; not an original randomized trial report.
000958	Mayer, E.F. et al.<br>Respiratory Syncytial Virus Vaccination in Solid Organ Transplant Recipients: Interim Findings From a Phase 3 Trial of mRNA-1345<br>Clinical Infectious Diseases (2026); pp. e1112–e1121<br>DOI: 10.1093/cid/ciag108<br>NCT06067230<br>Journal article	E4: mRNA-1345 RSV vaccine study, rather than RSVPreF3 OA.
000964	Meinrenken, S.<br>Respiratory syncytial virus vaccine is successful in phase 2a trial<br>Pneumologie (2022); pp. 849–850<br>DOI: 10.1055/a-1947-3783<br>Research news	E1: Research news note summarizing a phase 2a RSV vaccine study; not an original trial report.

Record	Report and bibliographic identifier	Specific exclusion reason
000969	Fulginiti, V.A. et al.<br>Respiratory virus immunization. I. A field trial of two inactivated respiratory virus vaccines; an aqueous trivalent parainfluenza virus vaccine and an alum-precipitated respiratory syncytial virus vaccine.<br>American journal of epidemiology (1969); pp. 435–448<br>Journal article	E4: Historical inactivated parainfluenza and alum-precipitated RSV vaccines, rather than RSVPreF3 OA.
000971	Douglas Jr., R.G.<br>Respiratory virus vaccines: recent advances<br>Amer.Soc.Microbiol. (1975); pp. 409–415<br>Journal article	E1: Review of respiratory-virus vaccine development; not an original eligible trial report.
000988	Lenney, W.<br>RSV disease-Is there a role for prevention?<br>Respiratory Medicine (2001); pp. 170–172<br>DOI: 10.1053/rmed.2000.1018<br>Editorial	E1: Editorial on RSV disease prevention; not an original eligible randomized trial report.
000993	Terstappen, J. et al.<br>RSV neutralizing antibodies in dried blood<br>medRxiv (2023)<br>DOI: 10.1101/2023.08.10.23293875<br>Preprint	E2: Development and evaluation of an RSV neutralizing-antibody assay using dried blood, rather than a randomized vaccine-schedule comparison.
000994	Terstappen, J. et al.<br>RSV Neutralizing Antibodies in Dried Blood<br>Journal of Infectious Diseases (2024); pp. e93–e101<br>DOI: 10.1093/infdis/jiad543<br>Journal article	E2: Development and evaluation of an RSV neutralizing-antibody assay using dried blood, rather than a randomized vaccine-schedule comparison.
001000	Jairam, N. et al.<br>RSV Vaccination Increases CD4 T-Cell Response, but Not CD8, in Solid Organ Transplant Recipients<br>American Journal of Transplantation (2025); pp. S807–S808<br>DOI: 10.1016/j.ajt.2025.07.1909<br>Conference abstract	E3: Prospective T-cell-response study in solid organ transplant vaccine recipients; no randomized coadministration versus sequential comparison.

Record	Report and bibliographic identifier	Specific exclusion reason
001002	[Anonymous]<br>RSV vaccine candidate immunogenic in older people<br>Human Vaccines & Immunotherapeutics (2017); pp. 1958–1958<br>DOI: 10.1080/21645515.2017.1374796<br>Research news	E1: RSV vaccine news subsection; not an original randomized trial report.
001019	Power, U.F. et al.<br>Safety and immunogenicity of a novel recombinant subunit respiratory syncytial virus vaccine (BBG2Na) in healthy young adults<br>Journal of Infectious Diseases (2001); pp. 1456–1460<br>DOI: 10.1086/324426<br>Journal article	E4: Recombinant BBG2Na RSV vaccine, rather than RSVPreF3 OA.
001028	Comeaux, C et al.<br>Safety and immunogenicity of a seasonal influenza vaccine and AD26.RSV.pref vaccine with and without co-administration: a randomized, double-blind, placebo-controlled phase 2A study in adults aged ≥ 60 years<br>Open forum infectious diseases (2019); pp. S979<br>DOI: 10.1093/ofid/ofz360.2452<br>NCT03339713<br>Conference abstract	E4: Ad26.RSV.preF, alone or combined with RSV preF protein, is outside the RSVPreF3 OA product scope.
001029	Glenn, G.M. et al.<br>Safety and immunogenicity of a Sf9 insect cell-derived respiratory syncytial virus fusion protein nanoparticle vaccine<br>Vaccine (2013); pp. 524–532<br>DOI: 10.1016/j.vaccine.2012.11.009<br>NCT01290419<br>Journal article	E4: RSV F protein nanoparticle vaccine, rather than adjuvanted RSVPreF3 OA.
001030	Hall, V.G. et al.<br>Safety and immunogenicity of adjuvanted respiratory syncytial virus vaccine in high-risk transplant recipients: an interventional cohort study<br>Clinical Microbiology and Infection (2026); pp. 161–168<br>DOI: 10.1016/j.cmi.2025.09.013<br>NCT06593210<br>Journal article	E3: Interventional transplant-recipient cohort; no randomized coadministration versus sequential comparison.

Record	Report and bibliographic identifier	Specific exclusion reason
001034	Athan, Eugene et al.<br>Safety and Immunogenicity of Bivalent RSVpreF Vaccine Coadministered With Seasonal Inactivated Influenza Vaccine in Older Adults.<br>Clinical infectious diseases : an official publication of the Infectious Diseases Society of America (2024); pp. 1360–1368<br>DOI: 10.1093/cid/ciad707<br>NCT05301322<br>Journal article	E4: Pfizer bivalent RSVpreF vaccine, rather than adjuvanted RSVPreF3 OA.
001035	Sarwar, U.N. et al.<br>Safety and immunogenicity of bivalent RSVpreF vaccine formulated in a multidose vial in healthy female participants in the USA: a multicentre, randomised, open-label, noninferiority phase 3 study<br>eClinicalMedicine (2026)<br>DOI: 10.1016/j.eclinm.2026.103767<br>NCT06473519<br>Journal article	E4: Bivalent RSVpreF multidose-vial formulation study, rather than RSVPreF3 OA.
001036	Neutel, J et al.<br>Safety and Immunogenicity of Coadministered Bivalent BNT162b2 COVID-19 Vaccine and Bivalent RSVpreF Respiratory Syncytial Virus Vaccine With and Without Quadrivalent Influenza Vaccine in Adults ≥ 65 Years of Age<br>Open forum infectious diseases (2025); pp. S111–S112<br>DOI: 10.1093/ofid/ofae631.172<br>NCT05886777<br>Conference abstract	E4: Pfizer bivalent RSVpreF and BNT162b2 formulations, rather than RSVPreF3 OA.
001037	Neutel, J.M. et al.<br>Safety and Immunogenicity of Concomitant Administration and Combined Administration of Bivalent BNT162b2 COVID-19 Vaccine and Bivalent RSVpreF Respiratory Syncytial Virus Vaccine with or Without Quadrivalent Influenza Vaccine in Adults ≥ 65 Years of Age<br>Vaccines (2025)<br>DOI: 10.3390/vaccines13020158<br>NCT05886777<br>Journal article	E4: Pfizer bivalent RSVpreF and BNT162b2 formulations, rather than RSVPreF3 OA.

Record	Report and bibliographic identifier	Specific exclusion reason
001038	Study Investigators<br>Safety and immunogenicity of mRNA-1345 RSV vaccine coadministered with an influenza or COVID-19 vaccine in adults aged 50 years or older: an observer-blinded, placebo controlled, randomised, phase 3 trial<br>Lancet Infectious Diseases (2025); pp. 411–423<br>DOI: 10.1016/s1473-3099(24)00589-9<br>NCT05330975<br>Journal article	E4: mRNA-1345 RSV vaccine, rather than RSVPreF3 OA; coadministration results are outside the current product scope.
001048	Sadoff, J. et al.<br>Safety and Immunogenicity of the Ad26.RSV.preF Investigational Vaccine Coadministered with an Influenza Vaccine in Older Adults<br>Journal of Infectious Diseases (2021); pp. 699–708<br>DOI: 10.1093/infdis/jiaa409<br>NCT03339713<br>Journal article	E4: Ad26.RSV.preF, alone or combined with RSV preF protein, is outside the RSVPreF3 OA product scope.
001049	Piedra, P.A. et al.<br>Safety and immunogenicity of the PFP vaccine against respiratory syncytial virus (RSV): The Western blot assay aids in distinguishing immune responses of the PFP vaccine from RSV infection<br>Vaccine (1995); pp. 1095–1101<br>DOI: 10.1016/0264-410x(95)00034-x<br>Journal article	E4: PFP-1 RSV vaccine in children with influenza vaccine as the comparator; outside the product, population and schedule scope.
001062	Shaw, C.A. et al.<br>Safety, Tolerability, and Immunogenicity of an mRNA-Based Respiratory Syncytial Virus Vaccine in Healthy Young Adults in a Phase 1 Clinical Trial<br>Journal of Infectious Diseases (2024); pp. e637–e646<br>DOI: 10.1093/infdis/jiae035<br>NCT04528719<br>Journal article	E4: mRNA-1345 RSV vaccine study, rather than RSVPreF3 OA.
001067	Mayer, EF et al.<br>Safety, Tolerability, and Immunogenicity of the mRNA-1345 RSV Vaccine in Solid Organ Transplant Recipients Aged ≥18 Years<br>Open forum infectious diseases (2026); pp. S55<br>DOI: 10.1093/ofid/ofaf695.081<br>NCT06067230<br>Conference abstract	E4: mRNA-1345 RSV vaccine study, rather than RSVPreF3 OA.

Record	Report and bibliographic identifier	Specific exclusion reason
001076	Seroconversion Following RSV Vaccination in Bone Marrow Transplant and CAR-T Patients<br>ClinicalTrials.gov (2025)<br>NCT07296120<br>ClinicalTrials.gov registry report	E4: Abrysvo vaccination in hematopoietic transplant or CAR-T recipients; outside the RSVPreF3 OA product scope.
001077	Arankalle, Vidya A et al.<br>Seroepidemiology of respiratory syncytial virus in western India with special reference to appropriate age for infant vaccination.<br>Journal of medical virology (2019)<br>DOI: 10.1002/jmv.25489<br>Journal article	E3: RSV seroepidemiology in children in western India; not a vaccination-schedule trial.
001087	Fierro, C. et al.<br>Shared Clinical and Immunologic Features of mRNA Vaccines: Preliminary Results from a Comparative Clinical Study<br>medRxiv (2024)<br>DOI: 10.1101/2024.11.26.24318005<br>NCT05397223<br>Preprint	E4: mRNA-1345 RSV vaccine study, rather than RSVPreF3 OA.
001088	Fierro, C. et al.<br>Shared clinical and immunologic features of mRNA vaccines: preliminary results from a comparative clinical study<br>Frontiers in Immunology (2025)<br>DOI: 10.3389/fimmu.2025.1501275<br>NCT05397223<br>Journal article	E4: mRNA-1345 RSV vaccine study, rather than RSVPreF3 OA.
001089	Nguyen, T. et al.<br>Short term safety profile of respiratory syncytial virus vaccine in adults aged ≥ 60 years in Australia<br>The Lancet Regional Health - Western Pacific (2025)<br>DOI: 10.1016/j.lanwpc.2025.101506<br>Comment reporting surveillance data	E3: AusVaxSafety active postmarketing surveillance of reported adverse events; no randomized vaccination-schedule comparison.

Record	Report and bibliographic identifier	Specific exclusion reason
001090	Yamaji, Y. et al.<br>Simultaneous administration of recombinant measles viruses expressing respiratory syncytial virus fusion (F) and nucleo (N) proteins induced humoral and cellular immune responses in cotton rats<br>Vaccines (2019)<br>DOI: 10.3390/vaccines7010027<br>Journal article	E2: Recombinant measles vectors expressing RSV proteins evaluated in a cotton-rat experiment.
001092	Biegus, J. et al.<br>Simultaneous vaccination against influenza and respiratory syncytial virus in high-risk heart failure patients<br>ESC Heart Failure (2025); pp. 3869–3877<br>DOI: 10.1002/ehf2.15432<br>NCT06259487<br>Journal article	E5: Coordinated RSV and influenza vaccination versus standard-care recommendations in heart failure; the comparator does not assign the same vaccine pair sequentially.
001097	Flynn, J.A. et al.<br>Stability characterization of a vaccine antigen based on the respiratory syncytial virus fusion glycoprotein<br>PLoS ONE (2016)<br>DOI: 10.1371/journal.pone.0164789<br>Journal article	E2: Biophysical stability characterization of an RSV antigen; not a clinical vaccination-schedule trial.
001100	Study on the Impact of Coordinated Immunizations Against RSV and Influenza on the Prognosis in Patients With Chronic Heart Failure.<br>ClinicalTrials.gov (2024)<br>NCT06259487<br>ClinicalTrials.gov registry report	E5: Coordinated RSV and influenza vaccination versus standard-care recommendations in heart failure; the comparator does not assign the same vaccine pair sequentially.
001104	Ellison III, R.T.<br>Surgical masks or N95 respirators for the flu?<br>Medicine Today (2009); pp. 69–70<br>Research commentary	E4: Surgical masks versus N95 respirators, rather than an RSV vaccination intervention.

Record	Report and bibliographic identifier	Specific exclusion reason
001112	Biegus, J. et al.<br>The early safety profile of simultaneous vaccination against influenza and Respiratory Syncytial Virus (RSV) in patients with high-risk heart failure<br>Vaccine (2024); pp. 2937–2940<br>DOI: 10.1016/j.vaccine.2024.03.060<br>NCT06259487<br>Journal article	E5: Early single-arm safety report of same-day RSV and influenza vaccination in heart failure; no sequential comparator.
001120	Zhang, Lei et al.<br>The global trends and clinical progress in influenza co-infection: a visualization and bibliometric analysis (2005-2025).<br>Frontiers in microbiology (2025)<br>DOI: 10.3389/fmicb.2025.1658752<br>Bibliometric analysis	E1: Bibliometric analysis of influenza coinfection research; not an individual eligible randomized trial.
001133	Wright, P.F. et al.<br>The interferon antagonist NS2 protein of respiratory syncytial virus is an important virulence determinant for humans<br>Journal of Infectious Diseases (2006); pp. 573–581<br>DOI: 10.1086/499600<br>Journal article	E4: NS2-deleted live RSV vaccine candidates, rather than RSVPreF3 OA.
001137	Zhu, T. et al.<br>The preventive effect of vaccine prophylaxis on severe respiratory syncytial virus infection: A meta-analysis<br>Virologica Sinica (2015); pp. 371–378<br>DOI: 10.1007/s12250-015-3630-3<br>Meta-analysis	E1: Meta-analysis of RSV vaccine prophylaxis; not an original randomized trial report.
001143	Sacconnay, L. et al.<br>The RSVPreF3-AS01 vaccine elicits broad neutralization of contemporary and antigenically distant respiratory syncytial virus strains<br>Science Translational Medicine (2023)<br>DOI: 10.1126/scitranslmed.adg6050<br>NCT03814590<br>Journal article	E5: RSVPreF3-AS01 neutralization-breadth analysis across RSV strains; no partner-vaccine schedule comparison.

Record	Report and bibliographic identifier	Specific exclusion reason
001147	The WHO Vaccine Trial Registry<br>European trial registry (2001); pp. 31–41<br>DOI: 10.1016/s0264-410x(01)00261-4<br>Registry methods review	E1: Descriptive report on the WHO Vaccine Trial Registry; not an individual eligible trial report.
001148	Francica, J.R. et al.<br>Thermoresponsive Polymer Nanoparticles Co-deliver RSV F Trimers with a TLR-7/8 Adjuvant<br>Bioconjugate Chemistry (2016); pp. 2372–2385<br>DOI: 10.1021/acs.bioconjchem.6b00370<br>Journal article	E2: Preclinical RSV F-trimer and TLR-7/8-adjuvant nanoparticle study; not clinical coadministration of two vaccines.
001159	Williams, G.P. et al.<br>Unaltered T cell responses to common antigens in individuals with Parkinson's disease<br>Journal of the Neurological Sciences (2023)<br>DOI: 10.1016/j.jns.2022.120510<br>Journal article	E3: Case-control comparison of antigen-specific T-cell responses in Parkinson disease; not a vaccination-schedule trial.
001172	Lequien, P.<br>Vaccination and extreme prematurity<br>Archives de Pediatrie (2002); pp. 431s–434s<br>Journal article	E4: Vaccination in extremely premature infants, outside the adult RSVPreF3 OA population.
001174	Luijckx, A.M.C. et al.<br>Vaccination of hospital staff against hepatitis B; the intramuscular method vs combined intradermal/intramuscular method<br>Nederlands Tijdschrift voor Geneeskunde (1985); pp. 2163–2166<br>Journal article	E4: Hepatitis B vaccination routes in hospital staff, rather than RSVPreF3 OA coadministration.
001176	Vaccination Response in Individual Monozygotic Twins<br>ClinicalTrials.gov (2014)<br>NCT02296359<br>ClinicalTrials.gov registry report	E4: Influenza-vaccine responses in monozygotic twins, rather than an RSVPreF3 OA vaccination-schedule comparison.
001179	McCool, RS et al.<br>Vaccination with prefusion-stabilized respiratory syncytial virus fusion protein elicits antibodies targeting a membrane-proximal epitope<br>Journal Of Virology (2023)<br>DOI: 10.1128/jvi.00929-23<br>Journal article	E4: Prefusion-stabilized DS-Cav1 vaccine immunology, rather than the RSVPreF3 OA product.

Record	Report and bibliographic identifier	Specific exclusion reason
001180	Mukhamedova, M. et al.<br>Vaccination with prefusion-stabilized respiratory syncytial virus fusion protein induces genetically and antigenically diverse antibody responses<br>Immunity (2021); pp. 769–780.e6<br>DOI: 10.1016/j.immuni.2021.03.004<br>Journal article	E4: Prefusion-stabilized DS-Cav1 vaccine immunology, rather than the RSVPreF3 OA product.
001182	Seufert, J. et al.<br>Vaccinations in Patients with Diabetes: Current Recommendations<br>Diabetologie und Stoffwechsel (2025); pp. 347–359<br>DOI: 10.1055/a-2603-9028<br>Recommendations	E1: Vaccination recommendations for people with diabetes; not an original randomized trial report.
001185	Malarski, M. et al.<br>Vaccine impact on antimicrobial resistance to inform Gavi, the vaccine alliance's 2018 vaccine investment strategy: Report from an expert survey.<br>F1000Research (2019)<br>DOI: 10.12688/f1000research.20100.1<br>Journal article	E3: Expert survey on vaccine impact on antimicrobial resistance, rather than an original randomized clinical comparison.
001186	Bartsch, Y.C. et al.<br>Vaccine-induced antibody Fc-effector functions in humans immunized with a combination Ad26.RSV.preF/RSV preF protein vaccine<br>Journal of Virology (2023)<br>DOI: 10.1128/jvi.00771-23<br>NCT03502707<br>Journal article	E4: Ad26.RSV.preF, alone or combined with RSV preF protein, is outside the RSVPreF3 OA product scope.
001189	Vaccine Responses in Patients With B Cell Malignancies<br>ClinicalTrials.gov (2021)<br>NCT05170399<br>ClinicalTrials.gov registry report	E5: Vaccine responses and possible BTK-inhibitor treatment interruption in B-cell malignancy; no assigned coadministration versus sequential vaccination schedules.
001190	Kricorian, C. et al.<br>Vaccine Status of Older Adults and Trust in Federal Institutions<br>Journal of the American Geriatrics Society (2026); pp. S244–S245<br>DOI: 10.1111/jgs.70425<br>Conference abstract	E3: Survey of vaccination status and trust in institutions, rather than a randomized vaccine-schedule comparison.

Record	Report and bibliographic identifier	Specific exclusion reason
001192	Black, S. et al.<br>Vaccines and otitis media<br>Pediatric Annals (2000); pp. 648–651<br>DOI: 10.3928/0090-4481-20001001-10<br>Review	E1: Review of vaccines and otitis media; not an original eligible randomized trial report.
001197	Bermingham, A. et al.<br>VII international symposium on respiratory viral infections<br>Antiviral Therapy (2007); pp. 671–693<br>Symposium report	E1: Multi-topic symposium report on respiratory viral infections; not an individual eligible trial report.
001198	Rashid, H. et al.<br>Viral respiratory infections at the Hajj: Comparison between UK and Saudi pilgrims<br>Clinical Microbiology and Infection (2008); pp. 569–574<br>DOI: 10.1111/j.1469-0691.2008.01987.x<br>Journal article	E3: Comparison of respiratory-virus infections in UK and Saudi Hajj pilgrims; not a randomized vaccination-schedule trial.
001205	Rebuffo-Scheer, C. et al.<br>Whole genome sequencing and evolutionary analysis of human respiratory syncytial virus A and B from Milwaukee, WI 1998-2010<br>PLoS ONE (2011)<br>DOI: 10.1371/journal.pone.0025468<br>Journal article	E2: RSV genome sequencing and evolutionary analysis, rather than a clinical vaccine-schedule comparison.
001206	Cilloniz, C.<br>Why RSV Infection Matter in Older Adults? The Urgent Need for Vaccination<br>Archivos de Bronconeumologia (2025); pp. 654–656<br>DOI: 10.1016/j.arbres.2025.05.010<br>Editorial	E1: Editorial on RSV vaccination in older adults; not an original randomized trial report.

Table S3. Primary and companion reports of the six included randomized trials (20 reports).

Study and record	Report and bibliographic identifier	Role and linkage

Study and record	Report and bibliographic identifier	Role and linkage
S01<br>NCT05879107<br>000586	Leroux-Roels, I. et al.<br>Immunogenicity and Safety of Co-administration of Adjuvanted Respiratory Syncytial Virus Prefusion F Protein Vaccine With 20-Valent Pneumococcal Conjugate Vaccine in Adults Aged ≥60 Years: A Randomized, Non-inferiority Trial<br>Clinical Infectious Diseases (2026)<br>DOI: 10.1093/cid/ciag250<br>NCT05879107	Primary article for analytical data<br>Trial registration explicitly identified.
S01<br>NCT05879107<br>000106	A Phase III, open-label, randomized, controlled, multi-country study to evaluate the immune response, safety, and reactogenicity of respiratory syncytial virus (RSV) prefusion protein 3 older adult (RSVPreF3 OA) investigational vaccine when co-administered with 20-Valent Pneumococcal Conjugate Vaccine (PCV20) in adults aged 60 Years and older<br>European trial registry (2023); 2022-501988-40-00<br>NCT05879107	European registry report<br>CTIS 2022-501988-40-00 matches the corresponding secondary registration identifier in NCT05879107.
S01<br>NCT05879107<br>000108	A Phase III, Open-label, Randomized, Controlled, Multi-Country Study to Evaluate the Immune Response, Safety and Reactogenicity of RSVPreF3 OA Investigational Vaccine When Co Administered With PCV20 in Adults Aged 60 Years and Older<br>ClinicalTrials.gov (2023)<br>NCT05879107	ClinicalTrials.gov registry report<br>Trial registration explicitly identified.
S02<br>NCT06374394<br>000587	Bonten, M et al.<br>Immunogenicity and Safety of Coadministration of AS01E-Adjuvanted Respiratory Syncytial Virus Prefusion F Protein Vaccine and a Coronavirus Disease 2019 mRNA Vaccine in Adults Aged ≥50 Years: A Phase 3, Randomized, Noninferiority Trial<br>Clinical Infectious Diseases (2026)<br>DOI: 10.1093/cid/ciag344<br>NCT06374394	Primary article for analytical data<br>Trial registration explicitly identified.
S02<br>NCT06374394<br>000065	A Phase 3, Open-label, Randomized, Controlled Study to Evaluate the Immune Response, Safety and Reactogenicity of RSVPreF3 OA Investigational Vaccine When Co-Administered With a COVID-19 mRNA Vaccine (Omicron XBB.1.5) in Adults Aged 50 Years and Above<br>ClinicalTrials.gov (2024)<br>NCT06374394	ClinicalTrials.gov registry report<br>Trial registration explicitly identified.
S02<br>NCT06374394<br>000163	A study on the immune response and safety of a vaccine against respiratory syncytial virus (RSV) when given alone and together with a vaccine against SARS-CoV-2 in adults aged 50 years and above<br>European trial registry (2024); 2023-510196-59-00<br>NCT06374394	European registry report<br>CTIS 2023-510196-59-00 matches the corresponding secondary registration identifier in NCT06374394.

Study and record	Report and bibliographic identifier	Role and linkage
S03<br>NCT05966090<br>000591	Roussy, J.-F. et al.<br>Immunogenicity and Safety of the Adjuvanted Respiratory Syncytial Virus Prefusion F Protein-Based Vaccine When Co-administered With the Adjuvanted Recombinant Herpes Zoster Subunit Vaccine in Adults ≥ 50 Years of Age<br>Clinical Infectious Diseases (2026)<br>DOI: 10.1093/cid/ciag208<br>NCT05966090	Primary article for analytical data<br>Trial registration explicitly identified.
S03<br>NCT05966090<br>000107	A Phase III, Open-label, Randomized, Controlled, Multi-country Study to Evaluate the Immune Response, Safety and Reactogenicity of RSVPreF3 OA Investigational Vaccine When Co-administered With Herpes Zoster Recombinant Subunit (HZ/su) Vaccine in Adults Aged 50 Years and Older<br>ClinicalTrials.gov (2023)<br>NCT05966090	ClinicalTrials.gov registry report<br>Trial registration explicitly identified.
S03<br>NCT05966090<br>000275	Mangan, N et al.<br>CO-ADMINISTRATION OF ADJUVANTED RESPIRATORY SYNCYTIAL VIRUS PREFUSION F PROTEIN VACCINE WITH ADJUVANTED RECOMBINANT ZOSTER VACCINE IN ADULTS AGED ≥50 YEARS<br>Internal medicine journal (2025); TO148, pp. 67–68<br>DOI: 10.1111/imj.70003<br>NCT05966090	Conference abstract<br>Trial registration explicitly identified.
S03<br>NCT05966090<br>000276	Dennis, P et al.<br>Co-administration of the adjuvanted respiratory syncytial virus (RSV) prefusion F protein vaccine (RSVPreF3 OA) with the adjuvanted recombinant zoster vaccine (RZV) in adults 50 years of age<br>European geriatric medicine (2024); p. S410<br>DOI: 10.1007/s41999-024-01090-y<br>NCT05966090	Conference abstract<br>Trial registration explicitly identified.
S03<br>NCT05966090<br>000277	RSV-020 study grp<br>Co-administration of the adjuvanted respiratory syncytial virus (RSV) prefusion F protein vaccine (RSVPreF3 OA) with the adjuvanted recombinant zoster vaccine (RZV) in adults ≥50years of age<br>Australasian Journal On Ageing (2025); 44(S1):e70037; subsection beginning Co-administration of the adjuvanted respiratory syncytial virus<br>DOI: 10.1111/ajag.70037<br>NCT05966090	Encore conference abstract<br>NCT05966090; 530 participants; GSK. Encore abstract from EuGMS 2024.

Study and record	Report and bibliographic identifier	Role and linkage
S04<br>NCT04841577<br>000598	Chandler, R. et al.<br>Immunogenicity, Reactogenicity, and Safety of AS01E-adjuvanted Respiratory Syncytial Virus (RSV) Prefusion F Protein-based Candidate Vaccine (RSVPreF3 OA) When Co-administered With a Seasonal Quadrivalent Influenza Vaccine in Older Adults: Results of a Phase 3, Open-Label, Randomized Controlled Trial<br>Clinical Infectious Diseases (2026); pp. e62–e70<br>DOI: 10.1093/cid/ciad786<br>NCT04841577	Primary article for analytical data<br>Trial registration explicitly identified.
S04<br>NCT04841577<br>000063	A Phase 3, Open-label, Randomized, Controlled, Multi-country Study to Evaluate the Immune Response, Safety and Reactogenicity of RSVPreF3 OA Investigational Vaccine When Co-administered With FLU-QIV Vaccine in Adults Aged 60 Years and Above<br>ClinicalTrials.gov (2021)<br>NCT04841577	ClinicalTrials.gov registry report<br>Trial registration explicitly identified.
S04<br>NCT04841577<br>000597	Chandler, R et al.<br>Immunogenicity, reactogenicity and safety of a Respiratory Syncytial Virus prefusion F (RSVPreF3) candidate vaccine co-administered with the seasonal quadrivalent influenza vaccine in older adults<br>Open forum infectious diseases (2022); pp. S864–S865<br>DOI: 10.1093/ofid/ofac492.1759<br>NCT04841577	Conference abstract<br>Trial registration explicitly identified.
S05<br>NCT05559476<br>000877	Buynak, R et al.<br>Randomized, Open-Label Phase 3 Study Evaluating Immunogenicity, Safety, and Reactogenicity of RSVPreF3 OA Coadministered with FLU-QIV-HD in Adults Aged ≥ 65<br>Infectious Diseases And Therapy (2024); pp. 1789–1805<br>DOI: 10.1007/s40121-024-00985-4<br>NCT05559476	Primary article for analytical data<br>Trial registration explicitly identified.
S05<br>NCT05559476<br>000064	A Phase 3, Open-label, Randomized, Controlled, Multicountry Study to Evaluate the Immune Response, Safety and Reactogenicity of RSVPreF3 OA Investigational Vaccine When Co-administered With FLU HD Vaccine in Adults Aged 65 Years and Above<br>ClinicalTrials.gov (2022)<br>NCT05559476	ClinicalTrials.gov registry report<br>Trial registration explicitly identified.
S05<br>NCT05559476<br>000790	Jaidhauser, I et al.<br>Phase 3, open-label, randomized, controlled study to evaluate immune response, safety and reactogenicity of the RSVPreF3 OA vaccine when coadministered with FLU-QIV-HD vaccine in adults aged ≥65 years<br>Innere Medizin (2024); P17-03, pp. S115–S116<br>DOI: 10.1007/s00108-024-01701-7<br>NCT05559476	Conference abstract<br>P17-03; RSVPreF3 OA plus FLU-QIV-HD; age ≥65 years; overlapping investigators include Valenciano, Buynak, Cannon, DeAtkine, Kirby and Usdan.

Study and record	Report and bibliographic identifier	Role and linkage
S06<br>NCT05568797<br>001043	Clark, R et al.<br>Safety and Immunogenicity of Respiratory Syncytial Virus Prefusion F Protein Vaccine when Co-administered with Adjuvanted Seasonal Quadrivalent Influenza Vaccine in Older Adults: A Phase 3 Randomized Trial<br>Clinical Infectious Diseases (2024); pp. 1088–1098<br>DOI: 10.1093/cid/ciae365<br>NCT05568797	Primary article for analytical data<br>Trial registration explicitly identified.
S06<br>NCT05568797<br>000105	A Phase III, Open-label, Randomized, Controlled, Multi-country Study to Evaluate the Immune Response, Safety and Reactogenicity of an RSVPreF3 OA Investigational Vaccine When Co-administered With FLU aQIV (Inactivated Influenza Vaccine - Adjuvanted) in Adults Aged 65 Years and Above<br>ClinicalTrials.gov (2022)<br>NCT05568797	ClinicalTrials.gov registry report<br>Trial registration explicitly identified.
S06<br>NCT05568797<br>000164	A study on the immune response and safety of a vaccine against respiratory syncytial virus (RSV) when given alone or co-administered with an adjuvanted vaccine against influenza in adults aged 65 years and above<br>European trial registry (2022); 2022-000623-21-BE<br>NCT05568797	European registry report<br>EudraCT 2022-000623-21-BE matches the corresponding secondary registration identifier in NCT05568797.

Note: The six primary articles and their supplements supplied antibody and adverse-event data. Nine registry reports supported protocol verification and trial characteristics, and five conference abstracts were linked as companion publications. ClinicalTrials.gov participant-flow results also supplied group-specific study-completion counts, cross-checked against the publications. All 20 reports represent the same six independent trials. The report set includes distinct ClinicalTrials.gov and European registry entries for the same trial. The high-dose QIV poster was linked by bibliographic matching of product, partner vaccine, age group, design and investigators.

Table S4 Source materials and dispositions for 71 reconciled reports

Original record	Corrected report identity	Material and source	Evidence supporting assessment	Disposition and trial linkage

Original record	Corrected report identity	Material and source	Evidence supporting assessment	Disposition and trial linkage
M3REC000001<br>doi:10.1016/j.japh.2025.102563	1076 Community Pharmacy Outreach Program to Promote RSV Vaccination in Eligible Older Adults and Improve Vaccination Rates<br>DOI:10.1016/j.japh.2025.102563<br>Conference abstract	Original indexed abstract, Methods or Design<br>https://doi.org/10.1016/j.japh.2025.102563	This study will specifically be comparing RSV vaccination rates at different community-based pharmacies in the same pharmacy chain during the winter respiratory season. Patients treated at the study pharmacy who are 75 years of age and older or 60-74 years of age with comorbid conditions will be included in the study. Patients younger than 60 years old will be excluded. At the start of each week during the study period, a report of the prescriptions ready to be picked up at the study pharmacy will be printed and used to identify patients to call that week to promote RSV vaccination. For patients 60-74 years of age, medication history consistent with treating comorbid conditions (heart/lung disease, immunosuppressing medications/conditions) will be used to confirm eligibility. The pharmacy-led outreach program will take place over three mo…	Excluded<br>Community pharmacy outreach evaluates vaccination uptake, without randomized assignment to coadministration or sequential administration.
M3REC000005<br>doi:10.1016/j.japh.2025.102587	1100 Preparation Time and Usability Assessment of Commercially Available Freeze-Dried Respiratory Syncytial Virus Vaccine Presentations<br>DOI:10.1016/j.japh.2025.102587<br>Conference abstract	Original indexed abstract, Methods or Design<br>https://doi.org/10.1016/j.japh.2025.102587	Two evaluations were conducted; an internal pilot study which then helped confirm the study design for a second expanded external study. The pilot evaluation was conducted in June 2024 with 13 Pharmacists and 4 nurses as voluntary internal study participants. Test sessions involved one participant and one test moderator. Participants were asked to complete 4 preparations of each presentation and no product brand names were disclosed. Trials 1 and 2 served as task familiarization which allowed for trained trials 3 and 4. Each reconstitution included evaluation of the following steps: i. Preparation / Injecting Diluent to Powder Vial, ii. Swirling, iii. Withdraw Medication to Syringe, and iv. It’s Ready to Inject. User perceptions (ratings, open-ended questions, efficiencies, benefits) were collected after completed reconstitution tasks. Th…	Excluded<br>Time-and-motion and usability assessment of vaccine preparation by healthcare providers, rather than a clinical vaccination-schedule trial.

Original record	Corrected report identity	Material and source	Evidence supporting assessment	Disposition and trial linkage
M3REC000106<br>doi:10.1002/central/cn-02730787/full	A Phase III, open-label, randomized, controlled, multi-country study to evaluate the immune response, safety, and reactogenicity of respiratory syncytial virus (RSV) prefusion protein 3 older adult (RSVPreF3 OA) investigational vaccine when co-administered with 20-Valent Pneumococcal Conjugate Vaccine (PCV20) in adults aged 60 Years and older<br>CTIS:2022-501988-40-00<br>European registry report	Original European registry record and ClinicalTrials.gov secondary identifiers<br>https://clinicaltrials.gov/study/NCT05879107	CTIS 2022-501988-40-00 matches the corresponding secondary registration identifier in NCT05879107.	Associated report<br>Associated registry record of NCT05879107.<br>NCT05879107; Matching registry secondary identifier
M3REC000163<br>doi:10.1002/central/cn-02731543/full	A study on the immune response and safety of a vaccine against respiratory syncytial virus (RSV) when given alone and together with a vaccine against SARS-CoV-2 in adults aged 50 years and above<br>CTIS:2023-510196-59-00<br>European registry report	Original European registry record and ClinicalTrials.gov secondary identifiers<br>https://clinicaltrials.gov/study/NCT06374394	CTIS 2023-510196-59-00 matches the corresponding secondary registration identifier in NCT06374394.	Associated report<br>Associated registry record of NCT06374394.<br>NCT06374394; Matching registry secondary identifier
M3REC000164<br>doi:10.1002/central/cn-02591734/full	A study on the immune response and safety of a vaccine against respiratory syncytial virus (RSV) when given alone or co-administered with an adjuvanted vaccine against influenza in adults aged 65 years and above<br>EudraCT:2022-000623-21-BE<br>European registry report	Original European registry record and ClinicalTrials.gov secondary identifiers<br>https://clinicaltrials.gov/study/NCT05568797	EudraCT 2022-000623-21-BE matches the corresponding secondary registration identifier in NCT05568797.	Associated report<br>Associated registry record of NCT05568797.<br>NCT05568797; Matching registry secondary identifier
M3REC000174<br>doi:10.1016/j.idh.2026.100448	Absenteeism due to COVID-19, influenza and RSV among hospital-based healthcare personnel in Greece during the 2024–2025 season: A cohort study<br>DOI:10.1016/j.idh.2026.100448<br>Journal article	Original indexed abstract<br>https://doi.org/10.1016/j.idh.2026.100448	Methods We followed 4421 hospital-based HCP from November 11, 2024, to May 25, 2025.	Excluded<br>Healthcare-personnel cohort measuring respiratory-virus-associated absenteeism; not a randomized vaccination-schedule comparison.

Original record	Corrected report identity	Material and source	Evidence supporting assessment	Disposition and trial linkage
M3REC000188<br>doi:10.1007/s00108-023-01640-9	After the COVID-19 pandemic—Which new vaccinations for adults are available or coming soon?<br>DOI:10.1007/s00108-023-01640-9<br>Review	Original indexed abstract<br>https://doi.org/10.1007/s00108-023-01640-9	The Standing Committee on Vaccination (STIKO) has responded accordingly by recommending the 20-valent pneumococcal conjugate vaccine PCV20 for standard and indication vaccination of adults.	Excluded<br>Overview of adult vaccines and recommendations; not an original randomized trial report.
M3REC000191<br>doi:10.1016/0167-7799(86)90133-2	An accidental human trial of recombinant vaccinia virus: A step towards acceptance of live recombinant vaccines?<br>DOI:10.1016/0167-7799(86)90133-2<br>Journal report	Original title and bibliographic publication type<br>https://doi.org/10.1016/0167-7799(86)90133-2	An accidental human trial of recombinant vaccinia virus: A step towards acceptance of live recombinant vaccines?	Excluded<br>Report concerns recombinant vaccinia virus, rather than the RSVPreF3 OA vaccination-schedule intervention.
M3REC000223<br>doi:10.34172/ajcmi.3719	Bacterial and Viral Respiratory Co-infections With SARS-CoV-2 in Deceased Patients of North Khorasan, Iran, During 2020-2023<br>DOI:10.34172/ajcmi.3719<br>Journal article	Original indexed abstract, Methods or Design<br>https://doi.org/10.34172/ajcmi.3719	We evaluated bacterial and viral co-infections in 480 samples from deceased patients and 480 randomly selected samples from surviving SARS-CoV-2-positive patients. Samples were tested for respiratory bacterial pathogens, including Streptococcus pneumoniae, Haemophilus influenzae, Klebsiella pneumoniae, Mycoplasma pneumoniae, Acinetobacter baumannii, Pseudomonas aeruginosa, and Staphylococcus aureus, as well as important respiratory viral pathogens, including influenza virus, human metapneumovirus, parainfluenza virus (A–D), adenovirus, respiratory syncytial virus (RSV), and bocavirus using polymerase chain reaction (PCR) and reverse transcription PCR (RT-PCR) assays.	Excluded<br>Observational comparison of bacterial and viral coinfections in SARS-CoV-2-positive patients; not a vaccination-schedule trial.
M3REC000247<br>doi:10.1034/j.1399-3038.2000.00507.x	Can immunization affect the development of allergy?<br>DOI:10.1034/j.1399-3038.2000.00507.x<br>Review	Original indexed abstract<br>https://doi.org/10.1034/j.1399-3038.2000.00507.x	A recent study showed that positive tuberculin responses in schoolchildren correlated with a lower prevalence of atopic disorders, but other studies did not find a relation between BCG vaccination and allergic disease or sensitization.	Excluded<br>Review of immunization and allergic disease; not an original eligible randomized comparison.
M3REC000274<br>report:M3REC000274	Co-administered RSV and pneumococcal disease vaccines in older adults<br>DOI:10.1016/s1473-3099(26)00248-3<br>Research news	Publisher metadata plus matching original author, journal, issue and page<br>https://api.elsevier.com/content/article/pii/S1473309926002483	Venkatesan; The Lancet Infectious Diseases 26(6), e216, June 2026; publisher title Research in brief.	Excluded<br>Research news item. Author, journal, volume, issue and page identify the parent Research in brief report; it is not an original randomized trial report.

Original record	Corrected report identity	Material and source	Evidence supporting assessment	Disposition and trial linkage
M3REC000277<br>report:M3REC000277	Co-administration of the adjuvanted respiratory syncytial virus (RSV) prefusion F protein vaccine (RSVPreF3 OA) with the adjuvanted recombinant zoster vaccine (RZV) in adults ≥50years of age<br>DOI:10.1111/ajag.70037#M3REC000277<br>Encore conference abstract	Publisher conference abstract, Methods and Funding<br>https://onlinelibrary.wiley.com/doi/10.1111/ajag.70037	NCT05966090; 530 participants; GSK. Encore abstract from EuGMS 2024.	Associated report<br>Associated conference report of NCT05966090.<br>NCT05966090; Publisher abstract registration
M3REC000285<br>doi:10.7326/annals-25-00904-jc	Coadministration of RSV + influenza or COVID-19 vaccines was noninferior to separate administration for immune responses in adults aged ≥50 y<br>DOI:10.7326/annals-25-00904-jc<br>ACP Journal Club summary	Original title and bibliographic publication type<br>https://doi.org/10.7326/annals-25-00904-jc	Coadministration of RSV + influenza or COVID-19 vaccines was noninferior to separate administration for immune responses in adults aged ≥50 y; indexed publication type: Note; Note	Excluded<br>ACP Journal Club evidence summary of a published coadministration trial; no independent trial dataset.
M3REC000301<br>doi:10.1093/infdis/jiag111	Comparison of Respiratory Syncytial Virus (RSV)-Specific Antibody Durability in Pregnant/Postpartum Individuals and Older Adults After RSV Vaccination<br>DOI:10.1093/infdis/jiag111<br>Journal article	Original indexed abstract<br>https://doi.org/10.1093/infdis/jiag111	We conducted prospective cohort studies among pregnant and older adults after first RSV vaccination in 2023-2024.	Excluded<br>Prospective comparison of antibody durability in pregnant or postpartum individuals and older adults; no randomized schedule comparison.
M3REC000310<br>doi:10.1093/ofid/ofab466.874	Compliance and Performance Characteristics of Subject Collected Versus Health-care Worker Collected Nasal Swabs for Respiratory Viral Surveillance<br>DOI:10.1093/ofid/ofab466.874<br>Conference abstract	Original indexed abstract, Methods or Design<br>https://doi.org/10.1093/ofid/ofab466.874	Adult Military Health System (MHS) beneficiaries were enrolled in an influenza vaccine effectiveness trial (PAIVED). Following vaccination, subjects were instructed on SCNS and completion of a symptom diary and were contacted weekly to ascertain ILI symptoms (fever, sore throat, and/or cough). In the event of an ILI, subjects completed the symptom diary and SCNS and were scheduled a clinic visit for HCWC. Swabs were tested with the Luminex NxTAG® Respiratory Pathogen Panel. We evaluated compliance with swab collection, positive percent agreement (PPA) of SCNS using PCR detection from either HCWC or SCNS as the reference standard, and agreement between paired swabs using the Cohen Kappa coefficient (K).	Excluded<br>Evaluation of participant-collected versus healthcare-worker-collected nasal swabs, rather than vaccination schedules.

Original record	Corrected report identity	Material and source	Evidence supporting assessment	Disposition and trial linkage
M3REC000358<br>doi:10.1056/nejme2513191	Current and Emerging Approaches to Evaluating Influenza Vaccine Performance<br>DOI:10.1056/nejme2513191<br>Editorial	Original title and bibliographic publication type<br>https://doi.org/10.1056/nejme2513191	Current and Emerging Approaches to Evaluating Influenza Vaccine Performance; indexed publication type: Editorial	Excluded<br>Editorial on evaluation of influenza vaccine performance; not an original RSV coadministration trial report.
M3REC000367<br>doi:10.1016/0264-410x(90)90051-m	Determinants of susceptibility to challenge and the antibody response of adult volunteers given experimental respiratory syncytial virus vaccines<br>DOI:10.1016/0264-410x(90)90051-m<br>Journal article	Original indexed abstract<br>https://doi.org/10.1016/0264-410x(90)90051-m	Ts1B might be developed further to produce a live-virus vaccine.	Excluded<br>Experimental temperature-sensitive live RSV vaccines and challenge, rather than RSVPreF3 OA.
M3REC000369<br>doi:10.1016/j.vaccine.2018.07.048	Development and application of a multiplex assay for the simultaneous measurement of antibody responses elicited by common childhood vaccines<br>DOI:10.1016/j.vaccine.2018.07.048<br>Journal article	Original indexed abstract<br>https://doi.org/10.1016/j.vaccine.2018.07.048	Thus, the PVMA is a reliable, high-throughput technique that requires minimal sample volume to measure multiple antibody concentrations concurrently, and is an efficient alternative to ELISAs for the measurement of vaccine-elicited antibody responses in large cohorts. © 2018 The Authors	Excluded<br>Multiplex assay development for antibody responses to childhood vaccines; not a clinical RSV coadministration trial.
M3REC000376<br>doi:10.1128/cvi.00594-13	Development of a luciferase immunoprecipitation system assay to detect IgG antibodies against human respiratory syncytial virus nucleoprotein<br>DOI:10.1128/cvi.00594-13<br>Journal article	Original indexed abstract<br>https://doi.org/10.1128/cvi.00594-13	The RSV-N gene was cloned into a mammalian expression vector, pREN2, and the expressed luciferase-tagged protein (Ruc-N) detected anti-RSV-N-specific IgG antibodies using a high-throughput immunoprecipitation method (the luciferase immunoprecipitation system [LIPS]-NRSV assay).	Excluded<br>Development of an RSV nucleoprotein antibody assay; not an eligible clinical vaccination-schedule comparison.
M3REC000449<br>doi:10.3760/cma.j.issn.1007-9408.2023.06.104	Efficacy of bivalent prefusion F vaccine during pregnancy in preventing respiratory syncytial virus-related diseases in infants: a phase III, double-blind, randomized controlled trial<br>DOI:10.3760/cma.j.issn.1007-9408.2023.06.104<br>Secondary research summary	Original title and bibliographic publication type<br>https://doi.org/10.3760/cma.j.issn.1007-9408.2023.06.104	Efficacy of bivalent prefusion F vaccine during pregnancy in preventing respiratory syncytial virus-related diseases in infants: a phase III, double-blind, randomized controlled trial; indexed publication type: Note	Excluded<br>Indexed as a note summarizing a maternal bivalent RSV vaccine efficacy trial with infant outcomes; not an original RSVPreF3 OA schedule trial report.

Original record	Corrected report identity	Material and source	Evidence supporting assessment	Disposition and trial linkage
M3REC000495<br>doi:10.1086/315859	Evaluation of a live, cold-passaged, temperature-sensitive, respiratory syncytial virus vaccine candidate in infancy<br>DOI:10.1086/315859<br>Journal article	Original indexed abstract<br>https://doi.org/10.1086/315859	A live-attenuated, intranasal respiratory syncytial virus (RSV) candidate vaccine, cpts-248/404, was tested in phase 1 trials in 114 children, including 37 1-2-month-old infants - a target age for RSV vaccines.	Excluded<br>Live-attenuated RSV vaccination in infants or children; outside the product and population scope.
M3REC000501<br>doi:10.1111/j.1600-0684.2011.00491.x	Evaluation of immunological parameters for correlates of protection against live siv challenge in recombinant simian varicella virus-simian immunodeficiency virus (RSVV-SIV) vaccinated nonhuman primates<br>DOI:10.1111/j.1600-0684.2011.00491.x#M3REC000501<br>Conference abstract	Original indexed abstract<br>https://doi.org/10.1111/j.1600-0684.2011.00491.x	A vaccine study tested SVV constructs with SIV env and gag gene inserts (rSVV-SIVenv & rSVV-SIVgag) against control animals vaccinated with rSVV containing respiratory syncytial virus G and M2 genes (SVV-RSV) following intravenous challenge with SIV. rSVV-SIV vaccinated animals showed humoral, cellular, and neutralizing immune responses and significantly reduced levels of virus in the peripheral circulation following SIV challenge when compared with controls (Traina-Dorge, in press).	Excluded<br>Recombinant simian varicella/SIV vaccination and SIV challenge in nonhuman primates.
M3REC000503<br>doi:10.1016/s0264-410x(99)00527-7	Evaluation of the live attenuated cpts 248/404 RSV vaccine in combination with a subunit RSV vaccine (PFP-2) in healthy young and older adults<br>DOI:10.1016/s0264-410x(99)00527-7<br>Journal article	Original indexed abstract<br>https://doi.org/10.1016/s0264-410x(99)00527-7	The safety and immunogenicity of the live attenuated cold-passaged, temperature-sensitive (cpts) 248/404 respiratory syncytial Virus (RSV) A2 and the RSV A2 purified F glycoprotein (PFP-2) vaccine candidates were evaluated in a placebo-controlled trial in 60 healthy young adults and 60 healthy elderly subjects using simultaneous and sequential (cpts 248/404 followed by PFP-2) vaccination schedules.	Excluded<br>Combination of live cpts248/404 RSV vaccine and PFP-2 subunit RSV vaccine; not RSVPreF3 OA with a partner vaccine.
M3REC000538<br>doi:10.2139/ssrn.6556803	Health events reported by US adults aged ≥60 years who received a respiratory syncytial virus vaccine alone or with other vaccines<br>DOI:10.2139/ssrn.6556803<br>Preprint	Original indexed abstract<br>https://doi.org/10.2139/ssrn.6556803	Data concerning reactogenicity of RSV vaccines when administered with other vaccines are limited.	Excluded<br>Voluntary v-safe survey of self-reported health events; observational exposure groups rather than randomized vaccine schedules.
M3REC000552<br>doi:10.1016/s0065-2776(08)60674-4	Human antibodies from combinatorial libraries<br>DOI:10.1016/s0065-2776(08)60674-4<br>Review	Original title and bibliographic publication type<br>https://doi.org/10.1016/s0065-2776(08)60674-4	Human antibodies from combinatorial libraries; indexed publication type: Review	Excluded<br>Review concerning combinatorial antibody libraries; not an original vaccine-schedule trial.

Original record	Corrected report identity	Material and source	Evidence supporting assessment	Disposition and trial linkage
M3REC000571<br>doi:10.1016/0264-410x(89)90278-8	Immunization of cotton rats with the fusion (F) and large (G) glycoproteins of respiratory syncytial virus (RSV) protects against RSV challenge without potentiating RSV disease<br>DOI:10.1016/0264-410x(89)90278-8<br>Journal article	Original indexed abstract<br>https://doi.org/10.1016/0264-410x(89)90278-8	In the present study, we evaluated a newly developed purified F and G glycoprotein vaccine over a wide dosage range for immunogenicity, efficacy and capacity to potentiate pulmonary pathology in cotton rats.	Excluded<br>Cotton-rat immunization with RSV F and G glycoproteins and viral challenge.
M3REC000577<br>doi:10.1016/0264-410x(93)90290-e	Immunogenciity and pathogenicity of a triple temperature-sensitive modified respiratory syncytial virus in adult volunteers<br>DOI:10.1016/0264-410x(93)90290-e<br>Journal article	Original indexed abstract<br>https://doi.org/10.1016/0264-410x(93)90290-e	The immunogenicity and pathogenicity of a strain of respiratory syncytial (RS) virus modified by sequential induction of three temperature-sensitive (ts) mutations have been evaluated by intranasal administration to 22 adult volunteers.	Excluded<br>Experimental temperature-sensitive live RSV vaccines and challenge, rather than RSVPreF3 OA.
M3REC000579<br>doi:10.1002/jmv.1890250405	Immunogenicity and pathogenicity of temperature-sensitive modified respiratory syncytial virus in adult volunteers<br>DOI:10.1002/jmv.1890250405<br>Journal article	Original indexed abstract<br>https://doi.org/10.1002/jmv.1890250405	The double mutant tslB in particular has characteristics that suggest that it may be suitable for further development as a live RS virus vaccine.	Excluded<br>Experimental temperature-sensitive live RSV vaccines and challenge, rather than RSVPreF3 OA.
M3REC000629<br>doi:10.1542/peds.104.4.993	IMpact-RSV study group report [1] (multiple letters)<br>DOI:10.1542/peds.104.4.993<br>Letters and comments	Original title and bibliographic publication type<br>https://doi.org/10.1542/peds.104.4.993	IMpact-RSV study group report [1] (multiple letters); indexed publication type: Letter	Excluded<br>Letters commenting on the IMpact-RSV palivizumab study; not an original RSVPreF3 OA trial report.
M3REC000630<br>doi:10.7326/j23-0038	In older adults, an AS01E-adjuvanted RSVPreF3 OA vaccine reduced RSV-related lower respiratory tract disease<br>DOI:10.7326/j23-0038<br>ACP Journal Club summary	Original title and bibliographic publication type<br>https://doi.org/10.7326/j23-0038	In older adults, an AS01E-adjuvanted RSVPreF3 OA vaccine reduced RSV-related lower respiratory tract disease; indexed publication type: Note	Excluded<br>ACP Journal Club evidence summary of RSV vaccine effectiveness; not an original vaccination-schedule trial report.
M3REC000631<br>doi:10.7326/annals-25-05113-jc	In older adults, RSV prefusion F vaccine reduced hospitalization for RSV-related respiratory tract disease vs. no vaccine<br>DOI:10.7326/annals-25-05113-jc<br>ACP Journal Club summary	Original title and bibliographic publication type<br>https://doi.org/10.7326/annals-25-05113-jc	In older adults, RSV prefusion F vaccine reduced hospitalization for RSV-related respiratory tract disease vs. no vaccine; indexed publication type: Note	Excluded<br>ACP Journal Club evidence summary of RSV vaccine effectiveness; not an original vaccination-schedule trial report.

Original record	Corrected report identity	Material and source	Evidence supporting assessment	Disposition and trial linkage
M3REC000645<br>doi:10.1007/s11428-025-01379-x	Indication-based vaccinations for people with diabetes mellitus<br>DOI:10.1007/s11428-025-01379-x<br>Recommendations	Original indexed abstract<br>https://doi.org/10.1007/s11428-025-01379-x	It also highlights new influenza vaccines as well as the available RSV vaccines and the associated at-risk populations.	Excluded<br>Vaccination recommendations for people with diabetes; not an original randomized trial report.
M3REC000658<br>pmid:23484387	[Influenza in Poland in 2010].<br>Report:M3REC000658<br>Journal article	Original indexed abstract<br>https://www.embase.com/search/results?subaction=viewrecord&id=L369169225&from=export	THE PURPOSE OF STUDY: To assess the epidemiological situation of influenza and influenza like illnesses in Poland in 2010 compared to the situation in 2009.	Excluded<br>National influenza surveillance report for Poland; not an RSV vaccination-schedule trial.
M3REC000663<br>doi:10.18683/germs.2025.1475	Innovative approaches in RSV prevention: The expanding role of monoclonal antibodies in protection for all infants<br>DOI:10.18683/germs.2025.1475<br>Review	Original title and bibliographic publication type<br>https://doi.org/10.18683/germs.2025.1475	Innovative approaches in RSV prevention: The expanding role of monoclonal antibodies in protection for all infants; indexed publication type: Article	Excluded<br>Overview of monoclonal-antibody prevention in infants; not an original RSVPreF3 OA trial report.
M3REC000693<br>doi:10.1007/978-1-4757-9209-6_17	Management of respiratory syncytial virus infections<br>DOI:10.1007/978-1-4757-9209-6_17<br>Review and conference paper	Original title and bibliographic publication type<br>https://doi.org/10.1007/978-1-4757-9209-6_17	Management of respiratory syncytial virus infections; indexed publication type: Conference Paper	Excluded<br>Review of RSV infection management, indexed as a conference paper; not an original eligible trial report.
M3REC000713<br>doi:10.1016/j.vaccine.2022.07.049	Midwives' attitudes toward participation of pregnant individuals in a preventive vaccine hypothetical clinical trial<br>DOI:10.1016/j.vaccine.2022.07.049<br>Journal article	Original indexed abstract, Methods or Design<br>https://doi.org/10.1016/j.vaccine.2022.07.049	We conducted an anonymous survey from the 11th of September to the 11th of November 2020. Primary endpoint was the willingness to encourage Pregnant individuals to participate in a hypothetical respiratory syncytial virus (RSV) vaccine clinical trial.	Excluded<br>Survey of midwives about a hypothetical maternal vaccine trial; no trial participants randomized to vaccination schedules.
M3REC000747<br>doi:10.1093/infdis/jit169	Nonreplicating vaccines can protect african green monkeys from the memphis 37 strain of respiratory syncytial virus<br>DOI:10.1093/infdis/jit169<br>Journal article	Original indexed abstract, Methods or Design<br>https://doi.org/10.1093/infdis/jit169	Monkeys were immunized with F and G proteins adjuvanted with immunostimulatory (CpG) oligodeoxyribonucleotides admixed with either Alhydrogel or ISCOMATRIX adjuvant. Delivery of F and G proteins via replication incompetent recombinant vesicular stomatitis viruses (VSVs) and human adenoviruses was also evaluated. Mucosally or parenterally administered recombinant adenoviruses were used in prime-boost regimens with adjuvanted proteins or recombinant DNA.	Excluded<br>RSV vaccination and challenge in African green monkeys.

Original record	Corrected report identity	Material and source	Evidence supporting assessment	Disposition and trial linkage
M3REC000750<br>doi:10.1177/10600280251382202	Novel Maintenance Therapies for Chronic Obstructive Pulmonary Disorder<br>DOI:10.1177/10600280251382202<br>Review	Original indexed abstract<br>https://doi.org/10.1177/10600280251382202	Study selection and data extraction: English-language primary literature and review articles evaluated.	Excluded<br>Review of COPD maintenance therapies and recommendations; not an original coadministration trial.
M3REC000789<br>doi:10.1111/jpc.15999	Phase 3 clinical trial results for nirsevimab: Is it time to consider population-level RSV immunoprophylaxis?<br>DOI:10.1111/jpc.15999<br>Commentary	Original title and bibliographic publication type<br>https://doi.org/10.1111/jpc.15999	Phase 3 clinical trial results for nirsevimab: Is it time to consider population-level RSV immunoprophylaxis?; indexed publication type: Note	Excluded<br>Comment on a nirsevimab trial and infant immunoprophylaxis; not an original eligible RSV vaccine trial report.
M3REC000790<br>report:M3REC000790	Phase 3, open-label, randomized, controlled study to evaluate immune response, safety and reactogenicity of the RSVPreF3 OA vaccine when coadministered with FLU-QIV-HD vaccine in adults aged ≥65 years<br>DOI:10.1007/s00108-024-01701-7#M3REC000790<br>Conference abstract	Original Web of Science full record, poster title, investigators and locator; publisher abstract-book identity<br>https://doi.org/10.1007/s00108-024-01701-7	P17-03; RSVPreF3 OA plus FLU-QIV-HD; age ≥65 years; overlapping investigators include Valenciano, Buynak, Cannon, DeAtkine, Kirby and Usdan.	Associated report<br>Associated conference report of NCT05559476.<br>NCT05559476; Bibliographic association
M3REC000804<br>doi:10.1016/s0002-9343(98)00262-9	Pneumonia in residents of long-term care facilities: Epidemiology, etiology, management, and prevention<br>DOI:10.1016/s0002-9343(98)00262-9<br>Review	Original indexed abstract<br>https://doi.org/10.1016/s0002-9343(98)00262-9	Pneumococcal and influenza vaccines should be administered to all residents.	Excluded<br>Review of pneumonia epidemiology, management and prevention in long-term care; not an original eligible trial.
M3REC000810<br>doi:10.1093/ofid/ofac492.194	Potential Clinical Impact of Respiratory Syncytial Virus (RSV) Vaccination in Older Adults in the United States<br>DOI:10.1093/ofid/ofac492.194<br>Conference abstract	Original indexed abstract, Methods or Design<br>https://doi.org/10.1093/ofid/ofac492.194	A decision-analytic model was developed (Figure 1). A strategy was used where the entire US population aged ≥ 60 years was given a single dose vaccine with an assumed efficacy of 75% against ARD, LRTD, and LRTD-hospitalizations and compared to a population who did not receive a vaccine. Given the uncertainty around RSV hospitalization rates, 2 base-case scenarios were created: (1) hospitalization rates that used RSV-specific claim codes and (2) hospitalization rates increased by 49% to account for underreporting in claims; Table 1 shows key inputs. One-way sensitivity analyses were used to assess the impact of specific parameter estimates.	Excluded<br>Decision-analytic model of hypothetical RSV vaccination impact; not a randomized trial.

Original record	Corrected report identity	Material and source	Evidence supporting assessment	Disposition and trial linkage
M3REC000814<br>doi:10.1007/s100960100613	Pre- and in-hospital management of community-acquired pneumonia in Southern France, 1998-99<br>DOI:10.1007/s100960100613<br>Journal article	Original indexed abstract<br>https://doi.org/10.1007/s100960100613	A prospective, hospital-based, multicenter study was undertaken to identify the reasons for hospital admission, to describe antibiotic treatment before and during hospitalization, and to determine the outcome of community-acquired pneumonia (CAP).	Excluded<br>Prospective observational study of community-acquired pneumonia management in southern France; not a vaccine trial.
M3REC000842<br>doi:10.1016/s1473-3099(24)00674-1	Prioritising at-risk adults in vaccine coadministration trials<br>DOI:10.1016/s1473-3099(24)00674-1<br>Commentary	Original title and bibliographic publication type<br>https://doi.org/10.1016/s1473-3099(24)00674-1	Prioritising at-risk adults in vaccine coadministration trials; indexed publication type: Note	Excluded<br>Comment on the Goswami mRNA-1345 coadministration trial; no independent trial dataset.
M3REC000882<br>doi:10.1111/apa.17301	Rapid review showed that real-world results on how nirsevimab prevented respiratory syncytial virus hospitalisations were similar to randomised trials<br>DOI:10.1111/apa.17301<br>Rapid review	Original title and bibliographic publication type<br>https://doi.org/10.1111/apa.17301	Rapid review showed that real-world results on how nirsevimab prevented respiratory syncytial virus hospitalisations were similar to randomised trials; indexed publication type: Article	Excluded<br>Rapid review of nirsevimab effectiveness; not an original RSVPreF3 OA trial report.
M3REC000908<br>doi:10.1056/nejmc2311560	RENOIR Trial — RSVpreF Vaccine Efficacy over Two Seasons: To the Editor<br>DOI:10.1056/nejmc2311560<br>Letter	Original title and bibliographic publication type<br>https://doi.org/10.1056/nejmc2311560	RENOIR Trial — RSVpreF Vaccine Efficacy over Two Seasons: To the Editor; indexed publication type: Letter	Excluded<br>Letter concerning RENOIR vaccine efficacy; not an original eligible coadministration trial report.
M3REC000909<br>doi:10.1177/1091581820985782	Repeated Dose Toxicity Study and Developmental and Reproductive Toxicology Studies of a Respiratory Syncytial Virus Candidate Vaccine in Rabbits and Rats<br>DOI:10.1177/1091581820985782<br>Journal article	Original indexed abstract<br>https://doi.org/10.1177/1091581820985782	The local and systemic effects of biweekly intramuscular injections of the RSVPreF3 vaccine (unadjuvanted, adjuvanted with AS01, or coadministered with a diphtheria-tetanus-acellular pertussis vaccine) was tested in a repeated dose toxicity study in rabbits.	Excluded<br>Repeated-dose and reproductive toxicology studies in rabbits and rats.
M3REC000914<br>doi:10.1006/biol.2001.0283	Residual DNA quantification in clinical batches of BBG2Na, a recombinant subunit vaccine against human respiratory syncytial virus<br>DOI:10.1006/biol.2001.0283<br>Journal article	Original indexed abstract<br>https://doi.org/10.1006/biol.2001.0283	BBG2Na, a well-defined recombinant protein produced in Escherichia coli, is a promising human respiratory syncytial virus subunit vaccine candidate.	Excluded<br>Residual-DNA quantification in BBG2Na vaccine manufacturing batches; not a clinical schedule comparison.

Original record	Corrected report identity	Material and source	Evidence supporting assessment	Disposition and trial linkage
M3REC000918<br>doi:10.2165/00151829-200605060-00011	Respiratory syncytial virus bronchiolitis: Current and future strategies for treatment and prophylaxis<br>DOI:10.2165/00151829-200605060-00011<br>Review	Original indexed abstract<br>https://doi.org/10.2165/00151829-200605060-00011	Efforts to develop a safe and effective RSV vaccine have continued despite the poor outcomes observed following the administration of formalin-inactivated formulations in the 1960s.	Excluded<br>Review of RSV bronchiolitis treatment and prophylaxis; not an original eligible trial report.
M3REC000924<br>report:M3REC000924	Respiratory syncytial virus infection<br>Report:M3REC000924<br>Conference paper	Original title and bibliographic publication type<br>https://www.embase.com/search/results?subaction=viewrecord&id=L28559372&from=export	Respiratory syncytial virus infection; indexed publication type: Conference Paper	Excluded<br>Italian conference paper on RSV infection in a pediatric journal; not independently eligible under the current report-format criteria.
M3REC000932<br>doi:10.1055/a-2586-3974	Respiratory Syncytial Virus Prevention in the Adult Population: State of the Art<br>DOI:10.1055/a-2586-3974<br>Review	Original indexed abstract<br>https://doi.org/10.1055/a-2586-3974	Several vaccination strategies have been developed, including nucleic acid, subunit, chimeric, live-attenuated, particle-based, and recombinant vaccines, with varying results.	Excluded<br>Review of RSV prevention strategies; not an original randomized coadministration trial report.
M3REC000933<br>pmid:11249487	Respiratory syncytial virus prevention: past and present strategies.<br>Report:M3REC000933<br>Review	Original indexed abstract<br>https://www.embase.com/search/results?subaction=viewrecord&id=L33452844&from=export	There is no safe and effective RSV vaccine to use in those infants who are at highest risk from the infection.	Excluded<br>Review of RSV prevention strategies; not an original randomized coadministration trial report.
M3REC000943<br>doi:10.1021/acs.jmedchem.5c03667	Respiratory Syncytial Virus (RSV) Prophylaxis: Clesrovimab and Beyond<br>DOI:10.1021/acs.jmedchem.5c03667<br>Editorial	Original title and bibliographic publication type<br>https://doi.org/10.1021/acs.jmedchem.5c03667	Respiratory Syncytial Virus (RSV) Prophylaxis: Clesrovimab and Beyond; indexed publication type: Editorial	Excluded<br>Editorial on clesrovimab and RSV prophylaxis; not an original eligible vaccine trial report.
M3REC000951<br>pmid:8035030	Respiratory syncytial virus-specific cell-mediated immune responses after vaccination with a purified fusion protein subunit vaccine<br>Report:M3REC000951<br>Journal article	Original indexed abstract<br>https://www.embase.com/search/results?subaction=viewrecord&id=L24226374&from=export	Vaccination with a respiratory syncytial virus (RSV) fusion protein subunit vaccine (PFP-2) was done to determine if this vaccine induced evidence of cell-mediated immunity to RSV and if cell-mediated immunity prevented RSV reinfection.	Excluded<br>PFP-2 subunit RSV vaccine in children aged 12–18 months; outside the product and population scope.

Original record	Corrected report identity	Material and source	Evidence supporting assessment	Disposition and trial linkage
M3REC000955<br>doi:10.1080/25310429.2025.2451456	Respiratory syncytial virus vaccination in older adults and patients with chronic disorders: A position paper from the Portuguese Society of Pulmonology, the Portuguese Association of General and Family Medicine, the Portuguese Society of Cardiology, the Portuguese Society of Infectious Diseases and Clinical Microbiology, the Portuguese Society of Endocrinology, Diabetes and Metabolism, and the Portuguese Society of Internal Medicine<br>DOI:10.1080/25310429.2025.2451456<br>Position paper	Original indexed abstract<br>https://doi.org/10.1080/25310429.2025.2451456	BackgroundRespiratory syncytial virus (RSV) is an important cause of lower respiratory tract infection, hospitalisation and death in adults.MethodsBased on evidence regarding the impact of RSV on adult populations at risk for severe infection and the efficacy and safety of RSV vaccines, the Portuguese Society of Pulmonology, the Portuguese Association of General and Family Medicine, the Portuguese Society of Cardiology, the Portuguese Society of Infectious Diseases and Clinical Microbiology, the Portuguese Society of Endocrinology, Diabetes and Metabolism, and the Portuguese Society of Internal Medicine endorses this position paper with recommendations to prevent RSV-associated disease and its complications in adults through vaccination.ConclusionThe RSV vaccine is recommended for people aged >= 50 years with risk factors (chronic obstruc…	Excluded<br>Professional-society position paper on RSV vaccination; not an original randomized trial report.
M3REC000969<br>pmid:4305199	Respiratory virus immunization. I. A field trial of two inactivated respiratory virus vaccines; an aqueous trivalent parainfluenza virus vaccine and an alum-precipitated respiratory syncytial virus vaccine.<br>Report:M3REC000969<br>Journal article	Original title and bibliographic publication type<br>https://www.embase.com/search/results?subaction=viewrecord&id=L89093989&from=export	Respiratory virus immunization. I. A field trial of two inactivated respiratory virus vaccines; an aqueous trivalent parainfluenza virus vaccine and an alum-precipitated respiratory syncytial virus vaccine.; indexed publication type: Article	Excluded<br>Historical inactivated parainfluenza and alum-precipitated RSV vaccines, rather than RSVPreF3 OA.
M3REC000971<br>report:M3REC000971	Respiratory virus vaccines: recent advances<br>Report:M3REC000971<br>Journal article	Original indexed abstract<br>https://www.scopus.com/pages/publications/0016805517?origin=resultslist	The author reviewed the recent advances with regard to development of respiratory viral vaccines, with special emphasis on respiratory syncytial and influenza virus vaccines since these virus infections are the most important in terms of morbidity and mortality.	Excluded<br>Review of respiratory-virus vaccine development; not an original eligible trial report.

Original record	Corrected report identity	Material and source	Evidence supporting assessment	Disposition and trial linkage
M3REC001002<br>doi:10.1080/21645515.2017.1334746	RSV vaccine candidate immunogenic in older people<br>DOI:10.1080/21645515.2017.1374796<br>Research news	Correct primary news report<br>https://pmc.ncbi.nlm.nih.gov/articles/PMC5612048/	RSV subsection belongs to Human Vaccines & Immunotherapeutics News, 2017, pp. 1958–1959; the imported DOI instead identifies a cancer immunotherapy paper.	Excluded<br>RSV vaccine news subsection in Human Vaccines & Immunotherapeutics News; imported DOI and abstract were mismatched and are corrected in this ledger.
M3REC001049<br>doi:10.1016/0264-410x(95)00034-x	Safety and immunogenicity of the PFP vaccine against respiratory syncytial virus (RSV): The Western blot assay aids in distinguishing immune responses of the PFP vaccine from RSV infection<br>DOI:10.1016/0264-410x(95)00034-x<br>Journal article	Original indexed abstract<br>https://doi.org/10.1016/0264-410x(95)00034-x	PFP-1 vaccine was evaluated in a randomized controlled study in 47 RSV seropositive children.	Excluded<br>PFP-1 RSV vaccine in children with influenza vaccine as the comparator; outside the product, population and schedule scope.
M3REC001077<br>doi:10.1002/jmv.25489	Seroepidemiology of respiratory syncytial virus in western India with special reference to appropriate age for infant vaccination.<br>DOI:10.1002/jmv.25489<br>Journal article	Original indexed abstract<br>https://doi.org/10.1002/jmv.25489	Respiratory syncytial virus (RSV) causes significant infant mortality worldwide and a vaccine may be available soon.	Excluded<br>RSV seroepidemiology in children in western India; not a vaccination-schedule trial.
M3REC001104<br>report:M3REC001104	Surgical masks or N95 respirators for the flu?<br>Report:M3REC001104<br>Research commentary	Original title and bibliographic publication type<br>https://www.embase.com/search/results?subaction=viewrecord&id=L358176034&from=export	Surgical masks or N95 respirators for the flu?; indexed publication type: Note	Excluded<br>Surgical masks versus N95 respirators, rather than an RSV vaccination intervention.
M3REC001133<br>doi:10.1086/499600	The interferon antagonist NS2 protein of respiratory syncytial virus is an important virulence determinant for humans<br>DOI:10.1086/499600<br>Journal article	Original indexed abstract, Methods or Design<br>https://doi.org/10.1086/499600	Three NS2 gene-deleted RSV vaccine candidates were studied: rA2cpΔNS2, rA2cp248/404ΔNS2, and rA2cp530/1009ΔNS2. rA2cpΔNS2, which had the fewest attenuating mutations, was evaluated in adults and RSV-seropositive children. rA2cp248/404ΔNS2 and rA2cp530/1009ΔNS2 were evaluated in adults and RSV-seropositive and RSV-seronegative children.	Excluded<br>NS2-deleted live RSV vaccine candidates, rather than RSVPreF3 OA.

Original record	Corrected report identity	Material and source	Evidence supporting assessment	Disposition and trial linkage
M3REC001147<br>doi:10.1016/s0264-410x(01)00261-4	The WHO Vaccine Trial Registry<br>DOI:10.1016/s0264-410x(01)00261-4<br>Registry methods review	Original indexed abstract<br>https://doi.org/10.1016/s0264-410x(01)00261-4	The WHO Vaccine Trial Registry prospectively registers clinical vaccine studies supported by WHO.	Excluded<br>Descriptive report on the WHO Vaccine Trial Registry; not an individual eligible trial report.
M3REC001148<br>doi:10.1021/acs.bioconjchem.6b00370	Thermoresponsive Polymer Nanoparticles Co-deliver RSV F Trimers with a TLR-7/8 Adjuvant<br>DOI:10.1021/acs.bioconjchem.6b00370<br>Journal article	Original indexed abstract<br>https://doi.org/10.1021/acs.bioconjchem.6b00370	Structure-based vaccine design has been used to develop immunogens that display conserved neutralization sites on pathogens such as HIV-1, respiratory syncytial virus (RSV), and influenza.	Excluded<br>Preclinical RSV F-trimer and TLR-7/8-adjuvant nanoparticle study; not clinical coadministration of two vaccines.
M3REC001172<br>pmid:12387157	Vaccination and extreme prematurity<br>Report:M3REC001172<br>Journal article	Original title and bibliographic publication type<br>https://www.embase.com/search/results?subaction=viewrecord&id=L35118498&from=export	Vaccination and extreme prematurity; indexed publication type: Conference Paper	Excluded<br>Vaccination in extremely premature infants, outside the adult RSVPreF3 OA population.
M3REC001174<br>pmid:4080007	Vaccination of hospital staff against hepatitis B; the intramuscular method vs combined intradermal/intramuscular method<br>Report:M3REC001174<br>Journal article	Original title and bibliographic publication type<br>https://www.embase.com/search/results?subaction=viewrecord&id=L16206692&from=export	Vaccination of hospital staff against hepatitis B; the intramuscular method vs combined intradermal/intramuscular method	Excluded<br>Hepatitis B vaccination routes in hospital staff, rather than RSVPreF3 OA coadministration.
M3REC001182<br>doi:10.1055/a-2603-9028	Vaccinations in Patients with Diabetes: Current Recommendations<br>DOI:10.1055/a-2603-9028<br>Recommendations	Original indexed abstract<br>https://doi.org/10.1055/a-2603-9028	The German Standing Committee on Vaccination (STIKO) recommends that people with diabetes receive all standard vaccinations and relevant indication-based vaccines in a timely manner.	Excluded<br>Vaccination recommendations for people with diabetes; not an original randomized trial report.
M3REC001190<br>doi:10.1111/jgs.70425	Vaccine Status of Older Adults and Trust in Federal Institutions<br>DOI:10.1111/jgs.70425#M3REC001190<br>Conference abstract	Original indexed abstract<br>https://doi.org/10.1111/jgs.70425	Results Among older adults surveyed, 38.1% reported receiving a COVID-19 vaccine and 43.5% a flu vaccine since August 2024, while 24.9% reported ever receiving an RSV vaccine.	Excluded<br>Survey of vaccination status and trust in institutions, rather than a randomized vaccine-schedule comparison.
M3REC001192<br>doi:10.3928/0090-4481-20001001-10	Vaccines and otitis media<br>DOI:10.3928/0090-4481-20001001-10<br>Review	Original title and bibliographic publication type<br>https://doi.org/10.3928/0090-4481-20001001-10	Vaccines and otitis media; indexed publication type: Review	Excluded<br>Review of vaccines and otitis media; not an original eligible randomized trial report.

Original record	Corrected report identity	Material and source	Evidence supporting assessment	Disposition and trial linkage
M3REC001197<br>pmid:17944274	VII international symposium on respiratory viral infections<br>Report:M3REC001197<br>Symposium report	Original indexed abstract<br>https://www.embase.com/search/results?subaction=viewrecord&id=L47235509&from=export	Topics covered in invited lectures included detection of novel respiratory viral pathogens and viral evolution, characterization of the 1918 pandemic virus, human metapneumovirus infections, human respiratory epithelial cultures for studying viral pathogenesis, the role of respiratory viruses in the pathogenesis of asthma, influenza-bacterial interactions, advances in generating vaccine candidates against global respiratory threats like avian influenza and SARS, antiviral resistance surveillance in influenza viruses, and a mini-symposium on advances in viral diagnostics.	Excluded<br>Multi-topic symposium report on respiratory viral infections; not an individual eligible trial report.
M3REC001206<br>doi:10.1016/j.arbres.2025.05.010	Why RSV Infection Matter in Older Adults? The Urgent Need for Vaccination<br>DOI:10.1016/j.arbres.2025.05.010<br>Editorial	Original title and bibliographic publication type<br>https://doi.org/10.1016/j.arbres.2025.05.010	Why RSV Infection Matter in Older Adults? The Urgent Need for Vaccination; indexed publication type: Editorial	Excluded<br>Editorial on RSV vaccination in older adults; not an original randomized trial report.

The records comprise 66 excluded reports and five associated reports of the six included trials. Original record identifiers are retained. Material type and source are specified for each eligibility or linkage decision. The underlying full evidence fields are provided in the accompanying CSV.

Table S5. PCV20 participant flow and source definitions

Source / stage	Co-Ad	Seq	Total	Definition	Analytical use
Article Figure 3: screened	—	—	1180	Screened population	Screening flow
Article Figure 3: ineligible	—	—	68	Not eligible before randomization	1180 − 68 = 1112
Article Figure 3: randomized	555	557	1112	Assigned to study groups	Randomized denominator
Article Figure 3: pre-vaccine withdrawals	2	0	2	Withdrawn before exposure	1112 − 2 = 1110
Article Figure 3: exposed	553	557	1110	Received ≥1 study vaccine	Safety denominator
Article Figure 3: RSV PPS	513	383	896	RSV per-protocol cohort	RSV cohort and retention
Article Figure 3: PCV20 PPS	514	497	1011	PCV20 per-protocol cohort	PCV20 cohort and retention
GSK study register: enrollment	—	—	1112	Enrollment field	Agrees with published randomized total

Source / stage	Co-Ad	Seq	Total	Definition	Analytical use
ClinicalTrials.gov: enrollment	—	—	1113	Actual enrollment field	Registry descriptor
ClinicalTrials.gov: started	553	557	1110	Participant-flow milestones	Matches article exposed set
ClinicalTrials.gov: pre-assignment narrative	—	—	1110	Described as eligible and randomized in registry narrative	Publication Figure 3 supplies the randomized denominator

The publication reports 1112 randomized participants and 1110 exposed participants after two withdrawals before vaccination. The GSK study register also lists 1112 participants. ClinicalTrials.gov reports enrollment of 1113 and a started population of 1110; its pre-assignment narrative also describes 1110 participants as randomized. Table 1 reports the publication's randomized population; safety outcomes use the exposed population, and immunogenicity outcomes use the endpoint-specific PPS populations. The reason for the one-participant difference between the ClinicalTrials.gov enrollment field and the published randomized total is not stated in these sources. PPS retention was 896/1112 (80.6%) for RSV and 1011/1112 (90.9%) for PCV20. Sources: Leroux-Roels et al. [15], Results and Figure 3; ClinicalTrials.gov NCT05879107, enrollmentInfo and participantFlowModule (registry record last posted 18 May 2025); GSK study register, study 219276, Enrollment field (https://www.gsk-studyregister.com/trials/219276; accessed 12 September 2026).

Table S6. Detailed characteristics of the included trials.

Characteristic	PCV20 [15] NCT05879107	COVID-19 mRNA [16] NCT06374394	RZV [17] NCT05966090	Seasonal QIV [18] NCT04841577	High-dose QIV [19] NCT05559476	Adjuvanted QIV [20] NCT05568797
Trial registration number	NCT05879107	NCT06374394	NCT05966090	NCT04841577	NCT05559476	NCT05568797
Countries	Belgium, Poland, Spain, USA	USA, Belgium, Netherlands, Spain	Canada, USA	New Zealand, Panama, South Africa	USA	Belgium, Finland, France, Spain, UK
Sites, n	38	24	20	14	48	37
Phase / design	Phase 3, randomized, open-label, non-inferiority	Phase 3, randomized, open-label, non-inferiority	Phase 3, randomized, open-label, non-inferiority	Phase 3, randomized, open-label, non-inferiority	Phase 3, randomized, open-label, non-inferiority	Phase 3, randomized, open-label, non-inferiority
Reported dates (period type)	Study: May 2023–May 2024	Study: 29 Apr 2024–18 Apr 2025	Study: 28 Jul 2023–29 Jul 2024	Enrollment (article): 27 Apr 2021–8 Feb 2022	Study: 20 Oct 2022–15 Aug 2023	Study: 14 Oct 2022–17 Jul 2023
Population; age eligibility	Medically stable adults ≥60 y (median 69; range 60–93)	Adults ≥50 y (mean 63.6); prior COVID vaccination required; immunocompromised excluded	Medically stable adults ≥50 y (mean 63.9); prior RSV/HZ vaccine excluded	Community-dwelling or minimally assisted medically stable adults ≥60 y	Community-dwelling or minimally assisted medically stable adults ≥65 y	Medically stable adults ≥65 y; immunocompromising conditions excluded
Randomized, n	1112 (555/557)	841	530 (265/265)	890 (445/445)	1037	1045 (523/522)
Female, %	56.8 (631/1110)	52.7 (439/833)	55.1 (292/530)	51.5 (456/885)	49.6 (510/1029)	49.3 (515/1045)

Characteristic	PCV20 [15] NCT05879107	COVID-19 mRNA [16] NCT06374394	RZV [17] NCT05966090	Seasonal QIV [18] NCT04841577	High-dose QIV [19] NCT05559476	Adjuvanted QIV [20] NCT05568797
Blinding	Participants/investigators: unblinded; laboratory antibody assessors: blinded. Safety: participant reports and investigator assessment.	Participants/investigators: unblinded; laboratory antibody assessors: blinded. Safety: participant reports and investigator assessment.	Participants/investigators: unblinded; laboratory antibody assessors: blinded. Safety: participant reports and investigator assessment.	Participants/investigators: unblinded; laboratory antibody assessors: blinded. Safety: participant reports and investigator assessment.	Participants/investigators: unblinded; laboratory antibody assessors: blinded. Safety: participant reports and investigator assessment.	Participants/investigators: unblinded; laboratory antibody assessors: blinded. Safety: participant reports and investigator assessment.
Coadministration schedule	Day 1: RSVPreF3 OA + PCV20	Day 1: RSVPreF3 OA + COVID-19 mRNA (XBB.1.5)	Day 1: RSVPreF3 OA + RZV dose 1; Day 61: RZV dose 2	Day 1: RSVPreF3 OA + seasonal QIV	Day 1: RSVPreF3 OA + high-dose QIV	Day 1: RSVPreF3 OA + adjuvanted QIV
Sequential schedule (partner first)	Day 1: PCV20; Day 31: RSVPreF3 OA	Day 1: COVID-19 mRNA (XBB.1.5); Day 31: RSVPreF3 OA	Day 1: RZV dose 1; Day 31: RSVPreF3 OA; Day 61: RZV dose 2	Day 1: seasonal QIV; Day 31: RSVPreF3 OA	Day 1: high-dose QIV; Day 31: RSVPreF3 OA	Day 1: adjuvanted QIV; Day 31: RSVPreF3 OA
Follow-up duration	6 months after last dose; approximately 6 months from Day 1 in Co-Ad and 7 months in Seq	6 months after last dose; approximately 6 months from Day 1 in Co-Ad and 7 months in Seq	6 months after last dose; approximately 8 months from Day 1 in both arms	6 months after last dose; approximately 6 months from Day 1 in Co-Ad and 7 months in Seq	6 months after last dose; approximately 6 months from Day 1 in Co-Ad and 7 months in Seq	6 months after last dose; approximately 6 months from Day 1 in Co-Ad and 7 months in Seq
RSV-A / RSV-B assay and endpoint status	RSV-A and RSV-B neutralizing-antibody titers (ED60); both confirmatory NI endpoints met	RSV-A and RSV-B neutralizing-antibody titers (ED60); both confirmatory NI endpoints met	RSV-A and RSV-B neutralizing-antibody titers (ED60); both confirmatory NI endpoints met	RSV-A/B neutralization (ED60). RSV-A: confirmatory NI met; RSV-B: secondary PPS subset (n = 425)	RSV-A and RSV-B neutralizing-antibody titers (ED60); both confirmatory NI endpoints met	RSV-A/B neutralization (ED60). RSV-A: confirmatory NI met; RSV-B: descriptive after hierarchical testing stopped
Partner primary endpoints	PCV20 OPA GMT ratio for 20 serotypes	SARS-CoV-2 XBB.1.5 pseudotyped neutralization (NT50)	VZV anti-gE GMC ratio (1 month post dose 2)	Influenza HI GMT ratio for 4 strains	Influenza HI GMT ratio for 4 strains	Influenza HI GMT ratio for 4 strains
Non-inferiority margins	RSV 1.5-fold (0.667); PCV20 2-fold (0.5)	RSV 1.5-fold (0.667); COVID 1.5-fold (0.667)	RSV 1.5-fold (0.667); RZV 1.5-fold (0.667)	RSV-A 1.5-fold (0.667); RSV-B descriptive; influenza 1.5-fold (0.667)	RSV 1.5-fold (0.667); influenza 1.5-fold (0.667)	RSV 1.5-fold (0.667); influenza 1.5-fold (0.667)
Immunogenicity analysis sets (coad/seq)	RSV PPS 513/383; PCV20 PPS 514/497	RSV PPS 382/364; COVID PPS 380/381	RSV PPS 240/219; RZV PPS 198/170	Post-RSV PPS flow: 427/397 (Days 31/61); RSV-B subset: 425 total. Influenza PPS: 427/410 (Day 31)	RSV model n: A 459/358; B 459/357. Post-RSV flow: 458/356. Influenza PPS: 458/440 (Table S8)	PPS cohorts: RSV 471/374; influenza 471/400. Assay n varies (Table S8)
Randomized → PPS retention, n (%)	RSV: 896/1112 (80.6%); 216 not retained. PCV20: 1011/1112 (90.9%); 101 not retained.	RSV: 746/841 (88.7%); 95 not retained. COVID: 761/841 (90.5%); 80 not retained.	RSV: 459/530 (86.6%); 71 not retained. RZV: 368/530 (69.4%); 162 not retained.	Post-RSV: 824/890 (92.6%); 66 not retained. Influenza: 837/890 (94.0%); 53 not retained.	Post-RSV flow: 814/1037 (78.5%); 223 not retained. Influenza: 898/1037 (86.6%); 139 not retained.	RSV: 845/1045 (80.9%); 200 not retained. Influenza: 871/1045 (83.3%); 174 not retained.

Characteristic	PCV20 [15] NCT05879107	COVID-19 mRNA [16] NCT06374394	RZV [17] NCT05966090	Seasonal QIV [18] NCT04841577	High-dose QIV [19] NCT05559476	Adjuvanted QIV [20] NCT05568797
Sponsor, funding and role	GSK funded the trial, participated in all stages of conduct and analysis, and funded manuscript development and publication.	GSK funded the trial. GSK-employed authors contributed to design, analysis and interpretation; GSK supported medical writing and publication coordination.	GSK funded the trial, participated in all stages of conduct and analysis, and funded manuscript development and publication.	GSK funded the trial and medical writing. GSK-employed authors contributed to design, methodology, analysis, supervision and manuscript review.	GSK funded the trial and publication fee. GSK-employed authors contributed to study design and data analysis.	GSK funded the trial, participated in all stages of conduct, and funded manuscript development and publication. GSK-employed authors contributed to design and analysis.
Original trial conclusion	PPS: RSV-A/B and PCV20 NI met	PPS: RSV-A/B NI met; COVID-19 XBB.1.5 NI not demonstrated (lower CI 0.662 < 0.667)	PPS: RSV-A/B and RZV NI met	PPS: RSV-A and influenza NI met; RSV-B evaluated descriptively	PPS: RSV-A/B and influenza NI met	PPS: RSV-A NI met; A/H3N2 NI not demonstrated (GMR 0.758, 95% CI 0.654–0.885); RSV-B descriptive
Risk of bias (RSV immunogenicity)	Some concerns	Some concerns	High	High	Some concerns	Some concerns

Counts separated by a slash are coadministration/sequential administration. PPS, per-protocol set; NI, non-inferiority; GMT, geometric mean titer; GMC, geometric mean concentration; OPA, opsonophagocytic assay; HI, hemagglutination inhibition; ED60, 60% effective dilution; NT50, 50% neutralizing titer.

Table S7. Trial-level non-inferiority assessments of RSV neutralizing-antibody responses.

Study (partner vaccine)	RSV-A endpoint status	RSV-A GMR (95% CI)	RSV-B endpoint status	RSV-B GMR (95% CI)	Analysis population	Prespecified RSV NI margin	Original PPS conclusion
PCV20 (NCT05879107) [15]	Co-primary; first testing step	0.943 (0.833–1.064)	Hierarchical second step; confirmatory criteria met	1.000 (0.885–1.124)	RSV PPS (coad 513 / seq 383)	1.5-fold (0.667)	RSV-A and RSV-B NI met
COVID-19 mRNA (NCT06374394) [16]	Primary confirmatory endpoint	0.893 (0.781–1.031)	Primary confirmatory endpoint	0.926 (0.813–1.064)	RSV PPS (coad 382 / seq 364)	1.5-fold (0.667)	RSV-A and RSV-B NI met
RZV (NCT05966090) [17]	Co-primary; first testing step	0.909 (0.714–1.000)	Hierarchical second step; confirmatory criteria met	1.000 (0.833–1.250)	RSV PPS (coad 240 / seq 219)	1.5-fold (0.667)	RSV-A and RSV-B NI met
Seasonal QIV (NCT04841577) [18]	Co-primary	0.787 (0.694–0.893)	Secondary; per-protocol subset (n = 425)	0.787 (0.671–0.926)	RSV PPS (coad 427 / seq 397); RSV-B subset 425	RSV-A: 1.5-fold (0.667); RSV-B: descriptive	RSV-A NI met; RSV-B descriptive (non-confirmatory)
High-dose QIV (NCT05559476) [19]	Co-primary; first testing step	0.847 (0.741–0.962)	Hierarchical second step; confirmatory criteria met	0.980 (0.862–1.124)	PPS model n: RSV-A 459/358; RSV-B 459/357 (Table S8)	1.5-fold (0.667)	RSV-A and RSV-B NI met

Study (partner vaccine)	RSV-A endpoint status	RSV-A GMR (95% CI)	RSV-B endpoint status	RSV-B GMR (95% CI)	Analysis population	Prespecified RSV NI margin	Original PPS conclusion
Adjuvanted QIV (NCT05568797) [20]	Co-primary; first testing step	1.010 (0.893–1.149)	Planned hierarchical second step; evaluated descriptively	0.862 (0.769–0.971)	RSV PPS cohort 471/374; post-dose assay n: A 471/373; B 469/373 (Table S8)	1.5-fold (0.667)	RSV-A NI met; RSV-B descriptive (non-confirmatory)

GMRs are expressed as coadministration/sequential administration. GMR, geometric mean ratio; NI, non-inferiority; PPS, per-protocol set.

Table S8. Immunogenicity estimates by analysis population

Trial and endpoint	PPS n Co-Ad/Seq	PPS GMR (95% CI)	ES n Co-Ad/Seq	ES GMR (95% CI)	Population comparison
PCV20 [15] RSV-A	513/383 (PPS cohort)	0.943 (0.833–1.064)	535/511	0.952 (0.855–1.064)	RSV-A criterion met in both populations.
PCV20 [15] RSV-B	513/383 (PPS cohort)	1.000 (0.885–1.124)	534/512	1.010 (0.909–1.124)	RSV-B criterion met in both populations; additional PPS model differs from main figure (note a).
COVID-19 [16] RSV-A	382/364	0.893 (0.781–1.031)	404/400	0.926 (0.806–1.053)	RSV-A criterion met in both populations.
COVID-19 [16] RSV-B	382/364	0.926 (0.813–1.064)	404/400	0.952 (0.833–1.075)	RSV-B criterion met in both populations.
COVID-19 [16] XBB.1.5 NT50	380/381	0.763 (0.662–0.885)	402/396	0.781 (0.680–0.901)	PPS: NI not demonstrated; ES: numerical criterion met.
RZV [17] RSV-A	240/219 (PPS cohort)	0.909 (0.714–1.000)	—	PPS-only immunogenicity reporting	NI met in PPS; ES is used for safety reporting.
RZV [17] RSV-B	240/219 (PPS cohort)	1.000 (0.833–1.250)	—	PPS-only immunogenicity reporting	NI met in PPS; ES is used for safety reporting.
RZV [17] VZV anti-gE	198/170 (PPS cohort)	0.833 (0.714–0.909)	—	PPS-only immunogenicity reporting	NI met in HZ PPS; ES is used for safety reporting.

Trial and endpoint	PPS n<br>Co-Ad/Seq	PPS GMR<br>(95% CI)	ES n<br>Co-Ad/Seq	ES GMR<br>(95% CI)	Population comparison
Seasonal QIV [18]<br>RSV-A	427/397 (post-RSV PPS flow; Days 31/61)	0.787 (0.694–0.893)	442/443 (ES cohort)	0.787 (0.699–0.885)	Numerical criterion met in both populations.
Seasonal QIV [18]<br>RSV-B	425 total (subset)	0.787 (0.671–0.926)	442/443 (ES cohort)	0.775 (0.662–0.909)	RSV-B is descriptive in both populations.
Seasonal QIV [18]<br>A/H1N1 HI	427/410 (PPS cohort)	0.820 (0.694–0.971)	442/443 (ES cohort)	0.840 (0.714–0.990)	Numerical criterion met in both populations.
Seasonal QIV [18]<br>A/H3N2 HI	427/410 (PPS cohort)	0.855 (0.741–0.980)	442/443 (ES cohort)	0.862 (0.752–0.990)	Numerical criterion met in both populations.
Seasonal QIV [18]<br>B/Victoria HI	427/410 (PPS cohort)	0.909 (0.794–1.053)	442/443 (ES cohort)	0.909 (0.794–1.042)	Numerical criterion met in both populations.
Seasonal QIV [18]<br>B/Yamagata HI	427/410 (PPS cohort)	0.855 (0.758–0.962)	442/443 (ES cohort)	0.862 (0.763–0.971)	Numerical criterion met in both populations.
High-dose QIV [19]<br>RSV-A	459/358	0.847 (0.741–0.962)	493/480	0.800 (0.714–0.909)	Numerical criterion met in both populations.
High-dose QIV [19]<br>RSV-B	459/357	0.980 (0.862–1.124)	493/479	0.952 (0.847–1.064)	Numerical criterion met in both populations.
High-dose QIV [19]<br>A/H1N1 HI	452/435	1.075 (0.926–1.250)	484/482	1.053 (0.909–1.220)	Numerical criterion met in both populations.
High-dose QIV [19]<br>A/H3N2 HI	458/441	1.020 (0.877–1.190)	491/490	1.010 (0.870–1.176)	Numerical criterion met in both populations.
High-dose QIV [19]<br>B/Victoria HI	458/441	1.053 (0.971–1.136)	491/490	1.053 (0.980–1.136)	Numerical criterion met in both populations.
High-dose QIV [19]<br>B/Yamagata HI	456/441	1.087 (0.980–1.190)	489/490	1.053 (0.962–1.163)	Numerical criterion met in both populations.
Adjuvanted QIV [20]<br>RSV-A	471/374 (cohort); 471/373 (post-dose)	1.010 (0.893–1.149)	519/499	0.971 (0.862–1.087)	Numerical criterion met in both populations.

Trial and endpoint	PPS n Co-Ad/Seq	PPS GMR (95% CI)	ES n Co-Ad/Seq	ES GMR (95% CI)	Population comparison
Adjuvanted QIV [20] RSV-B	471/374 (cohort); 469/373 (post-dose)	0.862 (0.769–0.971)	517/499	0.847 (0.758–0.943)	RSV-B remains descriptive after H3N2 failure.
Adjuvanted QIV [20] A/H1N1 HI	471/400 (cohort); 440/399 (post-dose)	0.962 (0.847–1.099)	465/448	0.943 (0.833–1.075)	Numerical criterion met in both populations.
Adjuvanted QIV [20] A/H3N2 HI	471/400 (cohort); 442/400 (post-dose)	0.758 (0.654–0.885)	474/453	0.730 (0.633–0.847)	H3N2 criterion not met in either population.
Adjuvanted QIV [20] B/Victoria HI	471/400 (cohort); 442/400 (post-dose)	1.031 (0.943–1.111)	474/453	1.031 (0.952–1.111)	Numerical criterion met in both populations.
Adjuvanted QIV [20] B/Yamagata HI	471/400 (cohort); 442/400 (post-dose)	0.962 (0.885–1.053)	473/453	0.962 (0.885–1.042)	Numerical criterion met in both populations.
PCV20 [15] 20 OPA serotypes	514/497 (PCV20 PPS)	0.676–0.847 (range)	—	PPS-only immunogenicity reporting	All 20 serotype criteria met in PPS.

PPS, per-protocol set; ES, exposed set; Seq, sequential administration. Ratios are Co-Ad/Seq. ES retains the original trial terminology and includes participants who received at least one study intervention. Cohort n describe the reported analysis set; post-dose n describe participants with measurements at that time point; model n describe the reported adjusted analysis. The subtype-specific totals in Tables 3 and S5 are 4,586 for RSV-A and 4,184 for RSV-B. Sources: PCV20, main Figure 4 and Supplementary Table 4 [15]; COVID-19, main Figure 4 [16]; RZV, main Figure 4 and Supplementary Table 2 [17]; seasonal QIV, main Figure 3, Supplementary Figure 1 and Supplementary Table 2 [18]; high-dose QIV, Supplementary Table S2 [19]; adjuvanted QIV, main Figure 3 and Supplementary Tables 3–4 [20]. (a) The additional PCV20 PPS analysis in Supplementary Table 4 reports model n 512/382 for RSV-A and 511/382 for RSV-B, with GMRs of 0.943 (0.833–1.064) and 1.010 (0.893–1.136); the main-figure estimates used in the synthesis are retained in this table. For the high-dose-QIV trial, Supplementary Table S2 reports adjusted-model counts of 459/358 for RSV-A and 459/357 for RSV-B; the original high-dose-QIV article's Results and Figure 2 report post-RSV PPS flow counts of 458/356. Table S8 identifies these counts by their source and analysis definition. Adjuvanted-QIV post-dose counts come from Supplementary Table 4 and are distinct from its cohort totals. For seasonal QIV, post-RSV PPS flow counts were 427/397 at Days 31/61, whereas influenza PPS counts were 427/410 at Day 31. Seasonal-QIV ES labels describe the cohort; RSV-B is descriptive. Trial-level numerical criteria are interpreted within the original analysis populations and testing sequence. RSV-B was confirmatory after successful first-step assessments in the PCV20, RZV and high-dose-QIV trials. It was a secondary subset endpoint in the seasonal-QIV trial and descriptive after the first-step A/H3N2 criterion was not met in the adjuvanted-QIV trial.

Table S9. RSV antibody geometric mean increases and between-schedule GMRs

Trial [reference]	Endpoint / population	Co-Ad MGI (95% CI)	Sequential MGI (95% CI)	Adjusted GMR (95% CI)	Source
PCV20 [15]	RSV-A; neutralization; RSV PPS	8.3 (7.6–9.1)	9.3 (8.3–10.3)	0.943 (0.833–1.064)	Figure 5
PCV20 [15]	RSV-B; neutralization; RSV PPS	9.4 (8.6–10.2)	9.1 (8.2–10.1)	1.000 (0.885–1.124)	Figure 5
COVID-19 mRNA [16]	RSV-A; neutralization; RSV PPS	8.31 (7.46–9.26)	10.21 (9.20–11.33)	0.893 (0.781–1.031)	Figure 5

Trial [reference]	Endpoint / population	Co-Ad MGI (95% CI)	Sequential MGI (95% CI)	Adjusted GMR (95% CI)	Source
COVID-19 mRNA [16]	RSV-B; neutralization; RSV PPS	6.79 (6.08–7.59)	8.19 (7.35–9.12)	0.926 (0.813–1.064)	Figure 5
RZV [17]	RSV-A; neutralization; RSV PPS	8.6 (7.4–9.8)	9.9 (8.6–11.5)	0.909 (0.714–1.000)	Figure 5
RZV [17]	RSV-B; neutralization; RSV PPS	9.0 (7.9–10.2)	8.7 (7.5–10.1)	1.000 (0.833–1.250)	Figure 5
Seasonal QIV [18]	RSV-A; neutralization; PPS	9.6 (8.7–10.6)	12.9 (11.7–14.3)	0.787 (0.694–0.893)	Results, pp. e66–e67
Seasonal QIV [18]	RSV-B; neutralization; PPS subset	7.67 (6.8–8.7)	9.23 (8.0–10.6)	0.787 (0.671–0.926)	Results, pp. e66–e67
High-dose QIV [19]	RSV-A; neutralization; PPS	5.59 (5.02–6.23)	6.75 (5.93–7.68)	0.847 (0.741–0.962)	Results, p. 1798
High-dose QIV [19]	RSV-B; neutralization; PPS	5.19 (4.69–5.75)	5.29 (4.65–6.03)	0.980 (0.862–1.124)	Results, p. 1798
Adjuvanted QIV [20]	RSV-A; neutralization; RSV PPS	8.50 (7.79–9.27)	7.58 (6.82–8.42)	1.010 (0.893–1.149)	Figure 4
Adjuvanted QIV [20]	RSV-B; neutralization; RSV PPS	7.11 (6.55–7.72)	7.46 (6.74–8.25)	0.862 (0.769–0.971)	Figure 4

MGI, mean geometric increase; GMFR, geometric mean fold rise; GMR, geometric mean ratio; PPS, per-protocol set. MGIs and GMFRs describe within-participant post-/pre-vaccination changes. Adjusted GMRs compare coadministration with sequential administration at the post-vaccination assessment, using the original trial models. Values retain the precision reported in the source; GMRs are expressed as coadministration/sequential. Sampling was approximately 1 month after the RSV vaccine dose. Trial-specific population and assay counts are detailed in Table S8. RSV-B in the seasonal-QIV trial was assessed in a per-protocol subset of 425 participants; RSV-B in the adjuvanted-QIV trial was descriptive after hierarchical testing stopped. The seasonal-QIV RSV-A PPS flow comprises 427 Co-Ad and 397 sequential participants at their post-RSV visits.

Table S10. Reported absolute pre- and post-vaccination antibody titers and concentrations

Trial [reference]	Endpoint / measure	Co-Ad pre → post	Sequential pre → post	Sampling / population	Source
PCV20 [15]	RSV-A; GMT (ED60)	1026.4 → 8533.5	960.8 → 8880.9	Pre-dose to 1 month post-dose; RSV PPS	Results
PCV20 [15]	RSV-B; GMT (ED60)	1008.8 → 9393.5	1053.6 → 9760.0	Pre-dose to 1 month post-dose; RSV PPS	Results
COVID-19 mRNA [16]	RSV-A; GMT (ED60)	973.4 → 8119.1	842.2 → 8407.1	Pre-dose to 1 month post-dose; RSV PPS	Figure 5
COVID-19 mRNA [16]	RSV-B; GMT (ED60)	1121.5 → 7537.1	945.8 → 7588.9	Pre-dose to 1 month post-dose; RSV PPS	Figure 5
COVID-19 mRNA [16]	SARS-CoV-2 Omicron XBB.1.5; GMT (NT50)	244.6 → 1030.4	250.0 → 1407.3	Pre-dose to 1 month post-dose; COVID PPS	Figure 5
RZV [17]	RSV-A; GMT (ED60)	1005.4 → 8553.3	961.2 → 9689.4	Pre-dose to 1 month post-dose; RSV PPS	Figure 5

Trial [reference]	Endpoint / measure	Co-Ad pre → post	Sequential pre → post	Sampling / population	Source
RZV [17]	RSV-B; GMT (ED60)	1148.8 → 10521.3	1167.9 → 10352.2	Pre-dose to 1 month post-dose; RSV PPS	Figure 5
RZV [17]	VZV anti-gE; GMC (mIU/mL)	1739.4 → 50235.2	1492.2 → 61204.7	Pre-dose 1 to 1 month post-dose 2; HZ PPS	Figure 5

GMT, geometric mean titer; GMC, geometric mean concentration. These are the numerically reported unadjusted arm-level values from the original trial reports. GMTs/GMCs summarize available samples at each visit, whereas MGIs use participants with paired observations and adjusted GMRs use the trial-specific models. In the COVID-19 trial, RSV GMT counts (pre/post) were 406/388 for Co-Ad and 389/365 for sequential; SARS-CoV-2 GMT counts were 405/387 and 404/389. In the RZV trial, RSV GMT counts were 261/247 and 249/219; anti-gE GMC counts were 261/198 and 262/170. Thus, the displayed pre- and post-vaccination means can have different sample sizes. Antibody magnitudes are interpreted within each assay and vaccine product. Paired MGIs and adjusted GMRs are presented in Tables S9 and S15.

Table S11. Heterogeneity and variance components across working correlations

Working ρ	Q (df = 11)	P value	Study variance	Endpoint variance	I² proxy (%)
0	17.710	0.088566	0.00242164	0.00097852	39.3
0.5	20.738	0.036193	0.00019690	0.00290509	37.2
0.8	39.493	0.000044	0.00000186	0.00384777	42.3

The approximate subtype-specific 95% prediction intervals were 0.745–1.076 for RSV-A and 0.776–1.096 for RSV-B. Approximate joint model-based 95% prediction intervals at $\rho = 0$, 0.5 and 0.8 are 0.798–1.032, 0.802–1.027 and 0.791–1.041, respectively. The interval construction and prediction target are specified in the Supplementary Methods. Pooled means and CR2 intervals change little across these correlations; Q and the variance partition vary with the covariance assumption. Subtype models give RSV-A $Q(5) = 8.937$, $\tau^2 = 0.003788$ and $I^2 = 45.0\%$, and RSV-B $Q(5) = 8.197$, $\tau^2 = 0.003082$ and $I^2 = 38.1\%$.

Table S12. Non-inferiority assessments of partner-vaccine antibody responses.

Trial and antibody endpoint	Endpoint status	Analysis population, n	GMR (95% CI)	NI boundary	Original NI conclusion	GRADE certainty
COVID-19 mRNA, SARS-CoV-2 XBB.1.5 (NT50) [16]	Primary	PPS: 380/381	0.763 (0.662–0.885)	0.667	Not demonstrated	Moderate
RZV, VZV anti-gE (ELISA) [17]	Primary	PPS cohort: 198/170	0.833 (0.714–0.909)	0.667	Met	Low
Seasonal QIV, A/H1N1 HI [18]	Primary	Post-dose PPS: 427/410	0.820 (0.694–0.971)	0.667	Met	Moderate
Seasonal QIV, A/H3N2 HI [18]	Primary	Post-dose PPS: 427/410	0.855 (0.741–0.980)	0.667	Met	Moderate
Seasonal QIV, B/Victoria HI [18]	Primary	Post-dose PPS: 427/410	0.909 (0.794–1.053)	0.667	Met	Low

Trial and antibody endpoint	Endpoint status	Analysis population, n	GMR (95% CI)	NI boundary	Original NI conclusion	GRADE certainty
Seasonal QIV, B/Yamagata HI [18]	Primary	Post-dose PPS: 427/410	0.855 (0.758–0.962)	0.667	Met	Moderate
High-dose QIV, A/H1N1 HI [19]	Primary	PPS: 452/435	1.075 (0.926–1.250)	0.667	Met	Low
High-dose QIV, A/H3N2 HI [19]	Primary	PPS: 458/441	1.020 (0.877–1.190)	0.667	Met	Low
High-dose QIV, B/Victoria HI [19]	Primary	PPS: 458/441	1.053 (0.971–1.136)	0.667	Met	Low
High-dose QIV, B/Yamagata HI [19]	Primary	PPS: 456/441	1.087 (0.980–1.190)	0.667	Met	Low
Adjuvanted QIV, A/H1N1 HI [20]	Primary; first testing step	Post-dose PPS: 440/399	0.962 (0.847–1.099)	0.667	Met	Low
Adjuvanted QIV, A/Darwin H3N2 HI [20]	Primary; first testing step[a]	Post-dose PPS: 442/400	0.758 (0.654–0.885)	0.667	Not demonstrated	Moderate
Adjuvanted QIV, B/Victoria HI [20]	Primary; first testing step	Post-dose PPS: 442/400	1.031 (0.943–1.111)	0.667	Met	Low
Adjuvanted QIV, B/Yamagata HI [20]	Primary; first testing step	Post-dose PPS: 442/400	0.962 (0.885–1.053)	0.667	Met	Low
PCV20 OPA serotype 1 [15]	Primary	PPS cohort: 514/497	0.68 (0.55, 0.82)	0.500	Met	Moderate
PCV20 OPA serotype 3 [15]	Primary	PPS cohort: 514/497	0.76 (0.65, 0.89)	0.500	Met	Moderate
PCV20 OPA serotype 4 [15]	Primary	PPS cohort: 514/497	0.75 (0.63, 0.89)	0.500	Met	Moderate
PCV20 OPA serotype 5 [15]	Primary	PPS cohort: 514/497	0.73 (0.58, 0.93)	0.500	Met	Moderate
PCV20 OPA serotype 6A [15]	Primary	PPS cohort: 514/497	0.73 (0.59, 0.89)	0.500	Met	Moderate
PCV20 OPA serotype 6B [15]	Primary	PPS cohort: 514/497	0.71 (0.59, 0.85)	0.500	Met	Moderate
PCV20 OPA serotype 7F [15]	Primary	PPS cohort: 514/497	0.75 (0.65, 0.86)	0.500	Met	Moderate
PCV20 OPA serotype 8 [15]	Primary	PPS cohort: 514/497	0.72 (0.61, 0.85)	0.500	Met	Moderate
PCV20 OPA serotype 9V [15]	Primary	PPS cohort: 514/497	0.81 (0.68, 0.95)	0.500	Met	Moderate
PCV20 OPA serotype 10A [15]	Primary	PPS cohort: 514/497	0.82 (0.69, 0.97)	0.500	Met	Moderate
PCV20 OPA serotype 11A [15]	Primary	PPS cohort: 514/497	0.85 (0.70, 1.02)	0.500	Met	Low
PCV20 OPA serotype 12F [15]	Primary	PPS cohort: 514/497	0.83 (0.69, 1.01)	0.500	Met	Low
PCV20 OPA serotype 14 [15]	Primary	PPS cohort: 514/497	0.70 (0.60, 0.83)	0.500	Met	Moderate
PCV20 OPA serotype 15B [15]	Primary	PPS cohort: 514/497	0.69 (0.57, 0.82)	0.500	Met	Moderate
PCV20 OPA serotype 18C [15]	Primary	PPS cohort: 514/497	0.78 (0.65, 0.94)	0.500	Met	Moderate

Trial and antibody endpoint	Endpoint status	Analysis population, n	GMR (95% CI)	NI boundary	Original NI conclusion	GRADE certainty
PCV20 OPA serotype 19A [15]	Primary	PPS cohort: 514/497	0.72 (0.62, 0.83)	0.500	Met	Moderate
PCV20 OPA serotype 19F [15]	Primary	PPS cohort: 514/497	0.75 (0.64, 0.89)	0.500	Met	Moderate
PCV20 OPA serotype 22F [15]	Primary	PPS cohort: 514/497	0.75 (0.62, 0.89)	0.500	Met	Moderate
PCV20 OPA serotype 23F [15]	Primary	PPS cohort: 514/497	0.73 (0.58, 0.92)	0.500	Met	Moderate
PCV20 OPA serotype 33F [15]	Primary	PPS cohort: 514/497	0.71 (0.61, 0.83)	0.500	Met	Moderate

GMRs and participant counts are presented as coadministration/sequential administration. [a] Failure of the A/H3N2 criterion precluded confirmatory testing of RSV-B. GRADE judgments are detailed in Table S19. GMR, geometric mean ratio; NI, non-inferiority; PPS, per-protocol set.

Table S13. GRADE Summary of Findings.

Outcome	Studies / data	Effect	95% CI	Approximate 95% PI	Certainty	Interpretation
RSV-A neutralizing antibody (Figure 2A)	6 randomized trials; 4,586 participants	GMR 0.895	0.816–0.983	0.745–1.076	Low	Lower mean RSV-A response with coadministration.
RSV-B neutralizing antibody (Figure 2B)	6 randomized trials; 4,184 participants	GMR 0.922	0.837–1.016	0.776–1.096	Very low	Lower RSV-B point estimate; CI includes 1.
Exploratory joint RSV neutralizing antibody at ~1 month (Figure 3)	6 RCT families; 12 correlated RSV-A/B rows	GMR 0.908	0.841-0.981	0.802-1.027	Low	Lower RSV neutralizing-antibody responses with coadministration (GMR 0.908, 95% CI 0.841–0.981); the clinical significance of this difference is uncertain.
Exploratory cross-product influenza A/H1N1 HI at ~1 month	3 RCT families; QIV products differ	GMR 0.950	0.683-1.322	0.542-1.665	Very low	Indirect pooling across standard/high-dose/adjuvanted QIV; wide uncertainty.
SAE through study end (Figure 5B)	6 RCT families; exposed-set arms (2,716 per strategy)	RR 0.831	0.530-1.303	0.434-1.590	Low	The estimate is imprecise and compatible with both lower and higher risk.
Death through study end (Figure 5D)	6 randomized trials; 5,432 participants; 2 trials with zero deaths in both groups, retained with continuity correction	RR 0.475	0.174-1.297	0.174-1.297	Very low	The estimate is imprecise and compatible with both lower and higher mortality.
30-day any unsolicited AE (Figure 5A)	6 RCT families; exposed-set arms (2,716 per strategy)	RR 0.729	0.602-0.884	0.512-1.038	Low	Cumulative schedule-level reporting; sequential arms contributed more reporting windows in all six trials.

Note: Review question. In adults receiving RSVPreF3 OA with an influenza or other indicated vaccine, does same-day co-administration versus the prespecified sequential schedule alter RSV immunogenicity, partner-vaccine immunogenicity, and safety? Registration. PROSPERO CRD420261470812. Methods. The exploratory joint RSV estimate is the REML three-level model (working rho = 0.5; 12 correlated RSV-A/RSV-B estimates nested in six randomized trials) with CR2/Satterthwaite interval. Separate RSV-A and RSV-B estimates use REML with Hartung–Knapp intervals. Binary outcomes use a REML two-level model with Hartung-Knapp intervals. Ratio directions are Co-Ad/Sequential; values below 1 indicate a lower co-administration value for GMR or a lower proportion of participants with the specified outcome for RR. I-squared for the multilevel joint outcome is an I-squared proxy. Absolute-frequency context: the observed numbers of participants with at least one unsolicited AE were 426/2,716 with coadministration and 588/2,716 with sequential administration. Corresponding counts were 86/2,716 versus 105/2,716 for SAEs and 5/2,716 versus 16/2,716 for deaths. These are crude observed proportions; pooled RRs and their confidence intervals are presented in the table. The unsolicited AE comparison reflects unequal schedule-specific reporting windows. Certainty footnotes: RSV totals are 4,586 for RSV-A and 4,184 for RSV-B, using source-defined cohort, post-dose assay or adjusted-model counts (Table S8). The subtype populations overlap in the joint analysis. Randomized evidence starts at high certainty. RSV certainty was downgraded for PPS selection, missing outcome data and between-trial variation. RSV-B was additionally downgraded for imprecision because its Hartung–Knapp CI included both lower and higher antibody responses. H1N1 was also downgraded for combining vaccine formulations and for a CI spanning lower and higher responses. Unsolicited AE certainty was downgraded for missing follow-up, open-label reporting and indirectness: unequal observation intervals provide indirect evidence about comparative safety over a common follow-up period. All six trial risk ratios were below 1 with overlapping CIs (I-squared 40.1%); inconsistency was not rated serious. There were 1,014 participants with events among 5,432 exposed participants, and the mean RR CI was 0.602–0.884; imprecision was not rated serious for cumulative reporting. SAE certainty was downgraded for ascertainment/follow-up concerns and imprecision. Death was downgraded for ascertainment/follow-up concerns and very serious imprecision (21 deaths). No additional publication-bias downgrade was applied after considering registration, published primary non-inferiority failures and sponsor involvement; all trials were GSK-funded. Product-specific antibody assessments are provided in Table S19.

Table S13 (continued). GRADE domain judgments for the main outcomes.

Outcome	Risk of bias	Inconsistency	Indirectness	Imprecision	Publication bias	GRADE certainty
RSV-A neutralizing antibody (GMR)	Serious (−1): PPS selection and missing outcome data	Serious (−1): trial estimates vary	Not serious for antibody outcome	Not serious for mean antibody ratio	No added downgrade (note)	Low
RSV-B neutralizing antibody (GMR)	Serious (−1): PPS selection and missing outcome data	Serious (−1): trial estimates vary	Not serious for antibody outcome	Serious (−1): Hartung–Knapp CI crosses 1	No added downgrade (note)	Very low
Exploratory joint RSV neutralizing antibody (GMR)	Serious (−1): PPS selection and missing outcome data	Serious (−1): variation across trials and subtypes	Not serious	Not serious for mean antibody ratio	No added downgrade (note)	Low
Serious adverse events (RR)	Serious (−1): incomplete follow-up	Not serious	Not serious	Serious (−1)	No added downgrade (note)	Low
Death (RR)	Serious (−1): incomplete follow-up	Not interpretable (rare events)	Not serious	Very serious (−2)	No added downgrade (note)	Very low

Outcome	Risk of bias	Inconsistency	Indirectness	Imprecision	Publication bias	GRADE certainty
30-day unsolicited adverse events (RR)	Serious (−1): missing follow-up and open-label reporting	Not serious: same direction; overlapping CIs	Serious (−1): unequal observation intervals	Not serious: 1,014 participants with at least one event; CI excludes 1	No added downgrade (note)	Low

Publication bias was assessed qualitatively using trial registration, outcome reporting and sponsorship; no additional downgrade was applied. GRADE, Grading of Recommendations Assessment, Development and Evaluation; PPS, per-protocol set; CI, confidence interval.

Table S14. PCV20 serotype-specific OPA GMRs (Leroux-Roels et al., single study): coadministration versus sequential administration. Opsonophagocytic assay (OPA) geometric mean ratios (GMRs) with 95% CIs. Serotype-specific estimates are shown separately.

Serotype	GMR (95% CI)
1	0.68 (0.55, 0.82)
3	0.76 (0.65, 0.89)
4	0.75 (0.63, 0.89)
5	0.73 (0.58, 0.93)
6A	0.73 (0.59, 0.89)
6B	0.71 (0.59, 0.85)
7F	0.75 (0.65, 0.86)
8	0.72 (0.61, 0.85)
9V	0.81 (0.68, 0.95)
10A	0.82 (0.69, 0.97)
11A	0.85 (0.70, 1.02)
12F	0.83 (0.69, 1.01)
14	0.70 (0.60, 0.83)
15B	0.69 (0.57, 0.82)
18C	0.78 (0.65, 0.94)
19A	0.72 (0.62, 0.83)
19F	0.75 (0.64, 0.89)
22F	0.75 (0.62, 0.89)
23F	0.73 (0.58, 0.92)
33F	0.71 (0.61, 0.83)

Note: GMR values are 1/(control/coadministration OPA ratio) as reported; GMR < 1 indicates a numerically lower OPA response with coadministration.

Table S15. Partner-vaccine antibody geometric mean increases and between-schedule GMRs

Trial [reference]	Endpoint / population	Co-Ad MGI or GMFR (95% CI)	Sequential MGI or GMFR (95% CI)	Adjusted GMR (95% CI)	Source
COVID-19 mRNA [16]	SARS-CoV-2 Omicron XBB.1.5; pseudotyped virus neutralization; COVID PPS	4.25 (3.73–4.85)	5.46 (4.74–6.30)	0.763 (0.662–0.885)	Figure 5
RZV [17]	VZV anti-gE; ELISA; HZ PPS	28.2 (23.7–33.7)	43.2 (35.0–53.5)	0.833 (0.714–0.909)	Figure 5
High-dose QIV [19]	A/Darwin/6/2021 H3N2; HI; PPS	6.20 (5.49–7.00)	5.87 (5.20–6.62)	1.020 (0.877–1.190)	Results, p. 1798
High-dose QIV [19]	A/Victoria/2570/2019 H1N1; HI; PPS	5.57 (4.81–6.46)	4.53 (3.94–5.21)	1.075 (0.926–1.250)	Results, p. 1798
High-dose QIV [19]	B/Austria/1359417/2021 Victoria; HI; PPS	2.92 (2.69–3.18)	2.71 (2.51–2.93)	1.053 (0.971–1.136)	Results, p. 1798
High-dose QIV [19]	B/Phuket/3073/2013 Yamagata; HI; PPS	3.13 (2.85–3.44)	2.87 (2.62–3.14)	1.087 (0.980–1.190)	Results, p. 1798
Adjuvanted QIV [20]	A/Darwin H3N2; HI; FLU PPS	5.10 (4.60–5.66)	6.87 (6.12–7.71)	0.758 (0.654–0.885)	Figure 4
Adjuvanted QIV [20]	A/Victoria H1N1; HI; FLU PPS	3.85 (3.40–4.35)	3.79 (3.34–4.31)	0.962 (0.847–1.099)	Figure 4
Adjuvanted QIV [20]	B/Victoria; HI; FLU PPS	1.89 (1.77–2.03)	1.84 (1.71–1.98)	1.031 (0.943–1.111)	Figure 4
Adjuvanted QIV [20]	B/Yamagata; HI; FLU PPS	1.94 (1.82–2.07)	2.11 (1.97–2.27)	0.962 (0.885–1.053)	Figure 4
Adjuvanted QIV [20]	A/Darwin H3N2; microneutralization; FLU PPS (post hoc)	3.55 (3.21–3.92)	4.31 (3.86–4.82)	0.813 (0.704–0.943)	Figure 4
PCV20 [15]	Serotype 1; OPA; PCV20 PPS	5.5 (4.8–6.4)	8.3 (7.1–9.6)	0.676 (0.552–0.820)	Figure 5B
PCV20 [15]	Serotype 3; OPA; PCV20 PPS	4.0 (3.5–4.5)	5.2 (4.6–5.9)	0.763 (0.649–0.893)	Figure 5B
PCV20 [15]	Serotype 4; OPA; PCV20 PPS	28.7 (23.9–34.4)	37.6 (30.7–46.1)	0.746 (0.633–0.885)	Figure 5B
PCV20 [15]	Serotype 5; OPA; PCV20 PPS	14.7 (12.4–17.5)	19.8 (16.6–23.6)	0.735 (0.585–0.926)	Figure 5B
PCV20 [15]	Serotype 6A; OPA; PCV20 PPS	31.5 (25.8–38.6)	35.3 (28.4–44.0)	0.730 (0.592–0.893)	Figure 5B
PCV20 [15]	Serotype 6B; OPA; PCV20 PPS	15.7 (13.2–18.6)	21.5 (17.9–25.8)	0.709 (0.588–0.855)	Figure 5B
PCV20 [15]	Serotype 7F; OPA; PCV20 PPS	17.9 (14.6–21.9)	26.7 (21.4–33.3)	0.746 (0.645–0.862)	Figure 5B
PCV20 [15]	Serotype 8; OPA; PCV20 PPS	20.7 (17.1–24.9)	28.0 (23.1–34.0)	0.725 (0.610–0.855)	Figure 5B
PCV20 [15]	Serotype 9V; OPA; PCV20 PPS	7.6 (6.3–9.1)	10.5 (8.8–12.7)	0.806 (0.680–0.952)	Figure 5B
PCV20 [15]	Serotype 10A; OPA; PCV20 PPS	13.2 (11.1–15.8)	16.9 (14.1–20.3)	0.820 (0.694–0.971)	Figure 5B
PCV20 [15]	Serotype 11A; OPA; PCV20 PPS	15.1 (12.3–18.6)	15.1 (12.2–18.6)	0.847 (0.704–1.020)	Figure 5B
PCV20 [15]	Serotype 12F; OPA; PCV20 PPS	64.7 (52.3–80.0)	74.1 (59.6–92.0)	0.833 (0.685–1.010)	Figure 5B

Trial [reference]	Endpoint / population	Co-Ad MGI or GMFR (95% CI)	Sequential MGI or GMFR (95% CI)	Adjusted GMR (95% CI)	Source
PCV20 [15]	Serotype 14; OPA; PCV20 PPS	5.8 (4.9–6.8)	8.3 (7.0–9.9)	0.704 (0.599–0.826)	Figure 5B
PCV20 [15]	Serotype 15B; OPA; PCV20 PPS	14.2 (11.8–17.2)	19.6 (16.0–24.0)	0.685 (0.568–0.820)	Figure 5B
PCV20 [15]	Serotype 18C; OPA; PCV20 PPS	18.6 (15.3–22.7)	22.5 (18.3–27.6)	0.775 (0.645–0.935)	Figure 5B
PCV20 [15]	Serotype 19A; OPA; PCV20 PPS	8.2 (7.0–9.7)	10.5 (8.8–12.6)	0.719 (0.621–0.833)	Figure 5B
PCV20 [15]	Serotype 19F; OPA; PCV20 PPS	9.2 (7.8–10.9)	11.6 (9.6–13.9)	0.752 (0.637–0.893)	Figure 5B
PCV20 [15]	Serotype 22F; OPA; PCV20 PPS	23.5 (19.7–28.2)	29.5 (24.3–35.8)	0.746 (0.625–0.893)	Figure 5B
PCV20 [15]	Serotype 23F; OPA; PCV20 PPS	23.1 (19.1–27.9)	28.7 (23.6–35.0)	0.735 (0.585–0.917)	Figure 5B
PCV20 [15]	Serotype 33F; OPA; PCV20 PPS	4.7 (4.1–5.2)	6.3 (5.5–7.2)	0.714 (0.610–0.833)	Figure 5B

MGI, mean geometric increase; GMFR, geometric mean fold rise; GMR, geometric mean ratio; PPS, per-protocol set. MGIs and GMFRs describe within-participant post-/pre-vaccination changes. Adjusted GMRs compare coadministration with sequential administration at the post-vaccination assessment, using the original trial models. Values retain the precision reported in the source; GMRs are expressed as coadministration/sequential. Sampling was approximately 1 month after the relevant vaccine dose, with the RZV anti-gE assessment 1 month after dose 2. Trial-specific population and assay counts are detailed in Table S8. OPA, opsonophagocytic assay; HI, hemagglutination inhibition. The adjuvanted-QIV A/Darwin microneutralization result is a separate post hoc assay. The seasonal-QIV influenza MGIs are displayed without numeric labels in the original Supplementary Figure 3C; its trial-level HI GMRs are reported in Table S12.

Table S16. Trial-level construction of the 30-day unsolicited adverse-event outcome.

Product / trial	Coadministration n/N	Sequential n/N	Coadministration windows	Sequential windows	Original report location
PCV20 [15]<br>NCT05879107	60/553	98/557	Days 1–30	Days 1–30 and 31–60	PDF pp.5–6 Results; PDF p.6 Results
COVID-19 XBB.1.5 [16]<br>NCT06374394	87/417	108/416	Days 1–30	Days 1–30 and 31–60	PDF p.10 Table 2
RZV [17]<br>NCT05966090	62/265	80/265	Days 1–30 and 61–90	Days 1–30, 31–60, and 61–90	PDF p.9 Table 2
Seasonal QIV [18]<br>NCT04841577	83/442	105/443	Days 1–30	Days 1–30 and 31–60	PDF p.6 Table 3
High-dose QIV [19]<br>NCT05559476	63/516	69/513	Days 1–30	Days 1–30 and 31–60	PDF pp.12–13 Results

Product / trial	Coadministration n/N	Sequential n/N	Coadministration windows	Sequential windows	Original report location
Adjuvanted QIV [20] NCT05568797	71/523	128/522	Days 1–30	Days 1–30 and 31–60	PDF p.10 Table 3
Total	426/2716	588/2716	—	—	—

Numerators are trial-reported participants with at least one unsolicited adverse event across the assigned schedule; denominators are exposed-set participants. Each participant contributes once to this outcome within the assigned schedule. Day ranges follow the planned vaccination schedules and include the vaccination day and the following 29 days. The windows differ in cumulative duration between schedules.

Table S17. Joint model and sensitivity results

Analysis	ρ	Trials / rows	GMR (95% CI)	df	Inference
Joint RSV	0	6 / 12	0.907 (0.840–0.980)	4.906	CR2
Joint RSV	0.5	6 / 12	0.908 (0.841–0.981)	4.819	CR2
Joint RSV	0.8	6 / 12	0.908 (0.840–0.980)	4.819	CR2
Study composite	0	6 / 6	0.907 (0.840–0.979)	5.000	PM / HK
Study composite	0.5	6 / 6	0.905 (0.839–0.977)	5.000	PM / HK
Study composite	0.8	6 / 6	0.899 (0.833–0.970)	5.000	PM / HK
Fixed GLS	0.5	6 / 12	0.906 (0.864–0.951)	—	Wald z
Omit NCT05879107	0.5	5 / 10	0.893 (0.817–0.977)	3.847	CR2
Omit NCT06374394	0.5	5 / 10	0.907 (0.819–1.005)	3.874	CR2
Omit NCT05966090	0.5	5 / 10	0.903 (0.823–0.992)	3.976	CR2
Omit NCT04841577	0.5	5 / 10	0.933 (0.897–0.970)	3.805	CR2
Omit NCT05559476	0.5	5 / 10	0.906 (0.817–1.005)	3.880	CR2
Omit NCT05568797	0.5	5 / 10	0.902 (0.812–1.001)	3.895	CR2
Exclude two RSV-B rows	0.5	6 / 10	0.922 (0.853–0.998)	4.714	CR2

GMRs are coadministration/sequential. Joint and exclusion analyses retain six independent trials; leave-one-study-out analyses contain five. Composite analyses contain one estimate per trial. Degrees of freedom are Satterthwaite for CR2 and five for Hartung–Knapp. The fixed GLS interval uses a normal approximation. Trial registration numbers identify the omitted trial. CR2, bias-reduced cluster-robust variance; PM, Paule–Mandel; HK, Hartung–Knapp; GLS, generalized least squares.

Table S18. Sensitivity analyses for deaths through study end.

Analysis	Effect measure	Trials, n	Estimate (95% CI)
Fixed correction 0.5 (primary)	RR	6	0.475 (0.174, 1.297)
Fixed correction 0.1	RR	6	0.468 (0.164, 1.341)
Addition of 0.5 to all cells	RD, percentage points	6	-0.175 (-0.642, 0.292)

REML models with Hartung–Knapp intervals. Corrections were added to all four cells in studies containing zero cells for RR analyses and in all studies for the RD analysis.

Table S19. GRADE evidence profile for product-specific partner-vaccine antibody endpoints

Product and antibody endpoint	PPS n<br>Co-Ad/Seq	GMR (95% CI)	Risk of bias	Imprecision	GRADE certainty
COVID-19 mRNA, SARS-CoV-2 XBB.1.5 (NT50)	380/381	0.763 (0.662–0.885)	Serious (−1): PPS selection and unresolved missing antibody outcomes.	Not serious: CI below 1.	Moderate
RZV, VZV anti-gE (ELISA)	198/170 (PPS cohort)	0.833 (0.714–0.909)	Very serious (−2): 198/265 and 170/265 in the HZ PPS; 27/60 schedule exclusions and 27/30 laboratory exclusions, with overlapping reasons and no broader anti-gE analysis.	Not serious for response direction: 95% CI 0.714–0.909 lies below 1; endpoint PPS cohort n = 368.	Low
Seasonal QIV, A/H1N1 HI	427/410 (post-dose PPS)	0.820 (0.694–0.971)	Serious (−1): PPS selection and possible missing antibody outcomes.	Not serious: CI below 1.	Moderate

Product and antibody endpoint	PPS n<br>Co-Ad/Seq	GMR (95% CI)	Risk of bias	Imprecision	GRADE certainty
Seasonal QIV, A/H3N2 HI	427/410 (post-dose PPS)	0.855 (0.741–0.980)	Serious (−1): PPS selection and possible missing antibody outcomes.	Not serious: CI below 1.	Moderate
Seasonal QIV, B/Victoria HI	427/410 (post-dose PPS)	0.909 (0.794–1.053)	Serious (−1): PPS selection and possible missing antibody outcomes.	Serious (−1): CI includes lower and higher antibody responses.	Low
Seasonal QIV, B/Yamagata HI	427/410 (post-dose PPS)	0.855 (0.758–0.962)	Serious (−1): PPS selection and possible missing antibody outcomes.	Not serious: CI below 1.	Moderate
High-dose QIV, A/H1N1 HI	452/435	1.075 (0.926–1.250)	Serious (−1): PPS selection and possible missing antibody outcomes.	Serious (−1): CI includes lower and higher antibody responses.	Low
High-dose QIV, A/H3N2 HI	458/441	1.020 (0.877–1.190)	Serious (−1): PPS selection and possible missing antibody outcomes.	Serious (−1): CI includes lower and higher antibody responses.	Low
High-dose QIV, B/Victoria HI	458/441	1.053 (0.971–1.136)	Serious (−1): PPS selection and possible missing antibody outcomes.	Serious (−1): CI includes lower and higher antibody responses.	Low
High-dose QIV, B/Yamagata HI	456/441	1.087 (0.980–1.190)	Serious (−1): PPS selection and possible missing antibody outcomes.	Serious (−1): CI includes lower and higher antibody responses.	Low
Adjuvanted QIV, A/H1N1 HI	440/399 (post-dose PPS)	0.962 (0.847–1.099)	Serious (−1): PPS selection and unresolved missing antibody outcomes.	Serious (−1): CI includes lower and higher antibody responses.	Low
Adjuvanted QIV, A/Darwin H3N2 HI	442/400 (post-dose PPS)	0.758 (0.654–0.885)	Serious (−1): PPS selection and unresolved missing antibody outcomes.	Not serious: CI below 1.	Moderate

Product and antibody endpoint	PPS n<br>Co-Ad/Seq	GMR (95% CI)	Risk of bias	Imprecision	GRADE certainty
Adjuvanted QIV, B/Victoria HI	442/400 (post-dose PPS)	1.031 (0.943–1.111)	Serious (−1): PPS selection and unresolved missing antibody outcomes.	Serious (−1): CI includes lower and higher antibody responses.	Low
Adjuvanted QIV, B/Yamagata HI	442/400 (post-dose PPS)	0.962 (0.885–1.053)	Serious (−1): PPS selection and unresolved missing antibody outcomes.	Serious (−1): CI includes lower and higher antibody responses.	Low
PCV20 OPA serotype 1	514/497 (cohort)	0.68 (0.55, 0.82)	Serious (−1): PPS selection and possible missing antibody outcomes.	Not serious: CI below 1.	Moderate
PCV20 OPA serotype 3	514/497 (cohort)	0.76 (0.65, 0.89)	Serious (−1): PPS selection and possible missing antibody outcomes.	Not serious: CI below 1.	Moderate
PCV20 OPA serotype 4	514/497 (cohort)	0.75 (0.63, 0.89)	Serious (−1): PPS selection and possible missing antibody outcomes.	Not serious: CI below 1.	Moderate
PCV20 OPA serotype 5	514/497 (cohort)	0.73 (0.58, 0.93)	Serious (−1): PPS selection and possible missing antibody outcomes.	Not serious: CI below 1.	Moderate
PCV20 OPA serotype 6A	514/497 (cohort)	0.73 (0.59, 0.89)	Serious (−1): PPS selection and possible missing antibody outcomes.	Not serious: CI below 1.	Moderate
PCV20 OPA serotype 6B	514/497 (cohort)	0.71 (0.59, 0.85)	Serious (−1): PPS selection and possible missing antibody outcomes.	Not serious: CI below 1.	Moderate
PCV20 OPA serotype 7F	514/497 (cohort)	0.75 (0.65, 0.86)	Serious (−1): PPS selection and possible missing antibody outcomes.	Not serious: CI below 1.	Moderate

**Product and antibody endpoint**	**PPS n Co-Ad/Seq**	**GMR (95% CI)**	**Risk of bias**	**Imprecision**	**GRADE certainty**
PCV20 OPA serotype 8	514/497 (cohort)	0.72 (0.61, 0.85)	Serious (−1): PPS selection and possible missing antibody outcomes.	Not serious: CI below 1.	Moderate
PCV20 OPA serotype 9V	514/497 (cohort)	0.81 (0.68, 0.95)	Serious (−1): PPS selection and possible missing antibody outcomes.	Not serious: CI below 1.	Moderate
PCV20 OPA serotype 10A	514/497 (cohort)	0.82 (0.69, 0.97)	Serious (−1): PPS selection and possible missing antibody outcomes.	Not serious: CI below 1.	Moderate
PCV20 OPA serotype 11A	514/497 (cohort)	0.85 (0.70, 1.02)	Serious (−1): PPS selection and possible missing antibody outcomes.	Serious (−1): CI includes lower and higher antibody responses.	Low
PCV20 OPA serotype 12F	514/497 (cohort)	0.83 (0.69, 1.01)	Serious (−1): PPS selection and possible missing antibody outcomes.	Serious (−1): CI includes lower and higher antibody responses.	Low
PCV20 OPA serotype 14	514/497 (cohort)	0.70 (0.60, 0.83)	Serious (−1): PPS selection and possible missing antibody outcomes.	Not serious: CI below 1.	Moderate
PCV20 OPA serotype 15B	514/497 (cohort)	0.69 (0.57, 0.82)	Serious (−1): PPS selection and possible missing antibody outcomes.	Not serious: CI below 1.	Moderate
PCV20 OPA serotype 18C	514/497 (cohort)	0.78 (0.65, 0.94)	Serious (−1): PPS selection and possible missing antibody outcomes.	Not serious: CI below 1.	Moderate
PCV20 OPA serotype 19A	514/497 (cohort)	0.72 (0.62, 0.83)	Serious (−1): PPS selection and possible missing antibody outcomes.	Not serious: CI below 1.	Moderate

Product and antibody endpoint	PPS n<br>Co-Ad/Seq	GMR (95% CI)	Risk of bias	Imprecision	GRADE certainty
PCV20 OPA serotype 19F	514/497 (cohort)	0.75 (0.64, 0.89)	Serious (−1): PPS selection and possible missing antibody outcomes.	Not serious: CI below 1.	Moderate
PCV20 OPA serotype 22F	514/497 (cohort)	0.75 (0.62, 0.89)	Serious (−1): PPS selection and possible missing antibody outcomes.	Not serious: CI below 1.	Moderate
PCV20 OPA serotype 23F	514/497 (cohort)	0.73 (0.58, 0.92)	Serious (−1): PPS selection and possible missing antibody outcomes.	Not serious: CI below 1.	Moderate
PCV20 OPA serotype 33F	514/497 (cohort)	0.71 (0.61, 0.83)	Serious (−1): PPS selection and possible missing antibody outcomes.	Not serious: CI below 1.	Moderate

Each row evaluates the specified antibody outcome for the vaccine product and schedule studied. Imprecision was assessed using the confidence interval and endpoint-specific information, with GMR = 1 as the reference; one level was deducted when the interval included both lower and higher responses. Risk-of-bias judgments considered postrandomization exclusions, missing antibody measurements and reported exposed-set analyses. RZV anti-gE was downgraded two levels for risk of bias because 368 of 530 randomized participants contributed to the HZ per-protocol analysis, with substantial schedule-related and laboratory exclusions. Other partner-antibody outcomes were downgraded one level for risk of bias. Trial-specific non-inferiority conclusions are presented in Table S12. Inconsistency is not downgraded within a single-trial product/endpoint comparison. Indirectness is not downgraded for the measured laboratory outcome in the evaluated product and schedule. Publication bias is not additionally downgraded after considering registration, reported primary failures and sponsorship. Cohort, post-dose and model counts retain their original definitions (Table S8); participants are not added across serotypes. PPS, per-protocol set; GMR, geometric mean ratio, Co-Ad/Seq. Sources: PCV20 [15]; COVID-19 [16]; RZV [17]; seasonal QIV [18]; high-dose QIV [19]; adjuvanted QIV [20].

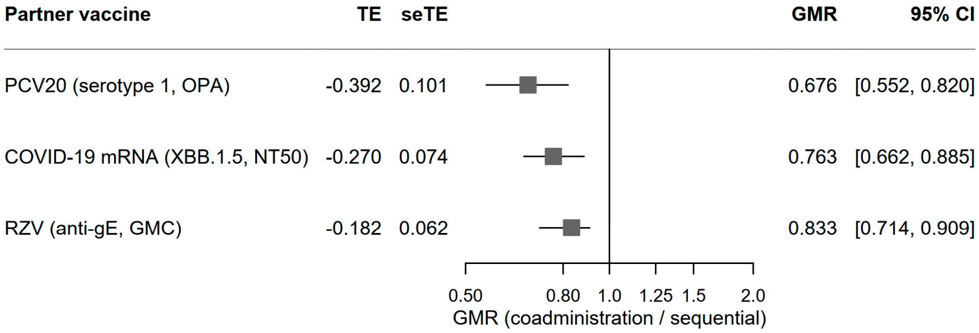

Figure S1. Non-influenza partner-vaccine antibody responses 1 month after the final partner-vaccine dose in the trial-specific per-protocol populations: coadministration versus sequential administration. Single-study estimates are shown for PCV20 (serotype 1, opsonophagocytic assay [OPA]), the COVID-19 mRNA vaccine (SARS-CoV-2 Omicron XBB.1.5, 50% neutralizing titre [NT50]) and RZV (varicella-zoster virus anti-gE, geometric mean concentration [GMC]); different vaccines and assays were not pooled. GMRs were 0.676 (95% CI 0.552–0.820) for PCV20 serotype 1, 0.763 (0.662–0.885) for SARS-CoV-2 XBB.1.5 and 0.833 (0.714–0.909) for RZV anti-gE. Squares and horizontal lines show GMRs and 95% CIs; GMR < 1 indicates a lower response with coadministration. Study sources: PCV20 (Leroux-Roels 2026); COVID-19 mRNA (Bonten 2026); RZV (Roussy 2026). TE, log(GMR); seTE, standard error of TE.

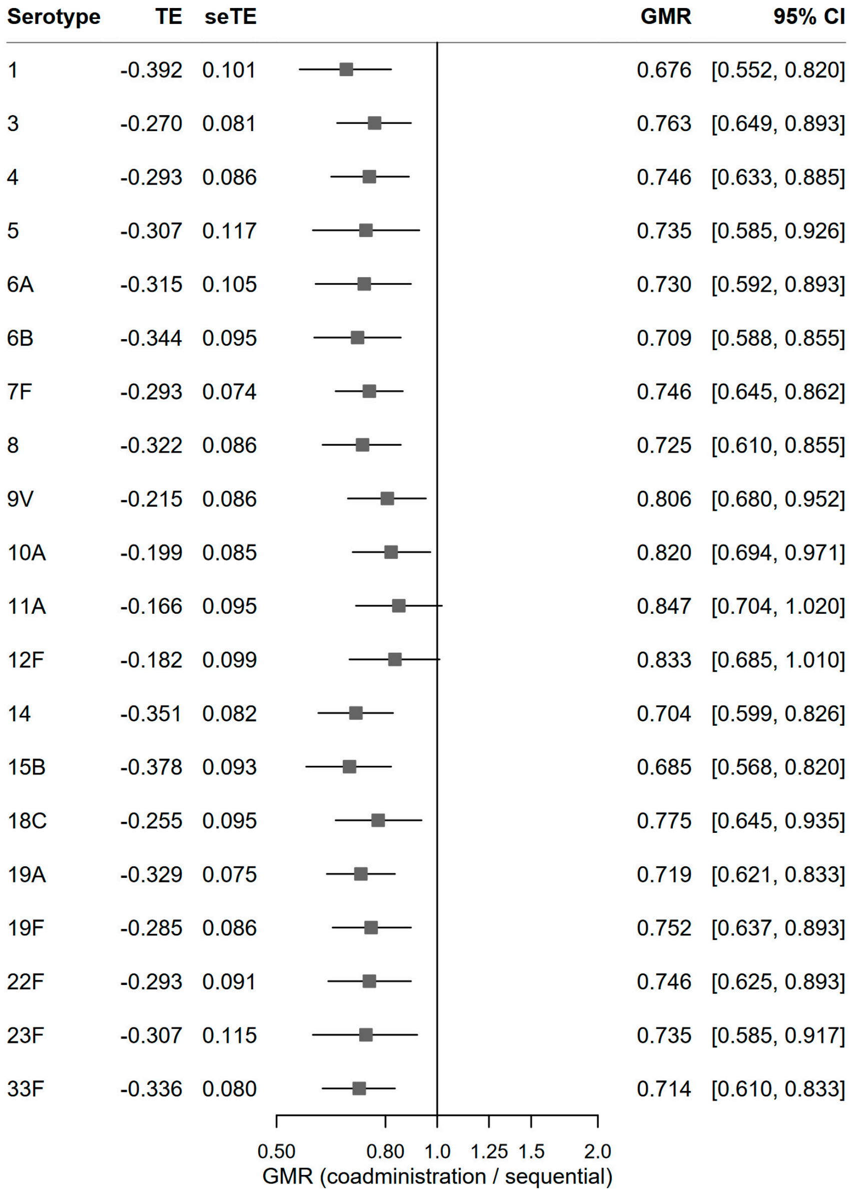

Figure S2. PCV20 serotype-specific opsonophagocytic antibody responses 1 month after vaccination in the per-protocol population (Leroux-Roels 2026, single study): coadministration versus sequential administration. GMRs (95% CIs) are shown for all 20 pneumococcal serotypes, which were not pooled. GMRs ranged from 0.676 (95% CI 0.552–0.820; serotype 1) to 0.847 (0.704–1.020; serotype 11A), and all lower 95% confidence limits exceeded the trial-specific non-inferiority boundary of 0.5. Squares and horizontal lines show GMRs and 95% CIs; GMR < 1 indicates a lower response with coadministration. TE, log(GMR); seTE, standard error of TE.

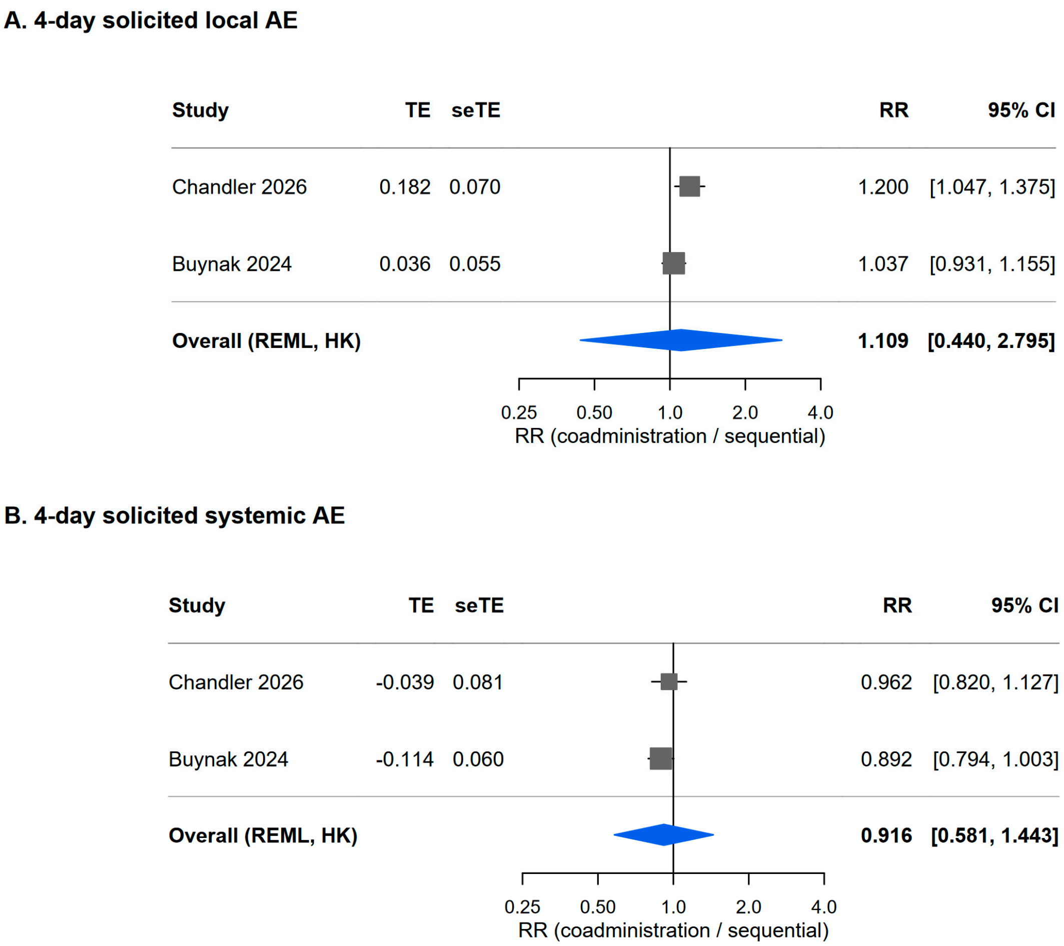

Figure S3. Solicited reactions within 4 days after vaccination: (A) local; (B) systemic. Two trials reported 4-day solicited reactions (Chandler 2026; Buynak 2024). Squares and horizontal lines show study-specific RRs and 95% CIs; diamonds show pooled random-effects estimates (REML, Hartung–Knapp): local RR 1.109 (95% CI 0.440–2.795); systemic RR 0.916 (0.581–1.443). Reactions were recorded across one coadministration visit or two sequential vaccination visits, and the 4-day window was not combined with other solicited-reaction windows. RR < 1 indicates fewer reactions with coadministration. TE, log(RR); seTE, standard error of TE.

A. Study non-completion for any reason

Study	Co-Ad n/N	Seq n/N	RR	95% CI
Leroux-Roels 2026	14/553	26/557	0.542	[0.286, 1.028]
Bonten 2026	8/417	18/416	0.443	[0.195, 1.008]
Roussy 2026	9/265	10/265	0.900	[0.372, 2.179]
Chandler 2026	13/442	26/443	0.501	[0.261, 0.962]
Buynak 2024	23/516	28/513	0.817	[0.477, 1.398]
Clark 2024	5/523	23/522	0.217	[0.083, 0.566]
**Overall (REML, HK)**			**0.557**	**[0.349, 0.889]**

k = 6; I² = 22.3%

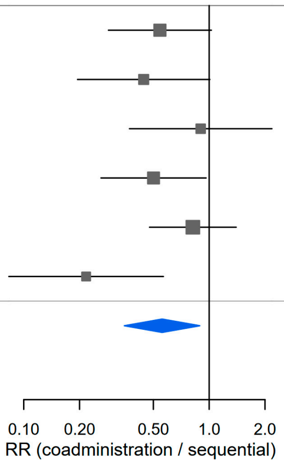

B. Study completion

Study	Co-Ad n/N	Seq n/N	RR	95% CI
Leroux-Roels 2026	539/553	531/557	1.022	[0.999, 1.046]
Bonten 2026	409/417	398/416	1.025	[1.000, 1.051]
Roussy 2026	256/265	255/265	1.004	[0.971, 1.037]
Chandler 2026	429/442	417/443	1.031	[1.002, 1.061]
Buynak 2024	493/516	485/513	1.011	[0.983, 1.039]
Clark 2024	518/523	499/522	1.036	[1.015, 1.057]
**Overall (REML, HK)**			**1.024**	**[1.012, 1.036]**

k = 6; I² = 0.0%

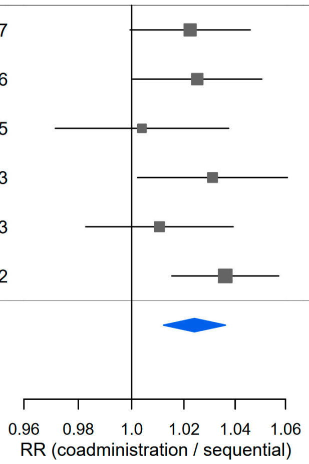

Figure S4. Study completion status: (A) non-completion for any reason; (B) completion. All six trials contributed to each panel. Squares and horizontal lines show study-specific RRs and 95% CIs; diamonds show pooled random-effects estimates (REML, Hartung–Knapp): non-completion RR 0.557 (95% CI 0.349–0.889; I² 22.3%); completion RR 1.024 (1.012–1.036; I² 0.0%). n/N, participants with the outcome/participants analysed. RR < 1 for non-completion and RR > 1 for completion indicate higher retention with coadministration. These outcomes describe overall study retention and are reported separately from adverse-event outcomes. Co-Ad, coadministration; Seq, sequential administration; TE, log(RR); seTE, standard error of TE.

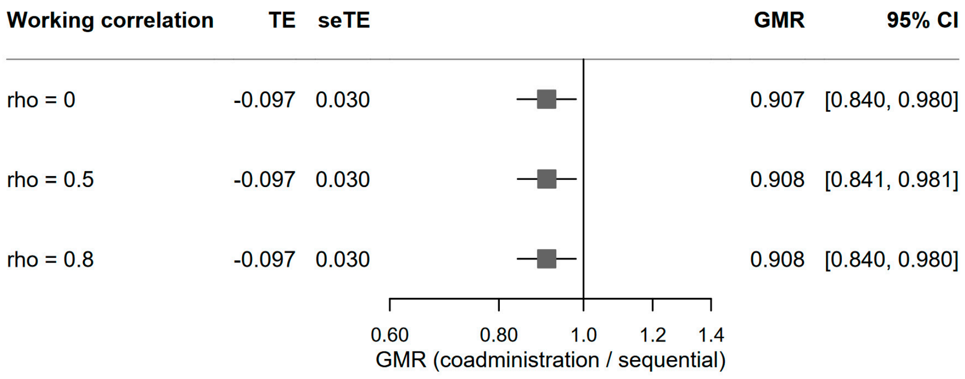

Figure S5. Sensitivity of the exploratory joint RSV neutralizing-antibody estimate to the assumed within-study working correlation (rho). Rows are model estimates (not study rows) from the three-level REML model with CR2/Satterthwaite inference at rho = 0, 0.5 and 0.8: GMRs were 0.907 (95% CI 0.840–0.980), 0.908 (0.841–0.981) and 0.908 (0.840–0.980), respectively; no model is refit at the graphics stage. Squares and horizontal lines show GMRs and 95% CIs. TE, log(GMR); seTE, CR2 standard error of TE.

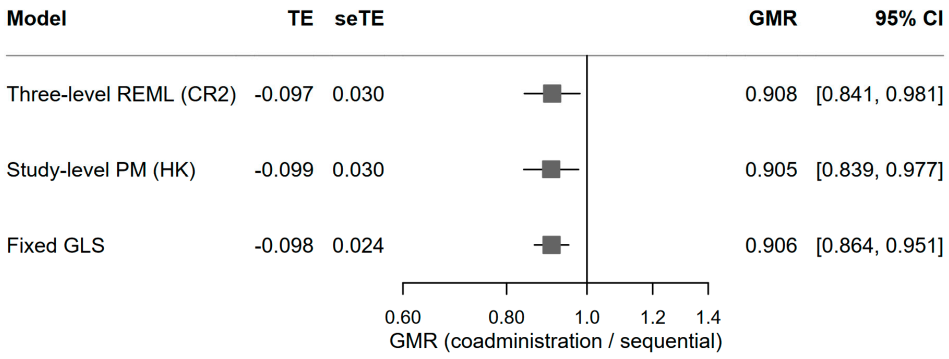

Figure S6. Model sensitivity of the exploratory joint RSV neutralizing-antibody estimate at rho = 0.5. Rows are model estimates (not study rows). Squares and horizontal lines show GMRs and 95% CIs: three-level REML with CR2/Satterthwaite inference, 0.908 (95% CI 0.841–0.981); study-level composites with the Paule–Mandel estimator and Hartung–Knapp adjustment (PM/HK), 0.905 (0.839–0.977); fixed-effects generalized least squares (GLS), 0.906 (0.864–0.951). TE, log(GMR); seTE, standard error of TE.

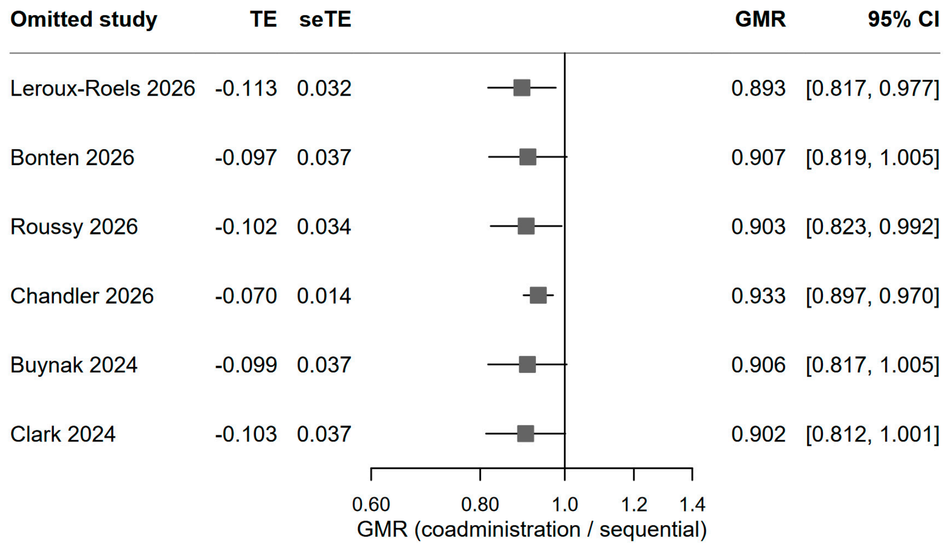

Figure S7. Leave-one-study-out sensitivity analysis of the exploratory joint RSV neutralizing-antibody estimate. Each row is the three-level REML (rho = 0.5, CR2/Satterthwaite) estimate after omitting the indicated trial; rows are model estimates, not study rows. Squares and horizontal lines show GMRs and 95% CIs. GMRs ranged from 0.893 (95% CI 0.817–0.977; omitting Leroux-Roels 2026) to 0.933 (0.897–0.970; omitting Chandler 2026); three of the six 95% CIs included 1. TE, log(GMR); seTE, CR2 standard error of TE.

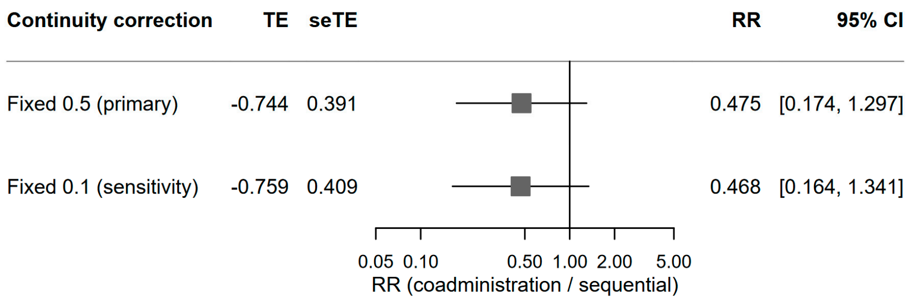

Figure S8. Continuity-correction sensitivity analysis for deaths through study end. Fixed corrections of 0.5 (primary) or 0.1 (sensitivity) were added to all four cells of the 2 × 2 table in trials containing zero cells; all six trials contributed. Squares and horizontal lines show pooled RRs and Hartung–Knapp 95% CIs: RR 0.475 (95% CI 0.174–1.297) with the 0.5 correction and RR 0.468 (0.164–1.341) with the 0.1 correction; wide confidence intervals reflect sparse events. TE, log(RR); seTE, standard error of TE. The corresponding risk-difference analysis is reported in Table S18.

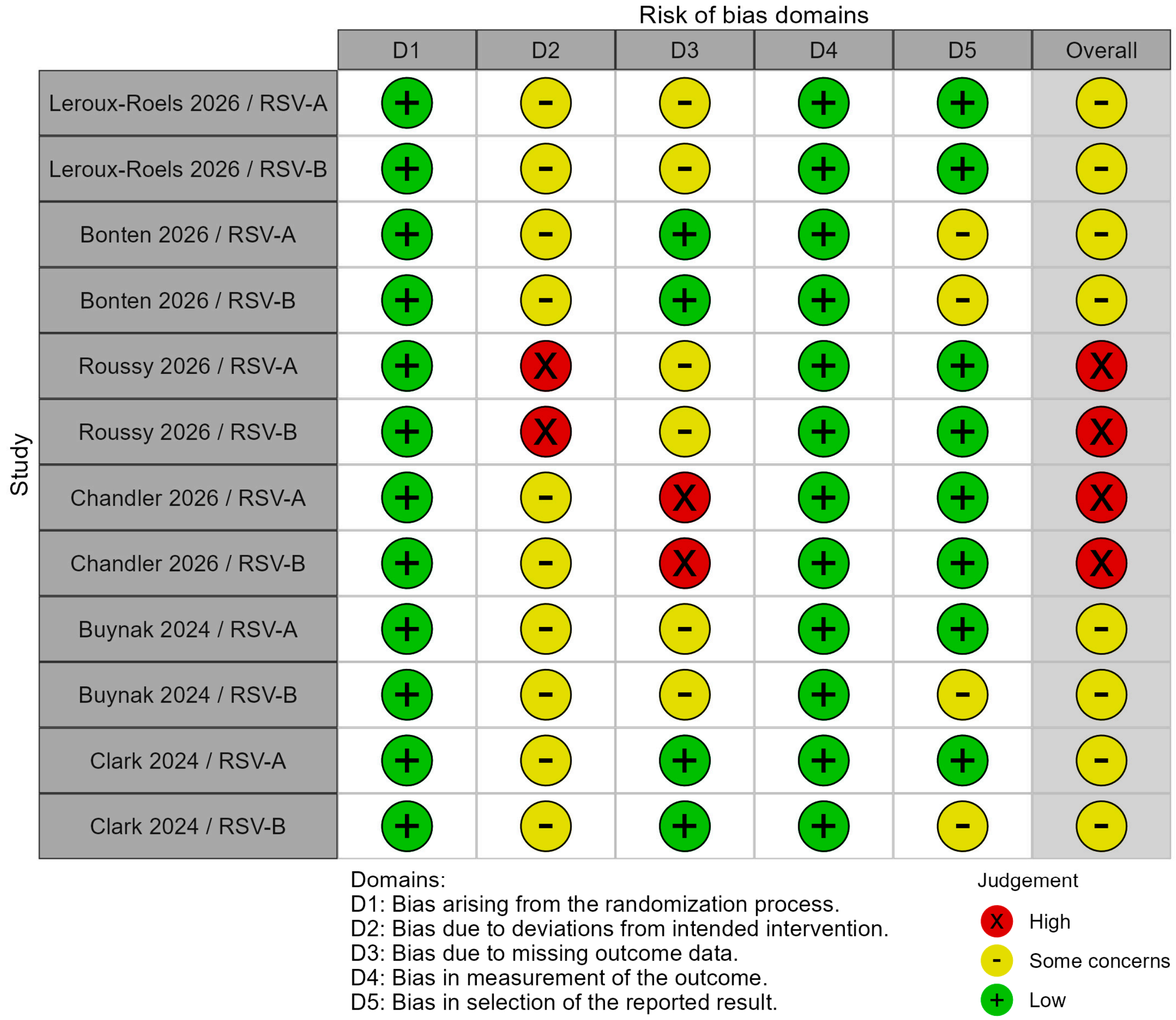

Figure S9. Risk-of-bias assessments for RSV-A and RSV-B neutralizing-antibody outcomes using RoB 2. Rows show outcome-level judgments for each trial; columns show the five RoB 2 domains (D1, bias arising from the randomization process; D2, bias due to deviations from intended interventions; D3, bias due to missing outcome data; D4, bias in measurement of the outcome; D5, bias in selection of the reported result) and the overall judgment. Green plus, low risk; yellow minus, some concerns; red cross, high risk.

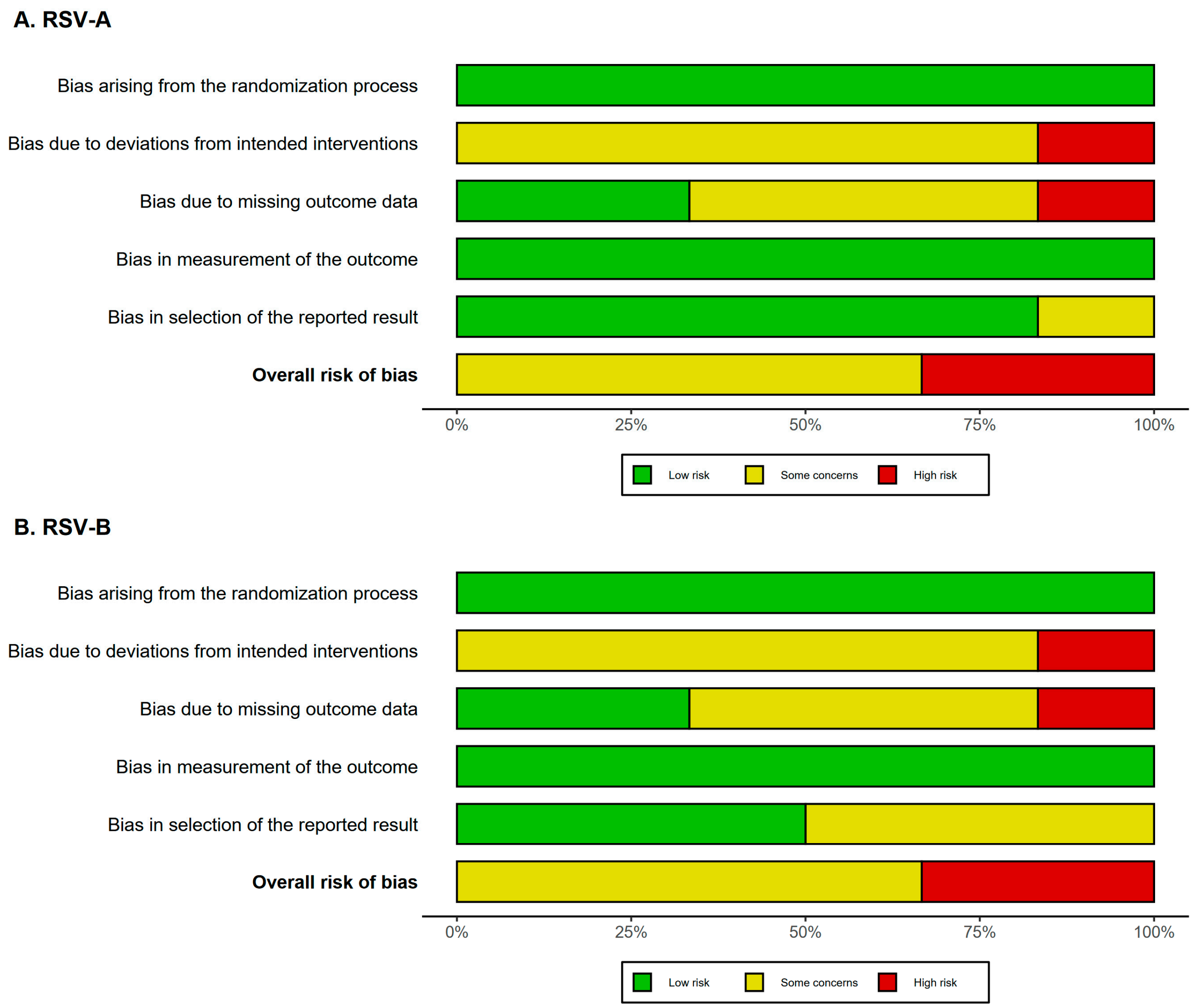

Figure S10. Distribution of RoB 2 judgments for RSV-A (A) and RSV-B (B) neutralizing-antibody outcomes across the six trials. Bars show the percentage of judgments rated low risk (green), some concerns (yellow) or high risk (red) within each domain and overall.
